# Exosomal circRNAs in the plasma serve as novel biomarkers for IPF diagnosis and progression prediction

**DOI:** 10.1186/s12967-024-05034-9

**Published:** 2024-03-10

**Authors:** Wenhua Gan, Wenwen Song, Yujuan Gao, Xuexue Zheng, Fengjuan Wang, Zirui Zhang, Ke Zen, Hongwei Liang, Xin Yan

**Affiliations:** 1 Department of Emergency, Nanjing Drum Tower Hospital, School of Life Science and Technology, China Pharmaceutical University, Nanjing, 210009 China; 2 Department of Respiratory and Critical Care Medicine, Nanjing Drum Tower Hospital, The Affiliated Hospital of China Pharmaceutical University, Nanjing, 210008 China; 3 Department of Thoracic Surgery, Medical School, Nanjing Drum Tower Hospital, Nanjing University, Nanjing, 210008 China

**Keywords:** Idiopathic pulmonary fibrosis, Exosome, Circular RNA, Biomarker

## Abstract

**Background:**

Idiopathic Pulmonary Fibrosis (IPF) is a type of chronic interstitial pneumonia, often fatal, with elusive causes and a bleak prognosis. Its treatment options are limited and largely ineffective. Early detection and precise diagnosis are pivotal in managing the disease effectively and enhancing patient survival rates. Recently, the quest for trustworthy biomarkers for IPF has gained momentum. Notably, emerging studies indicate that circular RNAs (circRNAs) found in exosomes may hold significant potential as valuable diagnostic markers.

**Methods:**

In this study, we initially explored the expression profile of circRNAs in exosomes sourced from the blood of IPF patients and healthy volunteers, employing a human circRNA microarray. We then utilized RT-qPCR to corroborate the dysregulated circRNAs identified by the microarray during the training phase. Next, the circRNAs that displayed a significant increase during the training phase were selected for further validation in a larger cohort encompassing 113 IPF patients and 76 healthy volunteers. Ultimately, the expression level and function of hsa_circ_0044226 were substantiated through a series of in vivo and in vitro experiments.

**Results:**

Utilizing a human circRNA microarray, we identified 11 dysregulated circRNAs in the exosomes derived from the blood of IPF patients and control volunteers. Subsequent RT-qPCR analysis revealed significant increases in three circRNAs (hsa_circ_0044226, hsa_circ_0004099, hsa_circ_0008898) within the IPF patients. Notably, hsa_circ_0044226 was markedly elevated in patients experiencing acute exacerbation of IPF (AE-IPF) compared to those with stable IPF (S-IPF). Additionally, an upregulation of hsa_circ_0044226 was observed in the blood exosomes derived from a bleomycin-induced IPF mouse model.

**Conclusion:**

The expression levels of hsa_circ_0044226, hsa_circ_0004099, and hsa_circ_0008898 in plasma exosomes introduce a new paradigm of biomarkers for the diagnosis and progression of IPF.

**Supplementary Information:**

The online version contains supplementary material available at 10.1186/s12967-024-05034-9.

## Introduction

Idiopathic Pulmonary Fibrosis (IPF) is a progressive and fatal interstitial lung disease, characterized by an unknown cause and a median survival of 3–5 years. It is characterized by excessive collagen deposition and the development of fibrotic lesions throughout the pulmonary interstitium [1]. Despite advancements in understanding its pathogenesis and treatment, the incidence of IPF has been steadily increasing over the past few decades, and prognosis remains poor [2]. The clinical management of IPF is challenging due to the lack of precise diagnostic and progression indicators, as well as simple short-term treatment response biomarkers [3]. Therefore, there is an urgent need to develop more effective and non-invasive methods for the early detection and prediction of IPF progression to improve patient outcomes and treatment strategies.

Exosomes, which are small extracellular vesicles secreted by nearly all cells, play a significant role in intercellular communication [4]. They contain various biomolecules, such as proteins, lipids, nucleic acids, and noncoding RNAs, including long noncoding RNAs (lncRNA), microRNAs (miRNAs), and circular RNAs (circRNAs) [4, 5]. These exosome-derived inclusions have been implicated in the development and progression of various diseases, and can be found in biological fluids like urine, blood, saliva, and breast milk [4]. Recent evidence suggests that circRNAs in exosomes could serve as valuable diagnostic indicators for diseases like cancers, neurological diseases, and cardiovascular diseases [4]. CircRNAs are a novel class of noncoding RNA with a unique covalent loop structure that provides them with unusual stability [4, 6]. They have been found to interact with proteins, microRNAs, and can even be translated into peptides or proteins, playing a role in various human diseases [6] including idiopathic pulmonary fibrosisetc [7–13]. However, there is currently no research on the function and application of exosomal circRNAs in idiopathic pulmonary fibrosis (IPF). Therefore, identifying the differentially-expressed exosomal circRNAs in IPF could provide novel targets and valuable insights for diagnosing this disease.

In this study, we utilized human circRNA microarray and RT-qPCR techniques to identify novel biomarkers for early diagnosis and progression prediction in patients with IPF. Initially, we investigated the expression profile of circRNAs in exosomes derived from the blood of both IPF patients and control volunteers. We identified six upregulated exosomal circRNAs in IPF patients. Next, we validated the expression levels of these six circRNAs using RT-qPCR in a cohort of 10 IPF patients and 10 control volunteers. Three circRNAs, namely hsa_circ_0044226, hsa_circ_0004099, and hsa_circ_0008898, were found to be significantly increased in IPF patients. These three exosomal circRNAs were further validated in a larger cohort of 113 IPF patients and 76 control volunteers. Importantly, these three circRNAs were consistently upregulated in IPF patients, suggesting their potential as diagnostic biomarkers for early detection of IPF. Additionally, the exosomal hsa_circ_0044226 exhibited higher expression levels in patients with acute exacerbation of IPF (AE-IPF) compared to patients with stable IPF (S-IPF), indicating its potential as a marker for predicting IPF progression.

## Materials and methods

### Study population

A total of 189 plasma samples were collected from 113 IPF patients and 76 control volunteers at the Department of Respiration and Critical Care Medicine of Nanjing Drum Tower in China. The diagnosis of IPF was established based on internationally recognized guidelines from the ATS 1 [14]. This diagnosis involved a comprehensive assessment, including respiratory function tests, HRCT scans showing a probable UIP pattern, bronchoalveolar lavage (when available), and consideration of the patient's clinical history. The patient characteristics are summarized in Table 1. The study was approved by the institutional ethics committee of the Drum Tower Hospital of Nanjing University, and all subjects provided written informed consent to participate.

**Table 1:** Basic characteristics of the participants in the validation cohort

	Control Volunteers (n = 76)	IPF (n = 113)	*P* value
Age (years)	63.24 ± 10.49	63.68 ± 6.34	0.72
Sex (male/Female)	41/35	70/43	0.17
Smoking (%)	23 (30.26)	46 (40.71)	0.09
Drinking (%)	17 (22.37)	18 (15.93)	0.17
Hypertension (%)	25 (32.89)	41 (36.28)	0.37
Diabetes (%)	12 (15.79)	22 (19.46)	0.32
Hyperlipemia (%)	12 (15.79)	15 (13.27)	0.38
BMI (Kg/m^2^)	23.46 ± 3.14	24.54 ± 3.84	0.09
SBP (mmHg)	129.46 ± 20.91	130.66 ± 16.35	0.66
DBP (mmHg)	79.36 ± 13.40	77.35 ± 10.02	0.38

*BMI* body mass index, *SBP* systolic blood pressure, *DBP* diastolic blood pressure, *IPF* idiopathic pulmonary fibrosis

### Isolation and identification of plasma exosomes

10 ml anti-coagulated blood was collected to isolate plasma (about 5 ml per sample) by centrifugation at 800 g for 30 min at 4 °C. The plasma was then centrifuged at 3000 g for 30 min and following 10,000 g for 30 min at 4 °C to remove the precipitate and retain the supernatant. Subsequently, the exosomes was isolated from the supernatant by CD63 Exosome Capture Beads (ab239686, abcam) according to the manufacturer’s instructions. The exosomes were eluted using elution buffer (0.1 M Glycine, 0.1% (v/v) Tween-20, pH 2.5), and identified by Transmission electron microscopy (TEM), Nanoparticle tracking analysis (NTA) and Western blotting according to the previous reported [15].

### RNA isolation for circRNA array and RT-qPCR

The total RNAs of exosomes and the Human fetal lung fibroblast 1 (HFL1) cell line were extracted using TRIzol reagent (Invitrogen, USA) according to the manufacturer's instructions, and the RNA concentration was measured using a NanoDrop. Exosomal RNAs from three IPF patients and control volunteers were analyzed using the Arraystar Human circRNA Array 2.0 following the manufacturer's instructions. For the detection of exosomal circRNAs, a hydrolysis probe-based RTqPCR assay was performed using a LightCycler 480 Instrument (Roche Molecular Diagnostics, Mannheim, Germany). Briefly, reverse transcription was carried out using the HiScript II Q Select RT SuperMix for qPCR kit (R232-01, Vazyme) in a reaction system containing 2 μL of extracted RNA, 3 μL of diethyl pyrocarbonate (DEPC) treated water, 4 μL of 5 × HiScript II Select qRT SuperMix, and 1 μL of random hexamers (50 ng/μL) under the following conditions: 50 °C for 15 min, 85 °C for 5 s, and then held at 4 °C. For real-time PCR, a total reaction volume of 20 μL was prepared, containing 2 μL of cDNA, 10 μL of 2 × AceQ Universal SYBR qPCR Master Mix, 0.5 μL of Forward Primer 1 (10 μM), 0.5 μL of Reverse Primer 1 (10 μM), and 7 μL of DEPC water using AceQ Universal SYBR Green qPCR Master Mix (Q511-02, Vazyme). The reactions were performed using a LightCycler 480 Instrument with the following cycling conditions: 1 cycle of 95 °C for 5 min, followed by 40 cycles of 95 °C for 10 s and 60 °C for 30 s. All reactions, including no-template controls, were performed in triplicate. The primer sequences used for the RT-qPCR are shown in Additional file 1: Table S1.

### Bleomycin-induced mouse model of IPF

Male C57BL/6 J mice aged 6–8 weeks were obtained from GemPharmatech Co., Ltd., Jiangsu, China. The mice were housed in the Experimental Animal Center of China Pharmaceutical University under controlled conditions (22–26 °C, 60% ± 2% relative humidity, 12-h light/dark cycle) with ad libitum access to food and water. Bleomycin hydrochloride was diluted in sterile saline and administered to mice via intratracheal drip at a dosage of 2 U per mouse. Control mice received saline via the same route. Blood and lung tissue samples were collected at 0, 7, 14, 21, 35, and 49 days after Bleomycin administration. The lung tissue was fixed in 10% neutral formalin fixative for 24 h and subjected to Hematoxylin and eosin (HE) staining following the manufacturer's instructions. The stained sections were scanned, and the fibrosis area was measured using statistical software to evaluate the degree of pulmonary fibrosis.

### HFL1 cell culture and transfection

HFL1 human fibroblast cells were purchased from PROCELL and cultured in Ham's F12K medium supplemented with 10% fetal bovine serum (FBS), 100 U/ml penicillin, and 100 μg/ml streptomycin. The cells were maintained at 37 °C in a humidified atmosphere containing 5% CO2. To silence hsa-circ-0044226, 40,000 HFL1 cells were seeded in each well of a 6-well plate and cultured in Ham's F12K medium supplemented with 10% FBS. The cells were then activated with 10 ng/ml TGF-β1 (Novoprotein) for 24 h. Subsequently, HFL1 cells were transfected with hsa-circ-0044226-specific siRNA (5’TGAGGTGTTGTACATGCATTATAA3’) or scramble RNA using Lipofectamine™ 3000 Transfection Reagent (Thermo Fisher Scientific, Cat#L3000-015) following the manufacturer's instructions. After 24 h, the cells were collected for RT-qPCR and Western blot analyses, according to the manufacturer's instructions. The primary antibodies used for Western blotting included α-SMA (Boster Biological Technology, Cat#BM0002), fibronectin (Cell Signaling Technology, Cat#26836S), collagen I (Cell Signaling Technology, Cat#84336), and GAPDH (Abclonal, Cat#A19056). The secondary antibodies used were goat anti-mouse IgG-HRP (Abclonal, Cat#AS003) and goat anti-rabbit IgG-HRP (abcam, Cat#ab6721).

### CCK8 assay of cell proliferation

Cell proliferation was assessed at 0, 24, 48, and 72 h post TGF-β1 activation and siRNA transfection using the CCK-8 assay. For this, 2000 cells per well were seeded in a 96-well plate, and at each time point, 10 μl of CCK-8 solution (Dojindo, Cat#CK04) was added to each well. After a 2-h incubation, the optical density (OD) value at 450 nm was measured to determine cell proliferation.

### Statistical analysis

Continuous variables were presented as mean ± standard deviation (SD). Differences between two groups were assessed using a t-test. Pearson's correlation analysis was employed to evaluate the relationship between Forced Expiratory Volume (FEV1) or Forced Vital Capacity (FVC) and hsa-circ-0044226. Receiver Operating Characteristic (ROC) analyses were conducted to determine the area under the ROC curve (AUC). Statistical significance was defined as p < 0.05. Data analysis was performed using GraphPad Prism 5 (GraphPad Software, CA, USA) and SPSS 22.0 (IBM, NY, USA).

## Results

### Isolation and identification of plasma exosomes

**Fig. 1:**
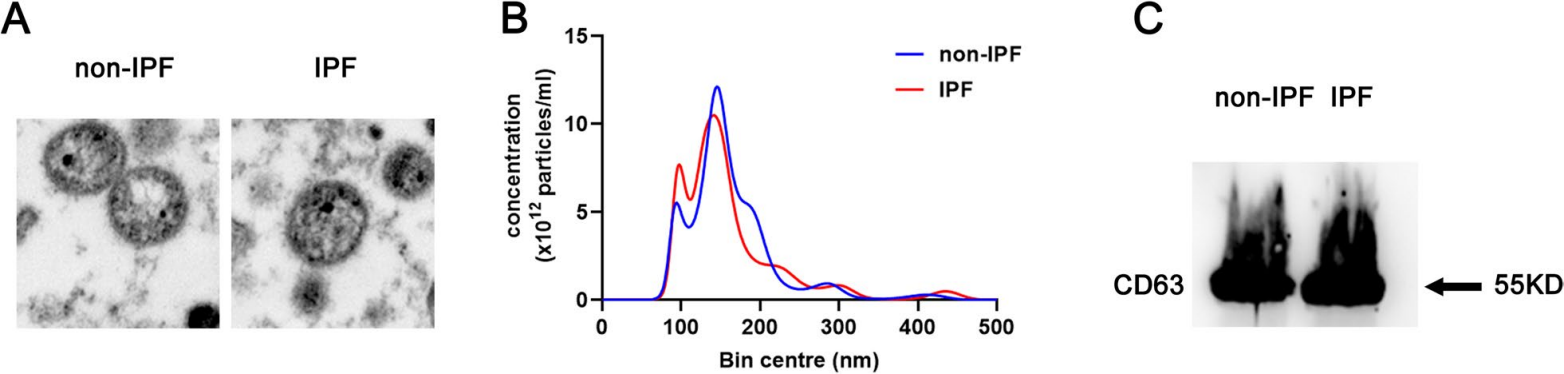
Identification of plasma exosomes. **A** Structural characteristics of exosomes under TEM. **B** Exosome diameter was detected by NTA. **C** Exosome specific protein CD63 was detected by Western blotting. *IPF* IPF patients, *non-IPF* control volunteers

Exosomes were isolated from the plasma of both IPF patients and control volunteers using CD63 Exosome Capture Beads. The isolated vesicles were then identified through TEM, NTA, and western blotting after elution. The TEM and NTA analysis revealed that the isolated vesicles had a spherical morphology with an average size distribution of 100 nm (Fig. 1A). Furthermore, the western blot analysis showed an enrichment of the exosomal marker CD63 (Fig. 1C). These results confirm the successful isolation of exosomes from the plasma. Additionally, there was no significant difference in the number and size of exosomes between IPF patients and control volunteers, as shown in Fig. 1.

### Screening the expression of circRNAs in exosomes derived from plasma of IPF patients and control volunteers

**Fig. 2:**
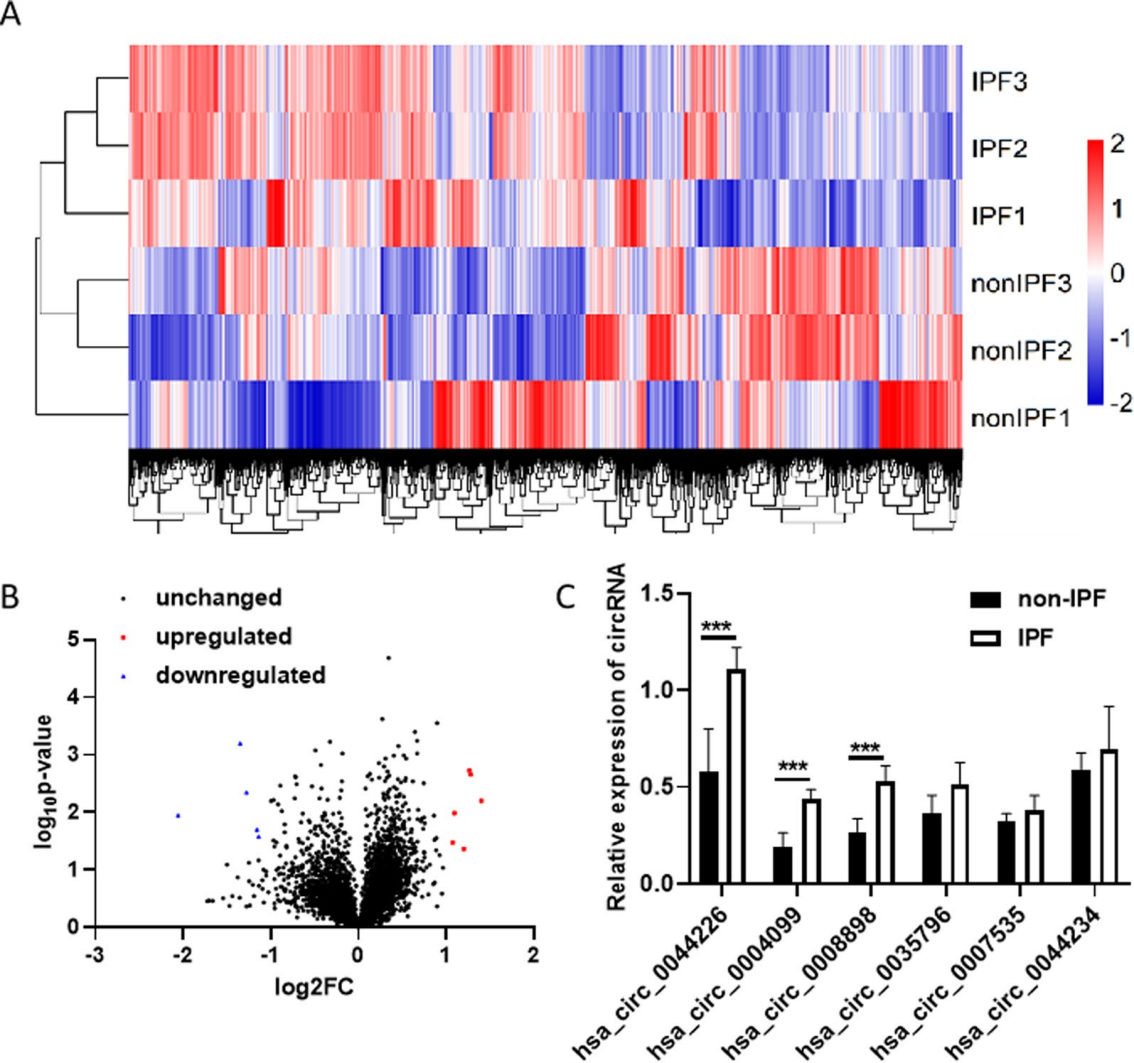
Exosomal circRNA expression profile in IPF patients and control volunteers. **A** The heat map of exosomal circRNA expression profile in IPF patients and control volunteers. **B** The volcano map of exosomal circRNA expression profile in IPF patients and control volunteers. The blue dots represent the descending circular RNA; The red dots represent rising circular RNA. **C** The six upregulated exosomal circRNAs in 10 IPF patients and 10 control volunteers by RT-qPCR. *IPF* IPF patients, *non-IPF* control volunteers. Each test was repeated three times. The mean differences between diagnostic groups were analyzed by Student’s t test. ***P < 0.001

A high-throughput circRNA microarray was conducted to identify dysregulated exosomal circRNAs in the plasma of three IPF patients and three control volunteers. The expression patterns of exosomal circRNAs in the plasma of IPF patients and control volunteers were found to have a significant difference, as confirmed by the heatmap (Fig. 2A and Additional file 1: Table S2). The volcano plot was used to visualize the differentially expressed exosomal circRNAs between IPF patients and control volunteers. The results indicated 11 dysregulated circRNAs, with six upregulated (marked by red dot) and five downregulated (marked by blue dot) circRNAs, exhibiting a fold change > 2 and p < 0.05 (Fig. 2B and Table 2). To further validate these findings, the six upregulated circRNAs were selected for individual sample analysis using RT-qPCR.

**Table 2:** Dysregulated exosomal circRNAs derived from plasma of IPF patients and control volunteers with fold change (FC) > 2 and *p* < 0.05

circRNA	Alias	circRNA_type	Gene symbol	position (genome browser link)	strand	FC(IPF vs non-IPF)	p
hsa_circRNA_102100	hsa_circ_0044226	Exonic	CDC27	chr17:45214517–45221348	−	2.644272	0.006303
hsa_circRNA_100759	hsa_circ_0004099	Exonic	DENND5A	chr11:9225206–9229179	−	2.43274	0.002181
hsa_circRNA_100705	hsa_circ_0008898	Exonic	OAT	chr10:126097110–126100769	−	2.409697	0.001874
hsa_circRNA_101550	hsa_circ_0035796	Exonic	HERC1	chr15:63988322–64008672	−	2.30596	0.043317
hsa_circRNA_102348	hsa_circ_0007535	Exonic	ELP2	chr18:33722243–33739978	+	2.135311	0.01038
hsa_circRNA_102101	hsa_circ_0044234	Exonic	CDC27	chr17:45247282–45249430	−	2.109072	0.033608
hsa_circRNA_102470	hsa_circ_0049888	Exonic	EPS15L1	chr19:16547747–16548676	−	0.454531	0.026483
hsa_circRNA_101192	hsa_circ_0005465	Exonic	CLIP1	chr12:122861935–122865105	−	0.448258	0.020135
hsa_circRNA_104780	hsa_circ_0001861	Exonic	GRHPR	chr9:37424841–37426651	+	0.412857	0.004532
hsa_circRNA_101225	hsa_circ_0029633	Exonic	ZMYM2	chr13:20625572–20641530	+	0.392345	0.000636
hsa_circRNA_102049	hsa_circ_0043278	Exonic	TADA2A	chr17:35797838–35800763	+	0.24053	0.011284

### Selecting exosomal circRNAs for qRT-PCR validation

To determine the expression levels of the selected six upregulated circRNAs (hsa_circ_0044226, hsa_circ_0004099, hsa_circ_0008898) with fold change > 2 and p < 0.05, we used circPrimer to design specific and accurate detection primers for circular RNAs across the circular junction sites [16]. We then investigated the specificity of the primer set for circRNAs by serially diluting and assessing synthetic circRNA of the six upregulated circRNAs using qRT-PCR assay to generate a standard curve. We consistently and efficiently amplified the six circRNAs with the specific primers. Moving forward, we analyzed these six circRNAs in the training stage involving 10 IPF patients and 10 control volunteers using qRT-PCR assays. Three of the six circRNAs, namely hsa_circ_0044226, hsa_circ_0004099, and hsa_circ_0008898, were found to be significantly upregulated in the exosomes with fold change > 2 and p < 0.01 (Fig. 2C). Therefore, we selected these three circRNAs for further validation in the validation cohort.

**Fig. 3:**
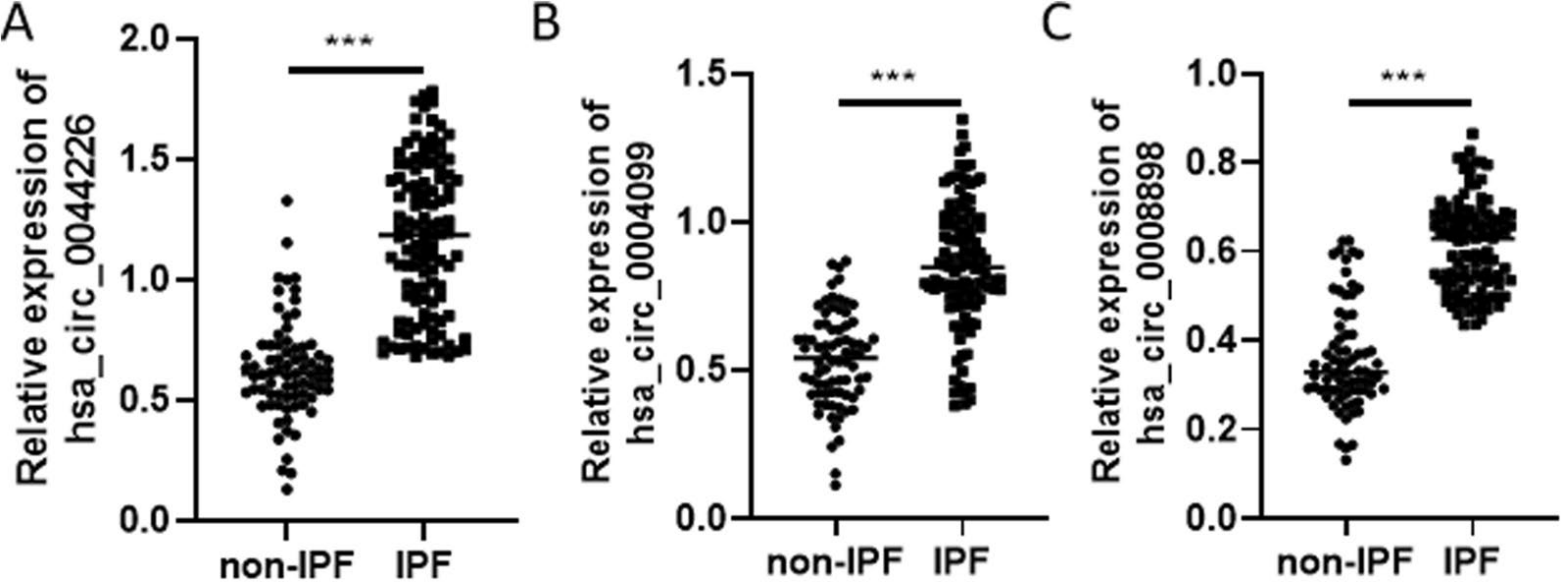
The expression level of exosomal hsa_circ_0044226 (**A**), hsa_circ_0004099 (**B**), hsa_circ_0008898 (**C**) in IPF patients and control volunteers by RT-qPCR. *IPF* IPF patients, *non-IPF* control volunteers. Each test was repeated three times. The mean differences between diagnostic groups were analyzed by Student’s t test. ***P < 0.001

**Fig. 4:**
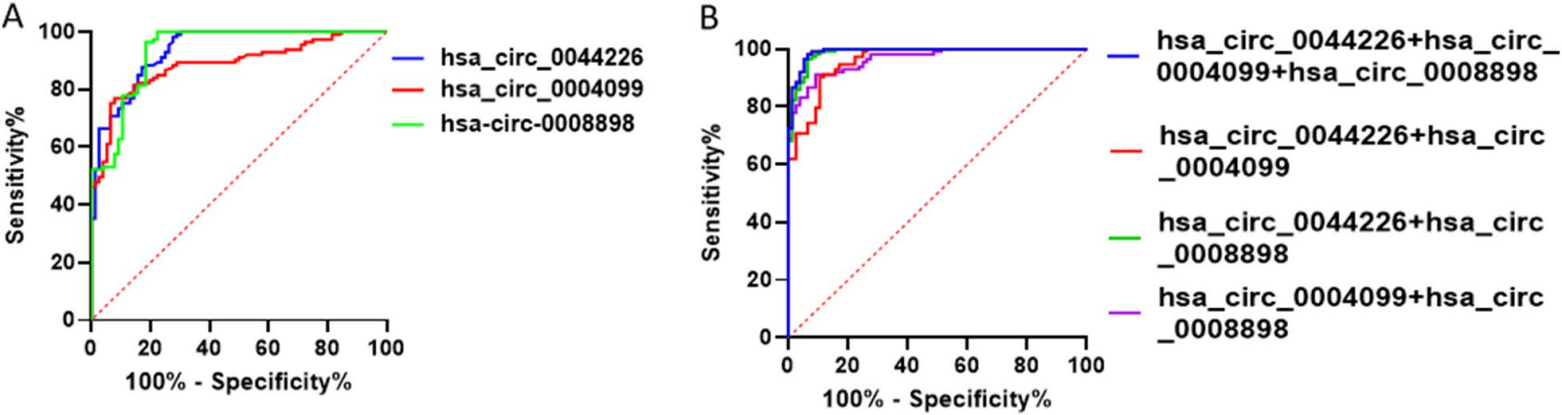
ROC curve analysis for exosomal circRNAs derived from the plasma. **A** The ROC curve analysis of hsa_circ_0044226, hsa_circ_0004099 and hsa_circ_0008898. **B** The ROC curve analysis of the combination of hsa_circ_0044226 + hsa_circ_0004099, hsa_circ_0044226 + hsa_circ_0008898, hsa_circ_0004099 + hsa_circ_0008898 and hsa_circ_0044226 + hsa_circ_0004099 + hsa_circ_0008898

In the validation cohort, we collected samples from 113 IPF patients and 76 control volunteers, including the samples used for the high-throughput circRNA microarray assay and training assay, at the Drum Tower Hospital of Nanjing University (Table 1). Consistent with the results from the training stage, exosomal hsa_circ_0044226, hsa_circ_0004099, and hsa_circ_0008898 were remarkably increased in the plasma of IPF patients compared to control volunteers (Fig. 3A–C). We performed ROC curve analysis to investigate the diagnostic value of these three exosomal circRNAs for IPF. The AUCs of hsa_circ_0044226, hsa_circ_0004099, and hsa_circ_0008898 were 0.936, 0.887, and 0.934, respectively (Fig. 4A and Table 3). Furthermore, we evaluated the performance of the combination of these three circRNAs in distinguishing IPF patients from non-IPF controls through logistic regression and ROC analysis. According to the results of the logistic regression, the risk score (Logit(P)) for the diagnosis based on the three exosomal circRNAs is shown in Fig. 4B and Table 3. The combination of the three circRNAs (hsa_circ_0044226 + hsa_circ_0004099 + hsa_circ_0008898) had the highest AUC value of 0.991, with a discriminative sensitivity and specificity of 98.23 and 93.42, respectively.

**Table 3:** Receiver operating characteristic (ROC) analysis

circRNA	AUC	95% CI	Sensitivity	Specificity
hsa_circ_0044226	0.936	0.903–0.969	98.23	72.37
hsa_circ_0004099	0.887	0.84–0.934	76.99	92.11
hsa_circ_0008898	0.934	0.899–0.97	96.46	81.58
hsa_circ_0044226+hsa_circ_0004099	0.965	0.944–0.986	91.15	90.79
hsa_circ_0044226+hsa_circ_0008898	0.987	0.976–0.999	96.46	93.42
hsa_circ_0004099+hsa_circ_0008898	0.96	0.935–0.984	90.27	89.47
hsa_circ_0044226+hsa_circ_0004099+hsa_circ_0008898	0.991	0.982–1	98.23	93.42

### Hsa_circ_0044226 is associated with lung function of IPF patients

**Fig. 5:**
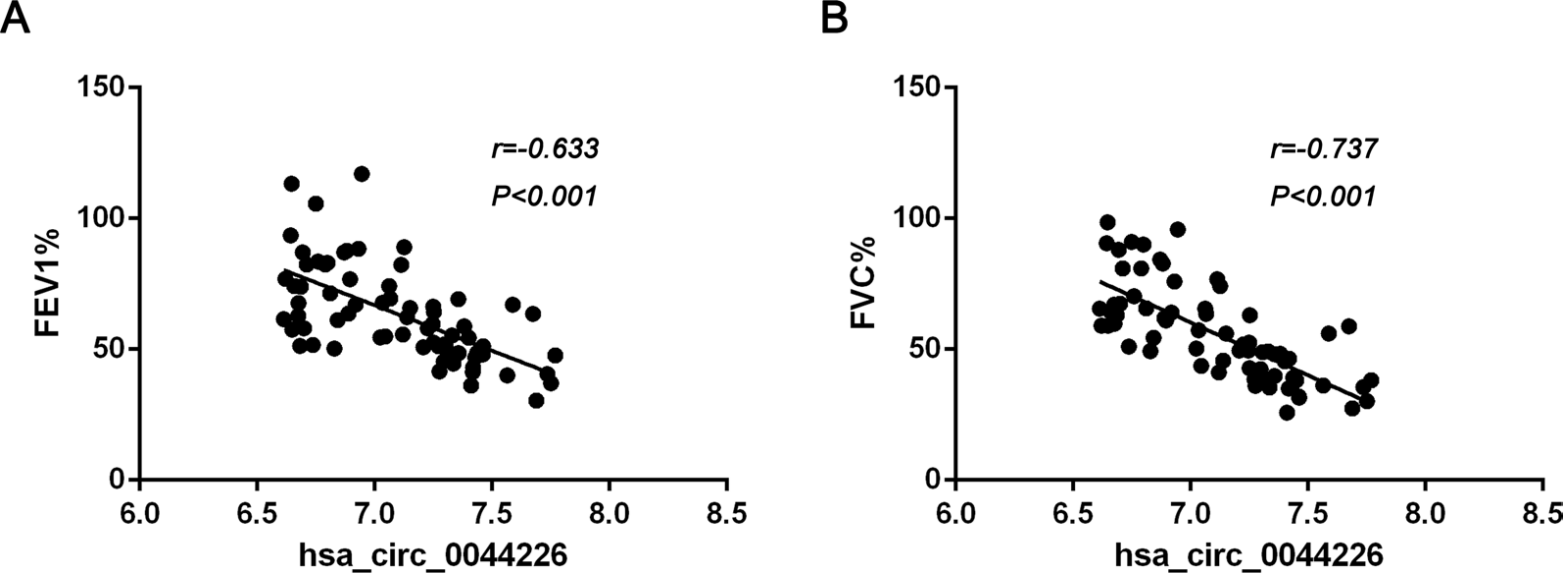
The association of lung function with exosomal circRNAs in plasma of IPF patients. **A** The association of FEV1 with hsa_circ_0044226. **B** The association of FVC with hsa_circ_0044226

Through the analysis of the relationship between lung function and hsa_circ_0044226, hsa_circ_0004099, and hsa_circ_0008898 in IPF patients, it was observed that the expression level of exosomal hsa_circ_0044226 exhibited an inverse correlation with Forced Expiratory Volume (FEV1) (r = −0.633, P < 0.001) and Forced Vital Capacity (FVC) (r = −0.737, P < 0.001) in IPF patients (Fig. 5A, B). The other two circRNAs (hsa_circ_0004099 and hsa_circ_0008898) don't have a clear correlation with FEV1 and FVC (data not shown).

### The diagnostic value of hsa_circ_0044226 in progression prediction of IPF

**Fig. 6:**
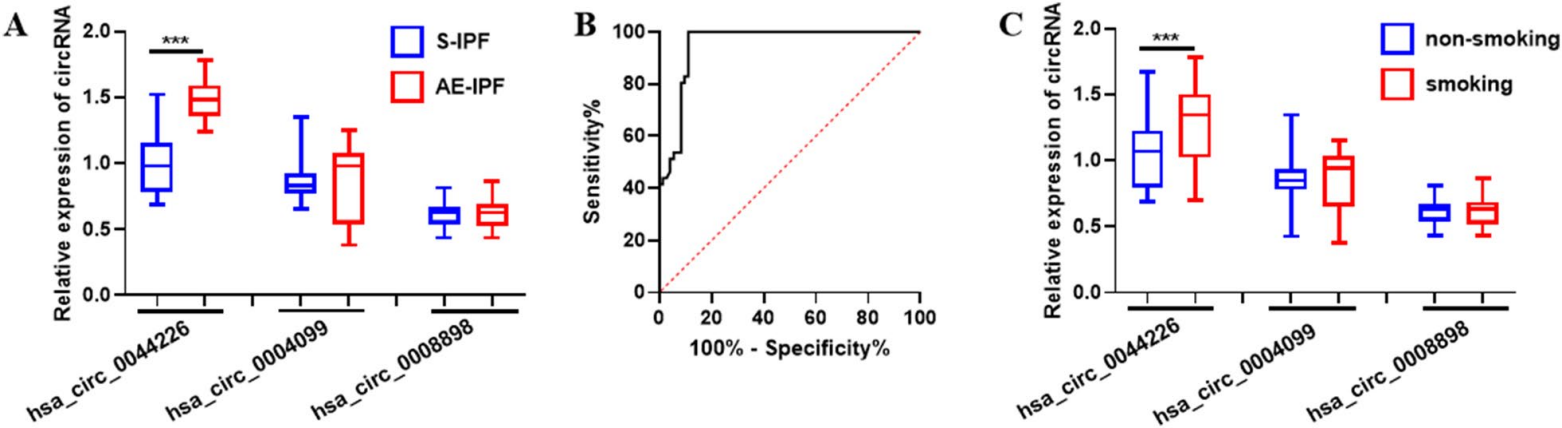
The expression of exosomal cirRNAs in the plasma of S-IPF patients and AE-IPF patients. **A** The level of exosomal cirRNAs in the plasma of stable IPF patients (S-IPF) and acute exacerbation IPF patients (AE-IPF).** B** Receiver operating characteristic curve of hsa_circ_0044226. **C** The level of exosomal cirRNAs in the plasma of IPF patients with smoking (smoking) and non-smoking (non-smoking). Each test was repeated three times. The mean differences between diagnostic groups were analyzed by Student’s t test. ***P < 0.001

Acute exacerbations of IPF (AE-IPF), characterized by the onset of widespread acute lung injury, significantly contribute to the progression and mortality associated with IPF [17, 18]. This can even occur in individuals with limited fibrosis and well-preserved lung function [17, 18]. Timely diagnosis is critical in reducing mortality rates related to acute exacerbations of IPF. Currently, there are no validated non-invasive biomarkers for diagnosing or predicting AE-IPF. The expression levels of exosomal hsa_circ_0044226, hsa_circ_0004099, and hsa_circ_0008898 were assessed in 71 Acute exacerbations of IPF (AE-IPF) patients and 42 stable IPF (S-IPF) patients. The results revealed that exosomal hsa_circ_0044226 was significantly higher in the plasma samples of AE-IPF patients compared to S-IPF patients (Fig. 6A). Moreover, the areas under the curve (AUCs) of hsa_circ_0044226 in distinguishing between AE-IPF and S-IPF were 0.9517 (Fig. 6B), with a discriminative sensitivity of 100 and specificity of 88.89.

Cigarette smoking is one of the most recognized risk factors for development of IPF [2]. The relationship between cigarette smoking and hsa_circ_0044226, hsa_circ_0004099, and hsa_circ_0008898 in IPF patients was also investigated. As the result showed in Fig. 6C, the expression level of exosomal hsa_circ_0044226 was significantly increased in IPF patients with cigarette smoking, while the hsa_circ_0004099 and hsa_circ_0008898 had no difference.

### Exosomal mmu_circ_0002687 upregulated in the plasma of bleomycin-induced IPF mice model

**Fig. 7:**
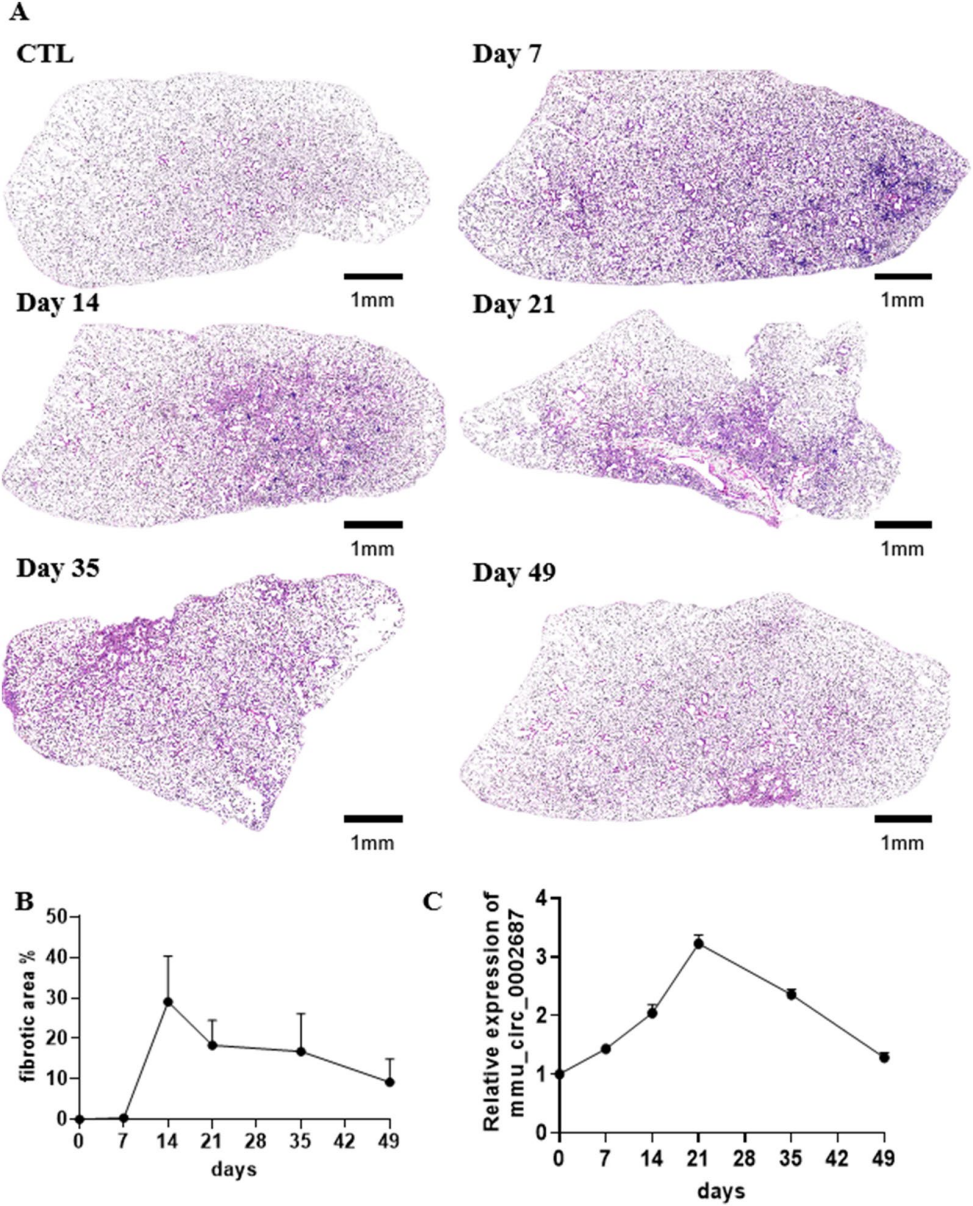
The pathology and expression level of plasma exosomal mmu_circ_0002687 of single does bleomycin-induced IPF mouse model. **A**–**B** The pathology of single does bleomycin-induced IPF mouse model. observation time points of bleomycin-induced mouse model at 7, 14, 21,35 and 49 days, CTL/0: mice without bleomycin treatment. **A**: Represent image (4x); **B**: statistic value of fibrotic area. **C** The expression level of plasma exosomal mmu_circ_0002687at 0, 7, 14, 21,35 and 49 days. 0 days means mice without bleomycin treatment (CTL). Five mice were at each time point. Each test was repeated three times. The mean differences were analyzed by Student’s t test. *P < 0.5;**P < 0.01;***P < 0.001

Through sequence alignment, hsa_circ_0044226 was observed to be conserved in both humans and mice, where it is referred to as mmu_circ_0002687 in mice (Additional file 1: Figure S1). Consequently, the expression level of exosomal hsa_circ_0044226 in plasma was investigated in an IPF mice model induced by a single dose of bleomycin. Consistent with previous reports [17, 18], the IPF induced by a single dose of bleomycin initially progressed rapidly and then entered a stable phase after approximately 21 days due to the disappearance of bleomycin-induced effects (Fig. 7A, B). In comparison to control mice without bleomycin treatment (CTL), noticeable inflammatory cell infiltration in the lungs and local fibrotic features such as collagen deposition were evident at day 7 (Fig. 7A). The lung inflammation gradually subsided over time after modeling and was replaced by the gradual accumulation of collagen deposition, leading to the collapse of alveolar structure and the loss of local lung tissue respiratory function (Fig. 7A, B). After 21 days, the IPF mice model entered a stable phase (Fig. 7A, B). Plasma samples were collected at 7, 14, 21, 35, and 49 days, and the expression level of exosomal mmu_circ_0002687 in the plasma was determined using RT-qPCR. The results demonstrated that exosomal mmu_circ_0002687 in the plasma rapidly increased initially and then began to decrease at 21 days, consistent with the trend of pathological changes (Fig. 7C). These findings reaffirm that blood exosomal hsa_circ_0044226 not only holds potential as a biomarker for IPF diagnosis but also indicates the progression of IPF.

### The biological function of hsa_circ_0044226

**Fig. 8:**
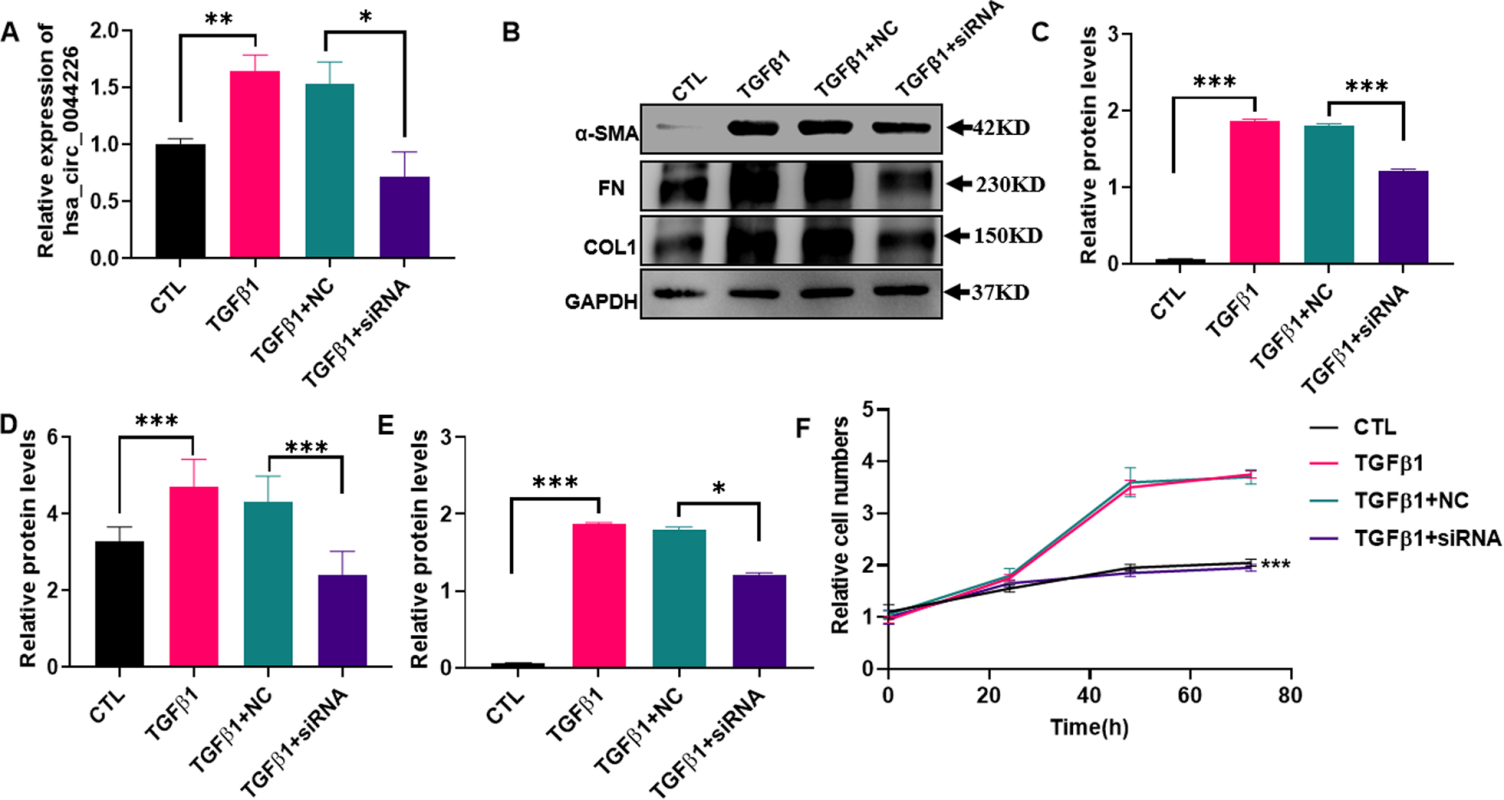
The biological function of hsa_circ_0044226. **A** The expression level of hsa_circ_0044226 in HFL1 cells treated with TGFβ1, TGFβ1 plus scramble RNA (TGFβ1 + NC) or TGFβ1 plus hsa_circ_0044226 siRNA (TGFβ1 + siRNA). **B**–**E** The protein levels of α-SMA, FN and COL1 in HFL1 cells treated with TGFβ1, TGFβ1 plus scramble RNA (TGFβ1 + NC) or TGFβ1 plus hsa_circ_0044226 siRNA (TGFβ1 + siRNA). **B** Represent image; **C**–**E** statistic value of α-SMA, FN and COL1. **F** the proliferation ability of HFL1 cells treated with TGFβ1, TGFβ1 plus scramble RNA (TGFβ1 + NC) or TGFβ1 plus hsa_circ_0044226 siRNA (TGFβ1 + siRNA). CTL: HFL1 cells treated without TGFβ1. Each test was repeated three times. The mean differences were analyzed by Student’s t test. *P < 0.5;**P < 0.01;***P < 0.001

Due to the inverse correlation between the expression level of blood exosomal hsa_circ_0044226 and lung function (Fig. 5), as well as its upregulation in AE-IPF, the function of hsa_circ_0044226 was investigated through in vitro experiments. The human fetal lung fibroblast cell line HFL1 was subjected to TGFβ1 treatment to induce myofibroblasts. Following TGFβ1 treatment, hsa_circ_0044226 exhibited a significant increase, which could be attenuated by hsa_circ_0044226 siRNAs (Fig. 8A). TGFβ1, a crucial cytokine in IPF, was found to induce the expression of collagen (COL1), smooth muscle actin (α-SMA), and fibronectin (FN) in myofibroblasts, leading to their secretion and deposition in the extracellular matrix (Fig. 8B–E). However, the protein levels of α-SMA, FN, and COL1 were downregulated by hsa_circ_0044226 siRNAs in the myofibroblasts (Fig. 8B–E). Furthermore, the impact of hsa_circ_0044226 on myofibroblast proliferation was assessed using a CCK-8 assay. As per previous reports, TGFβ1 was observed to promote myofibroblast proliferation (Fig. 8F), whereas the promotion by TGFβ1 was nullified by hsa_circ_0044226 siRNAs.

These findings suggest that hsa_circ_0044226 plays a critical role in the progression of IPF through the TGFβ1 signaling pathway and could potentially serve as a new therapeutic target for IPF treatment. However, further studies are required to validate these findings.

## Discussion

In this study, we conducted circRNA array screening and independent validation, which revealed significantly higher expression levels of exosomal hsa_circ_0044226, hsa_circ_0004099, and hsa_circ_0008898 in the plasma of IPF patients compared to non-IPF participants. The combination of these exosomal circRNAs in the plasma could potentially serve as ideal biomarkers for IPF diagnosis.

Notably, hsa_circ_0044226 in plasma exosomes exhibited an inverse correlation with lung function and was significantly upregulated in AE-IPF patients compared to S-IPF patients. This suggests that hsa_circ_0044226 may have the potential to serve as an indicator for predicting IPF progression. Furthermore, in vitro studies indicated that hsa_circ_0044226 may contribute to the progression of IPF through the TGFβ1 signaling pathway.

Due to their stable loop structure and intricate gene regulatory functions, exosomal circRNAs have emerged as promising biomarkers for diagnosing and monitoring various diseases, particularly cancers [4]. However, prior to our study, only one paper by Cheng et al. had reported the application of exosomal circRNAs in diagnosing IPF [19]. In their study, Cheng et al. found that hsa_circ_58493 levels in the serum of IPF patients were significantly higher, with an AUC of 0.823 for IPF screening. However, their study had a small sample size, comprising only 20 IPF cases and 24 healthy controls. In contrast, our study included 113 IPF patients and 76 control volunteers. Notably, we also evaluated the expression of hsa_circ_58493 in the exosomes of plasma and found no significant difference between IPF patients and control volunteers (data not shown). As a result, we were the first group to identify the diagnostic value of exosomal circRNAs in IPF.

Several studies have demonstrated the critical role of circRNAs in the development and progression of IPF [7–9, 12, 13]. For example, Zhang et al. found that circHIPK3 could promote lung fibroblast-to-myofibroblast transition by acting as a competing endogenous RNA of miR-338-3p, leading to increased SOX4 and COL1A1 expression [20]. Li et al. confirmed that circTADA2A acted as a sponge for miR-526b and miR-203, releasing the expression of their target genes (such as Caveolin (Cav)-1 and Cav2), which in turn suppressed lung fibroblast activation via Cav1 and reduced lung fibroblast proliferation via Cav2 [9]. Xu et al. reported that circANKRD42 could sponge miR-324-5p and miR-136-5p to promote YAP1 translation into the nucleus during IPF [13]. Additionally, Li et al. revealed that circSPON1 bound to miR-942-5p and miR-520f-3p, interfering with Smad7 mRNA and promoting Smad7 expression in the development of pulmonary fibrosis [7]. In our study, we found that hsa_circ_0044226 was positively associated with the lung function of IPF patients and upregulated in AE-IPF patients. Furthermore, the exosomal hsa_circ_0044226 in the plasma of a bleomycin-induced IPF mice model increased as lung fibrosis progressed. Therefore, hsa_circ_0044226 is not only considered a biomarker for predicting IPF progression but is also speculated to play a central role in the progression of IPF. Through in vitro experiments, we found that hsa_circ_0044226 is involved in the TGFβ1 signaling pathway. It has been demonstrated to upregulate the expression of CDC27 in a BLM-induced pulmonary fibrosis mouse model [12]. However, the underlying mechanism requires further study.

This study has several limitations. Firstly, plasma exosomes may not fully represent tissue expression, and additional research is necessary to investigate the expression profiles of relevant circRNAs in tissues. Secondly, the generalizability of these results to other ethnic groups is uncertain. Lastly, further studies are needed to fully elucidate the mechanism linking hsa_circ_0044226 from plasma exosomes to IPF.

In conclusion, our study suggests that the high expression of hsa_circ_0044226, hsa_circ_0004099, and hsa_circ_0008898 in plasma exosomes could serve as novel biomarkers for diagnosing and monitoring IPF. Furthermore, the clarified biological function of hsa_circ_0044226 indicates its potential as a promising novel therapy for IPF.

### Supplementary Information

**Additional file 1: Table S1.** The primer sequences of circRNAs used for the RT-qPCR. **Table S2.** The expression profile of exosomal circRNA in the plasma of IPF patients and control volunteers. **Figure S1.** Sequence alignmen of hsa_circ_0044226 and mmu_circ_0002687.

## Data Availability

The data and materials generated in this study are available upon request from the corresponding authors.

## Abbreviations

IPF: Idiopathic pulmonary fibrosis
HFL1: Human fetal lung fibroblast
ROC: Receiver operating characteristic
FEV1: Expiratory volume in 1 s
FVC: Forced vital capacity
AE-IPF: Acute exacerbations of IPF
HRCT: High-resolution computed tomography
COPD: Chronic obstructive pulmonary disease
RT-qPCR: Quantitative reverse transcriptase-polymerase chain reaction
BMI: Body mass index
SBP: Systolic blood pressure
DBP: Diastolic blood pressure
α-SMA: Smooth muscle actin
FN: Fibronectin
COL1: Collagen1
TEM: Transmission electron microscopy (TEM)
NTA: Nanoparticle tracking analysis
AUC: The area under the ROC curve

## Declarations

### Declaration of AI-assisted technologies in the writing process

During the preparation of this work the authors used chatGPT in order to improve language and readability. After using this tool, the authors reviewed and edited the content as needed and take full responsibility for the content of the publication.
