# Supplementary material for: Exosomal circRNAs in the plasma serve as novel biomarkers for IPF diagnosis and progression prediction
Source: J Transl Med. 2024 Mar 10;22:264. doi: 10.1186/s12967-024-05034-9 (PMC10926640; doi:10.1186/s12967-024-05034-9)
Supplement: Supplementary file 1 — Additional file 1: Table S1. The primer sequences of circRNAs used for the RT-qPCR. Table S2. The expression profile of exosomal circRNA in the plasma of IPF patients and control volunteers. Figure S1. Sequence alignmen of hsa_circ_0044226 and mmu_circ_0002687. [file 12967_2024_5034_MOESM1_ESM.docx]

**Table S1. The primer sequences of circRNAs used for the RT-qPCR.**

| **circRNA** | **Alias** | **F** | **R** | **PRODUCT SIZE** |
| --- | --- | --- | --- | --- |
| hsa_circRNA_102100 | hsa_circ_0044226 | TTTCAGTCTGCAACGGGAAC | CCTGAGGTGTTGTACATGCA | 200 |
| hsa_circRNA_100759 | hsa_circ_0004099 | CTCACAGCCATTATGCCAGT | GTCTTGAATGCCAGCCCTTT | 222 |
| hsa_circRNA_100705 | hsa_circ_0008898 | TTTTGCAGATCTGTGGTTTT | GGATGGTAGTTGTGTGCACC | 207 |
| hsa_circRNA_101550 | hsa_circ_0035796 | AGTTCAGGCTTCCAGTCCTC | AGAGTCCTGGTTTTCTGATATCTGA | 150 |
| hsa_circRNA_102348 | hsa_circ_0007535 | TGAAGTTCAAAAACTTTTCAG | TCACCTACTCGAACCTGTTCTA | 435 |
| hsa_circRNA_102101 | hsa_circ_0044234 | ACATGTATATTGTACACT | GTAACAATATCATCATGGCTT | 233 |
| mmu_circ_0002687 |  | GTGTGCTGCAGGGAATTGTT | CCTGAGGTGTTGTACATGCA | 218 |

**Table s2. the expression profile of exosomal circRNA in the plasma of IPF patients and control volunteers.**

| circRNA ID | control#1 | control#2 | control#3 | IPF#1 | IPF#2 | IPF#3 | Mean(control) | Mean(IPF) | FC | P | regulation |
| --- | --- | --- | --- | --- | --- | --- | --- | --- | --- | --- | --- |
| hsa_circ_102100 | 2547 | 2114 | 2969 | 4866 | 7813 | 7775 | 2543.310504 | 6817.783853 | 2.680673022 | 0.013211091 | up |
| hsa_circ_100759 | 1566 | 1308 | 1138 | 3829 | 3025 | 2897 | 1337.284151 | 3250.296508 | 2.430520474 | 0.003811672 | up |
| hsa_circ_100705 | 187 | 182 | 235 | 405 | 543 | 507 | 201.1356813 | 485.1066108 | 2.41183766 | 0.00316654 | up |
| hsa_circ_101550 | 227 | 388 | 507 | 592 | 976 | 948 | 373.9573968 | 838.6391151 | 2.242606036 | 0.034767458 | up |
| hsa_circ_102348 | 745 | 446 | 702 | 1414 | 1245 | 1288 | 630.7213072 | 1315.736579 | 2.086082337 | 0.002980247 | up |
| hsa_circ_102101 | 1835 | 2943 | 3295 | 4066 | 6337 | 6481 | 2691.248609 | 5627.997242 | 2.091221607 | 0.030685935 | up |
| hsa_circ_102470 | 3905 | 2193 | 2853 | 1809 | 1127 | 1125 | 2983.410345 | 1353.624268 | 0.453717093 | 0.041000819 | Down |
| hsa_circ_101192 | 231 | 452 | 376 | 168 | 131 | 161 | 352.8276554 | 153.1956654 | 0.434194041 | 0.038511187 | Down |
| hsa_circ_104780 | 1481 | 1001 | 1252 | 624 | 452 | 463 | 1244.422625 | 512.9482554 | 0.41219779 | 0.008057132 | Down |
| hsa_circ_101225 | 1940 | 1437 | 1884 | 701 | 675 | 670 | 1753.261979 | 681.96289 | 0.388968048 | 0.00255725 | Down |
| hsa_circ_102049 | 19596 | 9304 | 7161 | 3266 | 2377 | 2340 | 12020.4152 | 2661.148739 | 0.221385759 | 0.071902088 | Down |
| hsa_circ_100906 | 819 | 697 | 703 | 1377 | 1368 | 1383 | 739.7302669 | 1376.158879 | 1.860352267 | 8.85595E-05 | unchanged |
| hsa_circ_103712 | 554 | 319 | 543 | 889 | 854 | 806 | 472.0024081 | 849.5502787 | 1.799885476 | 0.009297144 | unchanged |
| hsa_circ_104532 | 594 | 573 | 693 | 758 | 1455 | 1355 | 620.1732297 | 1189.228183 | 1.917574197 | 0.061486942 | unchanged |
| hsa_circ_104211 | 220 | 191 | 280 | 312 | 490 | 467 | 230.2554026 | 422.8280322 | 1.836343588 | 0.035898828 | unchanged |
| hsa_circ_100657 | 228 | 245 | 286 | 578 | 399 | 417 | 253.0757191 | 464.6689912 | 1.836086816 | 0.023696139 | unchanged |
| hsa_circ_104208 | 190 | 155 | 172 | 231 | 337 | 313 | 172.2996963 | 293.9783358 | 1.706203448 | 0.022448687 | unchanged |
| hsa_circ_103690 | 102 | 139 | 181 | 195 | 246 | 254 | 140.5207576 | 231.6579688 | 1.648567605 | 0.037023065 | unchanged |
| hsa_circ_104980 | 118 | 130 | 167 | 169 | 281 | 256 | 138.4108838 | 235.4282302 | 1.700937265 | 0.057724422 | unchanged |
| hsa_circ_400037 | 279 | 336 | 333 | 432 | 603 | 568 | 316.1093357 | 534.2249496 | 1.690000545 | 0.017063919 | unchanged |
| hsa_circ_101487 | 64 | 63 | 81 | 113 | 114 | 119 | 69.15238438 | 115.3482662 | 1.668030209 | 0.001540147 | unchanged |
| hsa_circ_102367 | 120 | 77 | 84 | 148 | 158 | 155 | 93.75823218 | 153.7356007 | 1.639702426 | 0.011308439 | unchanged |
| hsa_circ_101272 | 150 | 206 | 142 | 278 | 268 | 263 | 165.9710602 | 269.7324135 | 1.625177384 | 0.007278517 | unchanged |
| hsa_circ_100832 | 922 | 709 | 1029 | 1312 | 1504 | 1508 | 886.7827165 | 1441.463773 | 1.625498272 | 0.00826069 | unchanged |
| hsa_circ_101691 | 82 | 68 | 85 | 123 | 112 | 142 | 78.26700646 | 125.635402 | 1.605215374 | 0.010431425 | unchanged |
| hsa_circ_104840 | 462 | 471 | 518 | 627 | 852 | 863 | 483.6977009 | 780.4365987 | 1.613480067 | 0.019707658 | unchanged |
| hsa_circ_001506 | 6694 | 5629 | 5754 | 12521 | 8364 | 8385 | 6025.551255 | 9756.607191 | 1.619205742 | 0.058630408 | unchanged |
| hsa_circ_100678 | 133 | 134 | 129 | 230 | 203 | 199 | 132.0202543 | 210.7284062 | 1.596182399 | 0.001361761 | unchanged |
| hsa_circ_102913 | 237 | 243 | 237 | 345 | 414 | 383 | 238.7588643 | 380.927765 | 1.595449727 | 0.002062063 | unchanged |
| hsa_circ_001651 | 83 | 68 | 93 | 99 | 131 | 158 | 81.30377019 | 129.5108885 | 1.592925989 | 0.059030683 | unchanged |
| hsa_circ_103626 | 123 | 74 | 89 | 136 | 148 | 156 | 95.39206561 | 146.6813228 | 1.537667958 | 0.029031971 | unchanged |
| hsa_circ_102380 | 756 | 726 | 775 | 1107 | 1174 | 1256 | 752.3168601 | 1178.97213 | 1.567121771 | 0.000709731 | unchanged |
| hsa_circ_102771 | 396 | 383 | 502 | 505 | 764 | 750 | 427.0074704 | 673.2387989 | 1.576644077 | 0.056002418 | unchanged |
| hsa_circ_101996 | 171 | 189 | 260 | 268 | 352 | 329 | 206.4894121 | 316.1223255 | 1.530937215 | 0.041638394 | unchanged |
| hsa_circ_104460 | 745 | 798 | 553 | 1013 | 953 | 1256 | 698.7565854 | 1074.089292 | 1.537143713 | 0.034188055 | unchanged |
| hsa_circ_102038 | 371 | 234 | 292 | 430 | 483 | 449 | 299.230704 | 453.7560841 | 1.516408838 | 0.022284146 | unchanged |
| hsa_circ_104576 | 88 | 63 | 55 | 92 | 106 | 114 | 68.59931292 | 103.9751571 | 1.515688024 | 0.039164426 | unchanged |
| hsa_circ_102241 | 1110 | 1121 | 1271 | 1965 | 1767 | 1656 | 1167.547639 | 1795.953253 | 1.538226958 | 0.003793044 | unchanged |
| hsa_circ_101486 | 575 | 599 | 674 | 717 | 1086 | 1063 | 615.8283991 | 955.4810877 | 1.551537878 | 0.050819548 | unchanged |
| hsa_circ_103206 | 99 | 73 | 104 | 135 | 139 | 141 | 91.77028035 | 138.2845307 | 1.506855271 | 0.009008488 | unchanged |
| hsa_circ_100624 | 234 | 216 | 264 | 300 | 369 | 422 | 238.1184202 | 363.6008164 | 1.526974755 | 0.029837371 | unchanged |
| hsa_circ_000095 | 244 | 197 | 249 | 285 | 389 | 377 | 230.1045807 | 350.4030622 | 1.52279916 | 0.031326984 | unchanged |
| hsa_circ_104230 | 159 | 114 | 169 | 231 | 212 | 211 | 147.2520523 | 218.0549146 | 1.480827678 | 0.017536924 | unchanged |
| hsa_circ_101701 | 74 | 82 | 93 | 108 | 136 | 129 | 82.98341941 | 124.0815111 | 1.495256667 | 0.014216398 | unchanged |
| hsa_circ_101334 | 265 | 337 | 356 | 387 | 512 | 533 | 319.2610399 | 477.4424243 | 1.49546097 | 0.041169048 | unchanged |
| hsa_circ_100300 | 339 | 274 | 274 | 402 | 468 | 446 | 295.7344092 | 438.3161878 | 1.482127795 | 0.008106018 | unchanged |
| hsa_circ_102988 | 362 | 396 | 395 | 460 | 594 | 648 | 384.0633941 | 567.1952272 | 1.476827096 | 0.032465617 | unchanged |
| hsa_circ_101408 | 1790 | 1894 | 1655 | 2707 | 2581 | 2495 | 1779.672078 | 2594.580597 | 1.457898131 | 0.000913137 | unchanged |
| hsa_circ_103636 | 269 | 255 | 351 | 364 | 476 | 431 | 292.0895902 | 423.562865 | 1.450112839 | 0.040844288 | unchanged |
| hsa_circ_105031 | 504 | 452 | 469 | 634 | 743 | 700 | 475.2413311 | 692.4227038 | 1.456991761 | 0.003416304 | unchanged |
| hsa_circ_102652 | 634 | 589 | 599 | 772 | 905 | 976 | 607.2122659 | 884.589416 | 1.45680426 | 0.010681655 | unchanged |
| hsa_circ_100363 | 158 | 144 | 191 | 192 | 258 | 266 | 164.1464865 | 238.701738 | 1.454199496 | 0.051459158 | unchanged |
| hsa_circ_101914 | 239 | 229 | 272 | 354 | 372 | 343 | 246.9490124 | 356.4546881 | 1.443434354 | 0.002090418 | unchanged |
| hsa_circ_101257 | 354 | 301 | 349 | 393 | 542 | 520 | 334.6977933 | 484.9677671 | 1.448972108 | 0.037722581 | unchanged |
| hsa_circ_100976 | 156 | 125 | 170 | 203 | 217 | 222 | 150.0728383 | 214.2543913 | 1.427669349 | 0.011485816 | unchanged |
| hsa_circ_100290 | 258 | 198 | 216 | 307 | 308 | 346 | 223.6486686 | 320.1320109 | 1.431405842 | 0.011907782 | unchanged |
| hsa_circ_103328 | 467 | 582 | 472 | 613 | 727 | 852 | 507.1474343 | 730.5686728 | 1.440544945 | 0.046703373 | unchanged |
| hsa_circ_101810 | 404 | 312 | 327 | 451 | 511 | 529 | 347.7501848 | 496.8500817 | 1.428755766 | 0.015668388 | unchanged |
| hsa_circ_104416 | 140 | 165 | 139 | 181 | 224 | 233 | 148.0016251 | 212.9297995 | 1.438699064 | 0.022747501 | unchanged |
| hsa_circ_101346 | 329 | 350 | 319 | 423 | 503 | 508 | 332.6305955 | 477.965137 | 1.436924755 | 0.007406652 | unchanged |
| hsa_circ_103812 | 413 | 445 | 401 | 568 | 621 | 613 | 419.8135168 | 600.8651587 | 1.431266824 | 0.00103229 | unchanged |
| hsa_circ_101969 | 2601 | 2032 | 2632 | 3596 | 3491 | 3239 | 2421.519267 | 3441.770126 | 1.421326757 | 0.010054121 | unchanged |
| hsa_circ_101369 | 556 | 570 | 561 | 666 | 887 | 878 | 562.3987486 | 810.4033295 | 1.44097641 | 0.026501648 | unchanged |
| hsa_circ_102357 | 195 | 207 | 211 | 246 | 320 | 316 | 204.5847991 | 294.0155937 | 1.437133135 | 0.021252239 | unchanged |
| hsa_circ_102940 | 188 | 222 | 232 | 285 | 312 | 314 | 214.1725934 | 303.6124549 | 1.417606474 | 0.005356313 | unchanged |
| hsa_circ_104771 | 662 | 791 | 707 | 883 | 1103 | 1081 | 720.186558 | 1022.117073 | 1.419239309 | 0.019154285 | unchanged |
| hsa_circ_103276 | 416 | 284 | 378 | 509 | 533 | 464 | 359.3721453 | 501.6369864 | 1.395870529 | 0.03241147 | unchanged |
| hsa_circ_104309 | 299 | 303 | 265 | 368 | 361 | 507 | 288.9067443 | 412.1700865 | 1.42665443 | 0.065820839 | unchanged |
| hsa_circ_103637 | 492 | 433 | 556 | 700 | 709 | 672 | 493.8324642 | 693.3831896 | 1.404085879 | 0.005859867 | unchanged |
| hsa_circ_100630 | 114 | 112 | 128 | 137 | 185 | 181 | 118.2679087 | 167.5603481 | 1.41678626 | 0.037114393 | unchanged |
| hsa_circ_101879 | 1403 | 1490 | 1509 | 2083 | 2209 | 1880 | 1467.239038 | 2057.1834 | 1.40207788 | 0.004320401 | unchanged |
| hsa_circ_103226 | 778 | 631 | 583 | 831 | 974 | 968 | 664.0309599 | 924.6918097 | 1.392543218 | 0.02530236 | unchanged |
| hsa_circ_101606 | 87 | 81 | 108 | 109 | 132 | 144 | 91.94034579 | 128.2213573 | 1.394614695 | 0.050134063 | unchanged |
| hsa_circ_104095 | 296 | 239 | 257 | 335 | 378 | 385 | 263.8953237 | 365.9956722 | 1.386897149 | 0.0117449 | unchanged |
| hsa_circ_104108 | 852 | 824 | 720 | 1076 | 1164 | 1077 | 798.4226326 | 1105.533736 | 1.384647292 | 0.003476861 | unchanged |
| hsa_circ_100202 | 5982 | 4611 | 5797 | 7961 | 7342 | 7295 | 5463.706792 | 7532.766823 | 1.37869163 | 0.012551999 | unchanged |
| hsa_circ_101738 | 290 | 243 | 258 | 298 | 405 | 399 | 263.5650889 | 367.2583732 | 1.393425718 | 0.049779942 | unchanged |
| hsa_circ_102349 | 102 | 74 | 97 | 115 | 127 | 132 | 90.87278556 | 124.6943468 | 1.37218581 | 0.02783237 | unchanged |
| hsa_circ_102862 | 164 | 127 | 169 | 190 | 223 | 219 | 153.404782 | 210.6696575 | 1.373292637 | 0.027692662 | unchanged |
| hsa_circ_104207 | 179 | 181 | 227 | 239 | 290 | 280 | 195.9043837 | 269.7494091 | 1.376944221 | 0.028949871 | unchanged |
| hsa_circ_104528 | 62 | 45 | 57 | 78 | 77 | 69 | 54.58658636 | 74.76920935 | 1.369735943 | 0.027493861 | unchanged |
| hsa_circ_103869 | 68 | 52 | 69 | 76 | 92 | 92 | 63.12931459 | 86.75976196 | 1.374318136 | 0.03407086 | unchanged |
| hsa_circ_104984 | 62 | 64 | 66 | 80 | 97 | 88 | 64.18192656 | 88.71736647 | 1.38227958 | 0.008178954 | unchanged |
| hsa_circ_100440 | 226 | 193 | 245 | 296 | 311 | 302 | 221.5483281 | 303.1876519 | 1.368494425 | 0.006819203 | unchanged |
| hsa_circ_103639 | 500 | 463 | 456 | 656 | 627 | 665 | 473.0511524 | 649.528427 | 1.373061716 | 0.000589034 | unchanged |
| hsa_circ_100757 | 233 | 254 | 214 | 280 | 342 | 338 | 233.2430833 | 320.088905 | 1.372340394 | 0.020549707 | unchanged |
| hsa_circ_101396 | 1334 | 1441 | 1309 | 1734 | 1962 | 1894 | 1361.508861 | 1863.561566 | 1.368747292 | 0.00313145 | unchanged |
| hsa_circ_100512 | 178 | 169 | 203 | 215 | 264 | 273 | 183.6572547 | 250.7551534 | 1.365343034 | 0.030837983 | unchanged |
| hsa_circ_101035 | 269 | 286 | 284 | 337 | 400 | 409 | 279.7652315 | 381.901157 | 1.365077265 | 0.011973592 | unchanged |
| hsa_circ_103317 | 151 | 132 | 154 | 170 | 227 | 202 | 145.7991166 | 199.2228635 | 1.366420237 | 0.040028968 | unchanged |
| hsa_circ_101314 | 132 | 99 | 136 | 165 | 164 | 163 | 122.0774064 | 163.9213045 | 1.342765295 | 0.022574783 | unchanged |
| hsa_circ_102923 | 113 | 111 | 106 | 138 | 148 | 162 | 110.0317964 | 149.273729 | 1.356641752 | 0.005792436 | unchanged |
| hsa_circ_102233 | 334 | 292 | 275 | 408 | 398 | 409 | 300.3670251 | 405.1208513 | 1.348752751 | 0.004375678 | unchanged |
| hsa_circ_103422 | 367 | 299 | 371 | 426 | 492 | 480 | 345.5197005 | 465.9469511 | 1.348539462 | 0.017825396 | unchanged |
| hsa_circ_001552 | 80 | 66 | 81 | 91 | 108 | 107 | 75.59922022 | 101.8133134 | 1.346750841 | 0.024433421 | unchanged |
| hsa_circ_100791 | 167 | 169 | 173 | 207 | 248 | 232 | 169.7187737 | 228.9557159 | 1.349029992 | 0.007978735 | unchanged |
| hsa_circ_101773 | 214 | 173 | 218 | 256 | 268 | 285 | 201.6006972 | 269.7780617 | 1.338180202 | 0.015139761 | unchanged |
| hsa_circ_103053 | 247 | 202 | 233 | 285 | 311 | 317 | 227.3679035 | 304.1792907 | 1.33782863 | 0.009697165 | unchanged |
| hsa_circ_104251 | 938 | 1000 | 777 | 1229 | 1197 | 1194 | 905.0198496 | 1206.672628 | 1.333310676 | 0.010963123 | unchanged |
| hsa_circ_103292 | 658 | 577 | 564 | 733 | 878 | 790 | 599.3767256 | 800.2101377 | 1.335070421 | 0.017308009 | unchanged |
| hsa_circ_100212 | 61 | 55 | 74 | 79 | 90 | 83 | 63.42487814 | 84.08022865 | 1.325666381 | 0.03550901 | unchanged |
| hsa_circ_100298 | 206 | 166 | 189 | 254 | 232 | 257 | 186.9598031 | 247.6702401 | 1.324724545 | 0.012419862 | unchanged |
| hsa_circ_000750 | 865 | 795 | 653 | 914 | 1114 | 1034 | 771.2483143 | 1020.448239 | 1.32311244 | 0.043270067 | unchanged |
| hsa_circ_103564 | 362 | 303 | 329 | 404 | 480 | 435 | 331.5173694 | 439.7423262 | 1.326453353 | 0.017487696 | unchanged |
| hsa_circ_104823 | 146 | 113 | 146 | 173 | 181 | 180 | 135.2491269 | 178.1124571 | 1.316921308 | 0.018393126 | unchanged |
| hsa_circ_100612 | 257 | 229 | 243 | 271 | 338 | 363 | 242.9737015 | 323.9791038 | 1.333391646 | 0.046536178 | unchanged |
| hsa_circ_100978 | 126 | 119 | 134 | 163 | 178 | 161 | 126.3227082 | 167.3327641 | 1.32464516 | 0.003856115 | unchanged |
| hsa_circ_102010 | 131 | 137 | 129 | 164 | 166 | 197 | 132.277467 | 175.7864572 | 1.32892216 | 0.017173966 | unchanged |
| hsa_circ_104987 | 179 | 175 | 178 | 249 | 223 | 232 | 177.2653713 | 234.9914021 | 1.325647532 | 0.001710094 | unchanged |
| hsa_circ_103556 | 133 | 137 | 164 | 185 | 193 | 192 | 144.3774197 | 190.0099409 | 1.316064113 | 0.010417542 | unchanged |
| hsa_circ_001259 | 1611 | 1340 | 1146 | 1791 | 1793 | 1775 | 1365.507594 | 1786.401796 | 1.30823278 | 0.035681125 | unchanged |
| hsa_circ_102511 | 67 | 60 | 66 | 75 | 89 | 90 | 64.24578733 | 84.94869344 | 1.322245348 | 0.019264755 | unchanged |
| hsa_circ_104209 | 1076 | 1212 | 1075 | 1329 | 1546 | 1562 | 1120.964292 | 1478.811514 | 1.319231598 | 0.015175452 | unchanged |
| hsa_circ_101557 | 106 | 85 | 109 | 121 | 144 | 129 | 100.0823094 | 131.4321266 | 1.313240345 | 0.034015077 | unchanged |
| hsa_circ_102020 | 364 | 468 | 359 | 514 | 516 | 518 | 396.8238733 | 516.3529667 | 1.301214472 | 0.02794006 | unchanged |
| hsa_circ_103398 | 115 | 126 | 130 | 155 | 155 | 177 | 123.8915882 | 162.5022814 | 1.311649029 | 0.010744003 | unchanged |
| hsa_circ_101243 | 357 | 287 | 300 | 399 | 430 | 398 | 314.7341729 | 408.9995689 | 1.299507979 | 0.016565133 | unchanged |
| hsa_circ_103562 | 1223 | 1194 | 989 | 1575 | 1422 | 1429 | 1135.161931 | 1474.994339 | 1.299369102 | 0.018780405 | unchanged |
| hsa_circ_103379 | 162 | 130 | 161 | 188 | 191 | 208 | 151.0731504 | 195.6484021 | 1.295057405 | 0.021322548 | unchanged |
| hsa_circ_101848 | 288 | 240 | 267 | 329 | 364 | 338 | 264.9381437 | 343.7825566 | 1.297595551 | 0.010805821 | unchanged |
| hsa_circ_100232 | 188 | 147 | 152 | 230 | 194 | 205 | 162.2785941 | 209.6815695 | 1.292108615 | 0.045149549 | unchanged |
| hsa_circ_104540 | 1529 | 1427 | 1623 | 1833 | 2086 | 2016 | 1526.200405 | 1978.117071 | 1.296105718 | 0.008695382 | unchanged |
| hsa_circ_103998 | 654 | 831 | 753 | 864 | 1002 | 1027 | 746.1310023 | 964.3809592 | 1.292508898 | 0.038799367 | unchanged |
| hsa_circ_100436 | 105 | 87 | 100 | 131 | 125 | 122 | 97.48480803 | 125.8364834 | 1.29083173 | 0.009749397 | unchanged |
| hsa_circ_103679 | 211 | 218 | 249 | 273 | 288 | 315 | 225.9123663 | 291.9712924 | 1.292409518 | 0.017289249 | unchanged |
| hsa_circ_104731 | 57 | 52 | 51 | 62 | 82 | 64 | 53.27075516 | 69.3388026 | 1.301629804 | 0.067292142 | unchanged |
| hsa_circ_101711 | 1852 | 1540 | 1419 | 1961 | 2059 | 2164 | 1603.921776 | 2061.271646 | 1.285144747 | 0.032015526 | unchanged |
| hsa_circ_100549 | 560 | 604 | 490 | 679 | 737 | 713 | 551.283185 | 709.6350776 | 1.287242377 | 0.013116049 | unchanged |
| hsa_circ_103358 | 282 | 270 | 314 | 319 | 403 | 400 | 288.7357767 | 373.9091544 | 1.294987267 | 0.04980379 | unchanged |
| hsa_circ_101517 | 449 | 512 | 454 | 538 | 657 | 632 | 471.6060465 | 609.1598734 | 1.291671042 | 0.02906354 | unchanged |
| hsa_circ_104107 | 272 | 217 | 259 | 299 | 324 | 336 | 249.3067118 | 319.6423533 | 1.282124942 | 0.024328608 | unchanged |
| hsa_circ_001383 | 139 | 122 | 145 | 164 | 182 | 174 | 135.1720603 | 173.2966598 | 1.282044969 | 0.010904842 | unchanged |
| hsa_circ_100135 | 163 | 149 | 132 | 183 | 204 | 182 | 147.8291253 | 189.3151898 | 1.280635256 | 0.021471473 | unchanged |
| hsa_circ_104072 | 131 | 121 | 122 | 151 | 160 | 170 | 124.7744309 | 160.2319366 | 1.284172851 | 0.004869721 | unchanged |
| hsa_circ_103612 | 133 | 101 | 121 | 146 | 154 | 153 | 118.517229 | 151.0873857 | 1.274813687 | 0.02724003 | unchanged |
| hsa_circ_103565 | 691 | 751 | 606 | 851 | 910 | 850 | 682.7304907 | 870.2396103 | 1.274645885 | 0.015611123 | unchanged |
| hsa_circ_102130 | 225 | 205 | 208 | 283 | 263 | 269 | 212.7532883 | 271.4815743 | 1.276039381 | 0.002252455 | unchanged |
| hsa_circ_100731 | 950 | 952 | 894 | 1064 | 1389 | 1135 | 931.8681452 | 1195.968268 | 1.283409326 | 0.057985271 | unchanged |
| hsa_circ_104626 | 229 | 214 | 250 | 255 | 307 | 322 | 230.9538533 | 294.7710387 | 1.276320073 | 0.048947672 | unchanged |
| hsa_circ_100994 | 245 | 244 | 239 | 311 | 311 | 305 | 242.8392319 | 308.8504884 | 1.271831104 | 1.7532E-05 | unchanged |
| hsa_circ_100629 | 161 | 182 | 162 | 230 | 194 | 219 | 168.4280016 | 214.1359974 | 1.271380028 | 0.023295423 | unchanged |
| hsa_circ_102360 | 204 | 183 | 209 | 246 | 261 | 249 | 198.5589293 | 251.8806046 | 1.268543326 | 0.004145817 | unchanged |
| hsa_circ_100070 | 299 | 248 | 297 | 333 | 364 | 369 | 280.9975366 | 355.4023912 | 1.264788281 | 0.020767412 | unchanged |
| hsa_circ_105045 | 73 | 65 | 62 | 86 | 90 | 78 | 66.82306762 | 84.71620782 | 1.267768913 | 0.01940227 | unchanged |
| hsa_circ_103269 | 2340 | 2423 | 2003 | 2627 | 3004 | 2934 | 2255.414194 | 2855.303529 | 1.265977458 | 0.025581941 | unchanged |
| hsa_circ_102510 | 63 | 57 | 70 | 73 | 84 | 82 | 63.18217918 | 79.80767488 | 1.263135839 | 0.027850464 | unchanged |
| hsa_circ_101224 | 129 | 139 | 134 | 159 | 173 | 177 | 134.2109872 | 169.7711507 | 1.264957172 | 0.005134949 | unchanged |
| hsa_circ_101709 | 75 | 61 | 75 | 82 | 92 | 91 | 70.25790615 | 88.21976983 | 1.25565612 | 0.035132265 | unchanged |
| hsa_circ_100875 | 257 | 268 | 285 | 345 | 333 | 340 | 270.1504555 | 339.5032782 | 1.256719251 | 0.001434935 | unchanged |
| hsa_circ_101429 | 182 | 142 | 152 | 185 | 203 | 207 | 158.6213262 | 198.595248 | 1.252008496 | 0.044253466 | unchanged |
| hsa_circ_102715 | 1493 | 1663 | 1568 | 2254 | 1783 | 1918 | 1574.714287 | 1985.08797 | 1.260601994 | 0.050401742 | unchanged |
| hsa_circ_100515 | 62 | 58 | 61 | 79 | 73 | 75 | 60.1813808 | 75.45259384 | 1.253753118 | 0.002728529 | unchanged |
| hsa_circ_104288 | 115 | 109 | 101 | 132 | 139 | 136 | 108.4609327 | 135.7709405 | 1.251795804 | 0.003708274 | unchanged |
| hsa_circ_102712 | 156 | 132 | 139 | 174 | 176 | 180 | 141.9070111 | 176.6313657 | 1.24469795 | 0.009161783 | unchanged |
| hsa_circ_102008 | 494 | 487 | 410 | 561 | 566 | 603 | 463.5727284 | 576.5188116 | 1.243642639 | 0.019572511 | unchanged |
| hsa_circ_101827 | 150 | 163 | 173 | 196 | 208 | 200 | 162.0011905 | 201.4811669 | 1.243701767 | 0.006468797 | unchanged |
| hsa_circ_103311 | 599 | 567 | 629 | 662 | 809 | 765 | 598.300875 | 745.5044208 | 1.246035986 | 0.035206912 | unchanged |
| hsa_circ_101267 | 255 | 215 | 201 | 293 | 274 | 265 | 223.8223859 | 276.9488775 | 1.237360045 | 0.04309884 | unchanged |
| hsa_circ_100075 | 472 | 432 | 451 | 560 | 554 | 566 | 451.6152499 | 559.7808907 | 1.239508389 | 0.000818348 | unchanged |
| hsa_circ_103586 | 81 | 64 | 79 | 92 | 91 | 93 | 74.82253589 | 92.25109399 | 1.232931936 | 0.030787236 | unchanged |
| hsa_circ_101058 | 269 | 276 | 258 | 295 | 341 | 361 | 267.4974897 | 332.2208091 | 1.241958605 | 0.03245175 | unchanged |
| hsa_circ_104570 | 108 | 88 | 102 | 127 | 126 | 114 | 99.33811611 | 122.4338851 | 1.232496547 | 0.033589841 | unchanged |
| hsa_circ_400071 | 12608 | 12408 | 12991 | 14775 | 16297 | 15888 | 12668.97816 | 15653.33929 | 1.235564471 | 0.003566628 | unchanged |
| hsa_circ_104294 | 51 | 55 | 51 | 60 | 70 | 64 | 52.52086475 | 64.86277011 | 1.234990521 | 0.01880766 | unchanged |
| hsa_circ_101271 | 325 | 316 | 299 | 363 | 400 | 395 | 313.267263 | 386.0232429 | 1.232248909 | 0.006348223 | unchanged |
| hsa_circ_103611 | 204 | 164 | 165 | 219 | 219 | 216 | 177.7497175 | 217.76237 | 1.225106701 | 0.040389824 | unchanged |
| hsa_circ_101605 | 73 | 69 | 67 | 89 | 89 | 81 | 69.98879978 | 86.21081193 | 1.231780116 | 0.008839926 | unchanged |
| hsa_circ_101184 | 427 | 441 | 468 | 496 | 580 | 569 | 445.3644831 | 548.198336 | 1.23089819 | 0.023642793 | unchanged |
| hsa_circ_102857 | 136 | 127 | 133 | 153 | 159 | 175 | 132.0276797 | 162.4351998 | 1.230311705 | 0.012796185 | unchanged |
| hsa_circ_104420 | 90 | 86 | 88 | 103 | 109 | 111 | 88.11530271 | 107.9460893 | 1.225054968 | 0.001896158 | unchanged |
| hsa_circ_100594 | 75 | 69 | 82 | 85 | 93 | 98 | 75.20478505 | 91.90545562 | 1.222069255 | 0.036407907 | unchanged |
| hsa_circ_102580 | 255 | 228 | 247 | 289 | 296 | 306 | 243.3270644 | 296.9972902 | 1.220568254 | 0.004553335 | unchanged |
| hsa_circ_100841 | 116 | 100 | 122 | 137 | 130 | 144 | 112.406729 | 136.9119621 | 1.218005036 | 0.032182223 | unchanged |
| hsa_circ_101288 | 98 | 82 | 93 | 108 | 109 | 115 | 91.11982357 | 110.9487566 | 1.217613821 | 0.020340213 | unchanged |
| hsa_circ_104467 | 244 | 225 | 245 | 282 | 286 | 302 | 238.1462688 | 289.887293 | 1.217265735 | 0.004325188 | unchanged |
| hsa_circ_101050 | 106 | 94 | 104 | 111 | 131 | 128 | 101.565078 | 123.244967 | 1.213458104 | 0.039500948 | unchanged |
| hsa_circ_105007 | 144 | 143 | 140 | 171 | 176 | 170 | 142.5251497 | 172.2646701 | 1.208661563 | 0.000268136 | unchanged |
| hsa_circ_101258 | 3261 | 3099 | 3320 | 3508 | 4000 | 4213 | 3226.900698 | 3907.048346 | 1.210774273 | 0.03593037 | unchanged |
| hsa_circ_102784 | 2377 | 2785 | 2408 | 2866 | 2997 | 3260 | 2523.578329 | 3040.873336 | 1.204984724 | 0.041546715 | unchanged |
| hsa_circ_101558 | 82 | 79 | 77 | 87 | 102 | 98 | 79.20500379 | 95.66807775 | 1.207853963 | 0.021987578 | unchanged |
| hsa_circ_104316 | 55 | 46 | 55 | 62 | 61 | 64 | 52.05231773 | 62.40964534 | 1.198979182 | 0.030535558 | unchanged |
| hsa_circ_100375 | 54363 | 44787 | 46540 | 58461 | 58461 | 57331 | 48563.38093 | 58084.29824 | 1.196051369 | 0.032638165 | unchanged |
| hsa_circ_101381 | 4845 | 4090 | 4334 | 4891 | 5653 | 5353 | 4423.083849 | 5299.08858 | 1.198052934 | 0.049318616 | unchanged |
| hsa_circ_100773 | 71 | 64 | 60 | 74 | 80 | 78 | 65.10538765 | 77.57645096 | 1.19155194 | 0.026554906 | unchanged |
| hsa_circ_100039 | 1265 | 1457 | 1299 | 1470 | 1618 | 1704 | 1340.550062 | 1597.128122 | 1.191397596 | 0.046924184 | unchanged |
| hsa_circ_102630 | 73 | 75 | 88 | 91 | 92 | 96 | 78.56186124 | 92.90434277 | 1.182562904 | 0.045340887 | unchanged |
| hsa_circ_102339 | 122 | 111 | 121 | 145 | 136 | 139 | 117.8177634 | 139.6636885 | 1.185421319 | 0.007619769 | unchanged |
| hsa_circ_104539 | 1054 | 962 | 962 | 1084 | 1232 | 1197 | 992.9226265 | 1171.019849 | 1.179366667 | 0.030117612 | unchanged |
| hsa_circ_101665 | 335 | 357 | 346 | 398 | 419 | 406 | 346.1130311 | 407.6140851 | 1.177690663 | 0.00242191 | unchanged |
| hsa_circ_103371 | 88 | 86 | 79 | 106 | 97 | 95 | 84.2770617 | 99.19154957 | 1.176969718 | 0.027960877 | unchanged |
| hsa_circ_100907 | 147 | 148 | 143 | 168 | 178 | 169 | 146.0363738 | 171.9091239 | 1.177166478 | 0.001654668 | unchanged |
| hsa_circ_102368 | 125 | 134 | 137 | 145 | 153 | 167 | 131.9439698 | 155.1635851 | 1.175980875 | 0.031684466 | unchanged |
| hsa_circ_100377 | 60 | 52 | 60 | 69 | 65 | 67 | 57.1707571 | 66.88924926 | 1.169990615 | 0.032326435 | unchanged |
| hsa_circ_101903 | 221 | 217 | 234 | 240 | 270 | 279 | 224.0472941 | 263.0537922 | 1.174099394 | 0.036707682 | unchanged |
| hsa_circ_400082 | 119 | 108 | 104 | 134 | 130 | 123 | 110.2488018 | 129.0837099 | 1.170840025 | 0.025823152 | unchanged |
| hsa_circ_100471 | 246 | 222 | 219 | 266 | 273 | 265 | 229.1125556 | 268.0375582 | 1.169894673 | 0.010829019 | unchanged |
| hsa_circ_101123 | 77 | 76 | 76 | 84 | 94 | 89 | 76.1644701 | 89.08644953 | 1.16965889 | 0.011212074 | unchanged |
| hsa_circ_103418 | 96 | 84 | 93 | 105 | 107 | 108 | 91.07301397 | 106.2240941 | 1.166361905 | 0.017466954 | unchanged |
| hsa_circ_100461 | 79 | 83 | 90 | 92 | 105 | 98 | 83.97175526 | 98.05402081 | 1.167702408 | 0.042714859 | unchanged |
| hsa_circ_103170 | 84 | 72 | 81 | 88 | 92 | 97 | 79.12676242 | 92.26796998 | 1.166077913 | 0.039162795 | unchanged |
| hsa_circ_102811 | 177 | 159 | 154 | 181 | 192 | 198 | 163.3232063 | 190.2780246 | 1.165039732 | 0.03528079 | unchanged |
| hsa_circ_102381 | 1914 | 1775 | 1709 | 1928 | 2146 | 2225 | 1799.405433 | 2099.690461 | 1.166880138 | 0.049322311 | unchanged |
| hsa_circ_101667 | 96 | 91 | 97 | 102 | 113 | 117 | 94.94528524 | 110.8247175 | 1.167248244 | 0.029295264 | unchanged |
| hsa_circ_104542 | 60 | 54 | 53 | 68 | 62 | 64 | 55.58773406 | 64.71700572 | 1.164231765 | 0.036834332 | unchanged |
| hsa_circ_102605 | 620 | 677 | 590 | 787 | 705 | 706 | 629.0996215 | 732.5064445 | 1.164372731 | 0.05012645 | unchanged |
| hsa_circ_103449 | 84 | 75 | 80 | 93 | 94 | 90 | 79.47873072 | 92.40968847 | 1.162697084 | 0.011300486 | unchanged |
| hsa_circ_101999 | 149 | 155 | 157 | 180 | 170 | 187 | 153.9462632 | 178.9564541 | 1.162460526 | 0.011045569 | unchanged |
| hsa_circ_102524 | 72 | 64 | 68 | 80 | 75 | 82 | 68.12164018 | 78.99395192 | 1.159601438 | 0.023332511 | unchanged |
| hsa_circ_100334 | 4142 | 4365 | 3701 | 4805 | 4591 | 4683 | 4069.454493 | 4692.908408 | 1.153203314 | 0.03810841 | unchanged |
| hsa_circ_104708 | 79 | 74 | 77 | 85 | 92 | 90 | 77.07848994 | 88.82709642 | 1.152423932 | 0.011287827 | unchanged |
| hsa_circ_104863 | 82 | 76 | 81 | 88 | 90 | 95 | 79.21130545 | 91.095462 | 1.150031065 | 0.015831032 | unchanged |
| hsa_circ_100348 | 140 | 129 | 152 | 164 | 159 | 159 | 140.3076553 | 160.6017952 | 1.144640289 | 0.040526444 | unchanged |
| hsa_circ_100167 | 73 | 69 | 67 | 86 | 75 | 80 | 69.91250381 | 80.23116101 | 1.147593873 | 0.048509714 | unchanged |
| hsa_circ_100915 | 81 | 82 | 79 | 90 | 97 | 90 | 80.71576239 | 92.47235962 | 1.145654292 | 0.009261462 | unchanged |
| hsa_circ_104335 | 84 | 79 | 74 | 96 | 88 | 87 | 78.96811857 | 90.23091745 | 1.142624632 | 0.048955874 | unchanged |
| hsa_circ_000990 | 85 | 78 | 81 | 88 | 95 | 95 | 81.23045448 | 92.69380044 | 1.141121283 | 0.018451175 | unchanged |
| hsa_circ_100547 | 79 | 89 | 84 | 92 | 98 | 95 | 83.79905855 | 94.88501543 | 1.132292141 | 0.031976484 | unchanged |
| hsa_circ_102026 | 68 | 63 | 61 | 72 | 74 | 69 | 63.99990064 | 72.05748635 | 1.125899972 | 0.038566751 | unchanged |
| hsa_circ_102902 | 199 | 179 | 189 | 203 | 221 | 214 | 188.8691012 | 212.694468 | 1.12614751 | 0.034335234 | unchanged |
| hsa_circ_000791 | 59995 | 53794 | 53273 | 63622 | 61809 | 61809 | 55687.12996 | 62413.76212 | 1.120793299 | 0.039932464 | unchanged |
| hsa_circ_102300 | 334 | 343 | 325 | 372 | 375 | 368 | 334.1019547 | 372.0550254 | 1.113597272 | 0.002330863 | unchanged |
| hsa_circ_103530 | 64 | 69 | 68 | 74 | 75 | 74 | 67.08324013 | 74.12307893 | 1.104941842 | 0.008931856 | unchanged |
| hsa_circ_102434 | 4112 | 4157 | 4333 | 4749 | 4695 | 4425 | 4200.785302 | 4622.783003 | 1.10045686 | 0.025046107 | unchanged |
| hsa_circ_100639 | 88 | 95 | 87 | 99 | 96 | 101 | 89.91858276 | 98.71269084 | 1.097800786 | 0.039901108 | unchanged |
| hsa_circ_102947 | 110 | 112 | 112 | 124 | 120 | 123 | 111.2851968 | 121.9413114 | 1.095755006 | 0.00222695 | unchanged |
| hsa_circ_101262 | 74 | 68 | 69 | 79 | 74 | 78 | 70.23277691 | 76.78102691 | 1.093236382 | 0.048699439 | unchanged |
| hsa_circ_102623 | 76 | 74 | 72 | 82 | 78 | 81 | 74.03774496 | 80.31855379 | 1.084832525 | 0.029895811 | unchanged |
| hsa_circ_104858 | 722 | 688 | 719 | 762 | 759 | 777 | 709.5476548 | 766.0059636 | 1.079569439 | 0.010530302 | unchanged |
| hsa_circ_101157 | 75 | 80 | 76 | 82 | 84 | 82 | 76.87871647 | 82.90393326 | 1.078373015 | 0.026680776 | unchanged |
| hsa_circ_104795 | 67 | 67 | 67 | 72 | 71 | 73 | 66.85452228 | 72.03799199 | 1.077533569 | 0.002619032 | unchanged |
| hsa_circ_103845 | 65 | 62 | 62 | 67 | 67 | 67 | 62.89633232 | 67.07924004 | 1.066504795 | 0.0159269 | unchanged |
| hsa_circ_103867 | 70 | 68 | 69 | 75 | 75 | 71 | 69.09030393 | 73.49461589 | 1.063747179 | 0.045791246 | unchanged |
| hsa_circ_101457 | 2538 | 2486 | 2433 | 2583 | 2699 | 2633 | 2485.940606 | 2638.439234 | 1.061344437 | 0.028133625 | unchanged |
| hsa_circ_101134 | 73 | 71 | 72 | 76 | 76 | 78 | 72.27525151 | 76.64051959 | 1.060397826 | 0.008720596 | unchanged |
| hsa_circ_103907 | 61 | 60 | 62 | 64 | 65 | 65 | 60.79773953 | 64.37089749 | 1.05877123 | 0.00364713 | unchanged |
| hsa_circ_101415 | 190 | 188 | 195 | 197 | 202 | 208 | 191.0914067 | 202.2185441 | 1.058229397 | 0.037888499 | unchanged |
| hsa_circ_101771 | 150 | 150 | 145 | 143 | 136 | 137 | 148.2825666 | 138.7167841 | 0.9354895 | 0.027927073 | unchanged |
| hsa_circ_000332 | 67 | 67 | 71 | 64 | 62 | 64 | 68.42868755 | 63.27060129 | 0.924620997 | 0.039839711 | unchanged |
| hsa_circ_101464 | 86 | 79 | 86 | 75 | 75 | 78 | 83.93948371 | 75.96814396 | 0.905034682 | 0.034268386 | unchanged |
| hsa_circ_103028 | 294 | 286 | 275 | 249 | 268 | 253 | 285.303466 | 256.8035476 | 0.900106652 | 0.022581839 | unchanged |
| hsa_circ_102468 | 66 | 72 | 67 | 60 | 60 | 64 | 68.47366417 | 61.50452831 | 0.89822166 | 0.041646802 | unchanged |
| hsa_circ_101646 | 148 | 156 | 155 | 137 | 129 | 142 | 152.8931091 | 135.9428219 | 0.889136357 | 0.020911625 | unchanged |
| hsa_circ_102089 | 5052 | 5272 | 5096 | 4536 | 4474 | 4573 | 5140.105569 | 4527.742878 | 0.880865737 | 0.00112165 | unchanged |
| hsa_circ_101019 | 69 | 69 | 73 | 61 | 61 | 63 | 70.38752133 | 61.77614843 | 0.877657677 | 0.003566365 | unchanged |
| hsa_circ_102493 | 113 | 103 | 106 | 92 | 93 | 97 | 107.3266749 | 93.95116289 | 0.875375698 | 0.014223868 | unchanged |
| hsa_circ_102198 | 197 | 196 | 216 | 178 | 178 | 176 | 203.3277263 | 177.4272155 | 0.872616926 | 0.015379612 | unchanged |
| hsa_circ_101968 | 378 | 397 | 399 | 340 | 352 | 334 | 391.4124347 | 341.825201 | 0.873312063 | 0.004268255 | unchanged |
| hsa_circ_001097 | 149 | 136 | 143 | 120 | 129 | 125 | 142.7051362 | 124.5740112 | 0.872946935 | 0.01690286 | unchanged |
| hsa_circ_100799 | 175 | 186 | 190 | 168 | 155 | 155 | 183.5819892 | 159.6204342 | 0.869477638 | 0.016601403 | unchanged |
| hsa_circ_101970 | 86 | 91 | 83 | 74 | 74 | 75 | 86.41241096 | 74.49696026 | 0.862109498 | 0.007213402 | unchanged |
| hsa_circ_100704 | 122 | 122 | 113 | 106 | 99 | 102 | 119.1765126 | 102.5081399 | 0.8601371 | 0.010722632 | unchanged |
| hsa_circ_100555 | 121 | 125 | 136 | 105 | 113 | 110 | 127.0918544 | 109.1248804 | 0.858630011 | 0.023057843 | unchanged |
| hsa_circ_100409 | 187 | 213 | 182 | 164 | 166 | 168 | 194.238169 | 166.1763822 | 0.855528978 | 0.044588044 | unchanged |
| hsa_circ_400066 | 1946 | 2046 | 1942 | 1818 | 1563 | 1677 | 1977.769846 | 1685.75596 | 0.852351937 | 0.022750255 | unchanged |
| hsa_circ_100666 | 237 | 263 | 240 | 198 | 214 | 217 | 246.6687214 | 209.7148169 | 0.850188122 | 0.02191126 | unchanged |
| hsa_circ_101435 | 139 | 116 | 119 | 104 | 106 | 106 | 124.7286292 | 105.3414341 | 0.844564995 | 0.059260985 | unchanged |
| hsa_circ_104496 | 80 | 93 | 91 | 71 | 72 | 80 | 87.9752162 | 74.2729323 | 0.844248363 | 0.045358071 | unchanged |
| hsa_circ_105039 | 59 | 66 | 67 | 56 | 55 | 51 | 63.77812067 | 53.81560579 | 0.843794161 | 0.031361217 | unchanged |
| hsa_circ_001190 | 164 | 191 | 166 | 147 | 149 | 143 | 173.8490766 | 146.1323129 | 0.840569969 | 0.03503959 | unchanged |
| hsa_circ_001484 | 93 | 82 | 87 | 81 | 70 | 70 | 87.08044269 | 73.40247253 | 0.842927186 | 0.046703557 | unchanged |
| hsa_circ_102877 | 84 | 85 | 74 | 64 | 70 | 67 | 80.8317229 | 67.27770923 | 0.83231814 | 0.02423342 | unchanged |
| hsa_circ_102272 | 140 | 165 | 136 | 126 | 120 | 117 | 146.9238717 | 121.2004701 | 0.824920203 | 0.057040846 | unchanged |
| hsa_circ_101797 | 88 | 92 | 102 | 70 | 83 | 79 | 94.0898386 | 77.63755827 | 0.825142857 | 0.045044337 | unchanged |
| hsa_circ_101461 | 229 | 211 | 233 | 195 | 190 | 171 | 224.4132186 | 185.0065714 | 0.824401399 | 0.016292661 | unchanged |
| hsa_circ_100424 | 116 | 121 | 125 | 106 | 96 | 94 | 120.5452136 | 98.88021472 | 0.820274914 | 0.009737602 | unchanged |
| hsa_circ_101624 | 65 | 81 | 70 | 61 | 55 | 60 | 71.82899709 | 58.48397731 | 0.814211247 | 0.057739201 | unchanged |
| hsa_circ_101883 | 119 | 100 | 113 | 94 | 94 | 81 | 110.4124499 | 89.75814016 | 0.812934957 | 0.042719217 | unchanged |
| hsa_circ_100281 | 134 | 161 | 134 | 117 | 119 | 111 | 142.9018637 | 115.7389635 | 0.809919203 | 0.042659589 | unchanged |
| hsa_circ_100245 | 155 | 181 | 158 | 139 | 130 | 129 | 165.0057916 | 132.6451057 | 0.803881515 | 0.021214648 | unchanged |
| hsa_circ_100007 | 341 | 336 | 318 | 261 | 266 | 268 | 331.731469 | 264.9035899 | 0.798548268 | 0.000787348 | unchanged |
| hsa_circ_100509 | 66 | 84 | 70 | 56 | 57 | 60 | 73.41451819 | 57.6859855 | 0.78575719 | 0.051943905 | unchanged |
| hsa_circ_103514 | 94 | 105 | 109 | 75 | 80 | 87 | 102.6687055 | 80.91884267 | 0.788154894 | 0.017580863 | unchanged |
| hsa_circ_102118 | 295 | 320 | 306 | 238 | 278 | 214 | 307.3043435 | 243.216096 | 0.791450239 | 0.032918924 | unchanged |
| hsa_circ_101078 | 518 | 468 | 454 | 422 | 355 | 357 | 479.8757092 | 378.2687628 | 0.788264035 | 0.025639908 | unchanged |
| hsa_circ_103217 | 189 | 214 | 209 | 160 | 152 | 167 | 203.8656497 | 159.6311185 | 0.783021165 | 0.006831176 | unchanged |
| hsa_circ_102663 | 86 | 86 | 77 | 71 | 61 | 62 | 82.80312057 | 64.57459183 | 0.779856983 | 0.013436541 | unchanged |
| hsa_circ_104019 | 69 | 82 | 86 | 64 | 57 | 61 | 78.87615724 | 60.80693607 | 0.770916563 | 0.028068137 | unchanged |
| hsa_circ_000596 | 300 | 355 | 302 | 250 | 233 | 249 | 318.9415495 | 244.24559 | 0.765800475 | 0.016435406 | unchanged |
| hsa_circ_102484 | 154 | 157 | 179 | 133 | 124 | 119 | 163.5957873 | 125.2619012 | 0.765679259 | 0.012980475 | unchanged |
| hsa_circ_102131 | 238 | 310 | 234 | 197 | 193 | 200 | 260.8691735 | 197.0680916 | 0.755428819 | 0.062247261 | unchanged |
| hsa_circ_103158 | 102 | 87 | 83 | 77 | 64 | 65 | 90.61734448 | 68.9660066 | 0.761068502 | 0.041080278 | unchanged |
| hsa_circ_104285 | 304 | 363 | 285 | 262 | 231 | 227 | 317.3345821 | 240.2626063 | 0.757127082 | 0.040638775 | unchanged |
| hsa_circ_103174 | 110 | 137 | 116 | 104 | 85 | 87 | 121.0651991 | 91.83515171 | 0.758559457 | 0.044447613 | unchanged |
| hsa_circ_101000 | 142 | 157 | 142 | 124 | 104 | 105 | 147.0476294 | 111.2622423 | 0.756640843 | 0.012563777 | unchanged |
| hsa_circ_102741 | 4160 | 3505 | 4252 | 3272 | 2783 | 2920 | 3972.21286 | 2991.410735 | 0.753084198 | 0.023904333 | unchanged |
| hsa_circ_104077 | 110 | 97 | 88 | 75 | 73 | 73 | 98.58098 | 73.70032197 | 0.747611983 | 0.01852729 | unchanged |
| hsa_circ_104547 | 145 | 157 | 146 | 118 | 103 | 114 | 149.4252992 | 111.6349027 | 0.747095059 | 0.003167225 | unchanged |
| hsa_circ_103175 | 92 | 98 | 96 | 75 | 67 | 72 | 95.57646561 | 71.20697693 | 0.745026262 | 0.001205347 | unchanged |
| hsa_circ_100016 | 185 | 230 | 184 | 156 | 144 | 143 | 199.4826534 | 147.7003289 | 0.740416905 | 0.029883376 | unchanged |
| hsa_circ_102990 | 402 | 520 | 431 | 328 | 337 | 326 | 450.9658354 | 330.4403187 | 0.732739141 | 0.028027937 | unchanged |
| hsa_circ_000441 | 112 | 100 | 94 | 77 | 77 | 71 | 101.9127752 | 74.7910246 | 0.733872907 | 0.008192342 | unchanged |
| hsa_circ_100349 | 419 | 452 | 358 | 354 | 265 | 283 | 409.605748 | 300.570859 | 0.733805276 | 0.048288665 | unchanged |
| hsa_circ_102469 | 115 | 132 | 102 | 93 | 79 | 79 | 116.3115215 | 83.99032495 | 0.722115263 | 0.031396933 | unchanged |
| hsa_circ_100190 | 105 | 140 | 123 | 83 | 90 | 87 | 122.2804589 | 86.54073696 | 0.707723358 | 0.025838546 | unchanged |
| hsa_circ_103109 | 91 | 94 | 98 | 69 | 63 | 70 | 94.70424341 | 67.39087066 | 0.711592936 | 0.000725027 | unchanged |
| hsa_circ_100632 | 92 | 121 | 94 | 66 | 76 | 74 | 102.1803294 | 72.12919291 | 0.705900963 | 0.035924563 | unchanged |
| hsa_circ_103414 | 952 | 747 | 757 | 629 | 537 | 567 | 818.6159759 | 577.864633 | 0.705904417 | 0.028706532 | unchanged |
| hsa_circ_101937 | 209 | 251 | 249 | 162 | 163 | 164 | 236.3313517 | 163.056399 | 0.689948235 | 0.005837717 | unchanged |
| hsa_circ_104419 | 73 | 109 | 98 | 67 | 63 | 61 | 93.35749986 | 63.69120877 | 0.682229161 | 0.04849551 | unchanged |
| hsa_circ_104948 | 104 | 143 | 143 | 78 | 94 | 94 | 130.3270273 | 88.80290261 | 0.681385162 | 0.041482669 | unchanged |
| hsa_circ_000881 | 12729 | 11817 | 15319 | 10370 | 8394 | 8533 | 13288.45213 | 9098.903074 | 0.684722568 | 0.026906646 | unchanged |
| hsa_circ_103360 | 166 | 214 | 181 | 119 | 141 | 121 | 186.8691555 | 127.0320965 | 0.679791676 | 0.020285257 | unchanged |
| hsa_circ_103415 | 583 | 475 | 470 | 388 | 322 | 322 | 509.3076595 | 343.9838355 | 0.675394978 | 0.018136302 | unchanged |
| hsa_circ_104469 | 213 | 328 | 234 | 163 | 176 | 176 | 258.4348782 | 171.7776815 | 0.664684592 | 0.071295321 | unchanged |
| hsa_circ_104886 | 248 | 237 | 171 | 150 | 141 | 142 | 218.6815158 | 144.6509024 | 0.661468355 | 0.038132987 | unchanged |
| hsa_circ_102910 | 995 | 677 | 942 | 654 | 533 | 539 | 871.5132742 | 575.1899122 | 0.659989847 | 0.048975553 | unchanged |
| hsa_circ_100311 | 674 | 902 | 872 | 432 | 617 | 578 | 816.2390929 | 542.2608586 | 0.66434071 | 0.039477899 | unchanged |
| hsa_circ_101213 | 12497 | 18369 | 19149 | 11236 | 10207 | 10262 | 16671.78212 | 10568.24079 | 0.633899886 | 0.045414472 | unchanged |
| hsa_circ_104261 | 194 | 249 | 194 | 150 | 127 | 131 | 212.3078506 | 136.1686338 | 0.641373522 | 0.017451747 | unchanged |
| hsa_circ_103977 | 84 | 98 | 61 | 59 | 47 | 47 | 81.03582532 | 50.95607597 | 0.628809243 | 0.058290226 | unchanged |
| hsa_circ_101137 | 97 | 101 | 75 | 64 | 53 | 55 | 90.90085759 | 57.48608872 | 0.632404251 | 0.019597029 | unchanged |
| hsa_circ_000950 | 338 | 316 | 237 | 223 | 163 | 171 | 296.8330757 | 185.7273395 | 0.62569624 | 0.036682077 | unchanged |
| hsa_circ_103178 | 214 | 306 | 245 | 191 | 144 | 141 | 254.9133339 | 158.801182 | 0.622961458 | 0.037109284 | unchanged |
| hsa_circ_001036 | 136 | 145 | 170 | 85 | 95 | 97 | 150.3653781 | 92.02607621 | 0.612016392 | 0.005485004 | unchanged |
| hsa_circ_101786 | 170 | 179 | 143 | 105 | 95 | 99 | 164.0384416 | 99.53337847 | 0.606768618 | 0.004289323 | unchanged |
| hsa_circ_101025 | 264 | 273 | 330 | 180 | 176 | 168 | 289.0064255 | 174.6889732 | 0.604446676 | 0.005592915 | unchanged |
| hsa_circ_100477 | 1698 | 2309 | 1944 | 1126 | 1230 | 1217 | 1983.544961 | 1191.146564 | 0.600514023 | 0.01176102 | unchanged |
| hsa_circ_101187 | 730 | 730 | 433 | 398 | 304 | 349 | 631.2619261 | 350.1604192 | 0.554699095 | 0.05185894 | unchanged |
| hsa_circ_103818 | 1065 | 1812 | 1501 | 655 | 899 | 893 | 1459.567017 | 815.8450849 | 0.558963772 | 0.049502634 | unchanged |
| hsa_circ_104310 | 4082 | 3205 | 3513 | 2578 | 1818 | 1710 | 3599.644847 | 2035.339292 | 0.565427807 | 0.014007908 | unchanged |
| hsa_circ_101103 | 173 | 289 | 205 | 135 | 115 | 115 | 222.2411482 | 121.66184 | 0.547431657 | 0.046184704 | unchanged |
| hsa_circ_103225 | 245 | 281 | 216 | 165 | 119 | 123 | 247.075066 | 135.7003013 | 0.549227017 | 0.009635801 | unchanged |
| hsa_circ_000424 | 323 | 312 | 256 | 183 | 146 | 152 | 297.0804899 | 160.2817621 | 0.539523017 | 0.004386504 | unchanged |
| hsa_circ_100476 | 1298 | 1573 | 1994 | 817 | 854 | 870 | 1621.674753 | 847.0180655 | 0.522310694 | 0.018900047 | unchanged |
| hsa_circ_101242 | 6831 | 13079 | 11822 | 5484 | 5087 | 5418 | 10577.42883 | 5329.604104 | 0.503865749 | 0.051651374 | unchanged |
| hsa_circ_103110 | 188 | 177 | 352 | 116 | 116 | 119 | 239.1405212 | 116.9583741 | 0.489078026 | 0.097451139 | unchanged |
| hsa_circ_000993 | 4067 | 2425 | 2870 | 2045 | 1410 | 1315 | 3120.458265 | 1589.837519 | 0.509488474 | 0.047412757 | unchanged |
| hsa_circ_101004 | 719 | 1072 | 925 | 416 | 495 | 462 | 905.3360394 | 457.6603973 | 0.505514392 | 0.012904478 | unchanged |
| hsa_circ_001166 | 108 | 166 | 144 | 73 | 66 | 68 | 139.4252837 | 68.89338547 | 0.494124047 | 0.01496705 | unchanged |
| hsa_circ_000006 | 72 | 65 | 63 | 70 | 62 | 59 | 66.48312883 | 63.45573987 | 0.954463801 | 0.506325182 | unchanged |
| hsa_circ_000010 | 766 | 943 | 604 | 862 | 836 | 870 | 771.1468411 | 856.0310801 | 1.110075325 | 0.43625022 | unchanged |
| hsa_circ_000031 | 667 | 515 | 658 | 861 | 746 | 760 | 613.3671479 | 789.0255931 | 1.28638385 | 0.045683503 | unchanged |
| hsa_circ_000041 | 160 | 174 | 253 | 160 | 304 | 293 | 195.4892568 | 252.4167667 | 1.291205311 | 0.355387304 | unchanged |
| hsa_circ_000042 | 140 | 281 | 162 | 150 | 111 | 116 | 194.5696347 | 125.6312542 | 0.645687877 | 0.204216157 | unchanged |
| hsa_circ_000046 | 3340 | 1533 | 1447 | 2484 | 1091 | 1103 | 2106.725372 | 1559.284973 | 0.740146292 | 0.516872685 | unchanged |
| hsa_circ_000082 | 271 | 127 | 141 | 195 | 116 | 116 | 179.5549135 | 142.4653928 | 0.793436336 | 0.520015771 | unchanged |
| hsa_circ_000085 | 84 | 66 | 62 | 86 | 61 | 65 | 70.44362303 | 70.71787411 | 1.0038932 | 0.98011009 | unchanged |
| hsa_circ_000094 | 549 | 379 | 342 | 488 | 449 | 449 | 423.5608651 | 461.9459889 | 1.090624812 | 0.587233833 | unchanged |
| hsa_circ_000104 | 163 | 191 | 188 | 164 | 130 | 144 | 180.5389948 | 146.0771868 | 0.809117094 | 0.059243016 | unchanged |
| hsa_circ_000113 | 90 | 128 | 93 | 87 | 78 | 83 | 103.6758654 | 82.48033349 | 0.795559633 | 0.161823626 | unchanged |
| hsa_circ_000162 | 503 | 318 | 392 | 706 | 498 | 512 | 404.5248544 | 571.9302304 | 1.413832115 | 0.122972605 | unchanged |
| hsa_circ_000166 | 2714 | 4218 | 5717 | 2843 | 6408 | 6128 | 4216.041907 | 5126.256165 | 1.215893076 | 0.560571123 | unchanged |
| hsa_circ_000167 | 243 | 300 | 353 | 274 | 357 | 369 | 298.802715 | 333.2550086 | 1.11530114 | 0.472523789 | unchanged |
| hsa_circ_000178 | 4345 | 2228 | 2323 | 2931 | 1742 | 1727 | 2964.929684 | 2133.284615 | 0.719505972 | 0.355769909 | unchanged |
| hsa_circ_000191 | 180 | 226 | 139 | 168 | 148 | 141 | 181.5846113 | 152.2061139 | 0.838210424 | 0.325191155 | unchanged |
| hsa_circ_000195 | 72 | 118 | 101 | 75 | 106 | 99 | 96.79847921 | 93.62372839 | 0.967202472 | 0.856168058 | unchanged |
| hsa_circ_000200 | 1462 | 478 | 327 | 670 | 240 | 211 | 755.3710567 | 373.7408828 | 0.49477787 | 0.378471155 | unchanged |
| hsa_circ_000212 | 122 | 95 | 113 | 115 | 104 | 110 | 110.3587708 | 110.0152829 | 0.996887534 | 0.969778513 | unchanged |
| hsa_circ_000230 | 160 | 104 | 263 | 134 | 118 | 121 | 175.9025901 | 124.4347451 | 0.707407122 | 0.333158852 | unchanged |
| hsa_circ_000250 | 389 | 222 | 325 | 489 | 369 | 370 | 312.0972597 | 409.4051636 | 1.311787114 | 0.196421171 | unchanged |
| hsa_circ_000274 | 4447 | 3017 | 4196 | 4389 | 3159 | 3228 | 3886.825297 | 3592.201632 | 0.924199406 | 0.646285003 | unchanged |
| hsa_circ_000280 | 525 | 1125 | 1185 | 566 | 1015 | 1085 | 945.1491286 | 888.727491 | 0.940303984 | 0.842562483 | unchanged |
| hsa_circ_000294 | 171 | 274 | 249 | 149 | 197 | 208 | 231.4600768 | 184.4979837 | 0.797104997 | 0.260196446 | unchanged |
| hsa_circ_000295 | 86 | 198 | 120 | 85 | 107 | 109 | 134.7422268 | 100.3006264 | 0.744388963 | 0.36987049 | unchanged |
| hsa_circ_000300 | 868 | 1183 | 1308 | 1038 | 1492 | 1467 | 1119.936021 | 1332.138073 | 1.189476942 | 0.342324261 | unchanged |
| hsa_circ_000329 | 99 | 146 | 104 | 95 | 102 | 100 | 116.2426045 | 99.11194309 | 0.852630096 | 0.318954782 | unchanged |
| hsa_circ_000368 | 87 | 82 | 107 | 95 | 108 | 109 | 91.97527556 | 104.1156358 | 1.13199591 | 0.244568297 | unchanged |
| hsa_circ_000390 | 89 | 105 | 118 | 95 | 114 | 105 | 104.0211797 | 104.4057153 | 1.003696705 | 0.971582506 | unchanged |
| hsa_circ_000401 | 57 | 132 | 66 | 52 | 56 | 55 | 84.63370646 | 54.69471921 | 0.646252203 | 0.273563239 | unchanged |
| hsa_circ_000404 | 60 | 89 | 66 | 58 | 68 | 64 | 71.45765139 | 63.40211285 | 0.887268356 | 0.43645529 | unchanged |
| hsa_circ_000410 | 5946 | 8905 | 8632 | 6687 | 9785 | 9748 | 7827.647548 | 8739.723101 | 1.116519752 | 0.54885061 | unchanged |
| hsa_circ_000422 | 145 | 140 | 129 | 163 | 96 | 93 | 138.1963414 | 117.6759357 | 0.851512671 | 0.427394331 | unchanged |
| hsa_circ_000425 | 82 | 95 | 80 | 80 | 80 | 82 | 85.61411233 | 80.85784363 | 0.944445272 | 0.384540205 | unchanged |
| hsa_circ_000442 | 307 | 337 | 259 | 263 | 229 | 225 | 300.6776536 | 238.7709005 | 0.794109232 | 0.07437099 | unchanged |
| hsa_circ_000446 | 523 | 513 | 404 | 563 | 259 | 268 | 479.8672988 | 363.545956 | 0.757596854 | 0.338082591 | unchanged |
| hsa_circ_000454 | 79 | 151 | 53 | 84 | 54 | 55 | 94.47930344 | 64.45155518 | 0.682176443 | 0.386864663 | unchanged |
| hsa_circ_000455 | 1919 | 1391 | 746 | 1575 | 1023 | 1031 | 1352.1968 | 1210.046216 | 0.894874338 | 0.73078666 | unchanged |
| hsa_circ_000465 | 65 | 61 | 60 | 60 | 65 | 65 | 62.02445805 | 63.70184415 | 1.027043946 | 0.491000942 | unchanged |
| hsa_circ_000466 | 574 | 706 | 607 | 519 | 603 | 631 | 628.857921 | 584.5877015 | 0.929602192 | 0.443111169 | unchanged |
| hsa_circ_000481 | 2027 | 2980 | 5702 | 2269 | 3943 | 4230 | 3569.549238 | 3480.848791 | 0.975150799 | 0.947229281 | unchanged |
| hsa_circ_000482 | 144 | 174 | 188 | 156 | 203 | 199 | 168.6706626 | 185.9982347 | 1.102730207 | 0.430652709 | unchanged |
| hsa_circ_000486 | 209 | 286 | 371 | 155 | 267 | 273 | 288.7832536 | 232.0392825 | 0.803506712 | 0.402212926 | unchanged |
| hsa_circ_000487 | 141 | 177 | 232 | 115 | 183 | 189 | 183.2631322 | 162.230758 | 0.885234013 | 0.585595784 | unchanged |
| hsa_circ_000508 | 332 | 416 | 893 | 341 | 520 | 536 | 546.9441407 | 465.4498019 | 0.851000618 | 0.683522645 | unchanged |
| hsa_circ_000519 | 61 | 114 | 57 | 60 | 50 | 58 | 77.50337998 | 56.01321103 | 0.72271959 | 0.307109372 | unchanged |
| hsa_circ_000525 | 109 | 61 | 66 | 116 | 76 | 76 | 78.7375038 | 89.22912622 | 1.133248095 | 0.63168015 | unchanged |
| hsa_circ_000526 | 459 | 275 | 228 | 526 | 268 | 262 | 320.5640324 | 352.0323685 | 1.098165524 | 0.792801361 | unchanged |
| hsa_circ_000543 | 271 | 422 | 472 | 167 | 298 | 301 | 388.2281342 | 255.4463943 | 0.65798012 | 0.150959053 | unchanged |
| hsa_circ_000552 | 189 | 398 | 332 | 225 | 280 | 290 | 306.4608104 | 265.0655957 | 0.864924932 | 0.559479688 | unchanged |
| hsa_circ_000554 | 770 | 542 | 501 | 660 | 431 | 410 | 604.527579 | 500.1258437 | 0.827300294 | 0.417963405 | unchanged |
| hsa_circ_000556 | 93 | 114 | 129 | 114 | 132 | 126 | 111.8663153 | 123.971642 | 1.108212437 | 0.347554012 | unchanged |
| hsa_circ_000569 | 73 | 65 | 78 | 65 | 81 | 93 | 72.35047305 | 79.75066978 | 1.102282631 | 0.458498747 | unchanged |
| hsa_circ_000571 | 81 | 191 | 115 | 84 | 115 | 121 | 129.2398113 | 106.8170475 | 0.826502657 | 0.550837268 | unchanged |
| hsa_circ_000578 | 110 | 200 | 105 | 121 | 93 | 105 | 138.4353027 | 106.2012304 | 0.767154247 | 0.370808854 | unchanged |
| hsa_circ_000581 | 98 | 89 | 103 | 92 | 119 | 110 | 96.75835204 | 107.0781049 | 1.106654905 | 0.321919665 | unchanged |
| hsa_circ_000585 | 1213 | 1768 | 2995 | 1371 | 2863 | 2804 | 1991.971547 | 2346.112543 | 1.177784164 | 0.647640264 | unchanged |
| hsa_circ_000593 | 343 | 243 | 212 | 307 | 152 | 158 | 265.9601189 | 205.5414679 | 0.772828155 | 0.398927312 | unchanged |
| hsa_circ_000598 | 1978 | 2607 | 4837 | 2208 | 3896 | 3885 | 3140.901641 | 3329.777469 | 1.06013427 | 0.86381995 | unchanged |
| hsa_circ_000604 | 66 | 61 | 61 | 64 | 61 | 61 | 62.53480613 | 61.92103504 | 0.990185129 | 0.77364444 | unchanged |
| hsa_circ_000617 | 266 | 145 | 94 | 253 | 95 | 92 | 168.1538686 | 146.6599681 | 0.872177187 | 0.784406062 | unchanged |
| hsa_circ_000618 | 347 | 275 | 147 | 256 | 114 | 106 | 256.3940866 | 159.0314883 | 0.62026192 | 0.269407737 | unchanged |
| hsa_circ_000620 | 126 | 99 | 99 | 112 | 84 | 86 | 107.9336008 | 94.03267382 | 0.871208531 | 0.344864608 | unchanged |
| hsa_circ_000621 | 298 | 363 | 411 | 320 | 522 | 523 | 357.4250942 | 454.8239657 | 1.272501492 | 0.263977499 | unchanged |
| hsa_circ_000623 | 166 | 272 | 247 | 173 | 270 | 282 | 228.2470738 | 241.4649289 | 1.057910294 | 0.792738697 | unchanged |
| hsa_circ_000624 | 1655 | 9330 | 10570 | 1806 | 12295 | 12050 | 7184.848318 | 8717.244931 | 1.213281693 | 0.747419388 | unchanged |
| hsa_circ_000626 | 131 | 207 | 112 | 142 | 84 | 89 | 149.9711452 | 105.0522982 | 0.700483404 | 0.263471397 | unchanged |
| hsa_circ_000629 | 69243 | 58712 | 75755 | 68488 | 71666 | 71666 | 67903.31419 | 70606.35071 | 1.039807137 | 0.622654826 | unchanged |
| hsa_circ_000638 | 2461 | 1622 | 1264 | 2214 | 2001 | 1961 | 1782.317444 | 2058.766389 | 1.155106458 | 0.489006234 | unchanged |
| hsa_circ_000639 | 321 | 371 | 206 | 311 | 169 | 156 | 299.1658391 | 211.7433119 | 0.707779045 | 0.277333619 | unchanged |
| hsa_circ_000644 | 416 | 369 | 431 | 418 | 273 | 269 | 405.5155976 | 319.8344919 | 0.788710703 | 0.177904205 | unchanged |
| hsa_circ_000645 | 1299 | 2177 | 2658 | 1482 | 2478 | 2434 | 2044.445759 | 2130.90967 | 1.042292103 | 0.874506291 | unchanged |
| hsa_circ_000651 | 192 | 254 | 137 | 189 | 124 | 122 | 194.4418294 | 145.2421905 | 0.746969883 | 0.289379813 | unchanged |
| hsa_circ_000653 | 456 | 548 | 634 | 452 | 670 | 686 | 545.780816 | 602.5123441 | 1.103945625 | 0.568056273 | unchanged |
| hsa_circ_000662 | 1036 | 304 | 278 | 1034 | 345 | 310 | 539.4476287 | 563.2548294 | 1.044132552 | 0.947937902 | unchanged |
| hsa_circ_000671 | 3438 | 2423 | 2169 | 3372 | 2399 | 2380 | 2676.497197 | 2717.203834 | 1.015208922 | 0.939916652 | unchanged |
| hsa_circ_000676 | 3675 | 2433 | 2395 | 2955 | 2345 | 2349 | 2834.471521 | 2549.706345 | 0.899535002 | 0.574744687 | unchanged |
| hsa_circ_000679 | 431 | 519 | 587 | 639 | 596 | 593 | 512.3069704 | 609.3225813 | 1.189370078 | 0.109901355 | unchanged |
| hsa_circ_000684 | 186 | 281 | 318 | 268 | 310 | 309 | 261.8950621 | 295.5445014 | 1.128484436 | 0.465790761 | unchanged |
| hsa_circ_000689 | 461 | 535 | 848 | 497 | 946 | 957 | 614.4334199 | 800.2833375 | 1.302473647 | 0.388919862 | unchanged |
| hsa_circ_000695 | 137 | 103 | 109 | 292 | 129 | 129 | 116.1988541 | 183.2982661 | 1.577453302 | 0.292020967 | unchanged |
| hsa_circ_000696 | 75 | 59 | 79 | 69 | 79 | 76 | 71.10061408 | 75.10488157 | 1.056318325 | 0.581208289 | unchanged |
| hsa_circ_000708 | 74 | 69 | 62 | 79 | 63 | 64 | 68.36548678 | 68.58872633 | 1.003265384 | 0.972974976 | unchanged |
| hsa_circ_000709 | 69 | 65 | 59 | 66 | 62 | 66 | 64.42772501 | 64.27670113 | 0.997655918 | 0.964777006 | unchanged |
| hsa_circ_000711 | 1581 | 1304 | 981 | 1638 | 1584 | 1582 | 1288.560366 | 1601.363523 | 1.24275398 | 0.147223449 | unchanged |
| hsa_circ_000721 | 459 | 666 | 572 | 494 | 673 | 702 | 565.7393799 | 623.3587522 | 1.101847908 | 0.549933175 | unchanged |
| hsa_circ_000724 | 66 | 63 | 60 | 56 | 63 | 68 | 62.87497712 | 62.4113034 | 0.992625465 | 0.90662797 | unchanged |
| hsa_circ_000749 | 92 | 88 | 123 | 91 | 144 | 120 | 100.8122264 | 118.4170328 | 1.174629676 | 0.402578746 | unchanged |
| hsa_circ_000754 | 196 | 314 | 290 | 190 | 261 | 259 | 266.677479 | 236.5971366 | 0.88720329 | 0.523263161 | unchanged |
| hsa_circ_000764 | 1619 | 4074 | 6480 | 2049 | 5324 | 5678 | 4057.714871 | 4350.330703 | 1.072113453 | 0.879895672 | unchanged |
| hsa_circ_000775 | 97 | 72 | 73 | 151 | 90 | 86 | 80.73007751 | 109.2208148 | 1.352913538 | 0.277073645 | unchanged |
| hsa_circ_000776 | 181 | 300 | 248 | 178 | 254 | 243 | 242.950616 | 224.9066845 | 0.925730044 | 0.687740268 | unchanged |
| hsa_circ_000780 | 3621 | 2639 | 3129 | 2742 | 2415 | 2450 | 3129.555115 | 2535.725143 | 0.810250994 | 0.120557146 | unchanged |
| hsa_circ_000781 | 137 | 97 | 83 | 121 | 76 | 76 | 105.7039962 | 91.16427972 | 0.862448753 | 0.546991802 | unchanged |
| hsa_circ_000782 | 68 | 82 | 194 | 73 | 98 | 102 | 114.5907961 | 90.88730937 | 0.793146679 | 0.592042984 | unchanged |
| hsa_circ_000792 | 1015 | 1444 | 1562 | 1310 | 1731 | 1656 | 1340.381448 | 1565.640863 | 1.168056202 | 0.345393781 | unchanged |
| hsa_circ_000798 | 97 | 87 | 123 | 102 | 154 | 151 | 102.2052433 | 135.6366368 | 1.327100571 | 0.173140523 | unchanged |
| hsa_circ_000799 | 99 | 95 | 81 | 90 | 72 | 79 | 91.6177067 | 80.45557429 | 0.87816621 | 0.220532686 | unchanged |
| hsa_circ_000803 | 91 | 73 | 99 | 103 | 111 | 101 | 87.63049876 | 104.9159319 | 1.19725362 | 0.111083795 | unchanged |
| hsa_circ_000807 | 91 | 83 | 66 | 85 | 61 | 58 | 80.10578652 | 68.2380863 | 0.851849651 | 0.348453114 | unchanged |
| hsa_circ_000811 | 76 | 120 | 86 | 67 | 91 | 90 | 94.08054469 | 82.30037563 | 0.874786343 | 0.491293965 | unchanged |
| hsa_circ_000815 | 328 | 204 | 190 | 294 | 253 | 259 | 240.5964022 | 268.5131169 | 1.116031306 | 0.573713513 | unchanged |
| hsa_circ_000818 | 167 | 258 | 173 | 222 | 182 | 187 | 199.2774383 | 196.8858669 | 0.987998785 | 0.944015552 | unchanged |
| hsa_circ_000820 | 99 | 69 | 92 | 102 | 102 | 116 | 86.47650317 | 106.7305494 | 1.234214445 | 0.12219817 | unchanged |
| hsa_circ_000832 | 64 | 76 | 60 | 67 | 68 | 66 | 66.87661373 | 67.08566285 | 1.003125893 | 0.96848734 | unchanged |
| hsa_circ_000853 | 70 | 46 | 66 | 71 | 77 | 74 | 60.48315083 | 74.23987177 | 1.227447161 | 0.1416518 | unchanged |
| hsa_circ_000855 | 367 | 417 | 375 | 413 | 485 | 489 | 386.0337066 | 462.3095899 | 1.197588661 | 0.059516013 | unchanged |
| hsa_circ_000863 | 111 | 146 | 119 | 112 | 121 | 133 | 125.2476347 | 121.9473685 | 0.973650072 | 0.799142942 | unchanged |
| hsa_circ_000864 | 6715 | 2813 | 2936 | 4246 | 1806 | 1722 | 4154.688554 | 2591.488303 | 0.623750317 | 0.363232176 | unchanged |
| hsa_circ_000868 | 351 | 574 | 559 | 305 | 516 | 584 | 494.7622538 | 468.1515293 | 0.946215128 | 0.821761489 | unchanged |
| hsa_circ_000872 | 3589 | 4791 | 5180 | 4043 | 4938 | 5530 | 4519.797915 | 4837.096287 | 1.070201894 | 0.648633051 | unchanged |
| hsa_circ_000888 | 70 | 54 | 58 | 74 | 63 | 60 | 60.7763174 | 65.5311571 | 1.078235074 | 0.502725654 | unchanged |
| hsa_circ_000896 | 371 | 591 | 417 | 396 | 354 | 388 | 459.5803954 | 379.5970109 | 0.825964325 | 0.305426455 | unchanged |
| hsa_circ_000903 | 249 | 261 | 186 | 254 | 194 | 196 | 232.1379333 | 214.6873547 | 0.924826682 | 0.595566504 | unchanged |
| hsa_circ_000905 | 68 | 97 | 78 | 72 | 66 | 67 | 80.80663122 | 68.73533656 | 0.850615049 | 0.2433139 | unchanged |
| hsa_circ_000911 | 11733 | 10400 | 15915 | 9669 | 8871 | 8849 | 12682.64875 | 9129.517225 | 0.719843103 | 0.102345461 | unchanged |
| hsa_circ_000921 | 224 | 387 | 439 | 233 | 188 | 182 | 349.9989227 | 201.2265112 | 0.574934659 | 0.090379262 | unchanged |
| hsa_circ_000923 | 146 | 132 | 258 | 117 | 114 | 122 | 178.5156439 | 117.6972915 | 0.659310797 | 0.200919125 | unchanged |
| hsa_circ_000926 | 968 | 657 | 567 | 792 | 409 | 445 | 730.4259876 | 548.7999159 | 0.751342265 | 0.351021452 | unchanged |
| hsa_circ_000931 | 503 | 369 | 548 | 691 | 355 | 432 | 473.5414013 | 492.9402214 | 1.040965415 | 0.874084897 | unchanged |
| hsa_circ_000936 | 109 | 285 | 154 | 115 | 128 | 138 | 182.4837117 | 127.0904203 | 0.696448023 | 0.357438245 | unchanged |
| hsa_circ_000941 | 247 | 200 | 261 | 246 | 346 | 350 | 235.8850512 | 314.0964674 | 1.331565802 | 0.111642599 | unchanged |
| hsa_circ_000942 | 889 | 542 | 547 | 770 | 344 | 363 | 659.0197881 | 492.6824818 | 0.74759892 | 0.408451307 | unchanged |
| hsa_circ_000943 | 317 | 427 | 332 | 396 | 383 | 538 | 358.656532 | 439.2799347 | 1.224792791 | 0.253089487 | unchanged |
| hsa_circ_000945 | 164 | 202 | 211 | 197 | 263 | 271 | 192.4713568 | 243.7134623 | 1.26623237 | 0.134630641 | unchanged |
| hsa_circ_000956 | 3857 | 4874 | 8580 | 7535 | 6059 | 6052 | 5770.067535 | 6548.626198 | 1.134930598 | 0.634972914 | unchanged |
| hsa_circ_000959 | 62 | 81 | 51 | 54 | 50 | 51 | 64.69646778 | 51.55610661 | 0.796892139 | 0.214489097 | unchanged |
| hsa_circ_000962 | 188 | 220 | 233 | 203 | 256 | 274 | 213.6190256 | 244.1206717 | 1.142785251 | 0.291489228 | unchanged |
| hsa_circ_000963 | 3295 | 1077 | 754 | 2008 | 617 | 586 | 1708.61052 | 1070.168895 | 0.626338702 | 0.528502535 | unchanged |
| hsa_circ_000964 | 59 | 102 | 50 | 62 | 54 | 52 | 70.27840872 | 55.90806444 | 0.795522629 | 0.433411653 | unchanged |
| hsa_circ_000987 | 2745 | 3360 | 7232 | 2942 | 5041 | 5509 | 4445.289284 | 4497.284823 | 1.011696773 | 0.975799975 | unchanged |
| hsa_circ_000996 | 917 | 958 | 1310 | 1151 | 1685 | 1717 | 1061.481097 | 1517.632797 | 1.429731346 | 0.108853001 | unchanged |
| hsa_circ_000997 | 240 | 213 | 191 | 231 | 169 | 171 | 214.6382076 | 190.4369911 | 0.887246466 | 0.386866222 | unchanged |
| hsa_circ_001012 | 803 | 1224 | 1355 | 1039 | 1501 | 1536 | 1127.36429 | 1358.531815 | 1.20505131 | 0.373483507 | unchanged |
| hsa_circ_001023 | 76 | 63 | 67 | 78 | 66 | 63 | 68.55132075 | 69.23926197 | 1.010035419 | 0.913387589 | unchanged |
| hsa_circ_001026 | 133 | 121 | 138 | 132 | 100 | 108 | 130.7495539 | 113.2896905 | 0.866463304 | 0.185514164 | unchanged |
| hsa_circ_001030 | 598 | 550 | 546 | 596 | 489 | 561 | 564.9325277 | 548.6879701 | 0.971245137 | 0.673815719 | unchanged |
| hsa_circ_001038 | 5131 | 10109 | 12037 | 5793 | 9126 | 10559 | 9092.503548 | 8492.967924 | 0.934062646 | 0.821936233 | unchanged |
| hsa_circ_001039 | 213 | 146 | 205 | 181 | 199 | 199 | 188.0837218 | 193.028273 | 1.026289097 | 0.834357712 | unchanged |
| hsa_circ_001040 | 917 | 536 | 442 | 545 | 330 | 325 | 631.7707034 | 400.0558641 | 0.633229528 | 0.226935731 | unchanged |
| hsa_circ_001046 | 3556 | 3100 | 2637 | 3869 | 3705 | 3431 | 3097.884673 | 3668.316857 | 1.18413603 | 0.124838257 | unchanged |
| hsa_circ_001049 | 693 | 966 | 957 | 764 | 1165 | 1153 | 872.2966621 | 1027.377677 | 1.177784717 | 0.385001376 | unchanged |
| hsa_circ_001054 | 64 | 83 | 74 | 61 | 64 | 65 | 73.62289539 | 63.42308071 | 0.861458659 | 0.147425433 | unchanged |
| hsa_circ_001055 | 64 | 59 | 58 | 63 | 59 | 57 | 60.44637452 | 59.6979273 | 0.987617996 | 0.780956215 | unchanged |
| hsa_circ_001058 | 78 | 154 | 271 | 67 | 60 | 60 | 167.6260095 | 62.40377687 | 0.372279798 | 0.134812221 | unchanged |
| hsa_circ_001059 | 508 | 958 | 718 | 659 | 693 | 735 | 727.8650596 | 695.7416189 | 0.955866214 | 0.819489395 | unchanged |
| hsa_circ_001064 | 116 | 140 | 99 | 109 | 88 | 83 | 118.2585718 | 93.31989281 | 0.789117367 | 0.158546314 | unchanged |
| hsa_circ_001066 | 60 | 48 | 64 | 64 | 65 | 60 | 57.48297591 | 63.04467211 | 1.096753797 | 0.354696789 | unchanged |
| hsa_circ_001067 | 35721 | 26764 | 22899 | 35361 | 27311 | 27869 | 28461.59161 | 30180.34732 | 1.060388601 | 0.72760641 | unchanged |
| hsa_circ_001072 | 106 | 174 | 154 | 93 | 149 | 130 | 144.7604074 | 123.9751909 | 0.856416427 | 0.468487978 | unchanged |
| hsa_circ_001073 | 479 | 543 | 343 | 461 | 353 | 366 | 454.9913136 | 393.2912235 | 0.864392818 | 0.416681881 | unchanged |
| hsa_circ_001082 | 253 | 309 | 266 | 310 | 209 | 209 | 276.1642859 | 242.3231747 | 0.877460219 | 0.418072771 | unchanged |
| hsa_circ_001090 | 75 | 64 | 156 | 68 | 76 | 81 | 98.22988 | 75.07477965 | 0.764276406 | 0.472202441 | unchanged |
| hsa_circ_001096 | 1446 | 2792 | 2990 | 1525 | 2243 | 2477 | 2409.197269 | 2081.538207 | 0.863996582 | 0.591970441 | unchanged |
| hsa_circ_001100 | 5808 | 7832 | 6100 | 5795 | 7129 | 7229 | 6579.799085 | 6717.677918 | 1.02095487 | 0.868720814 | unchanged |
| hsa_circ_001108 | 9072 | 5843 | 5957 | 7093 | 5334 | 4931 | 6957.075075 | 5785.949632 | 0.831664107 | 0.401436305 | unchanged |
| hsa_circ_001109 | 1730 | 1596 | 1311 | 1568 | 1447 | 1397 | 1545.621974 | 1470.658197 | 0.951499281 | 0.604841731 | unchanged |
| hsa_circ_001123 | 1288 | 1086 | 712 | 1014 | 888 | 766 | 1028.548523 | 889.2074627 | 0.864526508 | 0.48950934 | unchanged |
| hsa_circ_001129 | 65 | 148 | 102 | 66 | 97 | 82 | 104.7134373 | 81.76507052 | 0.780846018 | 0.418861182 | unchanged |
| hsa_circ_001131 | 177 | 319 | 182 | 187 | 147 | 160 | 226.0177738 | 164.5376321 | 0.727985367 | 0.269315768 | unchanged |
| hsa_circ_001143 | 272 | 111 | 170 | 194 | 168 | 166 | 184.5051449 | 175.8807626 | 0.953256684 | 0.865982336 | unchanged |
| hsa_circ_001153 | 1076 | 1396 | 1348 | 1169 | 1648 | 1389 | 1273.223285 | 1401.850785 | 1.101025092 | 0.492497501 | unchanged |
| hsa_circ_001157 | 75 | 134 | 81 | 73 | 80 | 87 | 96.66510896 | 79.99744146 | 0.827573075 | 0.439089847 | unchanged |
| hsa_circ_001172 | 109 | 84 | 106 | 133 | 124 | 117 | 99.59400653 | 124.5749815 | 1.250828095 | 0.047352277 | unchanged |
| hsa_circ_001175 | 6494 | 3339 | 2014 | 5613 | 2399 | 2394 | 3949.118919 | 3468.563994 | 0.878313382 | 0.792303606 | unchanged |
| hsa_circ_001181 | 74 | 53 | 76 | 72 | 76 | 73 | 67.50956092 | 73.81092868 | 1.093340375 | 0.449136848 | unchanged |
| hsa_circ_001195 | 67 | 68 | 63 | 64 | 58 | 56 | 65.73398618 | 59.16366023 | 0.900046744 | 0.094221041 | unchanged |
| hsa_circ_001196 | 201 | 181 | 177 | 244 | 171 | 171 | 186.1797982 | 195.4797885 | 1.049951662 | 0.732930933 | unchanged |
| hsa_circ_001200 | 94 | 71 | 91 | 99 | 99 | 93 | 85.2788576 | 97.24934212 | 1.140368725 | 0.184020514 | unchanged |
| hsa_circ_001201 | 159 | 157 | 410 | 160 | 230 | 234 | 241.9211263 | 207.6486578 | 0.858332056 | 0.715530747 | unchanged |
| hsa_circ_001205 | 1428 | 2683 | 2274 | 1631 | 2428 | 2410 | 2128.287134 | 2156.531725 | 1.013271043 | 0.953324357 | unchanged |
| hsa_circ_001210 | 132 | 100 | 109 | 145 | 110 | 122 | 114.0898853 | 125.8492542 | 1.10307109 | 0.448578607 | unchanged |
| hsa_circ_001216 | 1937 | 2248 | 2081 | 2123 | 2671 | 2652 | 2088.633452 | 2481.953583 | 1.18831458 | 0.121778597 | unchanged |
| hsa_circ_001225 | 6621 | 3182 | 3363 | 3918 | 2213 | 2214 | 4388.616673 | 2781.705904 | 0.633845722 | 0.269189742 | unchanged |
| hsa_circ_001233 | 1993 | 2413 | 2351 | 1945 | 2795 | 2666 | 2252.403098 | 2468.819522 | 1.096082457 | 0.504078274 | unchanged |
| hsa_circ_001240 | 1123 | 517 | 482 | 685 | 438 | 464 | 707.5132311 | 529.1739097 | 0.747935002 | 0.467637242 | unchanged |
| hsa_circ_001241 | 1664 | 1029 | 1433 | 1083 | 918 | 950 | 1375.376677 | 983.9343591 | 0.715392645 | 0.111553021 | unchanged |
| hsa_circ_001250 | 120 | 64 | 61 | 108 | 64 | 65 | 81.61812449 | 79.05362779 | 0.968579323 | 0.920848768 | unchanged |
| hsa_circ_001255 | 153 | 229 | 157 | 168 | 133 | 140 | 179.7806334 | 146.7247134 | 0.816131919 | 0.282778285 | unchanged |
| hsa_circ_001264 | 1024 | 708 | 863 | 722 | 809 | 810 | 864.9483299 | 780.1046119 | 0.901908918 | 0.425326002 | unchanged |
| hsa_circ_001275 | 73 | 122 | 60 | 65 | 57 | 59 | 84.90210433 | 60.395498 | 0.711354547 | 0.272506265 | unchanged |
| hsa_circ_001278 | 243 | 217 | 148 | 228 | 118 | 118 | 202.5812583 | 154.3984524 | 0.762155659 | 0.35759431 | unchanged |
| hsa_circ_001282 | 60 | 57 | 63 | 56 | 81 | 61 | 59.90067269 | 65.93330506 | 1.100710595 | 0.488685824 | unchanged |
| hsa_circ_001288 | 316 | 223 | 252 | 327 | 211 | 230 | 263.8797637 | 255.9552429 | 0.969969198 | 0.870012632 | unchanged |
| hsa_circ_001296 | 310 | 304 | 336 | 423 | 368 | 346 | 316.4462616 | 379.0371141 | 1.197792991 | 0.067215461 | unchanged |
| hsa_circ_001300 | 138 | 141 | 148 | 134 | 89 | 90 | 142.3698505 | 104.174869 | 0.731720015 | 0.066215772 | unchanged |
| hsa_circ_001302 | 6970 | 7727 | 8139 | 7588 | 8234 | 8554 | 7612.025935 | 8125.598019 | 1.067468515 | 0.312441474 | unchanged |
| hsa_circ_001304 | 100 | 66 | 60 | 107 | 71 | 70 | 75.41007065 | 82.70152213 | 1.096690686 | 0.700613931 | unchanged |
| hsa_circ_001309 | 380 | 230 | 134 | 297 | 108 | 108 | 247.9957458 | 171.2681998 | 0.690609427 | 0.466556791 | unchanged |
| hsa_circ_001340 | 119 | 61 | 98 | 117 | 135 | 127 | 93.00897538 | 126.1381155 | 1.356192937 | 0.136292326 | unchanged |
| hsa_circ_001343 | 54 | 103 | 51 | 54 | 52 | 47 | 69.26521891 | 50.92107401 | 0.735160804 | 0.344169957 | unchanged |
| hsa_circ_001350 | 4591 | 1305 | 584 | 2434 | 467 | 422 | 2159.712613 | 1107.799308 | 0.512938296 | 0.494269795 | unchanged |
| hsa_circ_001355 | 75 | 105 | 85 | 71 | 81 | 81 | 88.5026174 | 77.62209754 | 0.877059909 | 0.304495701 | unchanged |
| hsa_circ_001359 | 153 | 241 | 127 | 165 | 107 | 109 | 173.7312102 | 126.8422429 | 0.730106253 | 0.298692118 | unchanged |
| hsa_circ_001363 | 1039 | 643 | 669 | 1343 | 795 | 811 | 783.7323001 | 983.0952253 | 1.254376303 | 0.418013184 | unchanged |
| hsa_circ_001369 | 379 | 159 | 203 | 243 | 140 | 142 | 247.3866028 | 174.9263543 | 0.70709712 | 0.390394957 | unchanged |
| hsa_circ_001372 | 82 | 70 | 71 | 82 | 75 | 79 | 74.54241217 | 78.71920035 | 1.056032372 | 0.395186549 | unchanged |
| hsa_circ_001379 | 1672 | 2980 | 2303 | 2089 | 2350 | 2392 | 2318.141699 | 2277.114994 | 0.982301899 | 0.921136247 | unchanged |
| hsa_circ_001380 | 275 | 286 | 338 | 301 | 285 | 295 | 299.7625574 | 293.7688292 | 0.980005081 | 0.781000125 | unchanged |
| hsa_circ_001389 | 2204 | 965 | 827 | 1246 | 581 | 572 | 1331.697295 | 800.0507874 | 0.600775259 | 0.340116391 | unchanged |
| hsa_circ_001393 | 123 | 107 | 156 | 139 | 194 | 182 | 129.0824847 | 171.8619376 | 1.331411756 | 0.123858855 | unchanged |
| hsa_circ_001396 | 1242 | 1047 | 864 | 1270 | 1007 | 995 | 1050.872191 | 1090.864691 | 1.038056484 | 0.791131316 | unchanged |
| hsa_circ_001401 | 667 | 352 | 341 | 500 | 441 | 442 | 453.5027878 | 460.7571312 | 1.015996249 | 0.94992258 | unchanged |
| hsa_circ_001405 | 3623 | 2421 | 2628 | 2421 | 1396 | 1405 | 2890.621835 | 1740.766599 | 0.602211807 | 0.084395792 | unchanged |
| hsa_circ_001408 | 595 | 601 | 583 | 608 | 750 | 732 | 592.9017778 | 696.611283 | 1.174918526 | 0.081290431 | unchanged |
| hsa_circ_001409 | 3313 | 1810 | 1549 | 3666 | 1505 | 1574 | 2224.141711 | 2248.343952 | 1.01088161 | 0.979772783 | unchanged |
| hsa_circ_001416 | 1948 | 1933 | 1581 | 1785 | 1540 | 1591 | 1820.578795 | 1638.415366 | 0.899942024 | 0.266353543 | unchanged |
| hsa_circ_001420 | 64 | 94 | 58 | 62 | 71 | 67 | 71.88027198 | 66.47408593 | 0.924789015 | 0.660638053 | unchanged |
| hsa_circ_001426 | 213 | 233 | 233 | 377 | 253 | 254 | 226.4461045 | 294.4530859 | 1.300323035 | 0.177999504 | unchanged |
| hsa_circ_001430 | 293 | 346 | 254 | 280 | 307 | 295 | 297.7397434 | 293.8553487 | 0.986953725 | 0.896087392 | unchanged |
| hsa_circ_001445 | 90 | 108 | 82 | 96 | 86 | 90 | 93.20254917 | 90.85051893 | 0.974764314 | 0.792702414 | unchanged |
| hsa_circ_001449 | 175 | 133 | 175 | 193 | 202 | 205 | 161.0315597 | 200.1642659 | 1.243012651 | 0.051392661 | unchanged |
| hsa_circ_001459 | 799 | 239 | 283 | 508 | 238 | 247 | 440.3784314 | 330.7458145 | 0.751049077 | 0.613479849 | unchanged |
| hsa_circ_001465 | 961 | 1602 | 1250 | 1181 | 1569 | 1558 | 1271.257649 | 1436.088319 | 1.129659531 | 0.504565526 | unchanged |
| hsa_circ_001468 | 97 | 59 | 82 | 92 | 88 | 90 | 79.33365535 | 90.01270582 | 1.134609333 | 0.390126716 | unchanged |
| hsa_circ_001486 | 421 | 452 | 406 | 419 | 373 | 362 | 426.1439939 | 384.673158 | 0.902683514 | 0.133469292 | unchanged |
| hsa_circ_001493 | 461 | 250 | 226 | 455 | 298 | 238 | 312.3681262 | 330.4069124 | 1.057748485 | 0.863908455 | unchanged |
| hsa_circ_001498 | 1266 | 2324 | 5548 | 1490 | 4221 | 4294 | 3046.038157 | 3335.398356 | 1.094995592 | 0.863958621 | unchanged |
| hsa_circ_001503 | 847 | 605 | 563 | 781 | 467 | 471 | 671.8128017 | 572.9524397 | 0.852845373 | 0.509240978 | unchanged |
| hsa_circ_001527 | 62 | 64 | 138 | 59 | 58 | 56 | 87.94635293 | 57.64204291 | 0.655422777 | 0.292665871 | unchanged |
| hsa_circ_001535 | 74 | 67 | 91 | 76 | 107 | 107 | 77.3979572 | 96.33840253 | 1.244715055 | 0.20477066 | unchanged |
| hsa_circ_001543 | 129 | 100 | 60 | 109 | 62 | 57 | 96.36224172 | 76.27564766 | 0.791551196 | 0.481066959 | unchanged |
| hsa_circ_001547 | 494 | 520 | 663 | 498 | 339 | 368 | 558.9208355 | 401.736927 | 0.718772501 | 0.093717141 | unchanged |
| hsa_circ_001549 | 90 | 119 | 95 | 77 | 88 | 98 | 101.4319615 | 87.39353422 | 0.861597597 | 0.26447733 | unchanged |
| hsa_circ_001551 | 66 | 59 | 65 | 68 | 72 | 75 | 63.39950012 | 71.69861553 | 1.130901906 | 0.050850155 | unchanged |
| hsa_circ_001578 | 308 | 321 | 285 | 355 | 303 | 337 | 304.9090676 | 331.673178 | 1.087777352 | 0.226761428 | unchanged |
| hsa_circ_001579 | 694 | 1009 | 959 | 712 | 971 | 1008 | 887.3651556 | 897.0228348 | 1.010883546 | 0.946372769 | unchanged |
| hsa_circ_001583 | 368 | 413 | 506 | 448 | 454 | 470 | 428.9046221 | 457.196333 | 1.065962709 | 0.528238661 | unchanged |
| hsa_circ_001587 | 734 | 299 | 1029 | 656 | 648 | 614 | 687.3407489 | 639.4163606 | 0.930275648 | 0.832753332 | unchanged |
| hsa_circ_001594 | 6157 | 6304 | 6107 | 5863 | 6089 | 6300 | 6189.387084 | 6083.926769 | 0.982961105 | 0.491459234 | unchanged |
| hsa_circ_001609 | 72 | 77 | 74 | 72 | 72 | 71 | 74.3165735 | 71.53381406 | 0.962555332 | 0.101890488 | unchanged |
| hsa_circ_001612 | 58 | 95 | 53 | 61 | 55 | 54 | 68.8632372 | 56.57597124 | 0.821570021 | 0.412307624 | unchanged |
| hsa_circ_001617 | 71 | 73 | 805 | 67 | 57 | 55 | 316.123715 | 59.61383863 | 0.188577559 | 0.352835225 | unchanged |
| hsa_circ_001622 | 130 | 66 | 49 | 153 | 44 | 42 | 82.01030611 | 79.65081869 | 0.971229379 | 0.959840265 | unchanged |
| hsa_circ_001653 | 30797 | 14467 | 14896 | 22402 | 10845 | 11524 | 20053.6146 | 14923.48757 | 0.744179434 | 0.477221687 | unchanged |
| hsa_circ_001654 | 6038 | 3547 | 5404 | 4058 | 3112 | 3044 | 4996.423995 | 3404.265525 | 0.6813404 | 0.122789886 | unchanged |
| hsa_circ_001658 | 72 | 103 | 71 | 77 | 63 | 61 | 82.01623939 | 66.88029937 | 0.815451914 | 0.272947728 | unchanged |
| hsa_circ_001671 | 735 | 1000 | 958 | 827 | 1023 | 1005 | 897.6817345 | 951.5738629 | 1.060034783 | 0.629719768 | unchanged |
| hsa_circ_001676 | 1413 | 1872 | 1797 | 1616 | 1425 | 1432 | 1693.74758 | 1491.147881 | 0.880383771 | 0.261927902 | unchanged |
| hsa_circ_001678 | 297 | 364 | 332 | 334 | 333 | 348 | 331.1141956 | 338.2240796 | 1.021472604 | 0.740881999 | unchanged |
| hsa_circ_001679 | 214 | 303 | 240 | 218 | 246 | 227 | 252.0801635 | 230.3239267 | 0.913693182 | 0.475778769 | unchanged |
| hsa_circ_001689 | 77 | 210 | 85 | 82 | 81 | 83 | 123.9535902 | 82.18607055 | 0.663039049 | 0.389379933 | unchanged |
| hsa_circ_001695 | 64 | 129 | 67 | 59 | 64 | 64 | 86.48479201 | 62.32894075 | 0.720692497 | 0.317505459 | unchanged |
| hsa_circ_001701 | 163 | 173 | 209 | 184 | 204 | 210 | 181.7069778 | 199.1164306 | 1.09581059 | 0.342787681 | unchanged |
| hsa_circ_001702 | 88 | 90 | 89 | 84 | 88 | 90 | 89.12498159 | 87.09897647 | 0.97726782 | 0.320123215 | unchanged |
| hsa_circ_001724 | 1504 | 778 | 577 | 1194 | 595 | 601 | 952.8271136 | 796.7145258 | 0.836158538 | 0.674025441 | unchanged |
| hsa_circ_001729 | 68 | 85 | 75 | 70 | 69 | 74 | 76.26798683 | 71.01802195 | 0.931164239 | 0.363609987 | unchanged |
| hsa_circ_001741 | 118 | 80 | 117 | 109 | 132 | 128 | 104.7402311 | 123.1186385 | 1.175466554 | 0.26761253 | unchanged |
| hsa_circ_001745 | 77 | 70 | 56 | 81 | 68 | 68 | 67.5534968 | 72.40137703 | 1.071763572 | 0.562714153 | unchanged |
| hsa_circ_001747 | 63 | 189 | 62 | 97 | 58 | 56 | 104.510712 | 70.30593157 | 0.672715076 | 0.483358126 | unchanged |
| hsa_circ_001754 | 284 | 446 | 258 | 339 | 258 | 305 | 329.359348 | 300.5904093 | 0.912651823 | 0.672811038 | unchanged |
| hsa_circ_001769 | 254 | 129 | 116 | 238 | 124 | 108 | 166.2937709 | 156.5084802 | 0.941156601 | 0.878284683 | unchanged |
| hsa_circ_001772 | 93 | 142 | 102 | 94 | 86 | 87 | 112.0547605 | 89.18514769 | 0.795906816 | 0.211439534 | unchanged |
| hsa_circ_001783 | 65 | 67 | 80 | 72 | 61 | 64 | 70.35757296 | 65.74895326 | 0.934497176 | 0.465803798 | unchanged |
| hsa_circ_001785 | 118 | 85 | 82 | 100 | 73 | 77 | 94.96641852 | 83.0125279 | 0.874125077 | 0.449660978 | unchanged |
| hsa_circ_001800 | 1081 | 652 | 669 | 1052 | 622 | 625 | 800.5641438 | 766.5319687 | 0.957489758 | 0.873217004 | unchanged |
| hsa_circ_001805 | 101 | 90 | 115 | 111 | 140 | 134 | 101.868219 | 128.4224105 | 1.260671991 | 0.079716701 | unchanged |
| hsa_circ_001808 | 170 | 252 | 273 | 221 | 272 | 286 | 231.6996772 | 259.8185044 | 1.12135894 | 0.492716995 | unchanged |
| hsa_circ_001818 | 105 | 89 | 82 | 98 | 86 | 87 | 92.10341537 | 90.45094432 | 0.982058526 | 0.837404622 | unchanged |
| hsa_circ_001819 | 86 | 84 | 46 | 90 | 50 | 49 | 72.31742854 | 62.81774549 | 0.868639092 | 0.640335567 | unchanged |
| hsa_circ_001820 | 290 | 362 | 207 | 287 | 337 | 351 | 286.3798718 | 325.0554209 | 1.135049816 | 0.473520311 | unchanged |
| hsa_circ_001826 | 201 | 230 | 210 | 233 | 235 | 239 | 213.6948303 | 235.7100361 | 1.103021705 | 0.065791651 | unchanged |
| hsa_circ_001830 | 514 | 571 | 474 | 541 | 607 | 539 | 520.0266114 | 562.4565896 | 1.081591936 | 0.300506302 | unchanged |
| hsa_circ_001838 | 504 | 288 | 337 | 477 | 460 | 458 | 376.4458902 | 464.9812429 | 1.23518746 | 0.247430197 | unchanged |
| hsa_circ_001844 | 387 | 195 | 154 | 142 | 86 | 90 | 245.4582812 | 105.89319 | 0.431410134 | 0.131401351 | unchanged |
| hsa_circ_001846 | 434 | 693 | 611 | 492 | 656 | 697 | 579.5855604 | 614.9875787 | 1.061081608 | 0.738068228 | unchanged |
| hsa_circ_001857 | 129 | 58 | 59 | 146 | 60 | 54 | 81.93584935 | 86.75118706 | 1.058769608 | 0.904646108 | unchanged |
| hsa_circ_001860 | 76 | 61 | 77 | 72 | 80 | 78 | 71.11999328 | 76.33195882 | 1.073284112 | 0.410144862 | unchanged |
| hsa_circ_001882 | 262 | 215 | 182 | 508 | 174 | 187 | 219.7967286 | 289.7866333 | 1.318430147 | 0.563791555 | unchanged |
| hsa_circ_001886 | 703 | 2867 | 3189 | 1217 | 3299 | 3294 | 2253.13706 | 2603.555893 | 1.155524863 | 0.753960282 | unchanged |
| hsa_circ_001896 | 210 | 161 | 238 | 249 | 340 | 341 | 202.6427174 | 309.7549528 | 1.528576782 | 0.047496337 | unchanged |
| hsa_circ_001899 | 191 | 207 | 115 | 197 | 107 | 108 | 170.9562616 | 137.2569096 | 0.802877346 | 0.457007607 | unchanged |
| hsa_circ_001914 | 190 | 359 | 424 | 232 | 347 | 388 | 324.4850991 | 322.7715585 | 0.994719201 | 0.984697365 | unchanged |
| hsa_circ_001915 | 1778 | 1133 | 905 | 1187 | 772 | 803 | 1271.904106 | 920.9115795 | 0.724041675 | 0.297796629 | unchanged |
| hsa_circ_001937 | 397 | 597 | 559 | 370 | 371 | 365 | 517.3750807 | 369.0071665 | 0.713229493 | 0.072824702 | unchanged |
| hsa_circ_001946 | 87 | 114 | 71 | 87 | 60 | 66 | 90.83238105 | 70.99182508 | 0.781569571 | 0.254958054 | unchanged |
| hsa_circ_001950 | 1390 | 1064 | 1282 | 1960 | 1504 | 1497 | 1245.253112 | 1653.375996 | 1.327742914 | 0.086747351 | unchanged |
| hsa_circ_001952 | 231 | 196 | 305 | 273 | 447 | 442 | 243.8612667 | 387.1446661 | 1.587561122 | 0.094749303 | unchanged |
| hsa_circ_001955 | 421 | 453 | 329 | 371 | 373 | 391 | 401.0247186 | 378.52074 | 0.943883812 | 0.581394933 | unchanged |
| hsa_circ_001959 | 162 | 274 | 165 | 153 | 159 | 159 | 200.396771 | 156.8465869 | 0.782680211 | 0.302776615 | unchanged |
| hsa_circ_001968 | 83 | 77 | 80 | 81 | 100 | 97 | 79.87916056 | 92.40113028 | 1.156761409 | 0.113061743 | unchanged |
| hsa_circ_001978 | 373 | 309 | 313 | 311 | 299 | 298 | 331.6919084 | 302.5433338 | 0.912121539 | 0.237904554 | unchanged |
| hsa_circ_001992 | 86 | 86 | 104 | 89 | 130 | 128 | 92.23419912 | 115.7363499 | 1.254809507 | 0.179122849 | unchanged |
| hsa_circ_001993 | 89 | 165 | 82 | 96 | 73 | 77 | 111.801299 | 82.04892594 | 0.733881687 | 0.338317214 | unchanged |
| hsa_circ_001994 | 75 | 52 | 77 | 88 | 89 | 91 | 67.88445653 | 89.37693171 | 1.316603775 | 0.057306415 | unchanged |
| hsa_circ_002006 | 425 | 438 | 343 | 461 | 402 | 405 | 402.0653353 | 422.5878975 | 1.051042854 | 0.591656432 | unchanged |
| hsa_circ_002008 | 61 | 69 | 51 | 61 | 48 | 45 | 60.22288601 | 51.20009292 | 0.850176674 | 0.281220289 | unchanged |
| hsa_circ_002010 | 97 | 143 | 130 | 104 | 138 | 138 | 123.2117132 | 126.3978815 | 1.025859297 | 0.865626672 | unchanged |
| hsa_circ_002011 | 75 | 56 | 427 | 73 | 93 | 92 | 186.1029152 | 86.11477797 | 0.462726647 | 0.454289175 | unchanged |
| hsa_circ_002026 | 76 | 88 | 62 | 78 | 63 | 67 | 75.37209772 | 69.40365385 | 0.920813616 | 0.532201682 | unchanged |
| hsa_circ_002039 | 380 | 518 | 781 | 514 | 700 | 737 | 559.8312021 | 649.9914599 | 1.161049005 | 0.544641118 | unchanged |
| hsa_circ_002042 | 3097 | 4456 | 3895 | 3064 | 4068 | 3988 | 3816.099167 | 3706.683846 | 0.971327967 | 0.840366151 | unchanged |
| hsa_circ_002044 | 2771 | 1657 | 911 | 2574 | 1516 | 1547 | 1779.572704 | 1878.999585 | 1.055871211 | 0.884549771 | unchanged |
| hsa_circ_002048 | 386 | 592 | 543 | 410 | 651 | 616 | 506.8590392 | 559.1380731 | 1.103143142 | 0.620487353 | unchanged |
| hsa_circ_002050 | 256 | 291 | 295 | 247 | 292 | 343 | 281.0208334 | 294.0622091 | 1.046407149 | 0.690734312 | unchanged |
| hsa_circ_002051 | 111 | 96 | 210 | 111 | 111 | 124 | 138.9515557 | 115.2445058 | 0.829386222 | 0.545723977 | unchanged |
| hsa_circ_002076 | 211 | 116 | 126 | 176 | 177 | 172 | 151.195646 | 174.8830675 | 1.156667352 | 0.476245348 | unchanged |
| hsa_circ_002085 | 104 | 65 | 63 | 118 | 67 | 61 | 77.17439684 | 82.0720006 | 1.06346151 | 0.837604531 | unchanged |
| hsa_circ_002086 | 837 | 938 | 781 | 921 | 1157 | 1270 | 851.8134395 | 1116.1374 | 1.310307338 | 0.078861895 | unchanged |
| hsa_circ_002106 | 418 | 264 | 291 | 370 | 244 | 223 | 324.1501364 | 278.9955559 | 0.860698561 | 0.532529496 | unchanged |
| hsa_circ_002111 | 110 | 129 | 172 | 138 | 187 | 184 | 136.8821311 | 169.7925681 | 1.240429023 | 0.243979846 | unchanged |
| hsa_circ_002115 | 97 | 89 | 99 | 88 | 87 | 91 | 94.91475864 | 88.71111398 | 0.934639831 | 0.135903582 | unchanged |
| hsa_circ_002117 | 1196 | 1619 | 2216 | 1121 | 1256 | 1243 | 1676.611245 | 1206.470367 | 0.719588617 | 0.190881293 | unchanged |
| hsa_circ_002143 | 5533 | 5169 | 3635 | 7267 | 5401 | 4789 | 4779.29254 | 5818.986261 | 1.217541344 | 0.333193793 | unchanged |
| hsa_circ_002144 | 1845 | 3096 | 3517 | 2056 | 4077 | 4092 | 2819.626196 | 3408.403904 | 1.208814101 | 0.523051472 | unchanged |
| hsa_circ_002149 | 362 | 461 | 406 | 380 | 498 | 530 | 409.6693464 | 469.4841754 | 1.146007578 | 0.327629503 | unchanged |
| hsa_circ_002153 | 75 | 63 | 83 | 67 | 88 | 98 | 73.54888713 | 84.57767099 | 1.149951744 | 0.362433413 | unchanged |
| hsa_circ_002172 | 1595 | 3252 | 3605 | 1727 | 3504 | 3557 | 2817.38725 | 2929.273297 | 1.039712697 | 0.903164613 | unchanged |
| hsa_circ_002178 | 1072 | 1689 | 2181 | 1127 | 2390 | 2417 | 1647.374819 | 1977.927898 | 1.200654445 | 0.568726851 | unchanged |
| hsa_circ_100001 | 3116 | 3917 | 5543 | 3476 | 3698 | 4502 | 4192.027344 | 3892.108637 | 0.928454974 | 0.719808057 | unchanged |
| hsa_circ_100002 | 58 | 102 | 50 | 55 | 42 | 48 | 70.03171703 | 48.13551223 | 0.687338741 | 0.259846013 | unchanged |
| hsa_circ_100003 | 92 | 130 | 96 | 92 | 78 | 84 | 105.849514 | 84.73607939 | 0.800533476 | 0.174751695 | unchanged |
| hsa_circ_100004 | 572 | 784 | 899 | 617 | 1003 | 1039 | 751.5415275 | 886.4232102 | 1.179473359 | 0.461612918 | unchanged |
| hsa_circ_100005 | 272 | 192 | 162 | 208 | 141 | 140 | 208.702071 | 162.79538 | 0.780037204 | 0.313260963 | unchanged |
| hsa_circ_100006 | 83 | 145 | 106 | 86 | 77 | 88 | 111.4229957 | 83.33183415 | 0.747887217 | 0.197011408 | unchanged |
| hsa_circ_100008 | 553 | 731 | 582 | 599 | 782 | 837 | 622.3091007 | 739.5645601 | 1.188419966 | 0.265175545 | unchanged |
| hsa_circ_100009 | 2761 | 2824 | 2500 | 2377 | 2552 | 2567 | 2695.077552 | 2498.988692 | 0.927241849 | 0.167235033 | unchanged |
| hsa_circ_100010 | 58 | 94 | 90 | 68 | 83 | 84 | 80.94307467 | 78.22747363 | 0.966450483 | 0.83720937 | unchanged |
| hsa_circ_100011 | 452 | 492 | 435 | 480 | 530 | 462 | 459.7733192 | 490.6435126 | 1.067142203 | 0.306977149 | unchanged |
| hsa_circ_100012 | 94 | 157 | 123 | 100 | 114 | 111 | 124.6842144 | 108.165992 | 0.867519537 | 0.42572861 | unchanged |
| hsa_circ_100013 | 165 | 200 | 205 | 217 | 216 | 218 | 189.8018687 | 217.0239096 | 1.143423461 | 0.096011246 | unchanged |
| hsa_circ_100014 | 138 | 196 | 108 | 158 | 95 | 102 | 147.3482336 | 118.5029232 | 0.804237148 | 0.42507012 | unchanged |
| hsa_circ_100015 | 69 | 120 | 102 | 80 | 93 | 97 | 96.87910374 | 90.04716112 | 0.929479709 | 0.685866644 | unchanged |
| hsa_circ_100017 | 598 | 668 | 590 | 716 | 614 | 577 | 618.7314298 | 635.8551044 | 1.027675456 | 0.74100711 | unchanged |
| hsa_circ_100018 | 12622 | 6970 | 4542 | 18263 | 6820 | 6253 | 8044.627168 | 10445.40611 | 1.298432592 | 0.628296643 | unchanged |
| hsa_circ_100019 | 185 | 188 | 175 | 204 | 169 | 168 | 182.8388064 | 180.5135398 | 0.987282423 | 0.860787242 | unchanged |
| hsa_circ_100020 | 192 | 202 | 211 | 212 | 274 | 276 | 201.5242854 | 254.1367471 | 1.261072563 | 0.073858972 | unchanged |
| hsa_circ_100021 | 1522 | 1253 | 955 | 1238 | 830 | 869 | 1243.447694 | 978.6462943 | 0.787042591 | 0.274427227 | unchanged |
| hsa_circ_100022 | 144 | 180 | 143 | 148 | 161 | 167 | 155.8700442 | 158.7000685 | 1.018156306 | 0.842180088 | unchanged |
| hsa_circ_100023 | 99 | 121 | 112 | 96 | 105 | 101 | 110.4790776 | 101.0112193 | 0.914301798 | 0.235331003 | unchanged |
| hsa_circ_100024 | 1285 | 1848 | 1525 | 971 | 1181 | 1368 | 1552.931134 | 1173.596755 | 0.75573007 | 0.129815992 | unchanged |
| hsa_circ_100025 | 177 | 195 | 164 | 179 | 133 | 143 | 178.6185722 | 151.5175484 | 0.848274323 | 0.180280138 | unchanged |
| hsa_circ_100026 | 74 | 73 | 78 | 70 | 78 | 75 | 75.03848546 | 74.49465304 | 0.99275262 | 0.855560596 | unchanged |
| hsa_circ_100027 | 1014 | 1131 | 1192 | 1083 | 1488 | 1566 | 1112.421503 | 1378.91635 | 1.239562832 | 0.167902663 | unchanged |
| hsa_circ_100028 | 523 | 649 | 503 | 592 | 608 | 623 | 558.2248904 | 607.3841919 | 1.088063614 | 0.349475514 | unchanged |
| hsa_circ_100029 | 142 | 194 | 156 | 138 | 163 | 160 | 164.1436976 | 153.3901534 | 0.934487011 | 0.570182356 | unchanged |
| hsa_circ_100030 | 100 | 318 | 106 | 105 | 106 | 110 | 174.5492347 | 107.0339029 | 0.613201788 | 0.400498747 | unchanged |
| hsa_circ_100031 | 79 | 158 | 126 | 88 | 136 | 124 | 121.2114786 | 115.9034377 | 0.956208431 | 0.855035463 | unchanged |
| hsa_circ_100032 | 250 | 441 | 304 | 331 | 264 | 274 | 331.349568 | 289.5479188 | 0.873844262 | 0.527806724 | unchanged |
| hsa_circ_100033 | 1055 | 945 | 1655 | 1420 | 2130 | 2116 | 1218.325832 | 1888.643861 | 1.550196025 | 0.105750794 | unchanged |
| hsa_circ_100034 | 371 | 659 | 605 | 443 | 661 | 560 | 544.5970645 | 554.8599574 | 1.018844929 | 0.929314977 | unchanged |
| hsa_circ_100035 | 321 | 477 | 415 | 348 | 495 | 437 | 404.3749247 | 426.6405359 | 1.0550618 | 0.738977409 | unchanged |
| hsa_circ_100036 | 128 | 89 | 102 | 143 | 140 | 127 | 106.5005475 | 136.6147749 | 1.282761245 | 0.075365549 | unchanged |
| hsa_circ_100037 | 129 | 203 | 156 | 155 | 186 | 177 | 162.6764442 | 172.475716 | 1.060237804 | 0.697744279 | unchanged |
| hsa_circ_100038 | 127 | 149 | 127 | 128 | 131 | 155 | 134.4111571 | 138.0374616 | 1.026979193 | 0.766218485 | unchanged |
| hsa_circ_100040 | 1090 | 359 | 280 | 566 | 193 | 202 | 576.0543636 | 320.5989922 | 0.556542945 | 0.421633055 | unchanged |
| hsa_circ_100041 | 208 | 154 | 172 | 194 | 136 | 139 | 178.1749357 | 156.3013988 | 0.877235612 | 0.425175977 | unchanged |
| hsa_circ_100042 | 364 | 383 | 384 | 358 | 369 | 364 | 377.1986162 | 363.6509772 | 0.96408354 | 0.146823318 | unchanged |
| hsa_circ_100044 | 264 | 277 | 270 | 315 | 271 | 268 | 270.020458 | 284.6805839 | 1.054292649 | 0.400934869 | unchanged |
| hsa_circ_100045 | 1386 | 2255 | 1926 | 1566 | 2400 | 2498 | 1855.755024 | 2155.004816 | 1.161255008 | 0.485031506 | unchanged |
| hsa_circ_100046 | 133 | 154 | 168 | 172 | 213 | 226 | 151.8460612 | 203.5022579 | 1.340187927 | 0.053072332 | unchanged |
| hsa_circ_100047 | 101 | 107 | 100 | 102 | 85 | 91 | 102.6579204 | 92.91082948 | 0.905052714 | 0.148786622 | unchanged |
| hsa_circ_100048 | 384 | 321 | 366 | 336 | 314 | 325 | 356.9366523 | 325.1204416 | 0.910863145 | 0.180411345 | unchanged |
| hsa_circ_100049 | 86 | 127 | 92 | 90 | 88 | 90 | 101.6686606 | 89.29870996 | 0.878330741 | 0.386769061 | unchanged |
| hsa_circ_100050 | 316 | 243 | 221 | 206 | 174 | 175 | 260.1147811 | 184.9379712 | 0.710986013 | 0.070474669 | unchanged |
| hsa_circ_100051 | 377 | 436 | 435 | 393 | 482 | 531 | 415.8235318 | 468.5988887 | 1.126917677 | 0.302509332 | unchanged |
| hsa_circ_100052 | 139 | 129 | 180 | 162 | 211 | 230 | 149.621841 | 201.0005985 | 1.343390759 | 0.117119653 | unchanged |
| hsa_circ_100053 | 12265 | 22578 | 23873 | 12991 | 23733 | 23853 | 19571.95605 | 20192.29156 | 1.03169512 | 0.909814492 | unchanged |
| hsa_circ_100054 | 56 | 157 | 56 | 55 | 56 | 58 | 89.69975892 | 56.36420498 | 0.628365178 | 0.378863647 | unchanged |
| hsa_circ_100055 | 60 | 67 | 56 | 66 | 54 | 54 | 60.92208335 | 58.07780269 | 0.953312814 | 0.625122992 | unchanged |
| hsa_circ_100056 | 482 | 390 | 352 | 438 | 370 | 362 | 408.0069164 | 389.7955255 | 0.955364995 | 0.71048022 | unchanged |
| hsa_circ_100057 | 63 | 82 | 60 | 58 | 51 | 56 | 68.18842532 | 55.21228395 | 0.809701701 | 0.143744633 | unchanged |
| hsa_circ_100058 | 803 | 930 | 857 | 858 | 1215 | 1182 | 863.7794888 | 1085.3975 | 1.25656781 | 0.137655668 | unchanged |
| hsa_circ_100059 | 68 | 78 | 66 | 74 | 61 | 67 | 71.13280864 | 67.28447488 | 0.945899314 | 0.493231825 | unchanged |
| hsa_circ_100060 | 214 | 225 | 158 | 211 | 148 | 156 | 198.8106129 | 171.6428031 | 0.863348292 | 0.398134982 | unchanged |
| hsa_circ_100061 | 102 | 146 | 98 | 113 | 102 | 105 | 115.4831127 | 106.3728803 | 0.921111995 | 0.5936352 | unchanged |
| hsa_circ_100062 | 70 | 70 | 100 | 76 | 95 | 93 | 79.8232484 | 88.06496661 | 1.103249597 | 0.526568677 | unchanged |
| hsa_circ_100063 | 259 | 252 | 205 | 249 | 179 | 166 | 238.565033 | 198.284381 | 0.831154418 | 0.261244018 | unchanged |
| hsa_circ_100064 | 79 | 71 | 54 | 70 | 55 | 58 | 68.14628429 | 61.26188773 | 0.8989762 | 0.465845383 | unchanged |
| hsa_circ_100065 | 131 | 101 | 98 | 123 | 92 | 98 | 110.35008 | 104.3179056 | 0.945336021 | 0.691597267 | unchanged |
| hsa_circ_100066 | 113 | 126 | 163 | 120 | 193 | 153 | 134.4136152 | 155.6690361 | 1.158134433 | 0.456779138 | unchanged |
| hsa_circ_100067 | 251 | 301 | 288 | 262 | 357 | 338 | 279.9463105 | 318.9065471 | 1.139170388 | 0.298457379 | unchanged |
| hsa_circ_100068 | 567 | 778 | 662 | 669 | 831 | 819 | 668.8620521 | 773.2291492 | 1.156036804 | 0.26385525 | unchanged |
| hsa_circ_100069 | 1442 | 2256 | 3239 | 1408 | 2177 | 2145 | 2312.555027 | 1910.127669 | 0.8259815 | 0.523870008 | unchanged |
| hsa_circ_100071 | 154 | 226 | 207 | 174 | 238 | 235 | 195.8059948 | 215.8525923 | 1.102379897 | 0.54235179 | unchanged |
| hsa_circ_100072 | 1041 | 474 | 658 | 713 | 454 | 451 | 724.343116 | 539.4385448 | 0.744727924 | 0.380965086 | unchanged |
| hsa_circ_100073 | 1238 | 2201 | 2171 | 1231 | 2043 | 2197 | 1869.756329 | 1823.323368 | 0.975166303 | 0.920229885 | unchanged |
| hsa_circ_100074 | 80 | 139 | 83 | 87 | 89 | 95 | 100.7543901 | 90.26238017 | 0.895865481 | 0.612389648 | unchanged |
| hsa_circ_100076 | 90 | 375 | 156 | 91 | 102 | 99 | 207.0314279 | 97.48662642 | 0.470878395 | 0.272990985 | unchanged |
| hsa_circ_100077 | 117 | 229 | 149 | 116 | 139 | 152 | 165.0801198 | 135.4153991 | 0.820301071 | 0.446411801 | unchanged |
| hsa_circ_100078 | 69 | 107 | 80 | 67 | 81 | 86 | 85.63121357 | 77.90023351 | 0.909717733 | 0.574785597 | unchanged |
| hsa_circ_100079 | 244 | 114 | 137 | 267 | 145 | 151 | 164.963845 | 187.4811911 | 1.136498674 | 0.710745828 | unchanged |
| hsa_circ_100080 | 1029 | 1004 | 481 | 907 | 554 | 514 | 837.9509517 | 658.3379257 | 0.785652101 | 0.456320393 | unchanged |
| hsa_circ_100081 | 62 | 62 | 60 | 64 | 65 | 58 | 61.20736482 | 62.6704461 | 1.02390368 | 0.562072026 | unchanged |
| hsa_circ_100082 | 64 | 109 | 92 | 58 | 55 | 54 | 88.25634891 | 55.71009974 | 0.631230506 | 0.070164763 | unchanged |
| hsa_circ_100083 | 3101 | 3727 | 3353 | 2835 | 3162 | 3131 | 3393.650873 | 3042.457921 | 0.896514708 | 0.168826143 | unchanged |
| hsa_circ_100084 | 1595 | 2943 | 1923 | 1978 | 2109 | 2109 | 2153.578658 | 2065.029574 | 0.958882818 | 0.838953296 | unchanged |
| hsa_circ_100085 | 2580 | 1404 | 1428 | 3591 | 1393 | 1331 | 1803.98801 | 2105.052621 | 1.166888366 | 0.73766397 | unchanged |
| hsa_circ_100086 | 248 | 130 | 151 | 278 | 147 | 142 | 176.1747845 | 189.2631309 | 1.074291826 | 0.830785788 | unchanged |
| hsa_circ_100087 | 89 | 78 | 85 | 68 | 86 | 80 | 84.06970396 | 78.11925608 | 0.929220069 | 0.395143168 | unchanged |
| hsa_circ_100088 | 104 | 95 | 101 | 97 | 84 | 86 | 100.0263579 | 88.91903086 | 0.888955999 | 0.082350169 | unchanged |
| hsa_circ_100089 | 618 | 1392 | 1233 | 536 | 962 | 1031 | 1080.89705 | 843.2610974 | 0.780149319 | 0.447224491 | unchanged |
| hsa_circ_100090 | 7751 | 9978 | 14502 | 7396 | 12565 | 12342 | 10743.67465 | 10767.76034 | 1.002241848 | 0.993068208 | unchanged |
| hsa_circ_100091 | 349 | 503 | 445 | 326 | 475 | 487 | 432.3703208 | 429.590359 | 0.993570415 | 0.969545387 | unchanged |
| hsa_circ_100093 | 294 | 153 | 173 | 220 | 138 | 144 | 206.9836985 | 167.3955326 | 0.808737759 | 0.484317915 | unchanged |
| hsa_circ_100094 | 69 | 165 | 86 | 68 | 76 | 80 | 106.6675162 | 74.81586581 | 0.701393156 | 0.34244179 | unchanged |
| hsa_circ_100095 | 213 | 93 | 351 | 179 | 136 | 144 | 219.0454389 | 152.7173113 | 0.697194665 | 0.429861882 | unchanged |
| hsa_circ_100096 | 94 | 90 | 205 | 102 | 86 | 86 | 129.6511057 | 91.54263091 | 0.706069034 | 0.37448565 | unchanged |
| hsa_circ_100097 | 83 | 90 | 87 | 84 | 85 | 90 | 86.76891433 | 86.30034841 | 0.994599841 | 0.87084551 | unchanged |
| hsa_circ_100099 | 175 | 282 | 178 | 192 | 174 | 181 | 211.3327984 | 182.3272556 | 0.86274945 | 0.460940231 | unchanged |
| hsa_circ_100100 | 68 | 69 | 80 | 76 | 104 | 111 | 72.15146799 | 97.09322833 | 1.34568611 | 0.093330741 | unchanged |
| hsa_circ_100102 | 62 | 79 | 64 | 64 | 70 | 73 | 68.17648803 | 69.11887352 | 1.013822734 | 0.882275799 | unchanged |
| hsa_circ_100103 | 255 | 345 | 400 | 317 | 447 | 452 | 333.397273 | 405.3283595 | 1.215751874 | 0.304887832 | unchanged |
| hsa_circ_100104 | 516 | 289 | 349 | 385 | 179 | 203 | 384.5671027 | 255.4526198 | 0.664260198 | 0.24169661 | unchanged |
| hsa_circ_100105 | 71 | 79 | 69 | 66 | 74 | 74 | 72.94577047 | 71.48883758 | 0.980027178 | 0.730114849 | unchanged |
| hsa_circ_100106 | 223 | 135 | 186 | 227 | 231 | 266 | 181.6861714 | 241.4596417 | 1.328992954 | 0.103775755 | unchanged |
| hsa_circ_100107 | 207 | 156 | 225 | 188 | 262 | 259 | 195.7805722 | 236.4223411 | 1.207588365 | 0.269601087 | unchanged |
| hsa_circ_100108 | 5641 | 7715 | 7918 | 6103 | 7985 | 8156 | 7091.04631 | 7414.897428 | 1.045670428 | 0.757848664 | unchanged |
| hsa_circ_100109 | 106 | 90 | 83 | 103 | 93 | 77 | 93.02789457 | 91.20896772 | 0.980447511 | 0.865952656 | unchanged |
| hsa_circ_100110 | 943 | 1452 | 1389 | 823 | 1274 | 1413 | 1261.093647 | 1169.767114 | 0.927581482 | 0.722480624 | unchanged |
| hsa_circ_100111 | 221 | 160 | 186 | 207 | 242 | 229 | 188.8742679 | 226.3457616 | 1.198393852 | 0.139926911 | unchanged |
| hsa_circ_100112 | 1276 | 1350 | 1312 | 1366 | 1763 | 1696 | 1312.837673 | 1608.315317 | 1.22506792 | 0.07628336 | unchanged |
| hsa_circ_100113 | 111 | 110 | 93 | 113 | 125 | 109 | 104.5975866 | 115.65223 | 1.105687365 | 0.221044806 | unchanged |
| hsa_circ_100114 | 169 | 278 | 285 | 140 | 236 | 244 | 244.044466 | 206.3610666 | 0.845587979 | 0.494691308 | unchanged |
| hsa_circ_100115 | 296 | 219 | 202 | 304 | 264 | 262 | 238.8643308 | 276.7924112 | 1.158785032 | 0.302656403 | unchanged |
| hsa_circ_100116 | 205 | 136 | 122 | 210 | 148 | 150 | 154.2467481 | 169.4194992 | 1.098366749 | 0.667631122 | unchanged |
| hsa_circ_100117 | 1152 | 714 | 369 | 919 | 467 | 429 | 744.8792543 | 604.9355658 | 0.812125673 | 0.638563484 | unchanged |
| hsa_circ_100118 | 96 | 103 | 126 | 102 | 131 | 133 | 108.4565987 | 121.7477796 | 1.122548384 | 0.385775362 | unchanged |
| hsa_circ_100119 | 205 | 182 | 248 | 192 | 175 | 184 | 211.4182362 | 183.9025804 | 0.869852023 | 0.241379369 | unchanged |
| hsa_circ_100120 | 151 | 156 | 180 | 140 | 167 | 153 | 162.3263221 | 153.3304976 | 0.94458185 | 0.49633859 | unchanged |
| hsa_circ_100121 | 83 | 143 | 78 | 74 | 77 | 75 | 101.2355 | 75.37125743 | 0.744514102 | 0.289286719 | unchanged |
| hsa_circ_100122 | 828 | 965 | 701 | 958 | 894 | 820 | 831.3061362 | 890.428125 | 1.071119394 | 0.530483715 | unchanged |
| hsa_circ_100123 | 649 | 681 | 624 | 541 | 777 | 746 | 651.6233545 | 687.958476 | 1.055760926 | 0.657738855 | unchanged |
| hsa_circ_100124 | 70 | 56 | 209 | 73 | 93 | 92 | 111.9017332 | 86.02183843 | 0.768726596 | 0.627594992 | unchanged |
| hsa_circ_100125 | 120 | 119 | 138 | 121 | 164 | 153 | 125.5765596 | 145.9961119 | 1.1626064 | 0.226683495 | unchanged |
| hsa_circ_100126 | 89 | 78 | 99 | 83 | 137 | 137 | 88.78865867 | 118.8050651 | 1.338065772 | 0.191383188 | unchanged |
| hsa_circ_100127 | 124 | 104 | 160 | 130 | 148 | 155 | 129.5516321 | 144.1353915 | 1.11257102 | 0.461465021 | unchanged |
| hsa_circ_100128 | 85 | 84 | 67 | 85 | 71 | 78 | 78.52937904 | 77.8184502 | 0.99094697 | 0.922942208 | unchanged |
| hsa_circ_100129 | 200 | 260 | 238 | 238 | 327 | 379 | 232.5862999 | 314.6341765 | 1.352763153 | 0.139792032 | unchanged |
| hsa_circ_100131 | 110 | 121 | 111 | 105 | 126 | 114 | 113.9372761 | 115.0446524 | 1.009719175 | 0.880545024 | unchanged |
| hsa_circ_100132 | 55 | 114 | 142 | 52 | 49 | 52 | 103.6746714 | 51.18253051 | 0.493684039 | 0.110365122 | unchanged |
| hsa_circ_100134 | 234 | 190 | 172 | 266 | 197 | 197 | 198.6655835 | 220.4391103 | 1.109598887 | 0.500851451 | unchanged |
| hsa_circ_100136 | 276 | 104 | 194 | 241 | 170 | 161 | 191.5896846 | 190.7235308 | 0.995479121 | 0.988357139 | unchanged |
| hsa_circ_100137 | 115 | 81 | 84 | 79 | 67 | 66 | 93.06725038 | 70.68719125 | 0.759528093 | 0.126549151 | unchanged |
| hsa_circ_100138 | 156 | 100 | 168 | 139 | 201 | 195 | 141.0573869 | 178.3760735 | 1.264563858 | 0.26352548 | unchanged |
| hsa_circ_100139 | 3388 | 5271 | 8275 | 3756 | 6850 | 7087 | 5644.511273 | 5897.707048 | 1.044856988 | 0.893889377 | unchanged |
| hsa_circ_100140 | 231 | 220 | 276 | 254 | 260 | 247 | 242.253039 | 253.7056827 | 1.047275542 | 0.549791841 | unchanged |
| hsa_circ_100141 | 89 | 76 | 109 | 88 | 92 | 90 | 91.07895969 | 90.06363413 | 0.988852249 | 0.921336784 | unchanged |
| hsa_circ_100145 | 70 | 46 | 73 | 66 | 78 | 67 | 63.26329957 | 70.53390367 | 1.114926097 | 0.482829389 | unchanged |
| hsa_circ_100146 | 529 | 1278 | 933 | 945 | 1239 | 1503 | 913.3269974 | 1228.953184 | 1.345578514 | 0.307211219 | unchanged |
| hsa_circ_100147 | 550 | 372 | 323 | 473 | 288 | 294 | 414.8214401 | 351.8683389 | 0.848240483 | 0.531215464 | unchanged |
| hsa_circ_100148 | 97 | 113 | 153 | 107 | 161 | 152 | 120.9839805 | 139.8756751 | 1.15615038 | 0.467657151 | unchanged |
| hsa_circ_100149 | 179 | 222 | 270 | 171 | 313 | 226 | 223.7421328 | 236.601673 | 1.057474826 | 0.80631493 | unchanged |
| hsa_circ_100150 | 274 | 299 | 287 | 272 | 294 | 291 | 286.516232 | 285.7971675 | 0.997490319 | 0.947433111 | unchanged |
| hsa_circ_100151 | 82 | 115 | 81 | 75 | 70 | 73 | 92.8059323 | 72.66212401 | 0.782946976 | 0.15015021 | unchanged |
| hsa_circ_100152 | 127 | 115 | 143 | 129 | 169 | 167 | 128.4951749 | 154.9216097 | 1.205660912 | 0.160413518 | unchanged |
| hsa_circ_100153 | 75 | 83 | 61 | 76 | 65 | 60 | 72.89143952 | 66.73028982 | 0.915474989 | 0.490774657 | unchanged |
| hsa_circ_100154 | 61 | 105 | 58 | 57 | 57 | 63 | 74.80507838 | 58.97339371 | 0.78836083 | 0.361184492 | unchanged |
| hsa_circ_100155 | 71 | 78 | 53 | 72 | 56 | 60 | 67.18600219 | 62.70525207 | 0.933308279 | 0.637718187 | unchanged |
| hsa_circ_100156 | 81 | 67 | 218 | 82 | 116 | 114 | 122.1084514 | 104.0313293 | 0.851958469 | 0.732445791 | unchanged |
| hsa_circ_100157 | 525 | 526 | 576 | 568 | 502 | 653 | 542.5204076 | 574.4512417 | 1.058856466 | 0.532274181 | unchanged |
| hsa_circ_100158 | 221 | 269 | 244 | 228 | 235 | 274 | 244.8635635 | 245.6540048 | 1.003228089 | 0.970035009 | unchanged |
| hsa_circ_100159 | 320 | 373 | 399 | 266 | 383 | 357 | 363.8916122 | 335.3058452 | 0.921444282 | 0.534717681 | unchanged |
| hsa_circ_100160 | 97 | 68 | 85 | 90 | 121 | 150 | 83.27086236 | 120.3471613 | 1.445249369 | 0.124464596 | unchanged |
| hsa_circ_100163 | 595 | 186 | 140 | 184 | 81 | 85 | 307.1783208 | 116.8644329 | 0.380444924 | 0.269455854 | unchanged |
| hsa_circ_100165 | 79 | 86 | 65 | 69 | 64 | 64 | 76.77337794 | 65.56129914 | 0.853958767 | 0.156531804 | unchanged |
| hsa_circ_100166 | 66 | 72 | 79 | 67 | 78 | 75 | 72.34465324 | 73.23660851 | 1.012329249 | 0.870667293 | unchanged |
| hsa_circ_100168 | 641 | 908 | 672 | 634 | 574 | 528 | 740.2394167 | 578.5629771 | 0.781588989 | 0.146769565 | unchanged |
| hsa_circ_100170 | 128 | 83 | 72 | 124 | 79 | 73 | 94.19649426 | 91.93176437 | 0.975957387 | 0.927233748 | unchanged |
| hsa_circ_100171 | 74 | 56 | 59 | 78 | 64 | 66 | 62.77941057 | 69.1278282 | 1.101122606 | 0.427177392 | unchanged |
| hsa_circ_100172 | 708 | 1582 | 1110 | 794 | 979 | 1013 | 1133.478362 | 928.5989255 | 0.819247157 | 0.477292246 | unchanged |
| hsa_circ_100173 | 199 | 167 | 543 | 205 | 119 | 128 | 302.9376175 | 150.4058944 | 0.49649131 | 0.284076902 | unchanged |
| hsa_circ_100174 | 79 | 75 | 58 | 64 | 63 | 71 | 70.76751288 | 65.78393548 | 0.929578175 | 0.519908206 | unchanged |
| hsa_circ_100175 | 1016 | 1374 | 999 | 1257 | 1422 | 1250 | 1129.639563 | 1309.686719 | 1.159384605 | 0.251913371 | unchanged |
| hsa_circ_100176 | 177 | 209 | 167 | 206 | 194 | 228 | 184.2074233 | 209.4420447 | 1.136990252 | 0.18614016 | unchanged |
| hsa_circ_100177 | 9028 | 10328 | 8433 | 8128 | 9210 | 9822 | 9263.146477 | 9053.191581 | 0.977334387 | 0.792687739 | unchanged |
| hsa_circ_100178 | 63 | 77 | 57 | 59 | 59 | 62 | 65.48731156 | 60.00616438 | 0.91630215 | 0.425879735 | unchanged |
| hsa_circ_100179 | 255 | 201 | 283 | 263 | 329 | 340 | 246.5702014 | 310.8093856 | 1.260531013 | 0.13189319 | unchanged |
| hsa_circ_100180 | 238 | 375 | 276 | 261 | 270 | 336 | 296.2330674 | 289.3388821 | 0.976727158 | 0.891373776 | unchanged |
| hsa_circ_100181 | 339 | 357 | 353 | 278 | 344 | 314 | 349.5161558 | 311.9638166 | 0.892559075 | 0.130594924 | unchanged |
| hsa_circ_100182 | 85 | 60 | 65 | 80 | 68 | 67 | 69.89371208 | 71.61605342 | 1.024642293 | 0.853704681 | unchanged |
| hsa_circ_100183 | 70 | 74 | 87 | 78 | 100 | 102 | 77.02523597 | 93.45709314 | 1.213330825 | 0.153745383 | unchanged |
| hsa_circ_100184 | 276 | 348 | 521 | 270 | 284 | 302 | 381.6210277 | 285.0449303 | 0.746931929 | 0.257577481 | unchanged |
| hsa_circ_100185 | 186 | 262 | 373 | 174 | 198 | 244 | 273.7603321 | 205.4423155 | 0.750445888 | 0.303479016 | unchanged |
| hsa_circ_100186 | 305 | 338 | 265 | 316 | 325 | 286 | 302.8469297 | 308.9673158 | 1.020209503 | 0.812317191 | unchanged |
| hsa_circ_100187 | 83 | 74 | 54 | 73 | 58 | 55 | 70.33327083 | 61.90164837 | 0.880119005 | 0.457109635 | unchanged |
| hsa_circ_100188 | 168 | 283 | 270 | 177 | 296 | 270 | 240.3974943 | 247.7434239 | 1.03055743 | 0.892902623 | unchanged |
| hsa_circ_100189 | 151 | 237 | 191 | 174 | 190 | 204 | 192.9523146 | 189.1759203 | 0.980428354 | 0.892263604 | unchanged |
| hsa_circ_100191 | 2590 | 6176 | 7801 | 3691 | 7795 | 7926 | 5522.043683 | 6470.894389 | 1.171829627 | 0.671102301 | unchanged |
| hsa_circ_100192 | 2437 | 3999 | 5745 | 2888 | 4724 | 4849 | 4060.112724 | 4153.611217 | 1.023028546 | 0.938917901 | unchanged |
| hsa_circ_100193 | 77 | 78 | 100 | 82 | 101 | 98 | 84.94464602 | 93.50915521 | 1.100824591 | 0.418147745 | unchanged |
| hsa_circ_100194 | 66 | 60 | 54 | 58 | 68 | 58 | 59.96290539 | 61.55685707 | 1.026582296 | 0.760494517 | unchanged |
| hsa_circ_100195 | 566 | 587 | 613 | 549 | 725 | 716 | 588.3530122 | 663.4663314 | 1.127667094 | 0.270778492 | unchanged |
| hsa_circ_100196 | 98 | 68 | 98 | 104 | 134 | 144 | 87.92067131 | 127.3283982 | 1.448219131 | 0.065477084 | unchanged |
| hsa_circ_100197 | 102 | 64 | 98 | 91 | 151 | 144 | 87.80728795 | 128.5367246 | 1.463850298 | 0.143043878 | unchanged |
| hsa_circ_100198 | 168 | 117 | 158 | 185 | 205 | 234 | 147.6688197 | 208.1707656 | 1.409713751 | 0.046412412 | unchanged |
| hsa_circ_100199 | 65 | 74 | 109 | 71 | 114 | 95 | 83.01792067 | 93.24115845 | 1.123144951 | 0.606001812 | unchanged |
| hsa_circ_100204 | 78 | 94 | 126 | 83 | 100 | 102 | 99.15412267 | 94.8368515 | 0.956458985 | 0.789904603 | unchanged |
| hsa_circ_100205 | 1735 | 1667 | 1007 | 1989 | 1377 | 1350 | 1469.364543 | 1572.265207 | 1.070030725 | 0.75825572 | unchanged |
| hsa_circ_100206 | 137 | 123 | 137 | 121 | 149 | 140 | 132.3344779 | 136.6683766 | 1.032749581 | 0.669564325 | unchanged |
| hsa_circ_100207 | 3537 | 3373 | 3010 | 4057 | 3169 | 3319 | 3306.717063 | 3515.107017 | 1.063020195 | 0.545127983 | unchanged |
| hsa_circ_100208 | 579 | 659 | 468 | 690 | 650 | 720 | 568.5212867 | 686.6684254 | 1.207814802 | 0.115527373 | unchanged |
| hsa_circ_100209 | 1862 | 1901 | 1597 | 1870 | 2274 | 2193 | 1786.355173 | 2112.612013 | 1.182638282 | 0.104740458 | unchanged |
| hsa_circ_100211 | 342 | 315 | 251 | 375 | 332 | 320 | 302.6087397 | 342.1706446 | 1.130736161 | 0.281097634 | unchanged |
| hsa_circ_100213 | 1397 | 2023 | 1608 | 2103 | 1760 | 1750 | 1676.118155 | 1871.197495 | 1.116387582 | 0.420347735 | unchanged |
| hsa_circ_100215 | 140 | 156 | 215 | 153 | 155 | 173 | 170.1987443 | 160.2928703 | 0.941798195 | 0.69992899 | unchanged |
| hsa_circ_100216 | 104 | 108 | 120 | 98 | 113 | 108 | 110.5277034 | 106.3282488 | 0.962005411 | 0.542852027 | unchanged |
| hsa_circ_100218 | 76 | 60 | 81 | 66 | 73 | 73 | 72.32245926 | 70.60671759 | 0.976276503 | 0.815475839 | unchanged |
| hsa_circ_100219 | 92 | 118 | 89 | 88 | 88 | 82 | 99.32066115 | 85.96152104 | 0.865494853 | 0.231075818 | unchanged |
| hsa_circ_100220 | 73 | 63 | 64 | 76 | 63 | 64 | 66.70612505 | 67.82623769 | 1.016791751 | 0.840527928 | unchanged |
| hsa_circ_100221 | 68 | 60 | 223 | 67 | 69 | 65 | 117.121577 | 66.99009093 | 0.571970534 | 0.399372775 | unchanged |
| hsa_circ_100222 | 2038 | 3125 | 3161 | 2403 | 3365 | 3428 | 2774.701527 | 3065.328831 | 1.104741826 | 0.589369028 | unchanged |
| hsa_circ_100223 | 32406 | 28401 | 29651 | 26787 | 30444 | 30444 | 30152.76446 | 29225.07739 | 0.969233764 | 0.613985783 | unchanged |
| hsa_circ_100225 | 85 | 58 | 74 | 84 | 95 | 92 | 72.09751094 | 90.3029654 | 1.252511553 | 0.100844853 | unchanged |
| hsa_circ_100226 | 459 | 281 | 287 | 474 | 338 | 341 | 342.2452244 | 384.3023403 | 1.12288591 | 0.59873519 | unchanged |
| hsa_circ_100227 | 1008 | 642 | 460 | 1059 | 736 | 686 | 703.1578143 | 827.1289193 | 1.176306232 | 0.567109012 | unchanged |
| hsa_circ_100228 | 209 | 134 | 175 | 235 | 251 | 237 | 172.8472021 | 241.1222465 | 1.395002312 | 0.037751643 | unchanged |
| hsa_circ_100229 | 529 | 760 | 696 | 664 | 918 | 866 | 661.6980433 | 815.9253777 | 1.233078118 | 0.211432499 | unchanged |
| hsa_circ_100230 | 800 | 246 | 186 | 458 | 158 | 154 | 410.616448 | 256.5755611 | 0.624854563 | 0.52224468 | unchanged |
| hsa_circ_100231 | 212 | 132 | 136 | 173 | 146 | 142 | 160.0365872 | 153.649623 | 0.9600906 | 0.830179673 | unchanged |
| hsa_circ_100233 | 84 | 75 | 76 | 90 | 77 | 76 | 78.19596993 | 80.87966505 | 1.034320121 | 0.648124328 | unchanged |
| hsa_circ_100234 | 111 | 63 | 117 | 83 | 101 | 121 | 97.05735423 | 101.8414486 | 1.049291416 | 0.82419568 | unchanged |
| hsa_circ_100235 | 66 | 55 | 69 | 70 | 67 | 67 | 63.22943578 | 68.09015092 | 1.076874245 | 0.324898923 | unchanged |
| hsa_circ_100236 | 38295 | 32901 | 34105 | 48369 | 41089 | 38709 | 35100.32277 | 42722.56032 | 1.217155768 | 0.084212675 | unchanged |
| hsa_circ_100237 | 149 | 212 | 207 | 156 | 202 | 200 | 189.2327261 | 185.8044538 | 0.981883301 | 0.898349288 | unchanged |
| hsa_circ_100238 | 227 | 293 | 215 | 238 | 207 | 250 | 245.3537285 | 231.8932878 | 0.945138634 | 0.649811276 | unchanged |
| hsa_circ_100239 | 1060 | 890 | 1060 | 909 | 874 | 925 | 1003.431467 | 902.6714623 | 0.899584568 | 0.160040251 | unchanged |
| hsa_circ_100240 | 101 | 98 | 120 | 108 | 127 | 132 | 106.1863974 | 122.3463892 | 1.152185141 | 0.189396958 | unchanged |
| hsa_circ_100241 | 75 | 103 | 171 | 73 | 72 | 76 | 116.0145195 | 73.67961801 | 0.635089628 | 0.213097936 | unchanged |
| hsa_circ_100242 | 65 | 56 | 73 | 67 | 78 | 77 | 64.74637903 | 74.24909627 | 1.146768319 | 0.18390349 | unchanged |
| hsa_circ_100243 | 121 | 138 | 105 | 124 | 116 | 118 | 121.0834808 | 119.3101642 | 0.985354595 | 0.865635856 | unchanged |
| hsa_circ_100244 | 149 | 145 | 122 | 133 | 114 | 123 | 138.8681862 | 123.4564559 | 0.889018999 | 0.194800726 | unchanged |
| hsa_circ_100247 | 90 | 67 | 76 | 86 | 90 | 86 | 77.66427031 | 87.49934635 | 1.126635788 | 0.215711682 | unchanged |
| hsa_circ_100248 | 145 | 168 | 193 | 181 | 258 | 255 | 168.4765222 | 231.2630214 | 1.372672099 | 0.093699864 | unchanged |
| hsa_circ_100249 | 63 | 119 | 158 | 91 | 150 | 148 | 113.3509777 | 130.0477934 | 1.147301912 | 0.648067323 | unchanged |
| hsa_circ_100250 | 139 | 110 | 115 | 141 | 99 | 101 | 121.4276972 | 113.8038636 | 0.937215036 | 0.66338334 | unchanged |
| hsa_circ_100251 | 382 | 414 | 292 | 405 | 335 | 357 | 362.6618926 | 365.7242288 | 1.008444053 | 0.945218934 | unchanged |
| hsa_circ_100252 | 90 | 75 | 109 | 103 | 128 | 128 | 91.43912364 | 119.8885804 | 1.311130024 | 0.089606272 | unchanged |
| hsa_circ_100253 | 324 | 427 | 365 | 406 | 458 | 470 | 371.9617768 | 444.5722685 | 1.195209552 | 0.11231366 | unchanged |
| hsa_circ_100254 | 74 | 71 | 60 | 78 | 72 | 68 | 68.32589385 | 72.90004282 | 1.066946054 | 0.441779693 | unchanged |
| hsa_circ_100255 | 169 | 173 | 193 | 199 | 234 | 208 | 178.5697013 | 213.5953296 | 1.196145416 | 0.055277995 | unchanged |
| hsa_circ_100256 | 121 | 110 | 283 | 131 | 151 | 141 | 171.4794176 | 141.0133239 | 0.822333816 | 0.615611679 | unchanged |
| hsa_circ_100257 | 916 | 1742 | 1625 | 1246 | 1946 | 1862 | 1427.779892 | 1684.576034 | 1.179856954 | 0.491447851 | unchanged |
| hsa_circ_100259 | 97 | 99 | 107 | 95 | 100 | 102 | 101.2131292 | 99.0488212 | 0.978616332 | 0.607036941 | unchanged |
| hsa_circ_100261 | 85 | 65 | 93 | 88 | 101 | 104 | 80.93907949 | 97.60817658 | 1.205946215 | 0.165189118 | unchanged |
| hsa_circ_100262 | 419 | 351 | 294 | 382 | 422 | 348 | 354.7434321 | 383.9735621 | 1.082397946 | 0.524869664 | unchanged |
| hsa_circ_100263 | 60 | 51 | 67 | 63 | 62 | 63 | 59.24111196 | 62.88413409 | 1.06149483 | 0.450523443 | unchanged |
| hsa_circ_100266 | 64 | 54 | 164 | 69 | 82 | 73 | 93.81499166 | 74.70120433 | 0.796260843 | 0.615903338 | unchanged |
| hsa_circ_100267 | 89 | 94 | 94 | 110 | 87 | 87 | 92.01962266 | 94.76729691 | 1.029859656 | 0.734746161 | unchanged |
| hsa_circ_100269 | 770 | 899 | 372 | 761 | 315 | 337 | 680.497459 | 470.8428056 | 0.691909719 | 0.384682423 | unchanged |
| hsa_circ_100270 | 315 | 458 | 373 | 393 | 499 | 512 | 381.9560955 | 467.8970382 | 1.22500215 | 0.201339636 | unchanged |
| hsa_circ_100271 | 122 | 123 | 120 | 124 | 142 | 135 | 121.5049758 | 133.4326532 | 1.098166164 | 0.085520508 | unchanged |
| hsa_circ_100272 | 1507 | 1050 | 866 | 1265 | 1310 | 1301 | 1140.808941 | 1292.207287 | 1.132711395 | 0.472255538 | unchanged |
| hsa_circ_100273 | 735 | 668 | 656 | 704 | 906 | 958 | 686.3264809 | 855.8771243 | 1.247040801 | 0.104888226 | unchanged |
| hsa_circ_100274 | 62 | 59 | 58 | 68 | 59 | 54 | 59.80068778 | 60.57943959 | 1.013022456 | 0.864864779 | unchanged |
| hsa_circ_100277 | 49 | 96 | 52 | 48 | 52 | 57 | 65.87140716 | 52.40703217 | 0.795596063 | 0.433542842 | unchanged |
| hsa_circ_100278 | 282 | 356 | 250 | 277 | 243 | 269 | 296.1851951 | 263.0757616 | 0.888213746 | 0.37431438 | unchanged |
| hsa_circ_100279 | 112 | 70 | 102 | 125 | 127 | 122 | 94.7107704 | 124.8252857 | 1.317962944 | 0.076556884 | unchanged |
| hsa_circ_100280 | 177 | 212 | 184 | 147 | 170 | 164 | 191.0203238 | 160.5261963 | 0.840361869 | 0.073400589 | unchanged |
| hsa_circ_100283 | 224 | 93 | 100 | 141 | 105 | 101 | 138.722786 | 115.8095319 | 0.834827033 | 0.632762887 | unchanged |
| hsa_circ_100284 | 78 | 77 | 78 | 79 | 94 | 96 | 77.69790208 | 89.58070895 | 1.152936007 | 0.089290588 | unchanged |
| hsa_circ_100286 | 134 | 160 | 213 | 157 | 240 | 232 | 169.0398595 | 209.5493013 | 1.239644318 | 0.312414123 | unchanged |
| hsa_circ_100287 | 109 | 96 | 143 | 120 | 148 | 164 | 116.0087579 | 143.9853998 | 1.24115974 | 0.214772361 | unchanged |
| hsa_circ_100288 | 76 | 67 | 75 | 65 | 78 | 82 | 72.65913976 | 74.94212276 | 1.031420452 | 0.717264418 | unchanged |
| hsa_circ_100289 | 65 | 52 | 65 | 60 | 64 | 63 | 60.6000944 | 62.35202529 | 1.028909706 | 0.708593248 | unchanged |
| hsa_circ_100291 | 97 | 88 | 139 | 93 | 155 | 154 | 107.9161437 | 134.1302471 | 1.242911788 | 0.371118572 | unchanged |
| hsa_circ_100292 | 246 | 406 | 467 | 308 | 598 | 537 | 372.9797748 | 480.8472684 | 1.289204673 | 0.383644587 | unchanged |
| hsa_circ_100294 | 65 | 80 | 65 | 69 | 77 | 75 | 69.91132945 | 73.83182633 | 1.056078134 | 0.518049134 | unchanged |
| hsa_circ_100295 | 90 | 69 | 233 | 88 | 101 | 105 | 130.8490646 | 98.12366172 | 0.749899604 | 0.56097952 | unchanged |
| hsa_circ_100296 | 258 | 115 | 150 | 172 | 130 | 132 | 174.6828101 | 144.5848142 | 0.827699155 | 0.542249394 | unchanged |
| hsa_circ_100297 | 66 | 77 | 74 | 76 | 75 | 80 | 72.31531071 | 77.31370858 | 1.069119496 | 0.253645795 | unchanged |
| hsa_circ_100299 | 142 | 112 | 165 | 165 | 204 | 206 | 139.6561624 | 191.5469454 | 1.371560998 | 0.065458229 | unchanged |
| hsa_circ_100301 | 80 | 73 | 78 | 77 | 82 | 88 | 76.71461883 | 82.21766995 | 1.071734061 | 0.225531741 | unchanged |
| hsa_circ_100302 | 1494 | 814 | 1091 | 1053 | 639 | 641 | 1133.139213 | 777.6883671 | 0.686313171 | 0.213612799 | unchanged |
| hsa_circ_100303 | 115 | 92 | 107 | 111 | 126 | 113 | 104.4833203 | 116.6320526 | 1.116274371 | 0.215671641 | unchanged |
| hsa_circ_100305 | 67 | 72 | 147 | 75 | 62 | 72 | 95.41507301 | 69.99841936 | 0.733620141 | 0.386298369 | unchanged |
| hsa_circ_100306 | 114 | 83 | 127 | 121 | 130 | 128 | 107.892211 | 126.503218 | 1.172496298 | 0.239829795 | unchanged |
| hsa_circ_100307 | 77 | 62 | 78 | 71 | 80 | 82 | 72.6286967 | 77.46072166 | 1.06653052 | 0.475452055 | unchanged |
| hsa_circ_100308 | 702 | 685 | 606 | 576 | 576 | 620 | 664.2924547 | 590.5032815 | 0.888920651 | 0.090700158 | unchanged |
| hsa_circ_100309 | 101 | 106 | 111 | 100 | 96 | 99 | 105.883946 | 98.34630056 | 0.928812198 | 0.07760187 | unchanged |
| hsa_circ_100310 | 127 | 100 | 84 | 124 | 68 | 67 | 103.8123803 | 86.22048169 | 0.830541419 | 0.482720976 | unchanged |
| hsa_circ_100312 | 545 | 523 | 534 | 350 | 458 | 454 | 533.98909 | 420.2729548 | 0.787044085 | 0.034042115 | unchanged |
| hsa_circ_100313 | 516 | 505 | 748 | 407 | 904 | 868 | 589.5040313 | 726.4670022 | 1.232335936 | 0.486277344 | unchanged |
| hsa_circ_100314 | 59 | 78 | 57 | 60 | 63 | 65 | 64.71008283 | 62.52360014 | 0.966211097 | 0.764208415 | unchanged |
| hsa_circ_100315 | 80 | 93 | 70 | 75 | 64 | 66 | 81.16138024 | 68.6781007 | 0.846191877 | 0.159460484 | unchanged |
| hsa_circ_100317 | 61 | 102 | 188 | 74 | 63 | 63 | 116.7129679 | 66.71339939 | 0.571602287 | 0.253867294 | unchanged |
| hsa_circ_100318 | 132 | 118 | 151 | 148 | 154 | 154 | 133.9371958 | 151.7008973 | 1.132627097 | 0.145667752 | unchanged |
| hsa_circ_100319 | 359 | 221 | 183 | 309 | 213 | 223 | 254.3955763 | 248.473586 | 0.976721332 | 0.927876753 | unchanged |
| hsa_circ_100320 | 82 | 61 | 173 | 86 | 89 | 81 | 105.6166359 | 85.38787486 | 0.808469936 | 0.588688768 | unchanged |
| hsa_circ_100322 | 139 | 129 | 141 | 147 | 145 | 149 | 136.2447825 | 147.1062455 | 1.079720213 | 0.050602012 | unchanged |
| hsa_circ_100323 | 372 | 590 | 495 | 330 | 411 | 364 | 485.5593869 | 368.2481176 | 0.75839975 | 0.15654389 | unchanged |
| hsa_circ_100324 | 76 | 104 | 75 | 76 | 65 | 70 | 85.07486494 | 70.3457489 | 0.82686877 | 0.218625907 | unchanged |
| hsa_circ_100326 | 225 | 138 | 140 | 146 | 112 | 119 | 167.660564 | 125.7201208 | 0.749849087 | 0.239004581 | unchanged |
| hsa_circ_100327 | 607 | 687 | 575 | 547 | 484 | 574 | 623.1000018 | 534.8165723 | 0.858315793 | 0.107371852 | unchanged |
| hsa_circ_100328 | 140 | 149 | 181 | 127 | 167 | 135 | 156.7496181 | 143.0498354 | 0.912600854 | 0.469579439 | unchanged |
| hsa_circ_100329 | 2191 | 1776 | 1510 | 2057 | 1765 | 1753 | 1825.562736 | 1858.442277 | 1.018010633 | 0.889253061 | unchanged |
| hsa_circ_100330 | 79 | 76 | 55 | 70 | 60 | 62 | 69.69997624 | 63.99950887 | 0.918214214 | 0.513085051 | unchanged |
| hsa_circ_100331 | 207 | 235 | 289 | 206 | 261 | 263 | 243.6521338 | 243.2611874 | 0.998395473 | 0.990408715 | unchanged |
| hsa_circ_100332 | 346 | 445 | 337 | 331 | 333 | 384 | 376.1607123 | 349.4298047 | 0.928937534 | 0.527000057 | unchanged |
| hsa_circ_100333 | 123 | 82 | 103 | 115 | 100 | 102 | 102.7695008 | 105.6151835 | 1.027689953 | 0.835068187 | unchanged |
| hsa_circ_100335 | 101 | 99 | 119 | 125 | 118 | 125 | 106.3484174 | 122.7523853 | 1.15424741 | 0.073946336 | unchanged |
| hsa_circ_100336 | 111 | 109 | 145 | 135 | 171 | 171 | 121.4645342 | 159.0762636 | 1.309651946 | 0.087907022 | unchanged |
| hsa_circ_100337 | 136 | 121 | 92 | 151 | 121 | 133 | 116.1766298 | 135.0232897 | 1.162224192 | 0.294885329 | unchanged |
| hsa_circ_100339 | 318 | 236 | 238 | 331 | 337 | 327 | 263.8018673 | 331.6579172 | 1.257223539 | 0.067027569 | unchanged |
| hsa_circ_100342 | 260 | 303 | 251 | 263 | 249 | 260 | 271.3591896 | 257.2863776 | 0.948139542 | 0.442681338 | unchanged |
| hsa_circ_100344 | 184 | 140 | 150 | 193 | 176 | 171 | 158.1705315 | 180.0432516 | 1.138285684 | 0.217056911 | unchanged |
| hsa_circ_100345 | 123 | 93 | 89 | 98 | 82 | 87 | 101.4805601 | 88.92441831 | 0.876270472 | 0.348523615 | unchanged |
| hsa_circ_100347 | 129 | 73 | 121 | 122 | 138 | 138 | 107.7743425 | 132.6050606 | 1.23039545 | 0.24564454 | unchanged |
| hsa_circ_100350 | 943 | 750 | 713 | 768 | 804 | 777 | 802.1718388 | 783.1117967 | 0.976239452 | 0.80474086 | unchanged |
| hsa_circ_100351 | 1248 | 1490 | 1781 | 1495 | 1813 | 1849 | 1506.415997 | 1718.905296 | 1.141056189 | 0.327754588 | unchanged |
| hsa_circ_100352 | 414 | 1579 | 1379 | 855 | 1568 | 1542 | 1124.181637 | 1321.66122 | 1.175665192 | 0.669065667 | unchanged |
| hsa_circ_100353 | 247 | 345 | 267 | 282 | 309 | 309 | 286.3995821 | 300.2198932 | 1.048255347 | 0.681373475 | unchanged |
| hsa_circ_100354 | 110 | 173 | 117 | 107 | 117 | 125 | 133.1139844 | 116.1560576 | 0.872605971 | 0.453832438 | unchanged |
| hsa_circ_100355 | 148 | 115 | 152 | 129 | 139 | 132 | 138.2253205 | 133.3349104 | 0.964620013 | 0.709961489 | unchanged |
| hsa_circ_100356 | 1253 | 1781 | 1532 | 1395 | 1607 | 1754 | 1521.941243 | 1585.577608 | 1.041812629 | 0.747756681 | unchanged |
| hsa_circ_100357 | 177 | 304 | 309 | 265 | 373 | 348 | 263.4352858 | 328.6856718 | 1.24769038 | 0.294818515 | unchanged |
| hsa_circ_100358 | 99 | 109 | 91 | 105 | 74 | 68 | 99.6212579 | 82.20583836 | 0.825183702 | 0.236480188 | unchanged |
| hsa_circ_100359 | 74 | 58 | 84 | 75 | 88 | 85 | 72.20424087 | 82.97298075 | 1.149142762 | 0.278719631 | unchanged |
| hsa_circ_100360 | 286 | 281 | 321 | 505 | 358 | 363 | 296.1746928 | 408.6415348 | 1.379731438 | 0.087412566 | unchanged |
| hsa_circ_100361 | 207 | 362 | 200 | 170 | 211 | 209 | 256.4617758 | 196.6959019 | 0.766959916 | 0.333789279 | unchanged |
| hsa_circ_100362 | 70 | 61 | 74 | 61 | 67 | 66 | 68.20414789 | 64.45839379 | 0.94508026 | 0.424714773 | unchanged |
| hsa_circ_100364 | 289 | 154 | 237 | 330 | 361 | 361 | 226.8128161 | 350.516502 | 1.545399894 | 0.03841947 | unchanged |
| hsa_circ_100365 | 150 | 153 | 160 | 139 | 142 | 160 | 154.0846372 | 146.9124097 | 0.953452676 | 0.3667241 | unchanged |
| hsa_circ_100366 | 198 | 336 | 285 | 189 | 193 | 185 | 272.7451946 | 189.0539353 | 0.693152213 | 0.106844554 | unchanged |
| hsa_circ_100367 | 15562 | 16366 | 11715 | 18008 | 15968 | 17294 | 14547.58399 | 17089.64712 | 1.174741258 | 0.177338883 | unchanged |
| hsa_circ_100368 | 132 | 175 | 125 | 147 | 125 | 129 | 144.1539252 | 133.4418448 | 0.925689985 | 0.566060999 | unchanged |
| hsa_circ_100369 | 75 | 70 | 86 | 76 | 91 | 92 | 76.83141919 | 86.38940597 | 1.12440206 | 0.244413397 | unchanged |
| hsa_circ_100370 | 237 | 359 | 319 | 257 | 355 | 429 | 304.9615903 | 347.1497641 | 1.138339303 | 0.529365125 | unchanged |
| hsa_circ_100371 | 181 | 267 | 253 | 203 | 338 | 313 | 233.8209912 | 284.7420462 | 1.217777945 | 0.361056683 | unchanged |
| hsa_circ_100372 | 75 | 73 | 90 | 67 | 104 | 101 | 79.594726 | 90.44733714 | 1.13634837 | 0.445495863 | unchanged |
| hsa_circ_100373 | 3029 | 2064 | 1799 | 3611 | 2664 | 2535 | 2297.229084 | 2936.313944 | 1.27819814 | 0.274214544 | unchanged |
| hsa_circ_100374 | 1720 | 907 | 760 | 2708 | 1274 | 1315 | 1129.070006 | 1765.798259 | 1.563940455 | 0.31753147 | unchanged |
| hsa_circ_100376 | 73 | 61 | 63 | 70 | 65 | 64 | 65.78771076 | 66.24612923 | 1.006968148 | 0.914834776 | unchanged |
| hsa_circ_100378 | 66 | 65 | 96 | 63 | 62 | 64 | 75.86076106 | 63.35288863 | 0.835120657 | 0.291482147 | unchanged |
| hsa_circ_100379 | 77 | 83 | 68 | 78 | 84 | 82 | 76.21364286 | 81.06243826 | 1.063621095 | 0.373638002 | unchanged |
| hsa_circ_100380 | 222 | 135 | 142 | 159 | 162 | 160 | 166.4657323 | 160.4169064 | 0.963663237 | 0.83857747 | unchanged |
| hsa_circ_100381 | 153 | 104 | 162 | 132 | 178 | 175 | 139.7251694 | 161.7977355 | 1.157971296 | 0.398207393 | unchanged |
| hsa_circ_100382 | 454 | 517 | 524 | 485 | 681 | 657 | 498.6384015 | 607.7152942 | 1.218749483 | 0.170834585 | unchanged |
| hsa_circ_100383 | 111 | 163 | 128 | 122 | 142 | 141 | 133.7838593 | 134.8212879 | 1.007754513 | 0.952917095 | unchanged |
| hsa_circ_100384 | 130 | 100 | 111 | 142 | 113 | 133 | 113.8659997 | 129.4270666 | 1.136661224 | 0.273348959 | unchanged |
| hsa_circ_100385 | 74 | 68 | 60 | 80 | 48 | 49 | 67.01617531 | 58.75423608 | 0.876717237 | 0.504163352 | unchanged |
| hsa_circ_100387 | 153 | 337 | 277 | 163 | 266 | 279 | 255.7210828 | 235.7885673 | 0.922053688 | 0.775282306 | unchanged |
| hsa_circ_100388 | 625 | 656 | 457 | 668 | 715 | 700 | 579.1832605 | 694.382399 | 1.198899289 | 0.142713532 | unchanged |
| hsa_circ_100389 | 475 | 259 | 214 | 318 | 221 | 224 | 316.1812776 | 254.7281768 | 0.805639659 | 0.517080781 | unchanged |
| hsa_circ_100390 | 178 | 239 | 235 | 216 | 318 | 326 | 217.182172 | 286.7152343 | 1.320160083 | 0.162631624 | unchanged |
| hsa_circ_100391 | 21465 | 17797 | 9947 | 16209 | 11810 | 12109 | 16402.84532 | 13376.03763 | 0.81547057 | 0.457194178 | unchanged |
| hsa_circ_100395 | 5478 | 3202 | 3361 | 4769 | 3428 | 3528 | 4013.514276 | 3908.265902 | 0.973776504 | 0.90751665 | unchanged |
| hsa_circ_100396 | 148 | 86 | 138 | 132 | 158 | 158 | 124.3843467 | 149.2769356 | 1.200126378 | 0.303958646 | unchanged |
| hsa_circ_100397 | 646 | 283 | 236 | 454 | 244 | 252 | 388.1314951 | 316.6451262 | 0.815819201 | 0.651453784 | unchanged |
| hsa_circ_100398 | 151 | 85 | 78 | 124 | 91 | 82 | 104.7110442 | 98.97299262 | 0.945201085 | 0.839471022 | unchanged |
| hsa_circ_100399 | 72 | 59 | 72 | 65 | 73 | 69 | 67.47904855 | 69.25072845 | 1.026255259 | 0.727118076 | unchanged |
| hsa_circ_100403 | 771 | 856 | 671 | 771 | 795 | 796 | 765.8931224 | 787.3855004 | 1.02806185 | 0.711188941 | unchanged |
| hsa_circ_100404 | 1337 | 1264 | 1043 | 1565 | 1478 | 1461 | 1214.546355 | 1501.549093 | 1.236304474 | 0.038249668 | unchanged |
| hsa_circ_100405 | 639 | 1531 | 1671 | 662 | 1362 | 1487 | 1280.51013 | 1170.223513 | 0.913872905 | 0.802565255 | unchanged |
| hsa_circ_100407 | 163 | 129 | 178 | 183 | 211 | 212 | 156.6507116 | 202.2956032 | 1.291380047 | 0.056831148 | unchanged |
| hsa_circ_100410 | 114 | 102 | 137 | 111 | 150 | 151 | 117.5591832 | 137.1142218 | 1.166342076 | 0.30007706 | unchanged |
| hsa_circ_100411 | 5185 | 4152 | 4579 | 5429 | 4585 | 4242 | 4638.576816 | 4751.810051 | 1.024411202 | 0.818732477 | unchanged |
| hsa_circ_100412 | 4245 | 1944 | 1761 | 3234 | 1919 | 2046 | 2650.083553 | 2399.386619 | 0.905400366 | 0.794926579 | unchanged |
| hsa_circ_100413 | 615 | 187 | 164 | 547 | 211 | 191 | 322.3975449 | 316.3195293 | 0.981147451 | 0.975569839 | unchanged |
| hsa_circ_100414 | 78 | 78 | 118 | 82 | 106 | 115 | 91.4762098 | 100.9147667 | 1.103180454 | 0.601795037 | unchanged |
| hsa_circ_100416 | 433 | 431 | 363 | 456 | 390 | 396 | 409.0255164 | 413.9902699 | 1.012138004 | 0.880870765 | unchanged |
| hsa_circ_100418 | 81 | 65 | 61 | 71 | 81 | 72 | 68.99503994 | 74.84682547 | 1.084814583 | 0.439223025 | unchanged |
| hsa_circ_100419 | 80 | 85 | 73 | 87 | 76 | 74 | 79.31378546 | 78.87045661 | 0.994410444 | 0.936663757 | unchanged |
| hsa_circ_100420 | 2444 | 2694 | 2348 | 2723 | 2650 | 2808 | 2495.353628 | 2726.800069 | 1.092750959 | 0.109010305 | unchanged |
| hsa_circ_100421 | 853 | 923 | 676 | 916 | 991 | 1029 | 817.3381989 | 978.582807 | 1.197280157 | 0.115795585 | unchanged |
| hsa_circ_100422 | 1773 | 1328 | 1163 | 1538 | 1521 | 1612 | 1421.24327 | 1557.170091 | 1.095639378 | 0.502188125 | unchanged |
| hsa_circ_100425 | 747 | 1112 | 855 | 882 | 1203 | 1233 | 904.7193644 | 1105.989438 | 1.222466858 | 0.266974209 | unchanged |
| hsa_circ_100426 | 459 | 566 | 589 | 480 | 566 | 554 | 537.9106293 | 533.4324636 | 0.991674889 | 0.930452715 | unchanged |
| hsa_circ_100427 | 82 | 88 | 77 | 78 | 76 | 69 | 82.52312757 | 74.26799185 | 0.899965792 | 0.120235573 | unchanged |
| hsa_circ_100429 | 108 | 125 | 187 | 119 | 125 | 129 | 140.1013536 | 124.5018749 | 0.88865576 | 0.555818959 | unchanged |
| hsa_circ_100430 | 329 | 328 | 598 | 360 | 541 | 465 | 418.2874307 | 455.6362511 | 1.089289846 | 0.737579743 | unchanged |
| hsa_circ_100433 | 1670 | 1477 | 1200 | 1287 | 1497 | 1480 | 1448.80103 | 1421.642278 | 0.981254326 | 0.867017505 | unchanged |
| hsa_circ_100434 | 79 | 147 | 73 | 87 | 68 | 71 | 99.63887692 | 75.16361102 | 0.754360279 | 0.372848428 | unchanged |
| hsa_circ_100435 | 1898 | 2726 | 2441 | 2137 | 2979 | 3057 | 2354.991698 | 2724.537264 | 1.156920114 | 0.388003632 | unchanged |
| hsa_circ_100438 | 4112 | 5870 | 4722 | 5004 | 5011 | 5080 | 4901.060614 | 5031.623848 | 1.026639792 | 0.812746829 | unchanged |
| hsa_circ_100439 | 312 | 424 | 322 | 384 | 477 | 483 | 352.5069413 | 448.1661696 | 1.271368354 | 0.11738885 | unchanged |
| hsa_circ_100441 | 113 | 114 | 64 | 117 | 70 | 72 | 96.71935636 | 86.23722357 | 0.891623216 | 0.667309359 | unchanged |
| hsa_circ_100443 | 155 | 116 | 115 | 125 | 112 | 129 | 128.7733085 | 122.0163728 | 0.947528445 | 0.656252419 | unchanged |
| hsa_circ_100444 | 112 | 85 | 111 | 127 | 117 | 123 | 102.5844495 | 122.3571823 | 1.192745907 | 0.104608403 | unchanged |
| hsa_circ_100445 | 4353 | 6625 | 7722 | 5646 | 6368 | 7000 | 6233.43273 | 6337.69752 | 1.016726705 | 0.926801381 | unchanged |
| hsa_circ_100446 | 89 | 74 | 103 | 96 | 127 | 100 | 88.49308244 | 107.7577784 | 1.217697196 | 0.208733118 | unchanged |
| hsa_circ_100447 | 1020 | 1426 | 1268 | 1023 | 1208 | 1203 | 1238.05212 | 1144.372971 | 0.924333437 | 0.520056903 | unchanged |
| hsa_circ_100448 | 112 | 83 | 96 | 96 | 93 | 96 | 96.79684189 | 94.89590344 | 0.980361565 | 0.831928499 | unchanged |
| hsa_circ_100449 | 633 | 590 | 558 | 647 | 624 | 633 | 593.5547206 | 634.7650566 | 1.069429717 | 0.14155223 | unchanged |
| hsa_circ_100450 | 181 | 285 | 220 | 153 | 193 | 208 | 228.3048384 | 184.3703719 | 0.807562263 | 0.271340532 | unchanged |
| hsa_circ_100452 | 71 | 61 | 72 | 64 | 79 | 77 | 68.09624593 | 73.552676 | 1.080128207 | 0.403153473 | unchanged |
| hsa_circ_100453 | 1220 | 522 | 327 | 1013 | 408 | 412 | 689.3268432 | 610.8978466 | 0.886223789 | 0.827695773 | unchanged |
| hsa_circ_100454 | 87 | 124 | 91 | 121 | 109 | 123 | 100.3279835 | 117.6211983 | 1.172366813 | 0.238180465 | unchanged |
| hsa_circ_100455 | 107 | 131 | 125 | 118 | 125 | 125 | 120.9036503 | 122.6022284 | 1.014049022 | 0.832933436 | unchanged |
| hsa_circ_100456 | 90 | 84 | 62 | 80 | 73 | 80 | 78.59261297 | 77.64738835 | 0.987973111 | 0.920366479 | unchanged |
| hsa_circ_100457 | 72 | 75 | 96 | 77 | 88 | 80 | 81.14321447 | 81.68667994 | 1.006697608 | 0.951222141 | unchanged |
| hsa_circ_100459 | 155 | 105 | 180 | 156 | 160 | 161 | 146.7603564 | 159.093378 | 1.084035103 | 0.602917757 | unchanged |
| hsa_circ_100460 | 71 | 68 | 58 | 71 | 52 | 67 | 65.47644259 | 63.1473719 | 0.964428876 | 0.75626642 | unchanged |
| hsa_circ_100462 | 79 | 70 | 152 | 77 | 75 | 80 | 100.5060583 | 77.19062799 | 0.768019653 | 0.42080824 | unchanged |
| hsa_circ_100463 | 153 | 152 | 228 | 168 | 287 | 288 | 177.5916183 | 247.5029558 | 1.393663497 | 0.211520525 | unchanged |
| hsa_circ_100464 | 249 | 240 | 411 | 259 | 245 | 245 | 299.8464515 | 249.7684489 | 0.832987843 | 0.421780583 | unchanged |
| hsa_circ_100465 | 145 | 75 | 115 | 156 | 144 | 132 | 111.5222038 | 144.0340848 | 1.291528323 | 0.202366219 | unchanged |
| hsa_circ_100466 | 643 | 406 | 487 | 464 | 285 | 290 | 511.7483965 | 346.386002 | 0.676867782 | 0.14340119 | unchanged |
| hsa_circ_100468 | 213 | 178 | 160 | 199 | 151 | 152 | 183.5700946 | 167.7138627 | 0.913623012 | 0.512167192 | unchanged |
| hsa_circ_100469 | 144 | 137 | 178 | 119 | 179 | 178 | 153.0206301 | 158.3719287 | 1.034971093 | 0.829973311 | unchanged |
| hsa_circ_100470 | 653 | 354 | 1444 | 568 | 315 | 411 | 817.0268996 | 431.35675 | 0.527959055 | 0.311749352 | unchanged |
| hsa_circ_100472 | 67 | 118 | 87 | 59 | 76 | 82 | 90.35345178 | 72.33226436 | 0.800547881 | 0.334263023 | unchanged |
| hsa_circ_100473 | 299 | 404 | 409 | 320 | 384 | 394 | 370.7647483 | 365.9889728 | 0.987119122 | 0.916542418 | unchanged |
| hsa_circ_100475 | 135 | 129 | 90 | 132 | 98 | 96 | 118.0296227 | 108.2388849 | 0.91704847 | 0.620460733 | unchanged |
| hsa_circ_100478 | 443 | 679 | 616 | 430 | 729 | 782 | 579.2527541 | 647.0705759 | 1.117078117 | 0.630313671 | unchanged |
| hsa_circ_100479 | 224 | 142 | 195 | 253 | 277 | 272 | 186.9670794 | 267.2902441 | 1.429611272 | 0.033322292 | unchanged |
| hsa_circ_100483 | 106 | 167 | 138 | 100 | 119 | 125 | 136.8335087 | 114.4135353 | 0.83615144 | 0.308805827 | unchanged |
| hsa_circ_100486 | 65 | 54 | 61 | 67 | 56 | 58 | 59.92977953 | 60.49147149 | 1.009372502 | 0.910774381 | unchanged |
| hsa_circ_100487 | 63 | 74 | 57 | 61 | 59 | 57 | 64.7133332 | 59.15524446 | 0.914112155 | 0.322950138 | unchanged |
| hsa_circ_100488 | 147 | 265 | 216 | 155 | 234 | 243 | 208.9165293 | 210.740928 | 1.008732668 | 0.969025799 | unchanged |
| hsa_circ_100489 | 73 | 66 | 61 | 60 | 68 | 64 | 66.58009509 | 63.98499825 | 0.961022933 | 0.576445882 | unchanged |
| hsa_circ_100490 | 74 | 60 | 67 | 64 | 65 | 60 | 66.81891091 | 63.12385621 | 0.944700465 | 0.437847454 | unchanged |
| hsa_circ_100491 | 119 | 271 | 257 | 138 | 313 | 232 | 215.645171 | 227.873928 | 1.056707771 | 0.869990359 | unchanged |
| hsa_circ_100492 | 5891 | 8488 | 8717 | 6430 | 7323 | 7657 | 7698.85346 | 7136.538629 | 0.926961224 | 0.595860497 | unchanged |
| hsa_circ_100493 | 151 | 110 | 184 | 187 | 279 | 280 | 148.2892362 | 248.6616442 | 1.676869141 | 0.05581941 | unchanged |
| hsa_circ_100494 | 202 | 162 | 221 | 216 | 302 | 280 | 195.0416909 | 266.0599725 | 1.364118468 | 0.085990054 | unchanged |
| hsa_circ_100496 | 56 | 86 | 71 | 58 | 76 | 72 | 71.28365663 | 68.65917234 | 0.963182524 | 0.812054427 | unchanged |
| hsa_circ_100497 | 115 | 138 | 257 | 102 | 124 | 110 | 169.9889099 | 112.1512523 | 0.659756289 | 0.263957225 | unchanged |
| hsa_circ_100498 | 1482 | 1851 | 1586 | 1660 | 1664 | 1736 | 1639.901408 | 1686.672001 | 1.028520369 | 0.698952217 | unchanged |
| hsa_circ_100499 | 1387 | 854 | 951 | 1913 | 1283 | 1217 | 1064.261447 | 1471.024149 | 1.382201858 | 0.214233372 | unchanged |
| hsa_circ_100500 | 161 | 102 | 157 | 162 | 163 | 173 | 139.8491888 | 166.2112353 | 1.188503393 | 0.243898202 | unchanged |
| hsa_circ_100501 | 84 | 95 | 235 | 96 | 117 | 116 | 137.8736525 | 109.5249956 | 0.794386698 | 0.595531567 | unchanged |
| hsa_circ_100502 | 69 | 76 | 56 | 72 | 60 | 56 | 66.73961265 | 62.92519294 | 0.942846241 | 0.648275981 | unchanged |
| hsa_circ_100503 | 121 | 91 | 114 | 97 | 140 | 134 | 108.4546669 | 123.6584748 | 1.140185834 | 0.398277591 | unchanged |
| hsa_circ_100504 | 94 | 70 | 95 | 87 | 93 | 82 | 86.09137968 | 87.37610611 | 1.014922823 | 0.889384226 | unchanged |
| hsa_circ_100505 | 114 | 77 | 121 | 125 | 82 | 82 | 103.8640253 | 96.24772655 | 0.926670483 | 0.720455307 | unchanged |
| hsa_circ_100506 | 74 | 46 | 68 | 70 | 72 | 72 | 62.45353888 | 71.3393117 | 1.142278132 | 0.35858071 | unchanged |
| hsa_circ_100507 | 2851 | 3021 | 2274 | 2695 | 2516 | 2631 | 2715.42589 | 2613.885772 | 0.962606191 | 0.684166231 | unchanged |
| hsa_circ_100508 | 33200 | 36135 | 37231 | 28508 | 35630 | 35876 | 35522.19342 | 33338.16676 | 0.93851656 | 0.463821812 | unchanged |
| hsa_circ_100510 | 513 | 532 | 465 | 580 | 578 | 539 | 503.4833575 | 565.7735544 | 1.123718482 | 0.061650405 | unchanged |
| hsa_circ_100511 | 152 | 126 | 178 | 181 | 249 | 259 | 151.7113614 | 229.4579049 | 1.512463554 | 0.053441412 | unchanged |
| hsa_circ_100513 | 97 | 90 | 83 | 99 | 77 | 81 | 89.80290496 | 85.83248821 | 0.955787435 | 0.643930292 | unchanged |
| hsa_circ_100514 | 107 | 72 | 98 | 93 | 92 | 105 | 92.32587995 | 96.58638815 | 1.046146413 | 0.726705141 | unchanged |
| hsa_circ_100516 | 119 | 81 | 204 | 111 | 101 | 101 | 135.0910543 | 104.6112976 | 0.774376202 | 0.451066547 | unchanged |
| hsa_circ_100517 | 310 | 309 | 339 | 286 | 312 | 280 | 319.3604433 | 292.8575403 | 0.917012568 | 0.130834812 | unchanged |
| hsa_circ_100521 | 131 | 126 | 115 | 131 | 127 | 112 | 124.0194505 | 123.3464291 | 0.99457326 | 0.934926516 | unchanged |
| hsa_circ_100522 | 71 | 102 | 72 | 71 | 62 | 68 | 81.65392241 | 67.00482173 | 0.82059526 | 0.227439472 | unchanged |
| hsa_circ_100523 | 71 | 58 | 66 | 77 | 78 | 74 | 65.09367015 | 76.14118014 | 1.169717116 | 0.044366965 | unchanged |
| hsa_circ_100524 | 64 | 117 | 118 | 66 | 60 | 52 | 99.55274711 | 59.33541546 | 0.596019871 | 0.095245964 | unchanged |
| hsa_circ_100525 | 739 | 1089 | 917 | 753 | 1154 | 1147 | 914.8321053 | 1018.140341 | 1.112925896 | 0.568953357 | unchanged |
| hsa_circ_100526 | 125 | 95 | 114 | 120 | 112 | 112 | 111.3323687 | 114.7358132 | 1.030570125 | 0.724512114 | unchanged |
| hsa_circ_100527 | 172 | 135 | 128 | 150 | 111 | 115 | 144.8271018 | 125.3396915 | 0.865443622 | 0.351037516 | unchanged |
| hsa_circ_100528 | 387 | 336 | 308 | 464 | 405 | 364 | 343.6734507 | 410.7660095 | 1.195221827 | 0.144075096 | unchanged |
| hsa_circ_100529 | 198 | 173 | 139 | 248 | 196 | 211 | 169.8412981 | 218.2674513 | 1.285125901 | 0.103976814 | unchanged |
| hsa_circ_100530 | 209 | 199 | 144 | 240 | 226 | 227 | 184.0792563 | 230.9115684 | 1.254413849 | 0.089323198 | unchanged |
| hsa_circ_100531 | 169 | 178 | 212 | 176 | 288 | 288 | 186.2636232 | 250.7482762 | 1.346201002 | 0.177196858 | unchanged |
| hsa_circ_100532 | 60 | 146 | 60 | 55 | 51 | 58 | 89.0406094 | 54.67670404 | 0.6140648 | 0.298162086 | unchanged |
| hsa_circ_100533 | 2876 | 5982 | 4877 | 4046 | 5944 | 6271 | 4578.416456 | 5420.337222 | 1.183889075 | 0.502359143 | unchanged |
| hsa_circ_100534 | 423 | 185 | 188 | 363 | 178 | 183 | 265.3634724 | 241.3648677 | 0.90956327 | 0.821395219 | unchanged |
| hsa_circ_100536 | 116 | 87 | 164 | 114 | 115 | 116 | 122.5122093 | 114.8088902 | 0.937122029 | 0.748296764 | unchanged |
| hsa_circ_100537 | 160 | 90 | 128 | 152 | 138 | 142 | 126.0484057 | 144.2889887 | 1.144710938 | 0.425564384 | unchanged |
| hsa_circ_100538 | 64 | 74 | 74 | 62 | 73 | 75 | 70.82329404 | 69.96025662 | 0.987814215 | 0.879489674 | unchanged |
| hsa_circ_100539 | 68 | 57 | 74 | 69 | 81 | 76 | 66.21322255 | 75.33539669 | 1.137769675 | 0.20468505 | unchanged |
| hsa_circ_100540 | 70 | 61 | 51 | 65 | 57 | 55 | 60.55414737 | 58.95963257 | 0.97366795 | 0.813513921 | unchanged |
| hsa_circ_100541 | 3157 | 4124 | 2946 | 3664 | 3258 | 3126 | 3409.051014 | 3349.479212 | 0.9825254 | 0.888068071 | unchanged |
| hsa_circ_100542 | 1268 | 927 | 589 | 1194 | 842 | 720 | 928.1211916 | 918.9214593 | 0.99008779 | 0.97151785 | unchanged |
| hsa_circ_100544 | 329 | 370 | 224 | 320 | 260 | 255 | 307.4269651 | 278.4402925 | 0.905712004 | 0.580493667 | unchanged |
| hsa_circ_100545 | 62 | 142 | 55 | 57 | 56 | 54 | 86.3078924 | 55.82404827 | 0.646801199 | 0.334447598 | unchanged |
| hsa_circ_100546 | 84 | 69 | 160 | 87 | 89 | 90 | 104.1730458 | 88.57237626 | 0.850242743 | 0.609058136 | unchanged |
| hsa_circ_100548 | 87 | 121 | 101 | 115 | 117 | 121 | 103.079435 | 117.4542103 | 1.139453377 | 0.219956635 | unchanged |
| hsa_circ_100550 | 98 | 100 | 106 | 104 | 114 | 125 | 100.9870001 | 114.6334657 | 1.135130913 | 0.100530923 | unchanged |
| hsa_circ_100551 | 59 | 66 | 59 | 66 | 68 | 70 | 61.37429038 | 67.81729248 | 1.104978845 | 0.067108267 | unchanged |
| hsa_circ_100552 | 129 | 174 | 135 | 147 | 140 | 187 | 145.8829205 | 158.237409 | 1.084687697 | 0.577862949 | unchanged |
| hsa_circ_100553 | 176 | 228 | 467 | 178 | 253 | 234 | 290.0863931 | 221.6378389 | 0.764040797 | 0.499895662 | unchanged |
| hsa_circ_100554 | 104 | 77 | 174 | 96 | 80 | 73 | 118.4442824 | 82.82611541 | 0.699283357 | 0.298787571 | unchanged |
| hsa_circ_100556 | 73 | 81 | 69 | 74 | 66 | 67 | 74.36426673 | 69.27643516 | 0.931582307 | 0.293937822 | unchanged |
| hsa_circ_100559 | 135 | 116 | 131 | 152 | 153 | 139 | 127.4628587 | 147.9894671 | 1.161039918 | 0.04680247 | unchanged |
| hsa_circ_100560 | 86 | 61 | 81 | 85 | 78 | 78 | 76.08294584 | 80.09318227 | 1.052708743 | 0.635093965 | unchanged |
| hsa_circ_100561 | 91 | 85 | 85 | 87 | 91 | 87 | 87.02008887 | 88.00682372 | 1.011339162 | 0.707694355 | unchanged |
| hsa_circ_100562 | 1346 | 1327 | 1023 | 1559 | 1108 | 1128 | 1232.334044 | 1264.777069 | 1.026326485 | 0.86615678 | unchanged |
| hsa_circ_100563 | 124 | 84 | 100 | 128 | 123 | 113 | 102.5540933 | 121.1458905 | 1.181287714 | 0.20817019 | unchanged |
| hsa_circ_100564 | 65 | 52 | 67 | 65 | 72 | 76 | 61.12255475 | 70.70519925 | 1.156777552 | 0.170408218 | unchanged |
| hsa_circ_100565 | 1027 | 914 | 635 | 915 | 566 | 659 | 858.3276261 | 713.3116515 | 0.831048227 | 0.405737336 | unchanged |
| hsa_circ_100566 | 230 | 295 | 276 | 258 | 294 | 286 | 266.6907675 | 279.3547871 | 1.047485782 | 0.598688918 | unchanged |
| hsa_circ_100568 | 171 | 103 | 123 | 185 | 149 | 155 | 132.266968 | 162.8036498 | 1.230871565 | 0.255027693 | unchanged |
| hsa_circ_100570 | 407 | 426 | 291 | 350 | 336 | 331 | 374.3379633 | 338.8196844 | 0.90511708 | 0.449687226 | unchanged |
| hsa_circ_100571 | 1798 | 1513 | 1240 | 2145 | 1587 | 1520 | 1516.754489 | 1750.47741 | 1.154094102 | 0.411671884 | unchanged |
| hsa_circ_100572 | 81 | 54 | 110 | 76 | 117 | 115 | 81.84507572 | 102.5557914 | 1.253047792 | 0.379765083 | unchanged |
| hsa_circ_100573 | 134 | 95 | 117 | 127 | 122 | 133 | 115.3809852 | 127.4638381 | 1.104721354 | 0.365736685 | unchanged |
| hsa_circ_100576 | 72 | 64 | 75 | 72 | 77 | 78 | 70.31431882 | 75.59645334 | 1.075121748 | 0.236491284 | unchanged |
| hsa_circ_100578 | 61 | 82 | 61 | 56 | 57 | 59 | 67.85315088 | 57.22362361 | 0.843345119 | 0.198521232 | unchanged |
| hsa_circ_100579 | 386 | 495 | 287 | 582 | 323 | 284 | 389.4417948 | 396.4173947 | 1.017911791 | 0.952980531 | unchanged |
| hsa_circ_100580 | 145 | 166 | 165 | 163 | 179 | 191 | 158.6423919 | 177.789283 | 1.120692149 | 0.146649577 | unchanged |
| hsa_circ_100582 | 70 | 71 | 62 | 60 | 65 | 65 | 67.85328884 | 63.50040837 | 0.935848644 | 0.287951245 | unchanged |
| hsa_circ_100583 | 190 | 147 | 202 | 213 | 231 | 221 | 179.8480928 | 221.7863816 | 1.233187287 | 0.074646922 | unchanged |
| hsa_circ_100585 | 139 | 144 | 121 | 160 | 133 | 123 | 134.612461 | 138.6597115 | 1.030065943 | 0.773320536 | unchanged |
| hsa_circ_100587 | 3921 | 4561 | 2443 | 4283 | 3317 | 2981 | 3641.737207 | 3527.183887 | 0.96854432 | 0.884247549 | unchanged |
| hsa_circ_100588 | 133 | 123 | 101 | 133 | 86 | 92 | 118.7903086 | 103.7413863 | 0.873315235 | 0.442258368 | unchanged |
| hsa_circ_100589 | 105 | 69 | 93 | 103 | 108 | 110 | 88.8019589 | 106.7210152 | 1.201786723 | 0.170222996 | unchanged |
| hsa_circ_100590 | 71 | 93 | 68 | 79 | 72 | 74 | 77.38917687 | 75.02717955 | 0.969478971 | 0.79171997 | unchanged |
| hsa_circ_100593 | 1292 | 2232 | 2256 | 1514 | 2566 | 2457 | 1926.570395 | 2178.889448 | 1.130967991 | 0.613081411 | unchanged |
| hsa_circ_100595 | 92 | 73 | 78 | 86 | 74 | 73 | 80.9538074 | 77.50285972 | 0.957371397 | 0.652450592 | unchanged |
| hsa_circ_100597 | 126 | 136 | 404 | 133 | 188 | 186 | 222.0869759 | 168.8588383 | 0.760327514 | 0.596503307 | unchanged |
| hsa_circ_100598 | 68 | 63 | 120 | 67 | 130 | 61 | 83.4401874 | 86.1951539 | 1.033017262 | 0.92767414 | unchanged |
| hsa_circ_100599 | 82 | 81 | 113 | 74 | 110 | 109 | 91.85314357 | 97.4137786 | 1.06053832 | 0.742348847 | unchanged |
| hsa_circ_100600 | 392 | 513 | 474 | 492 | 761 | 695 | 459.7848509 | 649.2161301 | 1.411999827 | 0.09955525 | unchanged |
| hsa_circ_100601 | 359 | 407 | 421 | 344 | 436 | 416 | 395.5368461 | 398.4709954 | 1.007418144 | 0.934493255 | unchanged |
| hsa_circ_100602 | 209 | 94 | 130 | 105 | 74 | 76 | 144.5007199 | 84.80237752 | 0.586864741 | 0.168352886 | unchanged |
| hsa_circ_100604 | 5410 | 2480 | 1603 | 7731 | 1972 | 2179 | 3164.394416 | 3960.601653 | 1.251614411 | 0.736828799 | unchanged |
| hsa_circ_100605 | 113 | 95 | 151 | 130 | 182 | 182 | 119.7611314 | 164.7368485 | 1.375545193 | 0.134750932 | unchanged |
| hsa_circ_100606 | 2550 | 1340 | 1371 | 1537 | 1258 | 1245 | 1753.756035 | 1346.894219 | 0.768005465 | 0.376488227 | unchanged |
| hsa_circ_100607 | 124 | 113 | 146 | 109 | 173 | 150 | 127.9415169 | 143.8381899 | 1.124249527 | 0.493527585 | unchanged |
| hsa_circ_100609 | 420 | 323 | 380 | 399 | 456 | 441 | 374.4486651 | 432.3191539 | 1.154548525 | 0.15579799 | unchanged |
| hsa_circ_100610 | 199 | 266 | 300 | 228 | 339 | 335 | 255.0631305 | 300.7865642 | 1.179263203 | 0.385453003 | unchanged |
| hsa_circ_100611 | 224 | 263 | 260 | 226 | 280 | 307 | 248.9243376 | 270.9961557 | 1.088668783 | 0.456380209 | unchanged |
| hsa_circ_100613 | 420 | 524 | 453 | 448 | 408 | 409 | 465.6260718 | 421.5265159 | 0.905289762 | 0.260054115 | unchanged |
| hsa_circ_100614 | 192 | 252 | 261 | 211 | 303 | 304 | 235.1756806 | 272.8855823 | 1.160347794 | 0.374630765 | unchanged |
| hsa_circ_100615 | 892 | 985 | 972 | 908 | 683 | 824 | 949.7744595 | 805.0662865 | 0.847639435 | 0.113574172 | unchanged |
| hsa_circ_100616 | 154 | 257 | 246 | 148 | 199 | 205 | 218.8905569 | 184.3504035 | 0.842203548 | 0.408235366 | unchanged |
| hsa_circ_100617 | 148 | 162 | 75 | 135 | 80 | 85 | 128.4947663 | 100.4026272 | 0.781375227 | 0.432243414 | unchanged |
| hsa_circ_100618 | 87 | 75 | 89 | 98 | 84 | 84 | 83.5819656 | 88.99100274 | 1.064715362 | 0.452657268 | unchanged |
| hsa_circ_100620 | 1760 | 1638 | 2536 | 2187 | 2576 | 2529 | 1977.931467 | 2430.77121 | 1.228946124 | 0.213933948 | unchanged |
| hsa_circ_100621 | 433 | 650 | 695 | 278 | 625 | 601 | 592.6606681 | 501.0444702 | 0.845415424 | 0.543016104 | unchanged |
| hsa_circ_100623 | 3992 | 4128 | 2660 | 4079 | 3519 | 3388 | 3593.369271 | 3661.838114 | 1.019054218 | 0.900432922 | unchanged |
| hsa_circ_100625 | 283 | 357 | 377 | 365 | 537 | 575 | 339.108244 | 492.4000922 | 1.452044003 | 0.095642636 | unchanged |
| hsa_circ_100626 | 190 | 221 | 587 | 236 | 466 | 454 | 332.6167563 | 385.397812 | 1.158684296 | 0.739059829 | unchanged |
| hsa_circ_100627 | 95 | 94 | 79 | 91 | 79 | 94 | 89.50304242 | 87.9694809 | 0.982865817 | 0.832850864 | unchanged |
| hsa_circ_100628 | 61 | 62 | 68 | 61 | 68 | 66 | 63.34371182 | 64.96065762 | 1.02552654 | 0.598206088 | unchanged |
| hsa_circ_100631 | 103 | 101 | 242 | 83 | 83 | 83 | 148.5745801 | 83.27442868 | 0.560489073 | 0.234139319 | unchanged |
| hsa_circ_100633 | 1197 | 1387 | 1630 | 1040 | 1883 | 2071 | 1404.748938 | 1664.599109 | 1.184979795 | 0.488261538 | unchanged |
| hsa_circ_100634 | 309 | 376 | 300 | 305 | 314 | 309 | 328.0457069 | 309.4151632 | 0.943207476 | 0.481330319 | unchanged |
| hsa_circ_100635 | 734 | 570 | 554 | 540 | 444 | 453 | 619.3310896 | 479.0975616 | 0.7735726 | 0.098009885 | unchanged |
| hsa_circ_100636 | 132 | 93 | 108 | 122 | 113 | 111 | 110.811941 | 115.2630652 | 1.040168272 | 0.722574772 | unchanged |
| hsa_circ_100637 | 301 | 216 | 244 | 366 | 172 | 166 | 253.5410936 | 234.6447792 | 0.925470407 | 0.801230172 | unchanged |
| hsa_circ_100638 | 76 | 57 | 85 | 66 | 81 | 79 | 72.80069409 | 75.41026568 | 1.035845422 | 0.798971943 | unchanged |
| hsa_circ_100640 | 605 | 587 | 462 | 628 | 420 | 427 | 551.3804315 | 491.5464774 | 0.891483356 | 0.504420356 | unchanged |
| hsa_circ_100641 | 2037 | 1947 | 1798 | 2172 | 2110 | 1977 | 1927.20052 | 2086.237752 | 1.08252241 | 0.152820937 | unchanged |
| hsa_circ_100642 | 79 | 68 | 110 | 75 | 109 | 112 | 85.560774 | 98.35250488 | 1.149504619 | 0.498682192 | unchanged |
| hsa_circ_100643 | 65 | 50 | 55 | 72 | 56 | 55 | 56.39683583 | 61.20153635 | 1.085194505 | 0.535332045 | unchanged |
| hsa_circ_100644 | 282 | 290 | 328 | 262 | 358 | 368 | 299.8793599 | 329.4295401 | 1.098540227 | 0.464538092 | unchanged |
| hsa_circ_100646 | 9525 | 7188 | 7134 | 11208 | 9329 | 9557 | 7948.769154 | 10031.56084 | 1.262026943 | 0.102186453 | unchanged |
| hsa_circ_100647 | 266 | 58 | 74 | 155 | 77 | 73 | 132.5291991 | 101.8931855 | 0.768835745 | 0.693315164 | unchanged |
| hsa_circ_100649 | 93 | 71 | 62 | 89 | 61 | 62 | 75.29810832 | 70.56717399 | 0.937170608 | 0.734551638 | unchanged |
| hsa_circ_100650 | 137 | 150 | 141 | 148 | 167 | 165 | 142.5303116 | 160.1011252 | 1.123277732 | 0.071169164 | unchanged |
| hsa_circ_100651 | 240 | 184 | 148 | 221 | 198 | 217 | 190.840441 | 211.7942034 | 1.109797286 | 0.491607668 | unchanged |
| hsa_circ_100652 | 91 | 106 | 64 | 89 | 67 | 69 | 86.94755077 | 74.90405799 | 0.861485543 | 0.435760229 | unchanged |
| hsa_circ_100653 | 163 | 192 | 221 | 217 | 273 | 272 | 191.9487228 | 253.8255917 | 1.322361451 | 0.069340974 | unchanged |
| hsa_circ_100654 | 64 | 146 | 90 | 67 | 101 | 89 | 100.3994362 | 85.52250491 | 0.851822561 | 0.60077807 | unchanged |
| hsa_circ_100655 | 418 | 613 | 482 | 439 | 559 | 560 | 504.3018099 | 519.2751917 | 1.029691311 | 0.840646422 | unchanged |
| hsa_circ_100656 | 287 | 437 | 443 | 243 | 351 | 295 | 388.8465019 | 296.3574245 | 0.762145019 | 0.197229914 | unchanged |
| hsa_circ_100659 | 470 | 539 | 564 | 537 | 824 | 909 | 524.5228664 | 756.6341167 | 1.44251884 | 0.116190752 | unchanged |
| hsa_circ_100660 | 234 | 108 | 150 | 196 | 125 | 134 | 163.9845658 | 151.6337305 | 0.92468294 | 0.789924642 | unchanged |
| hsa_circ_100661 | 177 | 134 | 172 | 152 | 208 | 211 | 161.1065562 | 190.0912779 | 1.179910256 | 0.284141416 | unchanged |
| hsa_circ_100662 | 82 | 71 | 73 | 81 | 68 | 63 | 75.40419215 | 70.60365759 | 0.936335973 | 0.486764222 | unchanged |
| hsa_circ_100664 | 104 | 113 | 135 | 98 | 123 | 120 | 117.4667757 | 113.9714898 | 0.970244472 | 0.785001257 | unchanged |
| hsa_circ_100665 | 312 | 272 | 297 | 252 | 281 | 276 | 293.5348765 | 269.5955472 | 0.918444685 | 0.181812403 | unchanged |
| hsa_circ_100667 | 126 | 231 | 133 | 127 | 139 | 142 | 163.385673 | 136.2084907 | 0.833662391 | 0.471914216 | unchanged |
| hsa_circ_100668 | 140 | 106 | 224 | 170 | 299 | 269 | 156.7262869 | 245.9253468 | 1.569139112 | 0.164350343 | unchanged |
| hsa_circ_100669 | 211 | 155 | 216 | 242 | 247 | 253 | 194.3283956 | 247.6864997 | 1.274576981 | 0.055262819 | unchanged |
| hsa_circ_100670 | 267 | 286 | 406 | 291 | 310 | 295 | 319.7947384 | 298.5816871 | 0.933666666 | 0.654673204 | unchanged |
| hsa_circ_100671 | 225 | 129 | 190 | 205 | 235 | 226 | 181.2024616 | 222.2739515 | 1.226660772 | 0.235821654 | unchanged |
| hsa_circ_100672 | 105 | 109 | 125 | 103 | 103 | 107 | 113.2126585 | 104.2582244 | 0.92090607 | 0.223785595 | unchanged |
| hsa_circ_100674 | 1935 | 2969 | 3152 | 1976 | 1860 | 2141 | 2685.184754 | 1992.331897 | 0.741971998 | 0.14840804 | unchanged |
| hsa_circ_100675 | 5561 | 6462 | 7247 | 6188 | 8617 | 8446 | 6423.286642 | 7749.934389 | 1.206537217 | 0.22352092 | unchanged |
| hsa_circ_100676 | 268 | 259 | 319 | 229 | 280 | 259 | 281.7128572 | 256.030061 | 0.908833425 | 0.339931289 | unchanged |
| hsa_circ_100677 | 120 | 114 | 163 | 121 | 169 | 166 | 132.4209916 | 151.9355199 | 1.147367332 | 0.422852836 | unchanged |
| hsa_circ_100679 | 168 | 345 | 305 | 183 | 245 | 261 | 272.8796651 | 229.5100066 | 0.841066726 | 0.500051016 | unchanged |
| hsa_circ_100680 | 95 | 57 | 59 | 87 | 70 | 69 | 70.48279964 | 75.266226 | 1.067866577 | 0.744193932 | unchanged |
| hsa_circ_100684 | 1308 | 1244 | 937 | 1411 | 1310 | 1320 | 1162.95286 | 1347.042149 | 1.158294713 | 0.196309946 | unchanged |
| hsa_circ_100685 | 4603 | 4722 | 4426 | 4694 | 5614 | 5271 | 4583.876733 | 5192.990576 | 1.132881811 | 0.096806268 | unchanged |
| hsa_circ_100687 | 339 | 147 | 214 | 335 | 182 | 189 | 233.1163885 | 235.2398085 | 1.009108841 | 0.978812574 | unchanged |
| hsa_circ_100688 | 446 | 242 | 255 | 435 | 318 | 293 | 314.403881 | 348.590791 | 1.108735649 | 0.687971104 | unchanged |
| hsa_circ_100689 | 72 | 68 | 74 | 74 | 87 | 86 | 71.3799574 | 82.2243701 | 1.151925178 | 0.075972869 | unchanged |
| hsa_circ_100690 | 69 | 96 | 73 | 66 | 69 | 78 | 79.30839903 | 71.17917125 | 0.897498526 | 0.423517428 | unchanged |
| hsa_circ_100691 | 890 | 2507 | 1989 | 881 | 1660 | 1614 | 1795.443608 | 1384.99088 | 0.771392025 | 0.489099101 | unchanged |
| hsa_circ_100693 | 836 | 732 | 1077 | 776 | 937 | 935 | 881.7028806 | 882.661224 | 1.001086923 | 0.99374707 | unchanged |
| hsa_circ_100694 | 280 | 272 | 216 | 338 | 283 | 264 | 255.8949287 | 294.8174112 | 1.152103376 | 0.260426467 | unchanged |
| hsa_circ_100695 | 159 | 217 | 224 | 196 | 248 | 249 | 199.8351121 | 230.99917 | 1.15594886 | 0.316236204 | unchanged |
| hsa_circ_100696 | 29456 | 23696 | 20962 | 24911 | 21608 | 22372 | 24704.58989 | 22963.48447 | 0.929522999 | 0.553486229 | unchanged |
| hsa_circ_100697 | 228 | 191 | 167 | 233 | 196 | 182 | 195.7059499 | 203.5309985 | 1.039983703 | 0.754493199 | unchanged |
| hsa_circ_100698 | 106 | 126 | 91 | 104 | 94 | 97 | 107.8974916 | 98.72386516 | 0.914978316 | 0.441502233 | unchanged |
| hsa_circ_100699 | 65 | 94 | 66 | 71 | 72 | 70 | 75.15183544 | 70.80791224 | 0.942198043 | 0.666782491 | unchanged |
| hsa_circ_100700 | 113 | 107 | 131 | 112 | 122 | 117 | 116.933162 | 117.1248596 | 1.001639377 | 0.981591446 | unchanged |
| hsa_circ_100701 | 67 | 67 | 68 | 70 | 138 | 75 | 67.26109424 | 94.11912844 | 1.399310099 | 0.286223969 | unchanged |
| hsa_circ_100702 | 247 | 136 | 151 | 192 | 165 | 166 | 177.7771234 | 174.1753396 | 0.979739892 | 0.925119434 | unchanged |
| hsa_circ_100706 | 306 | 370 | 266 | 262 | 219 | 220 | 314.0465494 | 233.6801305 | 0.744093928 | 0.074270527 | unchanged |
| hsa_circ_100707 | 241 | 389 | 356 | 218 | 318 | 300 | 328.4112982 | 278.7395376 | 0.848751365 | 0.413403212 | unchanged |
| hsa_circ_100708 | 71 | 99 | 70 | 62 | 68 | 75 | 79.79483267 | 68.2979945 | 0.855920017 | 0.324996377 | unchanged |
| hsa_circ_100709 | 106 | 184 | 109 | 110 | 100 | 95 | 133.0318681 | 101.4367273 | 0.762499458 | 0.291596779 | unchanged |
| hsa_circ_100710 | 73 | 97 | 82 | 74 | 86 | 94 | 84.0258932 | 84.4131777 | 1.004609109 | 0.967522835 | unchanged |
| hsa_circ_100711 | 84 | 99 | 83 | 91 | 76 | 89 | 88.59570943 | 85.19323393 | 0.961595482 | 0.637208701 | unchanged |
| hsa_circ_100712 | 229 | 215 | 185 | 198 | 192 | 201 | 209.6873658 | 197.0970263 | 0.939956614 | 0.400968877 | unchanged |
| hsa_circ_100713 | 645 | 749 | 788 | 701 | 1014 | 1010 | 727.2222509 | 908.381815 | 1.24911169 | 0.181457067 | unchanged |
| hsa_circ_100714 | 1599 | 2095 | 1903 | 1712 | 2381 | 2220 | 1865.607972 | 2104.412107 | 1.128003385 | 0.39010266 | unchanged |
| hsa_circ_100715 | 132 | 144 | 121 | 129 | 168 | 165 | 132.3254923 | 153.6676769 | 1.161285511 | 0.202729536 | unchanged |
| hsa_circ_100716 | 89 | 66 | 96 | 92 | 121 | 120 | 83.27575356 | 110.7090067 | 1.329426657 | 0.105798818 | unchanged |
| hsa_circ_100718 | 388 | 226 | 212 | 378 | 263 | 247 | 275.3548896 | 295.933525 | 1.074734955 | 0.783447213 | unchanged |
| hsa_circ_100719 | 318 | 172 | 100 | 282 | 107 | 79 | 196.8384041 | 155.942847 | 0.792237916 | 0.674264058 | unchanged |
| hsa_circ_100720 | 1419 | 957 | 815 | 1026 | 760 | 763 | 1063.677468 | 849.9487633 | 0.799066248 | 0.351201812 | unchanged |
| hsa_circ_100721 | 235 | 130 | 138 | 207 | 159 | 153 | 167.5116875 | 173.0634413 | 1.033142486 | 0.8906199 | unchanged |
| hsa_circ_100722 | 75 | 76 | 62 | 79 | 68 | 70 | 71.28078894 | 72.38360696 | 1.015471462 | 0.854726469 | unchanged |
| hsa_circ_100723 | 282 | 180 | 187 | 233 | 228 | 228 | 216.355526 | 229.604479 | 1.061236952 | 0.708585942 | unchanged |
| hsa_circ_100725 | 85 | 80 | 85 | 91 | 82 | 82 | 83.05524116 | 85.04348445 | 1.023938806 | 0.612148213 | unchanged |
| hsa_circ_100726 | 1103 | 2284 | 2385 | 858 | 1666 | 1629 | 1923.975478 | 1384.245128 | 0.719471294 | 0.331091434 | unchanged |
| hsa_circ_100727 | 75 | 73 | 73 | 74 | 83 | 77 | 73.82488926 | 78.20108433 | 1.059278045 | 0.183425847 | unchanged |
| hsa_circ_100728 | 197 | 166 | 148 | 197 | 157 | 154 | 170.2650465 | 169.0710748 | 0.99298757 | 0.954647433 | unchanged |
| hsa_circ_100729 | 93 | 86 | 184 | 69 | 99 | 115 | 120.8065476 | 94.22136663 | 0.779935926 | 0.482477451 | unchanged |
| hsa_circ_100732 | 438 | 673 | 593 | 425 | 622 | 633 | 568.1894208 | 559.8707825 | 0.985359393 | 0.935515747 | unchanged |
| hsa_circ_100733 | 65 | 95 | 61 | 59 | 62 | 64 | 73.62288539 | 61.64655613 | 0.837328717 | 0.32026307 | unchanged |
| hsa_circ_100734 | 1000 | 1236 | 948 | 971 | 1183 | 1172 | 1061.205403 | 1108.732984 | 1.04478641 | 0.693295262 | unchanged |
| hsa_circ_100735 | 119 | 158 | 203 | 146 | 179 | 212 | 160.0529269 | 179.1791957 | 1.11949965 | 0.567960317 | unchanged |
| hsa_circ_100736 | 118 | 168 | 83 | 113 | 81 | 87 | 123.0754041 | 93.41037838 | 0.758968691 | 0.329065639 | unchanged |
| hsa_circ_100737 | 508 | 812 | 918 | 724 | 927 | 918 | 745.9074365 | 856.3949141 | 1.148124918 | 0.472856502 | unchanged |
| hsa_circ_100738 | 94 | 142 | 156 | 156 | 174 | 173 | 130.2813951 | 167.9794686 | 1.289358841 | 0.127292399 | unchanged |
| hsa_circ_100739 | 98 | 89 | 136 | 123 | 132 | 136 | 107.7405044 | 130.45199 | 1.210798025 | 0.204972219 | unchanged |
| hsa_circ_100740 | 573 | 609 | 774 | 666 | 804 | 921 | 651.810957 | 797.170183 | 1.223008258 | 0.205706942 | unchanged |
| hsa_circ_100741 | 97 | 67 | 100 | 92 | 92 | 90 | 88.16715374 | 91.21830178 | 1.034606403 | 0.787092718 | unchanged |
| hsa_circ_100742 | 74 | 68 | 111 | 71 | 103 | 100 | 84.4330447 | 91.11080059 | 1.079089365 | 0.709689972 | unchanged |
| hsa_circ_100745 | 155 | 147 | 116 | 149 | 112 | 118 | 139.2685868 | 126.0998345 | 0.905443485 | 0.466137887 | unchanged |
| hsa_circ_100746 | 79 | 56 | 63 | 82 | 66 | 70 | 65.84447089 | 72.59296834 | 1.102491483 | 0.467380445 | unchanged |
| hsa_circ_100747 | 66 | 56 | 60 | 68 | 69 | 68 | 60.51742504 | 68.09434578 | 1.125202299 | 0.05887038 | unchanged |
| hsa_circ_100748 | 1097 | 682 | 732 | 890 | 666 | 679 | 837.1589847 | 744.7519776 | 0.88961833 | 0.570222186 | unchanged |
| hsa_circ_100749 | 72 | 95 | 48 | 75 | 59 | 63 | 71.75610011 | 65.47693376 | 0.912492926 | 0.684141456 | unchanged |
| hsa_circ_100750 | 8043 | 7312 | 6791 | 7979 | 7793 | 7667 | 7381.950518 | 7813.080907 | 1.058403316 | 0.313649065 | unchanged |
| hsa_circ_100751 | 9699 | 8519 | 5739 | 8288 | 6322 | 6180 | 7985.645519 | 6929.828814 | 0.867785678 | 0.479874311 | unchanged |
| hsa_circ_100752 | 69 | 78 | 60 | 67 | 62 | 69 | 68.92209153 | 66.21959418 | 0.960789098 | 0.653196391 | unchanged |
| hsa_circ_100753 | 112 | 127 | 91 | 108 | 90 | 87 | 110.3068126 | 94.93614385 | 0.860655308 | 0.286158929 | unchanged |
| hsa_circ_100754 | 856 | 1958 | 1366 | 1164 | 1597 | 1643 | 1393.123885 | 1468.254061 | 1.053929286 | 0.841836604 | unchanged |
| hsa_circ_100755 | 118 | 122 | 117 | 125 | 124 | 134 | 119.2061121 | 127.6396191 | 1.070747271 | 0.065086908 | unchanged |
| hsa_circ_100756 | 409 | 632 | 533 | 468 | 612 | 623 | 524.8357304 | 567.7238241 | 1.081717176 | 0.626244175 | unchanged |
| hsa_circ_100758 | 149 | 110 | 103 | 137 | 103 | 105 | 120.3084215 | 114.9755996 | 0.955673744 | 0.781014362 | unchanged |
| hsa_circ_100761 | 91 | 68 | 87 | 89 | 87 | 88 | 81.98267135 | 88.05898668 | 1.074117069 | 0.441658389 | unchanged |
| hsa_circ_100762 | 88 | 67 | 80 | 88 | 87 | 91 | 78.17959251 | 88.36317332 | 1.130258812 | 0.179062113 | unchanged |
| hsa_circ_100763 | 74 | 65 | 289 | 68 | 91 | 90 | 142.9940932 | 83.20012617 | 0.581843098 | 0.462177762 | unchanged |
| hsa_circ_100765 | 218 | 258 | 252 | 218 | 291 | 288 | 242.5428947 | 265.5139755 | 1.094709354 | 0.441287689 | unchanged |
| hsa_circ_100766 | 54 | 76 | 60 | 52 | 66 | 58 | 62.99114942 | 58.79515277 | 0.93338752 | 0.619600264 | unchanged |
| hsa_circ_100767 | 163 | 182 | 147 | 176 | 157 | 155 | 164.1361418 | 162.675948 | 0.991103765 | 0.910766446 | unchanged |
| hsa_circ_100769 | 63 | 59 | 104 | 58 | 66 | 61 | 75.36178156 | 61.52565 | 0.816403868 | 0.397417016 | unchanged |
| hsa_circ_100770 | 643 | 441 | 197 | 578 | 189 | 180 | 427.1736846 | 315.7513105 | 0.739163769 | 0.577705322 | unchanged |
| hsa_circ_100772 | 357 | 195 | 218 | 326 | 283 | 278 | 256.6299872 | 295.6866576 | 1.152190595 | 0.501245853 | unchanged |
| hsa_circ_100774 | 61 | 78 | 65 | 61 | 71 | 69 | 68.05628926 | 66.85405458 | 0.982334701 | 0.850418155 | unchanged |
| hsa_circ_100775 | 219 | 227 | 243 | 199 | 234 | 214 | 229.6255247 | 215.748166 | 0.939565261 | 0.327478498 | unchanged |
| hsa_circ_100779 | 61 | 49 | 68 | 64 | 73 | 73 | 59.38526474 | 70.10847986 | 1.180570301 | 0.15812963 | unchanged |
| hsa_circ_100780 | 260 | 255 | 200 | 220 | 231 | 233 | 238.3838562 | 228.1107884 | 0.956905354 | 0.627526446 | unchanged |
| hsa_circ_100781 | 66 | 74 | 65 | 62 | 69 | 67 | 68.54680891 | 65.95363927 | 0.96216936 | 0.490769455 | unchanged |
| hsa_circ_100782 | 106 | 79 | 142 | 101 | 164 | 162 | 109.1046205 | 142.2879528 | 1.304142318 | 0.295798194 | unchanged |
| hsa_circ_100783 | 126 | 141 | 121 | 133 | 138 | 156 | 129.339035 | 142.6446497 | 1.102873929 | 0.213484856 | unchanged |
| hsa_circ_100787 | 142 | 206 | 180 | 151 | 203 | 203 | 175.8242829 | 185.6737788 | 1.056018974 | 0.717590851 | unchanged |
| hsa_circ_100788 | 488 | 377 | 248 | 427 | 316 | 321 | 371.2043909 | 354.6980518 | 0.955533018 | 0.843183121 | unchanged |
| hsa_circ_100789 | 93 | 68 | 112 | 86 | 116 | 123 | 90.76235265 | 107.9967212 | 1.18988455 | 0.368536281 | unchanged |
| hsa_circ_100790 | 389 | 230 | 203 | 272 | 341 | 322 | 274.1224096 | 311.5665606 | 1.136596461 | 0.576557391 | unchanged |
| hsa_circ_100793 | 62 | 48 | 57 | 74 | 64 | 58 | 55.67411642 | 65.13959437 | 1.170015773 | 0.201502208 | unchanged |
| hsa_circ_100794 | 68 | 86 | 69 | 76 | 71 | 76 | 74.38264965 | 74.51739385 | 1.0018115 | 0.982668043 | unchanged |
| hsa_circ_100795 | 275 | 158 | 217 | 235 | 169 | 177 | 216.4139785 | 193.917933 | 0.896050867 | 0.600924262 | unchanged |
| hsa_circ_100796 | 91 | 125 | 120 | 99 | 134 | 123 | 112.1727846 | 118.6768137 | 1.057982238 | 0.682195931 | unchanged |
| hsa_circ_100797 | 153 | 169 | 179 | 172 | 194 | 198 | 167.1563427 | 187.7424648 | 1.123154897 | 0.139394781 | unchanged |
| hsa_circ_100800 | 150 | 76 | 97 | 189 | 107 | 94 | 107.5666227 | 130.0766821 | 1.20926621 | 0.573925932 | unchanged |
| hsa_circ_100801 | 69 | 61 | 56 | 68 | 66 | 58 | 62.00880133 | 63.77409652 | 1.028468462 | 0.729552464 | unchanged |
| hsa_circ_100802 | 1113 | 500 | 668 | 739 | 490 | 482 | 760.5156821 | 570.3798618 | 0.749990928 | 0.398894224 | unchanged |
| hsa_circ_100803 | 82 | 142 | 98 | 86 | 88 | 93 | 107.3087379 | 89.14017133 | 0.830688843 | 0.368049036 | unchanged |
| hsa_circ_100805 | 1526 | 1167 | 798 | 1576 | 1203 | 1053 | 1163.466992 | 1277.360437 | 1.097891428 | 0.685607674 | unchanged |
| hsa_circ_100806 | 991 | 1330 | 1427 | 1323 | 1748 | 1844 | 1249.35588 | 1638.151555 | 1.311196898 | 0.134249524 | unchanged |
| hsa_circ_100807 | 125 | 95 | 112 | 132 | 125 | 118 | 110.5869022 | 125.1232441 | 1.131447229 | 0.198016324 | unchanged |
| hsa_circ_100808 | 815 | 1135 | 1110 | 1128 | 1469 | 1635 | 1020.095758 | 1410.744752 | 1.382953258 | 0.09746741 | unchanged |
| hsa_circ_100809 | 417 | 316 | 250 | 406 | 246 | 234 | 327.7600207 | 295.2984313 | 0.900959277 | 0.681930104 | unchanged |
| hsa_circ_100810 | 287 | 240 | 293 | 308 | 361 | 383 | 273.3995746 | 350.6953987 | 1.282721084 | 0.049246657 | unchanged |
| hsa_circ_100811 | 101 | 68 | 88 | 111 | 90 | 93 | 85.81419218 | 97.88715589 | 1.140687262 | 0.350813511 | unchanged |
| hsa_circ_100812 | 99 | 71 | 76 | 108 | 95 | 95 | 82.14509242 | 99.18161573 | 1.20739551 | 0.15527915 | unchanged |
| hsa_circ_100813 | 59 | 72 | 61 | 61 | 69 | 71 | 63.87850119 | 66.82914154 | 1.046191446 | 0.595268875 | unchanged |
| hsa_circ_100814 | 111 | 64 | 75 | 90 | 75 | 89 | 83.41871361 | 84.88934301 | 1.01762949 | 0.926416785 | unchanged |
| hsa_circ_100815 | 1469 | 732 | 752 | 1569 | 1015 | 928 | 984.2726709 | 1170.880706 | 1.189589776 | 0.58517906 | unchanged |
| hsa_circ_100817 | 73 | 76 | 91 | 69 | 96 | 97 | 79.65581129 | 87.0509904 | 1.092839166 | 0.530566764 | unchanged |
| hsa_circ_100818 | 269 | 332 | 335 | 274 | 421 | 410 | 311.8816582 | 368.3400164 | 1.181024939 | 0.33822355 | unchanged |
| hsa_circ_100819 | 63 | 78 | 59 | 56 | 56 | 57 | 66.91243626 | 56.39594823 | 0.842832086 | 0.145216606 | unchanged |
| hsa_circ_100820 | 171 | 180 | 138 | 142 | 119 | 119 | 162.8524635 | 126.663844 | 0.777782794 | 0.072245423 | unchanged |
| hsa_circ_100821 | 4176 | 4765 | 5006 | 4144 | 4657 | 4614 | 4649.158216 | 4471.659938 | 0.961821416 | 0.581435004 | unchanged |
| hsa_circ_100822 | 137 | 100 | 127 | 212 | 116 | 113 | 121.1837674 | 146.7989125 | 1.211374391 | 0.495816913 | unchanged |
| hsa_circ_100823 | 122 | 217 | 132 | 134 | 149 | 140 | 156.8538716 | 141.1150063 | 0.899659057 | 0.636241138 | unchanged |
| hsa_circ_100824 | 73 | 84 | 81 | 77 | 89 | 89 | 79.17098004 | 84.84544171 | 1.071673505 | 0.310719486 | unchanged |
| hsa_circ_100825 | 87 | 61 | 70 | 84 | 72 | 70 | 72.51631555 | 75.19889501 | 1.036992771 | 0.782649116 | unchanged |
| hsa_circ_100827 | 2826 | 1983 | 1704 | 4382 | 2074 | 2085 | 2170.838845 | 2847.169732 | 1.311552785 | 0.464955732 | unchanged |
| hsa_circ_100828 | 119 | 193 | 129 | 114 | 197 | 138 | 147.3435495 | 149.7483311 | 1.016320916 | 0.946651261 | unchanged |
| hsa_circ_100829 | 455 | 375 | 314 | 377 | 362 | 391 | 381.1643769 | 376.8812676 | 0.988763091 | 0.922825227 | unchanged |
| hsa_circ_100830 | 117 | 111 | 342 | 132 | 203 | 190 | 189.9651093 | 174.997333 | 0.921207761 | 0.859021957 | unchanged |
| hsa_circ_100831 | 76 | 77 | 90 | 67 | 87 | 90 | 80.85419909 | 81.39050871 | 1.006633046 | 0.952068906 | unchanged |
| hsa_circ_100833 | 142 | 228 | 139 | 125 | 130 | 114 | 169.8405744 | 122.7280799 | 0.722607542 | 0.187853326 | unchanged |
| hsa_circ_100834 | 192 | 213 | 258 | 160 | 224 | 217 | 221.3262611 | 200.4690866 | 0.905762767 | 0.499590389 | unchanged |
| hsa_circ_100835 | 178 | 270 | 236 | 199 | 209 | 206 | 227.9295577 | 204.7430023 | 0.898273152 | 0.439463736 | unchanged |
| hsa_circ_100836 | 861 | 863 | 564 | 885 | 896 | 964 | 763.0274266 | 915.0220415 | 1.199199412 | 0.211499718 | unchanged |
| hsa_circ_100837 | 59 | 87 | 70 | 65 | 73 | 75 | 72.12706034 | 71.06706373 | 0.98530376 | 0.907519672 | unchanged |
| hsa_circ_100839 | 120 | 117 | 98 | 122 | 95 | 114 | 111.4769523 | 110.6374377 | 0.992469165 | 0.939808407 | unchanged |
| hsa_circ_100840 | 906 | 1151 | 1094 | 1041 | 1346 | 1111 | 1050.406012 | 1166.086735 | 1.110129533 | 0.384279766 | unchanged |
| hsa_circ_100842 | 152 | 254 | 236 | 141 | 249 | 234 | 213.9347919 | 208.2487728 | 0.973421718 | 0.907772649 | unchanged |
| hsa_circ_100844 | 176 | 76 | 177 | 112 | 81 | 88 | 143.327257 | 93.60289916 | 0.653071168 | 0.226468928 | unchanged |
| hsa_circ_100845 | 4974 | 6547 | 5132 | 4968 | 4909 | 4982 | 5551.139988 | 4952.998927 | 0.892248968 | 0.298205986 | unchanged |
| hsa_circ_100846 | 76 | 78 | 87 | 79 | 71 | 74 | 80.6345066 | 74.75248361 | 0.927053277 | 0.234684049 | unchanged |
| hsa_circ_100847 | 88 | 62 | 74 | 85 | 74 | 80 | 74.58520022 | 79.38290476 | 1.064325155 | 0.590495164 | unchanged |
| hsa_circ_100848 | 449 | 423 | 554 | 379 | 524 | 574 | 475.2271198 | 492.3160636 | 1.03595953 | 0.820605766 | unchanged |
| hsa_circ_100849 | 613 | 1237 | 1145 | 691 | 1265 | 1261 | 998.2886969 | 1072.460576 | 1.074299027 | 0.798780416 | unchanged |
| hsa_circ_100850 | 3250 | 2168 | 2578 | 2731 | 2022 | 1883 | 2665.402352 | 2211.945454 | 0.829873003 | 0.331142902 | unchanged |
| hsa_circ_100851 | 2386 | 2090 | 1802 | 2328 | 2186 | 2191 | 2092.606236 | 2235.079413 | 1.068084083 | 0.461075894 | unchanged |
| hsa_circ_100852 | 1163 | 513 | 319 | 1509 | 407 | 398 | 664.9985273 | 771.1628098 | 1.159645891 | 0.824446136 | unchanged |
| hsa_circ_100853 | 606 | 1020 | 893 | 671 | 983 | 1025 | 839.8106273 | 892.6568019 | 1.062926299 | 0.765655729 | unchanged |
| hsa_circ_100854 | 153 | 111 | 213 | 154 | 154 | 158 | 159.1294671 | 155.1114601 | 0.974750075 | 0.898875431 | unchanged |
| hsa_circ_100855 | 409 | 329 | 324 | 401 | 414 | 411 | 353.6778501 | 408.726803 | 1.15564716 | 0.118503678 | unchanged |
| hsa_circ_100856 | 66 | 76 | 84 | 78 | 95 | 107 | 75.33770632 | 93.36261203 | 1.239254771 | 0.145030214 | unchanged |
| hsa_circ_100857 | 175 | 340 | 247 | 183 | 212 | 214 | 254.0948112 | 203.2865957 | 0.800042294 | 0.356588934 | unchanged |
| hsa_circ_100858 | 88 | 133 | 94 | 92 | 100 | 99 | 104.914554 | 97.07569845 | 0.92528343 | 0.614439325 | unchanged |
| hsa_circ_100860 | 1465 | 2470 | 2297 | 1601 | 2136 | 2067 | 2076.988808 | 1934.728222 | 0.93150633 | 0.707514481 | unchanged |
| hsa_circ_100861 | 6716 | 10741 | 11035 | 6536 | 12773 | 12928 | 9497.270936 | 10745.24546 | 1.131403487 | 0.646997159 | unchanged |
| hsa_circ_100862 | 680 | 1030 | 1030 | 665 | 1039 | 1032 | 913.4794757 | 911.9756609 | 0.998353751 | 0.993361755 | unchanged |
| hsa_circ_100863 | 68 | 122 | 66 | 65 | 64 | 72 | 85.19362683 | 66.98544314 | 0.786272937 | 0.381994316 | unchanged |
| hsa_circ_100867 | 109 | 114 | 196 | 98 | 105 | 102 | 139.7633862 | 101.3720643 | 0.725312022 | 0.2454148 | unchanged |
| hsa_circ_100868 | 104 | 73 | 72 | 89 | 66 | 68 | 83.24825555 | 74.23417727 | 0.891720515 | 0.525625779 | unchanged |
| hsa_circ_100869 | 102 | 98 | 124 | 104 | 127 | 130 | 108.1471398 | 120.6854974 | 1.115937949 | 0.336930227 | unchanged |
| hsa_circ_100870 | 85 | 103 | 94 | 83 | 101 | 98 | 94.42852217 | 94.22073923 | 0.997799574 | 0.979489933 | unchanged |
| hsa_circ_100872 | 265 | 287 | 217 | 319 | 244 | 250 | 256.4298843 | 270.9195061 | 1.056505199 | 0.670955949 | unchanged |
| hsa_circ_100873 | 534 | 656 | 396 | 502 | 465 | 468 | 528.3721805 | 478.0953969 | 0.904845892 | 0.544930412 | unchanged |
| hsa_circ_100874 | 414 | 517 | 502 | 419 | 566 | 634 | 477.5950771 | 539.564081 | 1.129752183 | 0.431933429 | unchanged |
| hsa_circ_100876 | 1327 | 1173 | 891 | 1599 | 1171 | 1231 | 1130.449579 | 1333.673566 | 1.179772713 | 0.333889192 | unchanged |
| hsa_circ_100877 | 223 | 90 | 87 | 131 | 87 | 88 | 133.0372773 | 101.8769516 | 0.765777485 | 0.544734592 | unchanged |
| hsa_circ_100878 | 416 | 472 | 449 | 461 | 517 | 551 | 445.7472251 | 509.4363945 | 1.142881808 | 0.107866351 | unchanged |
| hsa_circ_100879 | 567 | 646 | 521 | 611 | 681 | 808 | 577.9641235 | 700.1778472 | 1.211455554 | 0.148463499 | unchanged |
| hsa_circ_100880 | 64 | 62 | 57 | 65 | 59 | 53 | 60.98425693 | 59.17745739 | 0.970372689 | 0.671877728 | unchanged |
| hsa_circ_100881 | 1351 | 1525 | 1279 | 1361 | 1534 | 1262 | 1384.973422 | 1385.840474 | 1.000626042 | 0.993972095 | unchanged |
| hsa_circ_100882 | 1800 | 1077 | 1263 | 1270 | 775 | 764 | 1379.874229 | 936.2271458 | 0.678487304 | 0.180279872 | unchanged |
| hsa_circ_100883 | 892 | 255 | 238 | 489 | 168 | 156 | 461.7146434 | 271.1165484 | 0.58719504 | 0.474095165 | unchanged |
| hsa_circ_100884 | 83 | 92 | 93 | 77 | 82 | 83 | 89.6140956 | 80.52025953 | 0.898522258 | 0.065107935 | unchanged |
| hsa_circ_100885 | 68 | 92 | 72 | 69 | 71 | 72 | 77.19622345 | 70.86754809 | 0.918018329 | 0.447755756 | unchanged |
| hsa_circ_100886 | 114 | 119 | 161 | 114 | 151 | 144 | 131.1089786 | 136.3567143 | 1.040025754 | 0.792672359 | unchanged |
| hsa_circ_100887 | 181 | 291 | 190 | 173 | 220 | 240 | 220.6396125 | 210.8310403 | 0.955544826 | 0.819755019 | unchanged |
| hsa_circ_100888 | 102 | 79 | 61 | 108 | 62 | 60 | 80.61138612 | 77.0338321 | 0.955619743 | 0.86480473 | unchanged |
| hsa_circ_100889 | 95 | 86 | 72 | 95 | 89 | 93 | 84.37081055 | 92.44938864 | 1.095750865 | 0.314087269 | unchanged |
| hsa_circ_100890 | 162 | 204 | 218 | 180 | 231 | 219 | 194.5996494 | 210.2945737 | 1.080652377 | 0.526971561 | unchanged |
| hsa_circ_100891 | 1025 | 838 | 815 | 2453 | 965 | 1038 | 892.5745878 | 1485.533682 | 1.664324419 | 0.291981555 | unchanged |
| hsa_circ_100892 | 377 | 507 | 461 | 340 | 466 | 460 | 447.9377064 | 421.8124433 | 0.941676571 | 0.664994161 | unchanged |
| hsa_circ_100893 | 384 | 593 | 463 | 360 | 452 | 476 | 480.2309439 | 429.1800427 | 0.893695103 | 0.508325998 | unchanged |
| hsa_circ_100894 | 100 | 99 | 109 | 96 | 103 | 110 | 102.7739601 | 103.1635892 | 1.003791127 | 0.94415757 | unchanged |
| hsa_circ_100895 | 2393 | 3185 | 4108 | 1980 | 3877 | 3782 | 3228.897188 | 3212.614448 | 0.994957182 | 0.984571478 | unchanged |
| hsa_circ_100896 | 1033 | 828 | 748 | 1004 | 1002 | 942 | 869.7008261 | 982.7517082 | 1.129988243 | 0.265284392 | unchanged |
| hsa_circ_100897 | 268 | 393 | 476 | 250 | 385 | 378 | 379.1181994 | 337.6220929 | 0.890545728 | 0.608379028 | unchanged |
| hsa_circ_100898 | 62 | 60 | 67 | 64 | 71 | 76 | 63.00508642 | 70.25981432 | 1.115145115 | 0.16282755 | unchanged |
| hsa_circ_100899 | 277 | 294 | 240 | 287 | 262 | 264 | 270.3720365 | 271.051637 | 1.002513575 | 0.971061915 | unchanged |
| hsa_circ_100900 | 76 | 85 | 242 | 89 | 150 | 134 | 134.3383691 | 124.7361401 | 0.928522066 | 0.873927291 | unchanged |
| hsa_circ_100901 | 166 | 81 | 93 | 126 | 76 | 86 | 113.2862378 | 96.04208368 | 0.847782445 | 0.601950298 | unchanged |
| hsa_circ_100903 | 166 | 116 | 130 | 146 | 152 | 152 | 137.1160294 | 150.2426504 | 1.095733673 | 0.429276156 | unchanged |
| hsa_circ_100904 | 18629 | 11460 | 5391 | 14099 | 8706 | 7489 | 11826.74086 | 10098.12682 | 0.853838512 | 0.710214662 | unchanged |
| hsa_circ_100905 | 199 | 266 | 275 | 191 | 250 | 255 | 246.5257694 | 231.9970923 | 0.941066294 | 0.669860192 | unchanged |
| hsa_circ_100908 | 677 | 843 | 875 | 745 | 801 | 870 | 798.4711125 | 805.4085621 | 1.008688417 | 0.92702339 | unchanged |
| hsa_circ_100909 | 721 | 1153 | 1015 | 803 | 1371 | 1372 | 962.9315707 | 1182.005733 | 1.227507508 | 0.391106499 | unchanged |
| hsa_circ_100910 | 439 | 596 | 636 | 503 | 806 | 824 | 556.9799942 | 710.959916 | 1.276455031 | 0.26924944 | unchanged |
| hsa_circ_100911 | 81 | 73 | 94 | 81 | 110 | 112 | 82.48051415 | 101.0040166 | 1.224580347 | 0.186912057 | unchanged |
| hsa_circ_100912 | 880 | 1205 | 1139 | 929 | 1384 | 1365 | 1074.695423 | 1225.88861 | 1.140684685 | 0.444940236 | unchanged |
| hsa_circ_100913 | 170 | 224 | 262 | 198 | 329 | 331 | 218.6722973 | 285.8942524 | 1.307409562 | 0.260400207 | unchanged |
| hsa_circ_100914 | 83 | 75 | 106 | 88 | 121 | 131 | 88.07686259 | 113.4931614 | 1.288569529 | 0.187406087 | unchanged |
| hsa_circ_100916 | 615 | 1011 | 781 | 656 | 1079 | 1067 | 802.2665369 | 934.0208291 | 1.16422758 | 0.505987697 | unchanged |
| hsa_circ_100917 | 195 | 271 | 295 | 234 | 288 | 323 | 253.8276059 | 281.9154719 | 1.110657255 | 0.518481008 | unchanged |
| hsa_circ_100918 | 110 | 81 | 91 | 106 | 96 | 102 | 93.90062701 | 101.5766509 | 1.081746247 | 0.440870735 | unchanged |
| hsa_circ_100919 | 221 | 438 | 407 | 337 | 555 | 526 | 355.6127673 | 472.8558048 | 1.329692993 | 0.289758728 | unchanged |
| hsa_circ_100920 | 244 | 420 | 500 | 330 | 561 | 555 | 388.0988919 | 481.8931711 | 1.241676236 | 0.43095233 | unchanged |
| hsa_circ_100921 | 74 | 141 | 96 | 89 | 108 | 105 | 103.9579304 | 100.7526414 | 0.969167441 | 0.88292486 | unchanged |
| hsa_circ_100922 | 99 | 179 | 126 | 105 | 156 | 164 | 134.4706187 | 141.4558296 | 1.051946001 | 0.827397857 | unchanged |
| hsa_circ_100923 | 200 | 139 | 172 | 195 | 255 | 228 | 170.3395924 | 226.1906869 | 1.327880874 | 0.087212814 | unchanged |
| hsa_circ_100924 | 93 | 88 | 122 | 98 | 147 | 153 | 101.0590899 | 132.3955643 | 1.31008071 | 0.200090575 | unchanged |
| hsa_circ_100925 | 73 | 67 | 106 | 81 | 117 | 118 | 82.10905412 | 105.2782168 | 1.282175492 | 0.25260515 | unchanged |
| hsa_circ_100926 | 72 | 67 | 96 | 78 | 90 | 100 | 78.63184731 | 89.43364975 | 1.137371851 | 0.384806777 | unchanged |
| hsa_circ_100927 | 79 | 74 | 100 | 83 | 111 | 113 | 84.29369386 | 102.0234863 | 1.210333557 | 0.231925227 | unchanged |
| hsa_circ_100928 | 86 | 66 | 86 | 88 | 111 | 106 | 79.00595379 | 101.7150189 | 1.287434858 | 0.079537691 | unchanged |
| hsa_circ_100929 | 81 | 56 | 67 | 68 | 67 | 62 | 67.71183549 | 65.91325839 | 0.973437774 | 0.819513591 | unchanged |
| hsa_circ_100932 | 120 | 200 | 130 | 145 | 173 | 178 | 150.1374076 | 165.3925757 | 1.101608042 | 0.605249614 | unchanged |
| hsa_circ_100933 | 88 | 67 | 68 | 85 | 64 | 65 | 74.2558793 | 71.31708042 | 0.960423351 | 0.775367534 | unchanged |
| hsa_circ_100934 | 92 | 89 | 80 | 97 | 82 | 80 | 87.34002411 | 86.10068736 | 0.985810208 | 0.85721741 | unchanged |
| hsa_circ_100936 | 70 | 85 | 57 | 74 | 60 | 59 | 70.56870077 | 64.18809992 | 0.909583133 | 0.530175803 | unchanged |
| hsa_circ_100937 | 90 | 109 | 122 | 96 | 148 | 134 | 106.8211036 | 125.7544362 | 1.177243372 | 0.352426282 | unchanged |
| hsa_circ_100938 | 578 | 988 | 833 | 566 | 933 | 886 | 799.6596679 | 795.1638862 | 0.994377881 | 0.979694411 | unchanged |
| hsa_circ_100939 | 800 | 902 | 700 | 747 | 838 | 848 | 800.9103532 | 810.9921446 | 1.012587915 | 0.887172424 | unchanged |
| hsa_circ_100940 | 130 | 66 | 82 | 129 | 84 | 90 | 92.48679945 | 101.4089567 | 1.096469521 | 0.728892039 | unchanged |
| hsa_circ_100942 | 90 | 77 | 112 | 93 | 123 | 110 | 92.80413661 | 108.6624532 | 1.170879415 | 0.305797417 | unchanged |
| hsa_circ_100943 | 62 | 54 | 79 | 58 | 86 | 89 | 64.89107449 | 77.67427558 | 1.196994751 | 0.360584039 | unchanged |
| hsa_circ_100944 | 1798 | 2683 | 2845 | 1724 | 2750 | 2583 | 2442.007497 | 2352.195573 | 0.963222093 | 0.853108744 | unchanged |
| hsa_circ_100945 | 121 | 109 | 103 | 99 | 106 | 105 | 111.0608223 | 103.1672453 | 0.928925639 | 0.244434494 | unchanged |
| hsa_circ_100946 | 700 | 449 | 393 | 1031 | 536 | 692 | 514.058366 | 752.8819824 | 1.464584631 | 0.24217211 | unchanged |
| hsa_circ_100948 | 149 | 126 | 99 | 135 | 82 | 78 | 124.5523112 | 98.4351574 | 0.790311769 | 0.323844014 | unchanged |
| hsa_circ_100949 | 187 | 175 | 236 | 162 | 206 | 213 | 199.3825645 | 194.0343211 | 0.973175973 | 0.838923081 | unchanged |
| hsa_circ_100950 | 257 | 195 | 186 | 225 | 197 | 190 | 212.544433 | 203.8550868 | 0.959117507 | 0.742320826 | unchanged |
| hsa_circ_100951 | 177 | 269 | 178 | 216 | 205 | 200 | 208.0100838 | 206.9867657 | 0.99508044 | 0.975034538 | unchanged |
| hsa_circ_100952 | 102 | 112 | 88 | 102 | 87 | 89 | 100.6049812 | 92.47476605 | 0.919186754 | 0.385512812 | unchanged |
| hsa_circ_100953 | 85 | 87 | 70 | 96 | 84 | 86 | 80.56259581 | 88.58974276 | 1.099638633 | 0.297245665 | unchanged |
| hsa_circ_100956 | 354 | 638 | 522 | 548 | 723 | 689 | 504.7070735 | 653.172434 | 1.294161442 | 0.205686584 | unchanged |
| hsa_circ_100957 | 69 | 75 | 73 | 73 | 73 | 77 | 72.34323503 | 74.33899761 | 1.027587411 | 0.430460346 | unchanged |
| hsa_circ_100958 | 100 | 75 | 84 | 100 | 98 | 97 | 86.14163414 | 98.305156 | 1.141203751 | 0.17233714 | unchanged |
| hsa_circ_100960 | 1352 | 1919 | 2372 | 1771 | 3033 | 2927 | 1880.939351 | 2576.93163 | 1.370023775 | 0.236622137 | unchanged |
| hsa_circ_100961 | 124 | 129 | 184 | 116 | 202 | 227 | 145.8554262 | 181.7748657 | 1.24626742 | 0.406896821 | unchanged |
| hsa_circ_100962 | 214 | 187 | 184 | 207 | 211 | 196 | 194.9589826 | 205.0187127 | 1.051599213 | 0.391041432 | unchanged |
| hsa_circ_100963 | 60 | 104 | 71 | 61 | 73 | 66 | 78.49173598 | 66.99733624 | 0.853559109 | 0.448465868 | unchanged |
| hsa_circ_100964 | 161 | 269 | 184 | 161 | 193 | 231 | 204.6130433 | 195.1423072 | 0.953713918 | 0.817832304 | unchanged |
| hsa_circ_100966 | 83 | 69 | 66 | 78 | 59 | 63 | 72.41095986 | 66.6200783 | 0.920027554 | 0.494144333 | unchanged |
| hsa_circ_100967 | 95 | 67 | 78 | 96 | 71 | 81 | 80.05511645 | 83.00863971 | 1.036893623 | 0.797680522 | unchanged |
| hsa_circ_100968 | 494 | 846 | 880 | 436 | 733 | 823 | 739.9297906 | 663.7319194 | 0.897020133 | 0.677108009 | unchanged |
| hsa_circ_100969 | 123 | 96 | 122 | 126 | 114 | 125 | 113.4385491 | 121.7846227 | 1.073573522 | 0.431134102 | unchanged |
| hsa_circ_100970 | 6578 | 8530 | 9182 | 6901 | 9473 | 9552 | 8096.634361 | 8642.040564 | 1.067362089 | 0.66552193 | unchanged |
| hsa_circ_100972 | 1434 | 1697 | 1185 | 1616 | 1286 | 1312 | 1438.812195 | 1404.645354 | 0.976253439 | 0.86025896 | unchanged |
| hsa_circ_100973 | 62 | 60 | 86 | 65 | 73 | 79 | 69.3361059 | 72.24189511 | 1.041908745 | 0.767015092 | unchanged |
| hsa_circ_100974 | 203 | 523 | 440 | 220 | 242 | 252 | 388.6183506 | 237.9986075 | 0.612422463 | 0.192810803 | unchanged |
| hsa_circ_100975 | 96 | 82 | 75 | 121 | 89 | 85 | 84.05532075 | 98.26239962 | 1.169020578 | 0.33591991 | unchanged |
| hsa_circ_100977 | 123 | 156 | 227 | 207 | 354 | 323 | 168.6252228 | 294.8246405 | 1.748401785 | 0.080641076 | unchanged |
| hsa_circ_100979 | 412 | 450 | 287 | 365 | 311 | 305 | 383.2487703 | 327.0620805 | 0.853393685 | 0.345929774 | unchanged |
| hsa_circ_100980 | 430 | 319 | 454 | 448 | 533 | 532 | 401.2656945 | 504.1816308 | 1.256478283 | 0.108870159 | unchanged |
| hsa_circ_100981 | 104 | 115 | 79 | 96 | 70 | 72 | 99.37892327 | 79.49309085 | 0.799898894 | 0.21823018 | unchanged |
| hsa_circ_100982 | 124 | 107 | 142 | 136 | 183 | 187 | 124.3777265 | 168.7418756 | 1.356688857 | 0.084531063 | unchanged |
| hsa_circ_100983 | 63 | 63 | 81 | 66 | 83 | 89 | 68.99396276 | 79.34982952 | 1.150098159 | 0.325424735 | unchanged |
| hsa_circ_100984 | 568 | 1109 | 825 | 688 | 1023 | 1053 | 833.6309896 | 921.1404611 | 1.10497387 | 0.677236313 | unchanged |
| hsa_circ_100985 | 67 | 77 | 77 | 63 | 66 | 63 | 73.60143679 | 64.09457748 | 0.870833238 | 0.062601649 | unchanged |
| hsa_circ_100986 | 247 | 201 | 208 | 255 | 216 | 212 | 218.4623252 | 227.570059 | 1.04169018 | 0.668621125 | unchanged |
| hsa_circ_100987 | 159 | 185 | 198 | 187 | 199 | 200 | 180.8319399 | 195.6615623 | 1.08200776 | 0.292065536 | unchanged |
| hsa_circ_100988 | 446 | 491 | 467 | 362 | 403 | 440 | 467.9682585 | 401.5973879 | 0.858172281 | 0.064230198 | unchanged |
| hsa_circ_100989 | 169 | 186 | 318 | 166 | 612 | 718 | 224.3900514 | 498.5363364 | 2.221739927 | 0.193705367 | unchanged |
| hsa_circ_100990 | 71 | 102 | 81 | 73 | 73 | 85 | 84.65015039 | 76.79417985 | 0.907194842 | 0.479646951 | unchanged |
| hsa_circ_100993 | 108 | 68 | 119 | 107 | 141 | 154 | 98.58625671 | 134.0405252 | 1.359626886 | 0.164325506 | unchanged |
| hsa_circ_100996 | 196 | 168 | 257 | 142 | 277 | 236 | 207.1793034 | 218.3004923 | 1.053679053 | 0.828309892 | unchanged |
| hsa_circ_100997 | 839 | 430 | 511 | 712 | 509 | 493 | 593.0927205 | 571.412004 | 0.963444642 | 0.88717672 | unchanged |
| hsa_circ_100998 | 70 | 110 | 88 | 67 | 84 | 93 | 89.32274806 | 81.20523383 | 0.909121535 | 0.593583819 | unchanged |
| hsa_circ_100999 | 203 | 271 | 229 | 207 | 182 | 187 | 233.9908227 | 191.8283522 | 0.819811435 | 0.119952847 | unchanged |
| hsa_circ_101001 | 411 | 569 | 498 | 419 | 664 | 655 | 492.2693034 | 579.0957534 | 1.17637998 | 0.400444859 | unchanged |
| hsa_circ_101002 | 380 | 653 | 557 | 434 | 643 | 714 | 529.8647788 | 597.1186564 | 1.126926492 | 0.593325121 | unchanged |
| hsa_circ_101003 | 1756 | 2749 | 2417 | 2245 | 2870 | 3052 | 2307.374286 | 2722.073481 | 1.179727753 | 0.337277958 | unchanged |
| hsa_circ_101005 | 89 | 109 | 83 | 87 | 83 | 89 | 93.50146869 | 86.40016238 | 0.924051393 | 0.43261229 | unchanged |
| hsa_circ_101006 | 80 | 88 | 72 | 77 | 75 | 76 | 79.77378471 | 75.93977908 | 0.951939028 | 0.454773922 | unchanged |
| hsa_circ_101007 | 62 | 77 | 54 | 60 | 52 | 57 | 64.22921938 | 56.46555541 | 0.879125668 | 0.344262778 | unchanged |
| hsa_circ_101008 | 64 | 58 | 60 | 65 | 63 | 58 | 60.48651805 | 61.79952433 | 1.021707421 | 0.644038208 | unchanged |
| hsa_circ_101009 | 139 | 143 | 184 | 178 | 253 | 267 | 155.312739 | 232.3872464 | 1.49625361 | 0.068318341 | unchanged |
| hsa_circ_101010 | 122 | 132 | 106 | 134 | 108 | 115 | 119.8071437 | 118.9900245 | 0.993179712 | 0.943700321 | unchanged |
| hsa_circ_101011 | 298 | 316 | 368 | 362 | 502 | 512 | 327.5291024 | 458.4734798 | 1.399794633 | 0.068594055 | unchanged |
| hsa_circ_101013 | 148 | 192 | 208 | 145 | 181 | 174 | 182.7314926 | 166.8419426 | 0.913044272 | 0.490210864 | unchanged |
| hsa_circ_101014 | 649 | 937 | 882 | 885 | 1087 | 998 | 822.5598285 | 989.6552952 | 1.203140806 | 0.189473388 | unchanged |
| hsa_circ_101015 | 168 | 231 | 192 | 181 | 210 | 222 | 196.9723702 | 204.1957918 | 1.036672258 | 0.760464129 | unchanged |
| hsa_circ_101016 | 90 | 67 | 83 | 90 | 87 | 94 | 80.02268819 | 90.23576015 | 1.127627204 | 0.229267084 | unchanged |
| hsa_circ_101017 | 79 | 68 | 128 | 78 | 70 | 66 | 91.7776233 | 71.12922806 | 0.775017107 | 0.337371258 | unchanged |
| hsa_circ_101018 | 587 | 736 | 623 | 581 | 652 | 587 | 648.5044685 | 606.8193254 | 0.935721117 | 0.453001085 | unchanged |
| hsa_circ_101020 | 106 | 90 | 62 | 95 | 82 | 84 | 85.96007208 | 86.837754 | 1.010210344 | 0.95137095 | unchanged |
| hsa_circ_101021 | 118 | 101 | 105 | 107 | 121 | 107 | 107.9759064 | 111.9554255 | 1.036855621 | 0.605126561 | unchanged |
| hsa_circ_101022 | 171 | 87 | 120 | 198 | 185 | 161 | 126.0158817 | 181.3874653 | 1.439401628 | 0.106488517 | unchanged |
| hsa_circ_101023 | 154 | 218 | 225 | 159 | 240 | 239 | 199.3378043 | 212.745016 | 1.067258751 | 0.720936033 | unchanged |
| hsa_circ_101024 | 74 | 67 | 71 | 86 | 73 | 78 | 70.5712319 | 78.79598896 | 1.116545465 | 0.141368523 | unchanged |
| hsa_circ_101027 | 104 | 99 | 125 | 99 | 131 | 141 | 109.0755962 | 123.8801998 | 1.135727917 | 0.377397472 | unchanged |
| hsa_circ_101029 | 107 | 89 | 80 | 112 | 98 | 91 | 92.32167798 | 100.3920439 | 1.087415719 | 0.468682851 | unchanged |
| hsa_circ_101030 | 1272 | 795 | 472 | 1004 | 689 | 663 | 846.3581318 | 785.2940669 | 0.927850797 | 0.823800635 | unchanged |
| hsa_circ_101031 | 58249 | 33406 | 26487 | 53459 | 35852 | 36426 | 39380.61387 | 41912.40292 | 1.064290238 | 0.832835473 | unchanged |
| hsa_circ_101032 | 159 | 239 | 219 | 191 | 239 | 219 | 205.356758 | 216.2198411 | 1.052898591 | 0.715525967 | unchanged |
| hsa_circ_101034 | 162 | 157 | 217 | 201 | 305 | 273 | 178.5370668 | 259.7826633 | 1.455062907 | 0.088409725 | unchanged |
| hsa_circ_101037 | 352 | 283 | 247 | 404 | 213 | 222 | 293.9326956 | 279.7364136 | 0.95170227 | 0.848271819 | unchanged |
| hsa_circ_101039 | 65 | 62 | 56 | 64 | 64 | 62 | 60.84712179 | 63.40475364 | 1.042033736 | 0.405104199 | unchanged |
| hsa_circ_101040 | 1200 | 1461 | 1472 | 1401 | 1681 | 1764 | 1377.852423 | 1615.275583 | 1.172313926 | 0.16792956 | unchanged |
| hsa_circ_101043 | 2910 | 2829 | 3690 | 3567 | 4816 | 4438 | 3143.338473 | 4273.47592 | 1.359534125 | 0.070139119 | unchanged |
| hsa_circ_101045 | 82 | 70 | 83 | 83 | 85 | 84 | 78.62589926 | 83.99054295 | 1.068229982 | 0.277291995 | unchanged |
| hsa_circ_101051 | 16275 | 22259 | 31702 | 15267 | 24522 | 21797 | 23411.96163 | 20528.70358 | 0.876846798 | 0.61298848 | unchanged |
| hsa_circ_101052 | 68 | 61 | 76 | 66 | 71 | 64 | 68.28046759 | 67.19031239 | 0.984034157 | 0.827747856 | unchanged |
| hsa_circ_101054 | 125 | 134 | 104 | 121 | 121 | 114 | 121.01727 | 118.562255 | 0.979713515 | 0.803431229 | unchanged |
| hsa_circ_101055 | 419 | 315 | 248 | 585 | 327 | 351 | 327.3406562 | 421.0613286 | 1.286309295 | 0.385306302 | unchanged |
| hsa_circ_101056 | 90 | 57 | 82 | 91 | 89 | 86 | 76.28380215 | 88.54061832 | 1.160673902 | 0.292571246 | unchanged |
| hsa_circ_101057 | 1358 | 1698 | 1715 | 1459 | 2000 | 2084 | 1590.29127 | 1848.088185 | 1.162106729 | 0.321086712 | unchanged |
| hsa_circ_101060 | 88 | 124 | 116 | 103 | 119 | 130 | 109.3341499 | 117.5480874 | 1.075126916 | 0.579277614 | unchanged |
| hsa_circ_101061 | 295 | 335 | 289 | 340 | 326 | 368 | 306.0878062 | 344.6382425 | 1.125945678 | 0.115050183 | unchanged |
| hsa_circ_101062 | 317 | 314 | 271 | 336 | 273 | 320 | 300.7972709 | 309.4820992 | 1.028872697 | 0.738715566 | unchanged |
| hsa_circ_101063 | 104 | 79 | 124 | 104 | 116 | 112 | 102.1637893 | 110.3531825 | 1.08015945 | 0.577591266 | unchanged |
| hsa_circ_101064 | 126 | 68 | 97 | 130 | 114 | 127 | 97.00634428 | 123.5980792 | 1.274123668 | 0.204089774 | unchanged |
| hsa_circ_101065 | 63 | 83 | 54 | 60 | 55 | 56 | 66.44309305 | 57.2534027 | 0.86169081 | 0.345645119 | unchanged |
| hsa_circ_101066 | 4513 | 8057 | 9427 | 4574 | 5841 | 5805 | 7332.468835 | 5406.764764 | 0.73737303 | 0.274543272 | unchanged |
| hsa_circ_101067 | 221 | 286 | 243 | 249 | 209 | 215 | 250.0749799 | 224.0665948 | 0.895997652 | 0.316403284 | unchanged |
| hsa_circ_101068 | 73 | 66 | 232 | 71 | 74 | 76 | 124.0405474 | 73.83691081 | 0.595264309 | 0.405891402 | unchanged |
| hsa_circ_101069 | 65 | 91 | 82 | 74 | 65 | 64 | 79.47149912 | 67.94353278 | 0.854942131 | 0.235136856 | unchanged |
| hsa_circ_101070 | 121 | 103 | 109 | 125 | 127 | 125 | 110.8031589 | 125.7627901 | 1.135010872 | 0.047217097 | unchanged |
| hsa_circ_101072 | 483 | 408 | 257 | 432 | 245 | 264 | 382.6337214 | 313.5545119 | 0.819463874 | 0.481525913 | unchanged |
| hsa_circ_101073 | 234 | 159 | 170 | 241 | 218 | 223 | 187.9129312 | 227.2383822 | 1.209274853 | 0.182885373 | unchanged |
| hsa_circ_101074 | 69 | 103 | 95 | 72 | 87 | 82 | 89.23779071 | 80.46172058 | 0.901655229 | 0.478069974 | unchanged |
| hsa_circ_101075 | 1096 | 1357 | 1170 | 1250 | 1350 | 1340 | 1207.939739 | 1313.249048 | 1.087180929 | 0.278005705 | unchanged |
| hsa_circ_101076 | 1436 | 2531 | 3108 | 1822 | 2222 | 2412 | 2358.164814 | 2151.939367 | 0.912548331 | 0.712077349 | unchanged |
| hsa_circ_101077 | 296 | 315 | 254 | 307 | 261 | 257 | 288.4486912 | 275.3332497 | 0.954531111 | 0.614922066 | unchanged |
| hsa_circ_101079 | 67 | 54 | 78 | 66 | 97 | 99 | 66.22771742 | 87.39226564 | 1.319572364 | 0.167959844 | unchanged |
| hsa_circ_101080 | 200 | 194 | 161 | 175 | 117 | 122 | 184.9976126 | 138.2272801 | 0.747184129 | 0.102332117 | unchanged |
| hsa_circ_101081 | 72 | 58 | 64 | 69 | 66 | 66 | 64.73432885 | 66.92324494 | 1.033813838 | 0.644917052 | unchanged |
| hsa_circ_101083 | 77 | 60 | 78 | 74 | 100 | 90 | 71.56121152 | 87.8613709 | 1.227779254 | 0.163566232 | unchanged |
| hsa_circ_101084 | 97 | 79 | 106 | 103 | 143 | 125 | 94.02849641 | 124.0142555 | 1.318900762 | 0.101715126 | unchanged |
| hsa_circ_101085 | 372 | 407 | 491 | 364 | 634 | 624 | 423.2076493 | 540.8111398 | 1.277886023 | 0.284203245 | unchanged |
| hsa_circ_101086 | 69 | 61 | 88 | 73 | 99 | 96 | 72.49600451 | 89.16372314 | 1.229912238 | 0.214401923 | unchanged |
| hsa_circ_101087 | 88 | 89 | 84 | 94 | 81 | 85 | 86.79729501 | 86.48399721 | 0.996390466 | 0.941298818 | unchanged |
| hsa_circ_101088 | 442 | 351 | 292 | 383 | 276 | 279 | 361.7058443 | 312.5495257 | 0.864098633 | 0.43093249 | unchanged |
| hsa_circ_101089 | 140 | 129 | 130 | 138 | 129 | 128 | 132.7004318 | 131.6551932 | 0.992123322 | 0.83644253 | unchanged |
| hsa_circ_101090 | 1326 | 1182 | 1246 | 1387 | 1229 | 1340 | 1251.233806 | 1318.60716 | 1.053845535 | 0.342653532 | unchanged |
| hsa_circ_101091 | 115 | 103 | 146 | 122 | 155 | 149 | 121.5425973 | 141.7094155 | 1.165923871 | 0.280780289 | unchanged |
| hsa_circ_101092 | 285 | 161 | 175 | 257 | 200 | 192 | 206.8505964 | 216.5708808 | 1.046991812 | 0.837119525 | unchanged |
| hsa_circ_101093 | 133 | 95 | 355 | 125 | 257 | 239 | 194.5894924 | 207.0281966 | 1.063922795 | 0.897795181 | unchanged |
| hsa_circ_101094 | 3148 | 3556 | 2716 | 3698 | 2980 | 2879 | 3139.756049 | 3185.531371 | 1.014579261 | 0.903356253 | unchanged |
| hsa_circ_101096 | 102 | 103 | 106 | 111 | 152 | 144 | 103.6978192 | 135.9458998 | 1.31098128 | 0.063505214 | unchanged |
| hsa_circ_101098 | 64 | 62 | 75 | 70 | 82 | 73 | 66.94830631 | 75.07825421 | 1.1214362 | 0.198300448 | unchanged |
| hsa_circ_101099 | 74 | 76 | 190 | 116 | 108 | 101 | 113.1718668 | 108.6033417 | 0.959631972 | 0.911189485 | unchanged |
| hsa_circ_101101 | 207 | 157 | 191 | 181 | 211 | 212 | 185.3446708 | 201.3712042 | 1.086468812 | 0.418211181 | unchanged |
| hsa_circ_101102 | 119 | 80 | 126 | 117 | 114 | 111 | 108.3349455 | 113.885558 | 1.051235661 | 0.719303051 | unchanged |
| hsa_circ_101105 | 75 | 98 | 85 | 77 | 88 | 76 | 86.22888142 | 80.4362894 | 0.932823064 | 0.487257959 | unchanged |
| hsa_circ_101106 | 114 | 107 | 132 | 124 | 137 | 131 | 117.6897861 | 130.712871 | 1.110656033 | 0.180816487 | unchanged |
| hsa_circ_101109 | 97 | 100 | 107 | 107 | 98 | 98 | 101.3748873 | 101.220765 | 0.998479679 | 0.973180614 | unchanged |
| hsa_circ_101110 | 133 | 140 | 189 | 129 | 157 | 178 | 153.8944126 | 154.849412 | 1.00620555 | 0.968560918 | unchanged |
| hsa_circ_101113 | 119 | 80 | 71 | 123 | 90 | 86 | 90.06265481 | 99.59110483 | 1.105798014 | 0.63755516 | unchanged |
| hsa_circ_101114 | 406 | 314 | 290 | 405 | 299 | 321 | 336.6606873 | 341.9376694 | 1.015674483 | 0.917646483 | unchanged |
| hsa_circ_101115 | 1608 | 3373 | 3464 | 1511 | 2546 | 2532 | 2814.926408 | 2196.205002 | 0.780199794 | 0.423352192 | unchanged |
| hsa_circ_101116 | 113 | 106 | 148 | 105 | 213 | 221 | 122.3984809 | 179.7700985 | 1.468728183 | 0.220031831 | unchanged |
| hsa_circ_101117 | 432 | 544 | 459 | 430 | 520 | 533 | 477.9389563 | 494.4262959 | 1.034496748 | 0.743072013 | unchanged |
| hsa_circ_101118 | 67 | 63 | 80 | 72 | 99 | 100 | 69.84954613 | 89.99854203 | 1.288462804 | 0.127643013 | unchanged |
| hsa_circ_101119 | 260 | 212 | 266 | 213 | 290 | 280 | 246.2120433 | 261.0667648 | 1.060333042 | 0.642121104 | unchanged |
| hsa_circ_101120 | 82 | 78 | 74 | 92 | 65 | 67 | 77.97178818 | 74.80439084 | 0.959377649 | 0.743922974 | unchanged |
| hsa_circ_101122 | 80 | 77 | 88 | 77 | 78 | 81 | 81.84851007 | 78.6626047 | 0.961075585 | 0.418901837 | unchanged |
| hsa_circ_101124 | 82 | 93 | 83 | 84 | 92 | 100 | 86.24760599 | 91.88211542 | 1.065329459 | 0.384069566 | unchanged |
| hsa_circ_101125 | 301 | 481 | 373 | 367 | 389 | 382 | 384.8758456 | 379.0573334 | 0.984882106 | 0.917122171 | unchanged |
| hsa_circ_101126 | 134 | 94 | 96 | 141 | 112 | 115 | 107.8216924 | 122.6979582 | 1.137970992 | 0.401838927 | unchanged |
| hsa_circ_101127 | 1633 | 954 | 1064 | 1054 | 812 | 699 | 1216.983732 | 854.9726301 | 0.702534149 | 0.198075309 | unchanged |
| hsa_circ_101128 | 523 | 798 | 823 | 401 | 583 | 553 | 714.6472268 | 512.3670733 | 0.716951041 | 0.143433148 | unchanged |
| hsa_circ_101129 | 74 | 83 | 77 | 79 | 68 | 70 | 78.11087209 | 72.31704844 | 0.925825644 | 0.256062239 | unchanged |
| hsa_circ_101131 | 50 | 103 | 127 | 48 | 47 | 54 | 93.10040377 | 49.88079836 | 0.535774243 | 0.133073208 | unchanged |
| hsa_circ_101136 | 1311 | 604 | 639 | 753 | 444 | 435 | 851.5704656 | 544.1360499 | 0.638979476 | 0.290606939 | unchanged |
| hsa_circ_101138 | 566 | 886 | 713 | 621 | 954 | 903 | 721.78695 | 826.2063896 | 1.144667952 | 0.494119364 | unchanged |
| hsa_circ_101139 | 13327 | 6828 | 5942 | 15214 | 10570 | 9999 | 8698.855308 | 11927.82157 | 1.371194387 | 0.321187241 | unchanged |
| hsa_circ_101140 | 328 | 277 | 268 | 291 | 265 | 297 | 290.7028744 | 284.1949991 | 0.97761331 | 0.773433837 | unchanged |
| hsa_circ_101141 | 4927 | 6246 | 9132 | 5514 | 8193 | 8443 | 6768.484421 | 7383.371841 | 1.090845658 | 0.712865367 | unchanged |
| hsa_circ_101142 | 6398 | 4301 | 4464 | 5831 | 4302 | 5031 | 5054.548173 | 5054.72886 | 1.000035747 | 0.999831695 | unchanged |
| hsa_circ_101143 | 66 | 87 | 64 | 69 | 71 | 68 | 72.0221756 | 69.47523942 | 0.964636778 | 0.745838598 | unchanged |
| hsa_circ_101144 | 8362 | 10003 | 10792 | 8521 | 11619 | 10306 | 9719.022923 | 10148.7911 | 1.044219278 | 0.727139351 | unchanged |
| hsa_circ_101145 | 3734 | 6200 | 6991 | 5033 | 6378 | 6737 | 5641.681598 | 6049.427456 | 1.072273816 | 0.731810858 | unchanged |
| hsa_circ_101146 | 77 | 76 | 90 | 73 | 94 | 84 | 80.8898382 | 83.79706239 | 1.035940536 | 0.721381819 | unchanged |
| hsa_circ_101147 | 285 | 230 | 334 | 313 | 399 | 386 | 283.020445 | 365.9544555 | 1.293031871 | 0.107778626 | unchanged |
| hsa_circ_101151 | 132 | 87 | 121 | 133 | 157 | 153 | 113.5071974 | 147.786873 | 1.302004423 | 0.088925913 | unchanged |
| hsa_circ_101152 | 68 | 76 | 61 | 69 | 60 | 57 | 68.34540216 | 61.98865179 | 0.906990812 | 0.300939266 | unchanged |
| hsa_circ_101153 | 125 | 71 | 89 | 112 | 97 | 92 | 95.0066477 | 100.4825911 | 1.057637476 | 0.763733291 | unchanged |
| hsa_circ_101154 | 257 | 223 | 224 | 198 | 220 | 196 | 234.5352973 | 204.7271467 | 0.872905482 | 0.08844891 | unchanged |
| hsa_circ_101155 | 277 | 260 | 387 | 303 | 516 | 506 | 307.6830102 | 441.7140325 | 1.435613985 | 0.168628088 | unchanged |
| hsa_circ_101156 | 146 | 102 | 94 | 104 | 75 | 71 | 114.1601016 | 83.35200908 | 0.730132576 | 0.185045507 | unchanged |
| hsa_circ_101158 | 74 | 91 | 81 | 73 | 85 | 84 | 82.18636989 | 80.34641072 | 0.977612356 | 0.779767051 | unchanged |
| hsa_circ_101159 | 623 | 1256 | 1061 | 671 | 1222 | 1165 | 979.8707962 | 1019.299476 | 1.040238652 | 0.885066617 | unchanged |
| hsa_circ_101160 | 100 | 135 | 117 | 89 | 115 | 117 | 117.3264842 | 106.8599016 | 0.910790964 | 0.481991191 | unchanged |
| hsa_circ_101161 | 976 | 1469 | 1821 | 1066 | 1683 | 1678 | 1421.878096 | 1475.854919 | 1.037961639 | 0.874019974 | unchanged |
| hsa_circ_101163 | 102 | 111 | 108 | 110 | 113 | 108 | 107.0310914 | 110.0332436 | 1.028049347 | 0.395894735 | unchanged |
| hsa_circ_101164 | 39776 | 25983 | 23672 | 33780 | 29019 | 28623 | 29810.05364 | 30474.08177 | 1.022275308 | 0.906222046 | unchanged |
| hsa_circ_101165 | 182 | 191 | 161 | 209 | 225 | 313 | 177.7041349 | 249.1286323 | 1.401929294 | 0.099694173 | unchanged |
| hsa_circ_101166 | 93 | 116 | 96 | 99 | 132 | 146 | 101.5168768 | 125.6620065 | 1.237843504 | 0.20070064 | unchanged |
| hsa_circ_101167 | 155 | 127 | 115 | 132 | 77 | 77 | 132.6517456 | 95.62369508 | 0.720862697 | 0.163965787 | unchanged |
| hsa_circ_101170 | 327 | 245 | 195 | 411 | 206 | 203 | 255.830518 | 273.5738415 | 1.069355774 | 0.832860788 | unchanged |
| hsa_circ_101171 | 233 | 202 | 198 | 324 | 197 | 197 | 211.0637333 | 239.6081637 | 1.13524081 | 0.550300315 | unchanged |
| hsa_circ_101172 | 67 | 69 | 71 | 63 | 74 | 68 | 69.27477466 | 68.14550811 | 0.983698734 | 0.750273578 | unchanged |
| hsa_circ_101173 | 118 | 83 | 109 | 110 | 113 | 111 | 102.9425772 | 111.228862 | 1.080494243 | 0.477207208 | unchanged |
| hsa_circ_101174 | 597 | 1007 | 626 | 598 | 660 | 671 | 743.5861817 | 643.1388493 | 0.864915009 | 0.495476003 | unchanged |
| hsa_circ_101175 | 1080 | 731 | 643 | 913 | 681 | 712 | 817.9575171 | 768.8022848 | 0.939904908 | 0.762852495 | unchanged |
| hsa_circ_101176 | 196 | 247 | 288 | 247 | 386 | 361 | 243.8643417 | 331.316869 | 1.358611377 | 0.156544556 | unchanged |
| hsa_circ_101177 | 692 | 614 | 427 | 745 | 527 | 506 | 577.5590607 | 592.5980512 | 1.026038879 | 0.897494753 | unchanged |
| hsa_circ_101178 | 326 | 368 | 484 | 393 | 409 | 482 | 392.7721176 | 428.2282109 | 1.090271411 | 0.552896294 | unchanged |
| hsa_circ_101179 | 1918 | 2478 | 2400 | 1800 | 2517 | 2398 | 2265.509584 | 2238.415553 | 0.988040646 | 0.928305171 | unchanged |
| hsa_circ_101180 | 84 | 75 | 83 | 60 | 85 | 78 | 80.29348018 | 73.96237693 | 0.921150469 | 0.474614119 | unchanged |
| hsa_circ_101181 | 345 | 255 | 205 | 290 | 226 | 222 | 268.3201415 | 246.0759972 | 0.917098492 | 0.656773504 | unchanged |
| hsa_circ_101182 | 442 | 157 | 142 | 400 | 153 | 147 | 246.7096346 | 233.4338043 | 0.94618844 | 0.922603961 | unchanged |
| hsa_circ_101183 | 319 | 318 | 492 | 314 | 373 | 346 | 376.2340294 | 344.0610801 | 0.914486871 | 0.622700983 | unchanged |
| hsa_circ_101185 | 408 | 331 | 359 | 342 | 370 | 364 | 366.0135707 | 358.9519101 | 0.98070656 | 0.78552463 | unchanged |
| hsa_circ_101186 | 76 | 88 | 85 | 79 | 75 | 75 | 82.93370999 | 76.15318952 | 0.918241684 | 0.147501165 | unchanged |
| hsa_circ_101189 | 130 | 77 | 103 | 111 | 107 | 110 | 103.2479493 | 109.1667544 | 1.057326127 | 0.721622491 | unchanged |
| hsa_circ_101190 | 87 | 55 | 81 | 86 | 94 | 89 | 74.41601855 | 89.41278654 | 1.201526073 | 0.217786717 | unchanged |
| hsa_circ_101193 | 75 | 63 | 85 | 76 | 86 | 86 | 74.23574414 | 82.5942695 | 1.11259435 | 0.316667365 | unchanged |
| hsa_circ_101194 | 100 | 84 | 68 | 103 | 85 | 101 | 84.2010987 | 96.75581479 | 1.149103946 | 0.307877131 | unchanged |
| hsa_circ_101196 | 57 | 78 | 51 | 56 | 51 | 51 | 61.83649605 | 52.71115341 | 0.852427883 | 0.333717847 | unchanged |
| hsa_circ_101197 | 91 | 58 | 89 | 82 | 81 | 81 | 79.54782123 | 81.51135568 | 1.024683699 | 0.861692991 | unchanged |
| hsa_circ_101199 | 122 | 85 | 258 | 110 | 110 | 105 | 154.8735716 | 108.1785962 | 0.698496168 | 0.423628697 | unchanged |
| hsa_circ_101200 | 69 | 89 | 76 | 75 | 69 | 71 | 77.88557226 | 71.79866045 | 0.921848018 | 0.355447822 | unchanged |
| hsa_circ_101201 | 12508 | 17714 | 27020 | 14012 | 25477 | 24585 | 19080.70237 | 21357.90471 | 1.119345834 | 0.706028808 | unchanged |
| hsa_circ_101202 | 205 | 145 | 172 | 222 | 222 | 220 | 173.6896194 | 221.4821717 | 1.275160671 | 0.052077224 | unchanged |
| hsa_circ_101204 | 63 | 149 | 79 | 67 | 73 | 70 | 96.96018763 | 70.10268267 | 0.723004817 | 0.370364342 | unchanged |
| hsa_circ_101205 | 2446 | 1140 | 1413 | 2152 | 924 | 1031 | 1666.430308 | 1369.039246 | 0.821540055 | 0.622812614 | unchanged |
| hsa_circ_101206 | 128 | 209 | 183 | 139 | 195 | 204 | 173.2197862 | 179.4799444 | 1.036139972 | 0.851397063 | unchanged |
| hsa_circ_101207 | 277 | 205 | 215 | 272 | 290 | 288 | 232.3141802 | 283.4694333 | 1.220198582 | 0.0909265 | unchanged |
| hsa_circ_101208 | 81 | 151 | 95 | 65 | 68 | 71 | 108.9832054 | 67.72134119 | 0.621392451 | 0.130153216 | unchanged |
| hsa_circ_101209 | 87 | 85 | 74 | 74 | 70 | 74 | 81.88671132 | 72.72971486 | 0.888174817 | 0.097509758 | unchanged |
| hsa_circ_101210 | 326 | 180 | 151 | 211 | 135 | 143 | 219.1056852 | 163.0729379 | 0.744266118 | 0.398669887 | unchanged |
| hsa_circ_101211 | 178 | 87 | 74 | 130 | 76 | 70 | 112.9948798 | 91.89773592 | 0.813291152 | 0.605474983 | unchanged |
| hsa_circ_101212 | 186 | 117 | 121 | 176 | 142 | 128 | 141.3403857 | 148.4759498 | 1.050484963 | 0.802315445 | unchanged |
| hsa_circ_101214 | 129 | 104 | 108 | 104 | 95 | 96 | 113.9055968 | 98.41615884 | 0.864015129 | 0.129778652 | unchanged |
| hsa_circ_101215 | 899 | 1583 | 1364 | 1015 | 1446 | 1455 | 1282.180147 | 1304.993531 | 1.017792651 | 0.931260567 | unchanged |
| hsa_circ_101216 | 97 | 125 | 91 | 118 | 96 | 104 | 104.1314501 | 106.0639586 | 1.018558356 | 0.880791867 | unchanged |
| hsa_circ_101217 | 108 | 92 | 132 | 119 | 147 | 143 | 110.7935707 | 136.1866261 | 1.2291925 | 0.158567925 | unchanged |
| hsa_circ_101218 | 60 | 112 | 60 | 60 | 60 | 67 | 77.37582682 | 62.47077819 | 0.807368151 | 0.442110453 | unchanged |
| hsa_circ_101219 | 1980 | 2145 | 1944 | 1923 | 1799 | 1624 | 2022.926062 | 1781.635395 | 0.880721955 | 0.086072492 | unchanged |
| hsa_circ_101220 | 410 | 527 | 404 | 379 | 406 | 416 | 447.004996 | 400.0878926 | 0.89504121 | 0.32048537 | unchanged |
| hsa_circ_101221 | 341 | 150 | 194 | 251 | 177 | 191 | 228.2558545 | 206.4560686 | 0.904494078 | 0.742551713 | unchanged |
| hsa_circ_101222 | 218 | 228 | 165 | 208 | 156 | 145 | 203.8694308 | 169.498329 | 0.831406299 | 0.280951313 | unchanged |
| hsa_circ_101223 | 1749 | 1293 | 1418 | 1337 | 1130 | 1243 | 1486.609304 | 1236.769797 | 0.831940036 | 0.167716238 | unchanged |
| hsa_circ_101226 | 3480 | 3123 | 2759 | 2595 | 2781 | 2711 | 3120.385099 | 2695.570288 | 0.863858211 | 0.119397028 | unchanged |
| hsa_circ_101227 | 4006 | 7610 | 9861 | 4215 | 7251 | 7919 | 7158.914947 | 6462.012148 | 0.902652454 | 0.751134625 | unchanged |
| hsa_circ_101228 | 95 | 63 | 51 | 84 | 67 | 55 | 69.87192683 | 68.66831349 | 0.982774007 | 0.942323124 | unchanged |
| hsa_circ_101229 | 1654 | 990 | 1212 | 1154 | 895 | 869 | 1285.443218 | 972.7112948 | 0.756712767 | 0.219945863 | unchanged |
| hsa_circ_101231 | 247 | 352 | 389 | 300 | 576 | 563 | 329.4076831 | 479.6361082 | 1.456056227 | 0.20535651 | unchanged |
| hsa_circ_101232 | 183 | 127 | 135 | 181 | 121 | 124 | 148.374194 | 142.0315271 | 0.957252223 | 0.818658649 | unchanged |
| hsa_circ_101233 | 277 | 313 | 238 | 392 | 298 | 274 | 276.0986632 | 321.3476598 | 1.163887054 | 0.343412961 | unchanged |
| hsa_circ_101234 | 106 | 214 | 132 | 108 | 112 | 120 | 150.7236264 | 113.4276251 | 0.752553716 | 0.316345942 | unchanged |
| hsa_circ_101235 | 105 | 93 | 93 | 106 | 105 | 97 | 97.06344991 | 102.7261924 | 1.058340627 | 0.315488588 | unchanged |
| hsa_circ_101236 | 66 | 79 | 63 | 72 | 63 | 62 | 69.48892607 | 65.75704025 | 0.946295244 | 0.574965849 | unchanged |
| hsa_circ_101237 | 330 | 359 | 453 | 252 | 415 | 414 | 380.6742126 | 360.5710286 | 0.947190581 | 0.774273749 | unchanged |
| hsa_circ_101238 | 80 | 89 | 90 | 88 | 88 | 90 | 86.27171208 | 88.85404881 | 1.029932601 | 0.489555414 | unchanged |
| hsa_circ_101239 | 96 | 70 | 87 | 79 | 80 | 79 | 84.25917084 | 79.25446602 | 0.940603441 | 0.546329687 | unchanged |
| hsa_circ_101240 | 202 | 162 | 145 | 182 | 149 | 162 | 169.6801065 | 164.0577348 | 0.966864874 | 0.785398228 | unchanged |
| hsa_circ_101241 | 68 | 67 | 80 | 67 | 83 | 81 | 71.87730733 | 76.85485504 | 1.069250615 | 0.48644122 | unchanged |
| hsa_circ_101245 | 233 | 316 | 418 | 254 | 473 | 481 | 322.4049145 | 402.6119553 | 1.248777352 | 0.430866212 | unchanged |
| hsa_circ_101246 | 920 | 586 | 474 | 837 | 509 | 509 | 659.9390624 | 618.4636022 | 0.937152591 | 0.822278643 | unchanged |
| hsa_circ_101247 | 1307 | 1138 | 1108 | 1418 | 1211 | 1221 | 1184.597009 | 1282.960375 | 1.083035298 | 0.343095397 | unchanged |
| hsa_circ_101248 | 1301 | 599 | 511 | 1033 | 531 | 514 | 803.4798316 | 692.5843897 | 0.861981051 | 0.732457975 | unchanged |
| hsa_circ_101249 | 358 | 408 | 447 | 375 | 493 | 595 | 404.3200564 | 487.7848235 | 1.206432419 | 0.291176014 | unchanged |
| hsa_circ_101250 | 86 | 109 | 88 | 93 | 81 | 84 | 94.28815233 | 86.17335837 | 0.913936229 | 0.367727905 | unchanged |
| hsa_circ_101251 | 941 | 1466 | 1240 | 1035 | 1357 | 1456 | 1215.79242 | 1282.591478 | 1.054942815 | 0.752730546 | unchanged |
| hsa_circ_101252 | 224 | 317 | 407 | 238 | 461 | 433 | 316.1161617 | 377.486526 | 1.194138648 | 0.522126525 | unchanged |
| hsa_circ_101253 | 927 | 587 | 563 | 658 | 490 | 504 | 692.2520406 | 550.6651427 | 0.795469151 | 0.334869102 | unchanged |
| hsa_circ_101254 | 221 | 324 | 309 | 216 | 283 | 282 | 284.8541431 | 260.3338958 | 0.913919991 | 0.563698858 | unchanged |
| hsa_circ_101255 | 406 | 309 | 265 | 500 | 373 | 340 | 327.0640165 | 404.3372887 | 1.236263448 | 0.294814087 | unchanged |
| hsa_circ_101259 | 287 | 150 | 125 | 214 | 123 | 125 | 187.3478266 | 154.1006913 | 0.822537918 | 0.601381316 | unchanged |
| hsa_circ_101260 | 72 | 73 | 69 | 72 | 81 | 84 | 71.5638264 | 79.16297234 | 1.106186971 | 0.10334508 | unchanged |
| hsa_circ_101261 | 80 | 65 | 64 | 64 | 74 | 72 | 69.84384277 | 70.20189082 | 1.005126408 | 0.954875733 | unchanged |
| hsa_circ_101263 | 302 | 430 | 620 | 349 | 290 | 287 | 450.6243562 | 308.7127688 | 0.685077858 | 0.208526453 | unchanged |
| hsa_circ_101264 | 909 | 593 | 550 | 686 | 613 | 622 | 683.9663377 | 640.2283867 | 0.93605248 | 0.724228135 | unchanged |
| hsa_circ_101268 | 143 | 83 | 77 | 120 | 90 | 80 | 101.0652479 | 96.66704633 | 0.956481564 | 0.866172795 | unchanged |
| hsa_circ_101270 | 60 | 88 | 65 | 58 | 64 | 66 | 70.91082518 | 62.98618388 | 0.888244971 | 0.42675775 | unchanged |
| hsa_circ_101273 | 125 | 91 | 112 | 123 | 94 | 91 | 109.2071789 | 102.7519423 | 0.940889998 | 0.672387279 | unchanged |
| hsa_circ_101274 | 184 | 106 | 124 | 152 | 105 | 123 | 138.0930867 | 126.6699227 | 0.917279248 | 0.696941689 | unchanged |
| hsa_circ_101275 | 23679 | 16799 | 13415 | 20237 | 18749 | 18512 | 17964.45262 | 19166.10223 | 1.06689041 | 0.71524092 | unchanged |
| hsa_circ_101276 | 68 | 43 | 49 | 58 | 47 | 47 | 53.03538373 | 50.77051659 | 0.957295168 | 0.804222654 | unchanged |
| hsa_circ_101277 | 158 | 73 | 103 | 127 | 111 | 104 | 111.6081561 | 114.0910653 | 1.022246664 | 0.928209718 | unchanged |
| hsa_circ_101278 | 2959 | 1322 | 1353 | 1767 | 1223 | 1161 | 1878.041733 | 1383.456983 | 0.73664869 | 0.437477613 | unchanged |
| hsa_circ_101279 | 2302 | 2874 | 2885 | 2519 | 2502 | 2429 | 2687.094998 | 2483.615212 | 0.92427518 | 0.354861754 | unchanged |
| hsa_circ_101280 | 87 | 75 | 113 | 94 | 132 | 126 | 91.64437668 | 117.3403834 | 1.280388253 | 0.18703863 | unchanged |
| hsa_circ_101281 | 61 | 52 | 62 | 61 | 76 | 69 | 58.37638747 | 68.47725749 | 1.173030063 | 0.139280856 | unchanged |
| hsa_circ_101282 | 2098 | 1138 | 1743 | 1345 | 1096 | 1100 | 1659.732809 | 1180.370379 | 0.711180964 | 0.175916069 | unchanged |
| hsa_circ_101283 | 525 | 722 | 585 | 548 | 657 | 716 | 610.6527588 | 640.4332521 | 1.048768294 | 0.715683062 | unchanged |
| hsa_circ_101285 | 139 | 117 | 112 | 163 | 128 | 130 | 122.8438112 | 140.0413393 | 1.139995072 | 0.287889806 | unchanged |
| hsa_circ_101286 | 377 | 233 | 315 | 313 | 170 | 165 | 308.5367 | 215.8076716 | 0.699455435 | 0.220662773 | unchanged |
| hsa_circ_101287 | 2138 | 2444 | 2828 | 3310 | 2981 | 3024 | 2469.591917 | 3105.021257 | 1.257301352 | 0.047516017 | unchanged |
| hsa_circ_101289 | 172 | 130 | 173 | 145 | 230 | 215 | 158.1482321 | 196.9869646 | 1.245584362 | 0.261715527 | unchanged |
| hsa_circ_101290 | 5155 | 4086 | 4484 | 4218 | 4424 | 4350 | 4574.853244 | 4330.87238 | 0.946669139 | 0.485361738 | unchanged |
| hsa_circ_101291 | 509 | 355 | 547 | 622 | 601 | 548 | 470.3817745 | 590.2629557 | 1.254859324 | 0.128506355 | unchanged |
| hsa_circ_101292 | 108 | 85 | 98 | 106 | 120 | 113 | 97.23108945 | 112.8429619 | 1.160564615 | 0.115738785 | unchanged |
| hsa_circ_101293 | 184 | 132 | 150 | 196 | 154 | 176 | 155.5436679 | 175.6199384 | 1.129071603 | 0.363619933 | unchanged |
| hsa_circ_101295 | 221 | 116 | 117 | 171 | 95 | 96 | 151.5365585 | 120.5650568 | 0.795616965 | 0.510849224 | unchanged |
| hsa_circ_101296 | 1152 | 1483 | 834 | 1301 | 1076 | 1112 | 1156.502685 | 1162.82034 | 1.005462724 | 0.976325255 | unchanged |
| hsa_circ_101297 | 69 | 49 | 64 | 67 | 82 | 80 | 60.51251844 | 75.91212296 | 1.25448626 | 0.110790127 | unchanged |
| hsa_circ_101299 | 184 | 122 | 115 | 192 | 125 | 121 | 140.3705025 | 146.004073 | 1.040133578 | 0.868092923 | unchanged |
| hsa_circ_101300 | 79 | 55 | 67 | 72 | 76 | 80 | 66.69505657 | 76.23394466 | 1.143022415 | 0.25970302 | unchanged |
| hsa_circ_101301 | 120 | 118 | 106 | 138 | 113 | 128 | 114.3566529 | 126.400319 | 1.105316707 | 0.228944373 | unchanged |
| hsa_circ_101303 | 339 | 418 | 348 | 396 | 404 | 409 | 368.2658496 | 402.94864 | 1.094178677 | 0.241967041 | unchanged |
| hsa_circ_101305 | 944 | 425 | 360 | 517 | 249 | 246 | 576.1268496 | 337.398665 | 0.5856326 | 0.310283496 | unchanged |
| hsa_circ_101306 | 1129 | 507 | 518 | 730 | 470 | 443 | 717.7078646 | 547.5782031 | 0.762954163 | 0.491624229 | unchanged |
| hsa_circ_101307 | 200 | 205 | 162 | 266 | 202 | 211 | 189.2758921 | 226.1059844 | 1.19458417 | 0.206423344 | unchanged |
| hsa_circ_101308 | 334 | 291 | 269 | 348 | 319 | 319 | 298.1760966 | 328.7353794 | 1.102487366 | 0.225752714 | unchanged |
| hsa_circ_101309 | 471 | 450 | 325 | 613 | 454 | 445 | 415.4507356 | 504.1515617 | 1.21350504 | 0.281116578 | unchanged |
| hsa_circ_101310 | 84 | 96 | 109 | 84 | 123 | 120 | 96.30744017 | 109.3029332 | 1.134937582 | 0.416253258 | unchanged |
| hsa_circ_101311 | 594 | 898 | 872 | 655 | 1085 | 1076 | 787.8540661 | 938.3030248 | 1.190960439 | 0.431075449 | unchanged |
| hsa_circ_101312 | 119 | 162 | 149 | 129 | 179 | 180 | 143.317115 | 162.8489825 | 1.136284264 | 0.40591778 | unchanged |
| hsa_circ_101313 | 6591 | 6712 | 8724 | 6912 | 10314 | 10841 | 7342.22266 | 9355.819211 | 1.274248909 | 0.227038327 | unchanged |
| hsa_circ_101315 | 374 | 233 | 235 | 399 | 154 | 160 | 280.8764703 | 237.8967918 | 0.846980139 | 0.668437469 | unchanged |
| hsa_circ_101316 | 78 | 83 | 65 | 97 | 69 | 72 | 74.91476305 | 79.02997259 | 1.05493189 | 0.712514295 | unchanged |
| hsa_circ_101317 | 218 | 262 | 197 | 212 | 140 | 139 | 225.9067452 | 163.7101592 | 0.724680261 | 0.114493256 | unchanged |
| hsa_circ_101318 | 1243 | 1788 | 2240 | 1397 | 2006 | 1942 | 1756.801948 | 1781.656102 | 1.014147385 | 0.946344086 | unchanged |
| hsa_circ_101319 | 2012 | 3108 | 4345 | 1845 | 2545 | 3583 | 3155.103701 | 2657.666243 | 0.842338793 | 0.586454151 | unchanged |
| hsa_circ_101320 | 1384 | 1470 | 1133 | 1239 | 1226 | 1250 | 1328.83835 | 1238.143405 | 0.9317487 | 0.421425384 | unchanged |
| hsa_circ_101321 | 101 | 96 | 82 | 92 | 81 | 87 | 93.13615856 | 86.63469614 | 0.930194003 | 0.38693813 | unchanged |
| hsa_circ_101322 | 602 | 599 | 715 | 629 | 974 | 896 | 638.4889876 | 833.0322461 | 1.304693209 | 0.155672706 | unchanged |
| hsa_circ_101323 | 430 | 585 | 544 | 430 | 563 | 589 | 519.7265528 | 527.4945663 | 1.014946347 | 0.913932693 | unchanged |
| hsa_circ_101325 | 161 | 207 | 320 | 169 | 123 | 122 | 229.26262 | 137.9116104 | 0.601544248 | 0.140940849 | unchanged |
| hsa_circ_101326 | 93 | 106 | 69 | 84 | 64 | 68 | 89.43391985 | 72.0778987 | 0.805934693 | 0.236025795 | unchanged |
| hsa_circ_101328 | 1037 | 1701 | 1731 | 1190 | 1982 | 1895 | 1489.984546 | 1688.93991 | 1.133528475 | 0.587663735 | unchanged |
| hsa_circ_101329 | 331 | 302 | 367 | 361 | 465 | 461 | 332.9991151 | 428.9995213 | 1.288290274 | 0.069650503 | unchanged |
| hsa_circ_101330 | 79 | 60 | 75 | 71 | 86 | 83 | 71.71089508 | 79.90199696 | 1.114223952 | 0.33251396 | unchanged |
| hsa_circ_101331 | 772 | 503 | 541 | 586 | 454 | 419 | 605.3224924 | 486.2245488 | 0.803248772 | 0.292207093 | unchanged |
| hsa_circ_101332 | 1846 | 2120 | 1666 | 1813 | 1730 | 1735 | 1877.465047 | 1759.330963 | 0.937077878 | 0.429578186 | unchanged |
| hsa_circ_101333 | 133 | 319 | 150 | 143 | 138 | 147 | 200.900487 | 142.5355135 | 0.709483165 | 0.381931127 | unchanged |
| hsa_circ_101335 | 65 | 56 | 64 | 65 | 79 | 81 | 61.38118272 | 75.06348076 | 1.222907045 | 0.076175256 | unchanged |
| hsa_circ_101336 | 10050 | 7386 | 6361 | 8100 | 6876 | 7188 | 7932.361361 | 7387.986435 | 0.931372904 | 0.663042105 | unchanged |
| hsa_circ_101337 | 93 | 77 | 102 | 100 | 131 | 138 | 90.33805228 | 122.8923975 | 1.36036138 | 0.077900864 | unchanged |
| hsa_circ_101338 | 181 | 144 | 203 | 203 | 236 | 242 | 176.1067232 | 226.7833849 | 1.287761085 | 0.072845017 | unchanged |
| hsa_circ_101341 | 97 | 103 | 158 | 92 | 98 | 99 | 119.5234226 | 96.53130956 | 0.807635085 | 0.304524408 | unchanged |
| hsa_circ_101343 | 142 | 115 | 172 | 116 | 162 | 150 | 142.8722058 | 142.817809 | 0.999619263 | 0.99811363 | unchanged |
| hsa_circ_101344 | 135 | 104 | 193 | 103 | 162 | 164 | 144.0185524 | 143.0358668 | 0.993176674 | 0.977609203 | unchanged |
| hsa_circ_101345 | 181 | 170 | 173 | 159 | 180 | 208 | 174.8999873 | 182.4419308 | 1.043121464 | 0.628748569 | unchanged |
| hsa_circ_101347 | 225 | 327 | 402 | 212 | 400 | 398 | 318.1209686 | 336.5330606 | 1.057877644 | 0.830700945 | unchanged |
| hsa_circ_101348 | 248 | 355 | 422 | 239 | 414 | 408 | 341.6249372 | 353.6008567 | 1.035055753 | 0.883225045 | unchanged |
| hsa_circ_101349 | 459 | 544 | 683 | 385 | 727 | 719 | 561.710213 | 610.3553131 | 1.086601772 | 0.727704549 | unchanged |
| hsa_circ_101350 | 66 | 64 | 75 | 63 | 81 | 79 | 68.45506391 | 74.38961135 | 1.0866926 | 0.437132485 | unchanged |
| hsa_circ_101351 | 62 | 77 | 66 | 67 | 71 | 69 | 68.32400928 | 69.03825976 | 1.010453872 | 0.879106934 | unchanged |
| hsa_circ_101352 | 183 | 121 | 127 | 153 | 117 | 134 | 143.5599713 | 134.6449472 | 0.937900349 | 0.710905368 | unchanged |
| hsa_circ_101353 | 3252 | 2736 | 2933 | 2945 | 2747 | 2589 | 2973.88886 | 2760.334534 | 0.928190213 | 0.306015031 | unchanged |
| hsa_circ_101354 | 3136 | 3724 | 3713 | 2739 | 3368 | 3368 | 3524.185384 | 3158.026701 | 0.896101186 | 0.269451543 | unchanged |
| hsa_circ_101355 | 198 | 213 | 170 | 237 | 240 | 224 | 193.9320219 | 233.6129932 | 1.204612786 | 0.042138562 | unchanged |
| hsa_circ_101357 | 88 | 88 | 86 | 90 | 96 | 103 | 87.44364671 | 96.17035359 | 1.099798067 | 0.093028346 | unchanged |
| hsa_circ_101359 | 103 | 63 | 79 | 91 | 86 | 88 | 81.55374816 | 88.43942267 | 1.084431123 | 0.594030161 | unchanged |
| hsa_circ_101360 | 148 | 106 | 175 | 104 | 153 | 150 | 142.7187171 | 135.5535068 | 0.94979488 | 0.793022552 | unchanged |
| hsa_circ_101361 | 66 | 54 | 55 | 58 | 60 | 58 | 58.24470434 | 58.79041811 | 1.009369329 | 0.899565662 | unchanged |
| hsa_circ_101365 | 390 | 468 | 431 | 445 | 509 | 529 | 429.6514472 | 494.1956663 | 1.150224605 | 0.127717956 | unchanged |
| hsa_circ_101366 | 119 | 109 | 138 | 95 | 124 | 111 | 122.1681761 | 110.1250924 | 0.901422088 | 0.379853278 | unchanged |
| hsa_circ_101367 | 1738 | 1290 | 1029 | 1587 | 1356 | 1433 | 1352.470286 | 1458.837032 | 1.078646272 | 0.651264046 | unchanged |
| hsa_circ_101368 | 446 | 175 | 134 | 399 | 126 | 119 | 251.9317176 | 214.7170113 | 0.852282568 | 0.795799169 | unchanged |
| hsa_circ_101370 | 365 | 518 | 385 | 422 | 497 | 513 | 422.6757084 | 477.3196625 | 1.129281038 | 0.382784288 | unchanged |
| hsa_circ_101372 | 776 | 1119 | 913 | 973 | 1420 | 1502 | 935.8567739 | 1298.29617 | 1.387280838 | 0.132231766 | unchanged |
| hsa_circ_101373 | 3452 | 1931 | 2526 | 2435 | 1613 | 1608 | 2636.385684 | 1885.281047 | 0.715100624 | 0.222622665 | unchanged |
| hsa_circ_101375 | 51 | 99 | 59 | 55 | 48 | 46 | 69.57281635 | 49.7288326 | 0.71477389 | 0.26301045 | unchanged |
| hsa_circ_101376 | 88 | 81 | 226 | 87 | 77 | 81 | 131.5366416 | 81.62805911 | 0.620572778 | 0.350463557 | unchanged |
| hsa_circ_101377 | 69 | 83 | 61 | 74 | 63 | 66 | 71.13653301 | 67.73904686 | 0.952239925 | 0.667714575 | unchanged |
| hsa_circ_101378 | 975 | 1229 | 1312 | 993 | 1172 | 1279 | 1172.204331 | 1147.941373 | 0.979301427 | 0.862402551 | unchanged |
| hsa_circ_101380 | 153 | 89 | 110 | 133 | 77 | 77 | 117.5635205 | 95.77582065 | 0.814672955 | 0.459539058 | unchanged |
| hsa_circ_101382 | 244 | 185 | 169 | 223 | 154 | 141 | 199.6103566 | 172.9497668 | 0.866436841 | 0.479033071 | unchanged |
| hsa_circ_101383 | 124 | 80 | 111 | 113 | 101 | 100 | 104.7612712 | 104.6854241 | 0.999276001 | 0.995850624 | unchanged |
| hsa_circ_101384 | 667 | 394 | 421 | 541 | 442 | 458 | 493.7932906 | 480.4930523 | 0.97306517 | 0.892130756 | unchanged |
| hsa_circ_101386 | 111 | 77 | 120 | 111 | 157 | 141 | 102.5202443 | 136.1895732 | 1.328416393 | 0.149517409 | unchanged |
| hsa_circ_101387 | 495 | 157 | 110 | 332 | 142 | 133 | 253.9662776 | 202.2519558 | 0.796373273 | 0.725652216 | unchanged |
| hsa_circ_101388 | 152 | 80 | 90 | 124 | 94 | 89 | 107.2121353 | 102.3011266 | 0.954193537 | 0.854010647 | unchanged |
| hsa_circ_101392 | 202 | 184 | 159 | 205 | 167 | 176 | 181.841067 | 182.6451221 | 1.004421747 | 0.96435516 | unchanged |
| hsa_circ_101393 | 101 | 126 | 102 | 104 | 111 | 107 | 109.6451422 | 107.3991953 | 0.97951622 | 0.802964226 | unchanged |
| hsa_circ_101394 | 81 | 103 | 90 | 87 | 86 | 89 | 91.338955 | 87.20357082 | 0.954724858 | 0.539432821 | unchanged |
| hsa_circ_101395 | 164 | 103 | 115 | 161 | 116 | 107 | 127.4922128 | 127.824986 | 1.002610146 | 0.990009759 | unchanged |
| hsa_circ_101398 | 159 | 151 | 110 | 153 | 96 | 106 | 140.0670127 | 118.1313094 | 0.843391368 | 0.399110133 | unchanged |
| hsa_circ_101399 | 247 | 133 | 162 | 209 | 193 | 175 | 180.3940227 | 192.2690429 | 1.065828236 | 0.756324874 | unchanged |
| hsa_circ_101400 | 147 | 114 | 167 | 137 | 182 | 192 | 142.3803781 | 170.3913854 | 1.196733621 | 0.288534883 | unchanged |
| hsa_circ_101401 | 103 | 79 | 107 | 115 | 112 | 107 | 96.48901062 | 111.3509061 | 1.15402682 | 0.178448464 | unchanged |
| hsa_circ_101402 | 214 | 181 | 191 | 261 | 173 | 154 | 195.3306649 | 196.2718138 | 1.004818235 | 0.979521053 | unchanged |
| hsa_circ_101404 | 318 | 338 | 207 | 231 | 182 | 187 | 287.5085463 | 199.9145139 | 0.695334161 | 0.115670887 | unchanged |
| hsa_circ_101405 | 227 | 172 | 169 | 213 | 166 | 177 | 189.100431 | 185.4884499 | 0.980899139 | 0.886508476 | unchanged |
| hsa_circ_101406 | 79 | 111 | 96 | 84 | 102 | 102 | 95.23193577 | 96.09827647 | 1.009097166 | 0.940211659 | unchanged |
| hsa_circ_101407 | 4564 | 8339 | 10229 | 1369 | 6063 | 3522 | 7710.54907 | 3651.234078 | 0.473537493 | 0.131744615 | unchanged |
| hsa_circ_101409 | 85 | 73 | 60 | 89 | 63 | 67 | 72.32058132 | 72.82444244 | 1.00696705 | 0.965308382 | unchanged |
| hsa_circ_101410 | 127 | 146 | 92 | 126 | 96 | 91 | 121.8331644 | 104.4166324 | 0.857046051 | 0.416431689 | unchanged |
| hsa_circ_101411 | 110 | 70 | 96 | 119 | 108 | 105 | 92.19219326 | 110.7859942 | 1.2016852 | 0.210705374 | unchanged |
| hsa_circ_101412 | 970 | 453 | 673 | 710 | 535 | 501 | 698.7062134 | 581.9819565 | 0.832942294 | 0.513895094 | unchanged |
| hsa_circ_101413 | 190 | 209 | 114 | 162 | 111 | 110 | 170.8405105 | 127.3371766 | 0.745357036 | 0.266249001 | unchanged |
| hsa_circ_101414 | 154 | 229 | 107 | 145 | 102 | 100 | 163.0419363 | 115.7153729 | 0.709727666 | 0.2871273 | unchanged |
| hsa_circ_101416 | 137 | 72 | 55 | 162 | 45 | 47 | 88.19922731 | 84.63137171 | 0.959547768 | 0.942099593 | unchanged |
| hsa_circ_101418 | 98 | 98 | 72 | 86 | 72 | 72 | 89.28951295 | 76.73171257 | 0.859358619 | 0.264668538 | unchanged |
| hsa_circ_101419 | 1629 | 2291 | 1699 | 1826 | 1863 | 1949 | 1873.045797 | 1879.406625 | 1.003395981 | 0.977602023 | unchanged |
| hsa_circ_101420 | 209 | 304 | 286 | 239 | 301 | 296 | 266.3393092 | 278.9416795 | 1.047316974 | 0.740052474 | unchanged |
| hsa_circ_101422 | 110 | 87 | 120 | 113 | 156 | 133 | 105.5742517 | 133.9203914 | 1.26849482 | 0.144944274 | unchanged |
| hsa_circ_101423 | 129 | 86 | 94 | 121 | 108 | 108 | 102.8301363 | 112.1770557 | 1.090896693 | 0.538110874 | unchanged |
| hsa_circ_101424 | 107 | 103 | 105 | 103 | 130 | 120 | 104.9968892 | 117.5659664 | 1.119709043 | 0.183907084 | unchanged |
| hsa_circ_101425 | 214 | 371 | 269 | 302 | 314 | 283 | 284.7916721 | 299.9514832 | 1.05323123 | 0.762331329 | unchanged |
| hsa_circ_101427 | 169 | 97 | 94 | 122 | 95 | 91 | 119.8603734 | 102.955848 | 0.858964853 | 0.554508272 | unchanged |
| hsa_circ_101430 | 88 | 154 | 85 | 85 | 78 | 83 | 108.942941 | 81.94439701 | 0.752177206 | 0.300418043 | unchanged |
| hsa_circ_101431 | 303 | 335 | 704 | 366 | 1001 | 1022 | 447.0825406 | 796.4506699 | 1.781439885 | 0.235916003 | unchanged |
| hsa_circ_101432 | 65 | 70 | 64 | 62 | 60 | 60 | 66.27735637 | 60.73626636 | 0.916395428 | 0.055630745 | unchanged |
| hsa_circ_101433 | 153 | 167 | 194 | 182 | 204 | 202 | 171.3840647 | 196.0614125 | 1.143988578 | 0.15208247 | unchanged |
| hsa_circ_101434 | 299 | 344 | 356 | 309 | 352 | 339 | 333.0787235 | 333.5664625 | 1.001464335 | 0.983028513 | unchanged |
| hsa_circ_101436 | 132 | 124 | 114 | 136 | 115 | 125 | 123.068625 | 125.6034233 | 1.020596625 | 0.767086395 | unchanged |
| hsa_circ_101437 | 824 | 939 | 1001 | 991 | 1364 | 1325 | 921.3765935 | 1226.660019 | 1.331334036 | 0.077757613 | unchanged |
| hsa_circ_101440 | 218 | 109 | 146 | 183 | 136 | 127 | 157.8317211 | 148.7197043 | 0.94226752 | 0.814832976 | unchanged |
| hsa_circ_101441 | 15182 | 14718 | 17762 | 14791 | 16466 | 16260 | 15887.27772 | 15839.23776 | 0.996976199 | 0.966765618 | unchanged |
| hsa_circ_101444 | 339 | 293 | 191 | 399 | 285 | 297 | 274.5862272 | 326.9858648 | 1.190831267 | 0.40721365 | unchanged |
| hsa_circ_101445 | 168 | 181 | 209 | 173 | 240 | 237 | 186.3854522 | 216.4691986 | 1.161406086 | 0.296923886 | unchanged |
| hsa_circ_101446 | 228 | 228 | 301 | 211 | 275 | 283 | 252.1961947 | 256.4342841 | 1.016804732 | 0.905193669 | unchanged |
| hsa_circ_101447 | 61 | 110 | 65 | 71 | 70 | 72 | 78.52262162 | 70.9121712 | 0.903079517 | 0.652018551 | unchanged |
| hsa_circ_101448 | 154 | 283 | 206 | 164 | 190 | 192 | 214.4412635 | 182.1303283 | 0.84932501 | 0.449884095 | unchanged |
| hsa_circ_101450 | 375 | 532 | 490 | 322 | 425 | 428 | 465.3004383 | 391.5605159 | 0.841521915 | 0.275868517 | unchanged |
| hsa_circ_101456 | 159 | 218 | 216 | 177 | 251 | 255 | 197.4462921 | 227.451677 | 1.151967325 | 0.40073559 | unchanged |
| hsa_circ_101458 | 114 | 164 | 180 | 122 | 207 | 205 | 152.3994178 | 177.9098524 | 1.167391942 | 0.496038285 | unchanged |
| hsa_circ_101459 | 133 | 75 | 91 | 160 | 109 | 108 | 99.87207924 | 125.5462344 | 1.257070398 | 0.350585156 | unchanged |
| hsa_circ_101460 | 64 | 53 | 51 | 70 | 57 | 55 | 56.13361404 | 60.69328584 | 1.081228901 | 0.501233639 | unchanged |
| hsa_circ_101462 | 88 | 78 | 121 | 85 | 118 | 119 | 95.75736228 | 107.4853967 | 1.122476582 | 0.528347335 | unchanged |
| hsa_circ_101463 | 107 | 118 | 119 | 110 | 146 | 150 | 114.4881647 | 135.3856964 | 1.182530061 | 0.185306821 | unchanged |
| hsa_circ_101465 | 2162 | 1758 | 1612 | 2019 | 1658 | 1519 | 1843.816177 | 1732.03386 | 0.939374478 | 0.640733723 | unchanged |
| hsa_circ_101466 | 122 | 116 | 132 | 135 | 119 | 131 | 123.4624394 | 128.2469432 | 1.038752708 | 0.511643566 | unchanged |
| hsa_circ_101467 | 416 | 402 | 378 | 455 | 433 | 417 | 398.6171153 | 434.8653058 | 1.090934857 | 0.082345991 | unchanged |
| hsa_circ_101468 | 65 | 63 | 54 | 66 | 49 | 48 | 60.6574465 | 54.22352129 | 0.893930167 | 0.396349999 | unchanged |
| hsa_circ_101469 | 499 | 627 | 544 | 447 | 605 | 466 | 556.7066998 | 505.8784997 | 0.908698422 | 0.460809185 | unchanged |
| hsa_circ_101470 | 837 | 1110 | 1029 | 1104 | 1246 | 1270 | 992.1979895 | 1206.869616 | 1.216359667 | 0.089223656 | unchanged |
| hsa_circ_101471 | 216 | 291 | 267 | 277 | 505 | 527 | 258.0951699 | 436.0508275 | 1.689496273 | 0.098656092 | unchanged |
| hsa_circ_101472 | 90 | 84 | 73 | 102 | 91 | 87 | 82.42979918 | 93.45154697 | 1.13371072 | 0.179306636 | unchanged |
| hsa_circ_101473 | 85 | 95 | 76 | 88 | 78 | 86 | 85.58707048 | 83.75736612 | 0.97862172 | 0.787720439 | unchanged |
| hsa_circ_101474 | 420 | 575 | 595 | 387 | 618 | 549 | 529.8677104 | 517.7971129 | 0.977219602 | 0.897619388 | unchanged |
| hsa_circ_101475 | 152 | 158 | 386 | 134 | 99 | 106 | 232.0884545 | 113.0800055 | 0.487228051 | 0.201547043 | unchanged |
| hsa_circ_101476 | 390 | 188 | 152 | 242 | 140 | 137 | 243.1625771 | 173.0468717 | 0.71165092 | 0.44029455 | unchanged |
| hsa_circ_101478 | 594 | 554 | 326 | 853 | 341 | 410 | 491.3613605 | 534.4923523 | 1.087778558 | 0.823089259 | unchanged |
| hsa_circ_101479 | 54 | 115 | 56 | 62 | 61 | 64 | 75.29742198 | 62.27413253 | 0.827042027 | 0.549437213 | unchanged |
| hsa_circ_101481 | 101 | 88 | 101 | 93 | 106 | 105 | 96.59514844 | 101.6012712 | 1.051825819 | 0.454714954 | unchanged |
| hsa_circ_101483 | 91 | 86 | 118 | 92 | 135 | 138 | 98.12479133 | 121.850602 | 1.241792215 | 0.251814553 | unchanged |
| hsa_circ_101484 | 101 | 88 | 113 | 107 | 139 | 145 | 100.6153184 | 130.5233185 | 1.297250961 | 0.095288218 | unchanged |
| hsa_circ_101485 | 68 | 72 | 85 | 76 | 77 | 75 | 74.91739666 | 76.06825784 | 1.015361735 | 0.834504756 | unchanged |
| hsa_circ_101488 | 104 | 81 | 91 | 108 | 98 | 100 | 92.1763135 | 102.220401 | 1.108966036 | 0.246957067 | unchanged |
| hsa_circ_101489 | 69 | 59 | 65 | 70 | 77 | 97 | 64.32413385 | 81.72046889 | 1.270448026 | 0.113211581 | unchanged |
| hsa_circ_101490 | 158 | 103 | 124 | 155 | 156 | 156 | 127.9280484 | 155.7527518 | 1.217502758 | 0.157106091 | unchanged |
| hsa_circ_101491 | 34040 | 35266 | 37571 | 34256 | 27342 | 26958 | 35625.62644 | 29518.63344 | 0.828578649 | 0.077645644 | unchanged |
| hsa_circ_101492 | 1440 | 3333 | 3440 | 1593 | 2845 | 2628 | 2737.802831 | 2355.231292 | 0.860263297 | 0.639367704 | unchanged |
| hsa_circ_101493 | 193 | 146 | 198 | 205 | 217 | 225 | 179.1703092 | 215.7823529 | 1.204342136 | 0.103265711 | unchanged |
| hsa_circ_101495 | 788 | 969 | 758 | 742 | 874 | 884 | 838.443728 | 833.0292437 | 0.993542221 | 0.94945534 | unchanged |
| hsa_circ_101496 | 73 | 56 | 83 | 69 | 83 | 83 | 70.47026082 | 78.27866927 | 1.110804308 | 0.442617527 | unchanged |
| hsa_circ_101498 | 69 | 69 | 80 | 72 | 74 | 78 | 72.69194787 | 74.57306001 | 1.025877861 | 0.681048965 | unchanged |
| hsa_circ_101499 | 76 | 85 | 94 | 66 | 81 | 80 | 84.89604812 | 75.80263578 | 0.892887684 | 0.276404079 | unchanged |
| hsa_circ_101501 | 281 | 326 | 361 | 320 | 480 | 455 | 322.6526966 | 418.1004526 | 1.295821969 | 0.157009486 | unchanged |
| hsa_circ_101503 | 3050 | 1010 | 488 | 2966 | 653 | 682 | 1515.849936 | 1433.930544 | 0.945958112 | 0.943944384 | unchanged |
| hsa_circ_101504 | 289 | 192 | 175 | 231 | 163 | 168 | 218.781279 | 187.4976299 | 0.857009479 | 0.494467292 | unchanged |
| hsa_circ_101505 | 98 | 78 | 135 | 107 | 108 | 106 | 103.7687927 | 106.9508752 | 1.03066512 | 0.857580137 | unchanged |
| hsa_circ_101506 | 94 | 69 | 78 | 92 | 86 | 83 | 80.20868459 | 87.14435628 | 1.086470333 | 0.432914325 | unchanged |
| hsa_circ_101508 | 155 | 152 | 156 | 168 | 154 | 180 | 154.3438058 | 167.4474792 | 1.08489925 | 0.167740969 | unchanged |
| hsa_circ_101509 | 883 | 1727 | 1383 | 963 | 1311 | 1428 | 1331.214597 | 1234.051378 | 0.927011604 | 0.747793414 | unchanged |
| hsa_circ_101510 | 398 | 550 | 480 | 383 | 527 | 444 | 475.8565072 | 451.2000384 | 0.948185076 | 0.704733971 | unchanged |
| hsa_circ_101511 | 203 | 134 | 150 | 142 | 122 | 122 | 162.2277721 | 128.3897395 | 0.791416524 | 0.193265942 | unchanged |
| hsa_circ_101512 | 686 | 970 | 713 | 849 | 923 | 1195 | 789.651574 | 989.1749437 | 1.252672668 | 0.224052593 | unchanged |
| hsa_circ_101513 | 71 | 70 | 73 | 70 | 70 | 70 | 71.34233412 | 69.97246302 | 0.980798622 | 0.188108237 | unchanged |
| hsa_circ_101514 | 65 | 55 | 69 | 74 | 65 | 69 | 63.02265473 | 69.31043007 | 1.099770081 | 0.263163509 | unchanged |
| hsa_circ_101515 | 121 | 78 | 74 | 123 | 76 | 76 | 90.95649764 | 91.57923545 | 1.006846546 | 0.978607409 | unchanged |
| hsa_circ_101516 | 83 | 78 | 76 | 91 | 77 | 77 | 78.87415205 | 81.71508432 | 1.036018546 | 0.612443794 | unchanged |
| hsa_circ_101518 | 465 | 331 | 390 | 360 | 261 | 270 | 395.2870028 | 297.1060304 | 0.751621046 | 0.121921405 | unchanged |
| hsa_circ_101519 | 94 | 72 | 81 | 105 | 96 | 104 | 82.26191956 | 101.7265171 | 1.236617352 | 0.052470414 | unchanged |
| hsa_circ_101520 | 69 | 57 | 72 | 72 | 72 | 71 | 65.86352818 | 71.55077752 | 1.086348993 | 0.274749199 | unchanged |
| hsa_circ_101521 | 108 | 76 | 85 | 101 | 105 | 92 | 89.66872999 | 99.11987889 | 1.105400722 | 0.411476423 | unchanged |
| hsa_circ_101522 | 329 | 258 | 340 | 224 | 270 | 261 | 308.9593351 | 251.6704286 | 0.814574606 | 0.123401998 | unchanged |
| hsa_circ_101523 | 693 | 1037 | 1101 | 795 | 1351 | 1337 | 943.7283627 | 1160.832099 | 1.230048968 | 0.38469002 | unchanged |
| hsa_circ_101524 | 292 | 209 | 199 | 303 | 170 | 165 | 233.375592 | 212.8648253 | 0.912112631 | 0.723267022 | unchanged |
| hsa_circ_101525 | 8128 | 5827 | 4716 | 9661 | 3799 | 3712 | 6223.686979 | 5723.911909 | 0.91969791 | 0.832189736 | unchanged |
| hsa_circ_101527 | 620 | 740 | 626 | 616 | 751 | 709 | 662.057841 | 692.0386158 | 1.045284223 | 0.619820144 | unchanged |
| hsa_circ_101529 | 88 | 55 | 70 | 91 | 83 | 88 | 70.91727933 | 87.27600353 | 1.230673319 | 0.169789031 | unchanged |
| hsa_circ_101530 | 62 | 59 | 54 | 56 | 52 | 55 | 58.21375351 | 54.72741066 | 0.940111354 | 0.235602906 | unchanged |
| hsa_circ_101531 | 238 | 96 | 79 | 108 | 77 | 73 | 137.7597788 | 86.07104449 | 0.624790815 | 0.37425861 | unchanged |
| hsa_circ_101533 | 289 | 450 | 483 | 310 | 335 | 344 | 407.5376603 | 329.3921735 | 0.808249655 | 0.26693727 | unchanged |
| hsa_circ_101534 | 810 | 847 | 1118 | 818 | 1149 | 1132 | 925.187646 | 1033.022421 | 1.11655449 | 0.498047459 | unchanged |
| hsa_circ_101535 | 547 | 604 | 508 | 566 | 688 | 652 | 553.1342507 | 635.3522101 | 1.148640153 | 0.146722891 | unchanged |
| hsa_circ_101536 | 259 | 199 | 223 | 294 | 239 | 226 | 227.1107809 | 253.0960561 | 1.11441674 | 0.394577184 | unchanged |
| hsa_circ_101537 | 136 | 178 | 279 | 158 | 112 | 116 | 197.7980112 | 128.4967437 | 0.649636176 | 0.198847683 | unchanged |
| hsa_circ_101538 | 466 | 269 | 255 | 406 | 251 | 251 | 330.2872254 | 302.816433 | 0.916827566 | 0.763740926 | unchanged |
| hsa_circ_101539 | 722 | 957 | 1078 | 786 | 977 | 1018 | 919.2808728 | 926.9351028 | 1.008326324 | 0.954722863 | unchanged |
| hsa_circ_101540 | 157 | 136 | 112 | 146 | 95 | 105 | 134.8692478 | 115.3434575 | 0.855224296 | 0.383971258 | unchanged |
| hsa_circ_101541 | 586 | 646 | 732 | 678 | 701 | 776 | 654.5113063 | 718.2967735 | 1.097455104 | 0.283506506 | unchanged |
| hsa_circ_101542 | 334 | 168 | 187 | 371 | 247 | 252 | 229.3206496 | 290.3085914 | 1.265950502 | 0.40920779 | unchanged |
| hsa_circ_101543 | 150 | 143 | 146 | 139 | 157 | 160 | 146.1638189 | 152.1201475 | 1.040751047 | 0.429667657 | unchanged |
| hsa_circ_101544 | 124 | 154 | 133 | 112 | 128 | 139 | 136.9764148 | 126.2960045 | 0.922027377 | 0.42259922 | unchanged |
| hsa_circ_101545 | 97 | 106 | 129 | 89 | 165 | 122 | 110.3476517 | 125.4823827 | 1.13715499 | 0.558819275 | unchanged |
| hsa_circ_101546 | 71 | 66 | 223 | 67 | 74 | 73 | 119.8969832 | 71.30050151 | 0.594681364 | 0.39990865 | unchanged |
| hsa_circ_101547 | 100 | 64 | 102 | 75 | 107 | 108 | 88.71882529 | 96.80085377 | 1.091097109 | 0.647583017 | unchanged |
| hsa_circ_101548 | 108 | 63 | 112 | 93 | 118 | 114 | 94.44965921 | 108.5728467 | 1.149531376 | 0.46997678 | unchanged |
| hsa_circ_101549 | 61 | 51 | 68 | 62 | 68 | 64 | 60.09040618 | 64.66543607 | 1.076135779 | 0.410246612 | unchanged |
| hsa_circ_101551 | 61 | 46 | 57 | 59 | 61 | 59 | 54.70550024 | 59.4664385 | 1.087028512 | 0.362224189 | unchanged |
| hsa_circ_101552 | 95 | 64 | 83 | 99 | 86 | 89 | 80.55669306 | 91.23546666 | 1.132562214 | 0.331458702 | unchanged |
| hsa_circ_101553 | 70 | 80 | 73 | 68 | 74 | 79 | 74.38581249 | 73.81662787 | 0.99234821 | 0.903005322 | unchanged |
| hsa_circ_101554 | 95 | 94 | 110 | 89 | 104 | 114 | 99.82936059 | 102.4375713 | 1.026126689 | 0.788278626 | unchanged |
| hsa_circ_101555 | 2433 | 3626 | 2571 | 5469 | 3531 | 3818 | 2876.550736 | 4272.780465 | 1.485383314 | 0.121325234 | unchanged |
| hsa_circ_101556 | 591 | 354 | 262 | 463 | 201 | 220 | 402.3591282 | 294.8713267 | 0.732856063 | 0.453101003 | unchanged |
| hsa_circ_101559 | 331 | 232 | 297 | 311 | 379 | 362 | 286.5859989 | 350.6454149 | 1.223525979 | 0.145691267 | unchanged |
| hsa_circ_101560 | 1006 | 1277 | 1260 | 925 | 1277 | 1304 | 1180.767875 | 1168.675576 | 0.989758953 | 0.939735104 | unchanged |
| hsa_circ_101562 | 119 | 71 | 118 | 128 | 174 | 176 | 102.5011827 | 159.1084086 | 1.552259247 | 0.063248029 | unchanged |
| hsa_circ_101563 | 150 | 104 | 154 | 170 | 199 | 199 | 135.9240666 | 189.3232112 | 1.392860116 | 0.047294571 | unchanged |
| hsa_circ_101564 | 74 | 49 | 69 | 87 | 110 | 82 | 64.02289166 | 92.7612006 | 1.448875522 | 0.066624538 | unchanged |
| hsa_circ_101565 | 166 | 87 | 98 | 151 | 114 | 112 | 117.1347218 | 126.1004513 | 1.076542031 | 0.761620505 | unchanged |
| hsa_circ_101566 | 100 | 105 | 130 | 128 | 148 | 153 | 111.6201904 | 142.9495064 | 1.280677859 | 0.058298232 | unchanged |
| hsa_circ_101568 | 109 | 73 | 105 | 101 | 97 | 70 | 95.40454242 | 89.41276705 | 0.937196121 | 0.710292959 | unchanged |
| hsa_circ_101569 | 93 | 121 | 87 | 89 | 83 | 81 | 100.0198301 | 84.37262877 | 0.843559009 | 0.214505316 | unchanged |
| hsa_circ_101571 | 5806 | 12000 | 13942 | 6286 | 12687 | 14010 | 10582.79407 | 10994.26083 | 1.038880731 | 0.910071589 | unchanged |
| hsa_circ_101574 | 77 | 74 | 68 | 77 | 78 | 74 | 72.98761348 | 76.30861032 | 1.045500828 | 0.340438986 | unchanged |
| hsa_circ_101575 | 305 | 223 | 227 | 475 | 350 | 329 | 251.8929468 | 384.685803 | 1.527179732 | 0.065521691 | unchanged |
| hsa_circ_101579 | 401 | 790 | 645 | 378 | 611 | 530 | 611.7899901 | 506.1711847 | 0.827361011 | 0.470045033 | unchanged |
| hsa_circ_101580 | 163 | 226 | 197 | 155 | 157 | 135 | 195.2715629 | 148.7877209 | 0.761952835 | 0.076191702 | unchanged |
| hsa_circ_101581 | 120 | 116 | 140 | 109 | 135 | 132 | 125.5804289 | 125.1951005 | 0.996931621 | 0.9733566 | unchanged |
| hsa_circ_101582 | 85 | 104 | 86 | 82 | 75 | 80 | 91.46204654 | 79.00017565 | 0.863748174 | 0.133638291 | unchanged |
| hsa_circ_101584 | 205 | 314 | 276 | 237 | 252 | 261 | 264.8314575 | 249.8690325 | 0.943502086 | 0.670857623 | unchanged |
| hsa_circ_101585 | 90 | 71 | 89 | 82 | 98 | 96 | 83.23121758 | 91.85780101 | 1.103646008 | 0.337024016 | unchanged |
| hsa_circ_101586 | 354 | 266 | 467 | 302 | 271 | 265 | 362.4392397 | 279.1284167 | 0.770138512 | 0.231862602 | unchanged |
| hsa_circ_101589 | 1138 | 604 | 891 | 649 | 499 | 496 | 877.5823024 | 547.737704 | 0.624143972 | 0.111780971 | unchanged |
| hsa_circ_101590 | 385 | 629 | 482 | 418 | 425 | 476 | 498.5403978 | 439.709106 | 0.88199293 | 0.466157196 | unchanged |
| hsa_circ_101591 | 377 | 331 | 381 | 461 | 376 | 367 | 362.7800033 | 401.0962641 | 1.105618448 | 0.322189568 | unchanged |
| hsa_circ_101592 | 326 | 595 | 691 | 442 | 866 | 902 | 537.5839647 | 736.3831599 | 1.3698012 | 0.340261429 | unchanged |
| hsa_circ_101593 | 159 | 170 | 128 | 165 | 127 | 128 | 152.2864709 | 140.1859189 | 0.92054086 | 0.534277933 | unchanged |
| hsa_circ_101594 | 66 | 65 | 80 | 74 | 66 | 69 | 70.35663068 | 69.68477224 | 0.990450673 | 0.909081388 | unchanged |
| hsa_circ_101595 | 685 | 679 | 405 | 858 | 553 | 571 | 589.4928943 | 660.4400681 | 1.12035289 | 0.627804835 | unchanged |
| hsa_circ_101596 | 252 | 303 | 261 | 299 | 326 | 324 | 271.9347335 | 316.275689 | 1.163057344 | 0.068286096 | unchanged |
| hsa_circ_101597 | 139 | 178 | 168 | 133 | 141 | 151 | 161.877016 | 141.9204211 | 0.876717552 | 0.193513248 | unchanged |
| hsa_circ_101598 | 114 | 137 | 158 | 133 | 194 | 194 | 136.4528623 | 173.7557494 | 1.273375629 | 0.193751928 | unchanged |
| hsa_circ_101600 | 233 | 149 | 177 | 203 | 166 | 159 | 186.1461604 | 175.9039907 | 0.944977808 | 0.733174744 | unchanged |
| hsa_circ_101602 | 188 | 247 | 185 | 244 | 238 | 265 | 206.6051544 | 248.8970634 | 1.204699196 | 0.122808334 | unchanged |
| hsa_circ_101603 | 77 | 82 | 71 | 77 | 80 | 80 | 76.76256924 | 79.37772306 | 1.034068086 | 0.492162878 | unchanged |
| hsa_circ_101604 | 152 | 135 | 271 | 176 | 172 | 185 | 185.6744221 | 177.3693186 | 0.95527061 | 0.855864121 | unchanged |
| hsa_circ_101607 | 82 | 94 | 85 | 88 | 84 | 87 | 86.97421251 | 86.11978214 | 0.990176049 | 0.843285601 | unchanged |
| hsa_circ_101608 | 103 | 98 | 129 | 108 | 131 | 138 | 110.1839856 | 125.5421897 | 1.139386899 | 0.310296914 | unchanged |
| hsa_circ_101609 | 132 | 92 | 97 | 119 | 101 | 97 | 107.1364951 | 105.7475482 | 0.987035726 | 0.927761407 | unchanged |
| hsa_circ_101610 | 203 | 161 | 181 | 167 | 185 | 188 | 181.7372212 | 179.8967927 | 0.989873134 | 0.899853489 | unchanged |
| hsa_circ_101612 | 100 | 233 | 111 | 105 | 115 | 116 | 148.3458721 | 112.1175779 | 0.755784953 | 0.444142703 | unchanged |
| hsa_circ_101615 | 408 | 289 | 228 | 414 | 289 | 270 | 308.2808908 | 324.2789723 | 1.051894496 | 0.829395634 | unchanged |
| hsa_circ_101617 | 84 | 93 | 73 | 86 | 73 | 75 | 83.48565717 | 78.0011268 | 0.934305717 | 0.487375229 | unchanged |
| hsa_circ_101618 | 1092 | 450 | 443 | 759 | 388 | 367 | 661.6847591 | 504.9553763 | 0.763135873 | 0.565073661 | unchanged |
| hsa_circ_101621 | 124 | 95 | 99 | 122 | 101 | 102 | 105.9900584 | 108.3458213 | 1.022226262 | 0.84615555 | unchanged |
| hsa_circ_101622 | 87 | 122 | 79 | 83 | 81 | 83 | 95.94439629 | 82.53458649 | 0.860233528 | 0.36776907 | unchanged |
| hsa_circ_101623 | 301 | 83 | 112 | 236 | 132 | 130 | 165.2603291 | 166.1566344 | 1.005423597 | 0.991256773 | unchanged |
| hsa_circ_101625 | 73 | 67 | 92 | 63 | 78 | 82 | 77.68422254 | 74.52840849 | 0.959376384 | 0.756436584 | unchanged |
| hsa_circ_101626 | 73 | 84 | 81 | 77 | 77 | 75 | 79.62627575 | 76.5735495 | 0.961661823 | 0.414536522 | unchanged |
| hsa_circ_101627 | 135 | 119 | 158 | 161 | 185 | 169 | 137.6776461 | 171.502445 | 1.245681124 | 0.065351362 | unchanged |
| hsa_circ_101628 | 78 | 64 | 66 | 72 | 70 | 60 | 69.22038405 | 67.20181354 | 0.970838496 | 0.74833955 | unchanged |
| hsa_circ_101629 | 92 | 68 | 75 | 91 | 78 | 77 | 78.23304059 | 81.82994086 | 1.045976741 | 0.688746484 | unchanged |
| hsa_circ_101630 | 221 | 268 | 281 | 236 | 371 | 317 | 256.6517814 | 307.6595842 | 1.198743225 | 0.303863322 | unchanged |
| hsa_circ_101631 | 225 | 313 | 238 | 192 | 211 | 216 | 258.9016614 | 206.3660803 | 0.797082874 | 0.137905121 | unchanged |
| hsa_circ_101632 | 229 | 231 | 210 | 163 | 212 | 200 | 223.0519385 | 191.6703724 | 0.859308256 | 0.12577118 | unchanged |
| hsa_circ_101633 | 183 | 249 | 233 | 159 | 200 | 185 | 221.568973 | 181.2020496 | 0.817813285 | 0.157353222 | unchanged |
| hsa_circ_101634 | 218 | 369 | 311 | 243 | 357 | 387 | 299.3315742 | 328.7544566 | 1.098295285 | 0.659922338 | unchanged |
| hsa_circ_101636 | 64 | 74 | 80 | 69 | 78 | 76 | 72.62889826 | 74.61676073 | 1.027370131 | 0.730768614 | unchanged |
| hsa_circ_101637 | 156 | 131 | 173 | 148 | 176 | 173 | 153.5192979 | 165.9988645 | 1.081289888 | 0.45221111 | unchanged |
| hsa_circ_101638 | 101 | 77 | 130 | 95 | 135 | 128 | 102.8062904 | 118.8650022 | 1.156203591 | 0.45898071 | unchanged |
| hsa_circ_101639 | 453 | 462 | 529 | 468 | 568 | 581 | 481.4163363 | 539.1235675 | 1.119869699 | 0.252767497 | unchanged |
| hsa_circ_101640 | 67 | 76 | 65 | 75 | 74 | 65 | 69.56791086 | 71.42911707 | 1.026753803 | 0.695228668 | unchanged |
| hsa_circ_101641 | 56 | 66 | 76 | 67 | 79 | 78 | 65.74469097 | 74.55116452 | 1.133949577 | 0.272156894 | unchanged |
| hsa_circ_101642 | 594 | 558 | 461 | 598 | 558 | 552 | 537.8368394 | 569.1515915 | 1.058223516 | 0.499975354 | unchanged |
| hsa_circ_101643 | 737 | 579 | 593 | 549 | 450 | 428 | 636.1979014 | 475.9253385 | 0.748077505 | 0.06289526 | unchanged |
| hsa_circ_101644 | 1304 | 1230 | 1155 | 980 | 1251 | 1163 | 1229.627896 | 1131.299962 | 0.920034399 | 0.338526143 | unchanged |
| hsa_circ_101645 | 147 | 122 | 107 | 164 | 156 | 154 | 125.3045365 | 158.090659 | 1.26165152 | 0.053183557 | unchanged |
| hsa_circ_101647 | 126 | 217 | 173 | 139 | 155 | 152 | 171.8749651 | 148.5540715 | 0.864314773 | 0.429740483 | unchanged |
| hsa_circ_101648 | 68 | 61 | 92 | 61 | 93 | 95 | 73.65775061 | 83.11765444 | 1.128430528 | 0.545004226 | unchanged |
| hsa_circ_101649 | 101 | 94 | 116 | 111 | 123 | 114 | 104.0199859 | 116.2177068 | 1.117263243 | 0.173263619 | unchanged |
| hsa_circ_101651 | 411 | 627 | 495 | 436 | 568 | 569 | 510.9290162 | 524.142201 | 1.025861097 | 0.87231052 | unchanged |
| hsa_circ_101653 | 66 | 73 | 69 | 71 | 74 | 75 | 69.03350001 | 73.5448227 | 1.06534976 | 0.122656182 | unchanged |
| hsa_circ_101655 | 104 | 87 | 124 | 107 | 116 | 120 | 104.9457045 | 114.3496746 | 1.089607956 | 0.458109184 | unchanged |
| hsa_circ_101656 | 1298 | 835 | 692 | 1336 | 906 | 852 | 941.6782505 | 1031.297405 | 1.095169612 | 0.726057409 | unchanged |
| hsa_circ_101657 | 69 | 122 | 72 | 72 | 64 | 69 | 87.77655075 | 68.2336341 | 0.777356065 | 0.322987542 | unchanged |
| hsa_circ_101659 | 73 | 82 | 84 | 69 | 85 | 76 | 79.8690067 | 76.6991253 | 0.960311496 | 0.605319761 | unchanged |
| hsa_circ_101660 | 78 | 72 | 95 | 79 | 109 | 111 | 81.79002106 | 99.81571619 | 1.220389907 | 0.220459445 | unchanged |
| hsa_circ_101661 | 293 | 185 | 177 | 288 | 216 | 244 | 218.6096983 | 249.1722784 | 1.139804319 | 0.515531648 | unchanged |
| hsa_circ_101663 | 95 | 78 | 88 | 84 | 88 | 92 | 87.10918384 | 87.79007481 | 1.007816523 | 0.907279334 | unchanged |
| hsa_circ_101664 | 88 | 81 | 66 | 85 | 66 | 67 | 77.98008115 | 72.68585946 | 0.932108025 | 0.588728695 | unchanged |
| hsa_circ_101666 | 117 | 128 | 145 | 104 | 153 | 152 | 130.0131405 | 136.1491992 | 1.047195681 | 0.751937464 | unchanged |
| hsa_circ_101668 | 67 | 66 | 61 | 60 | 77 | 59 | 64.71202422 | 65.32167605 | 1.009420998 | 0.923411441 | unchanged |
| hsa_circ_101669 | 68 | 150 | 90 | 67 | 84 | 80 | 102.5660944 | 76.96347488 | 0.750379308 | 0.361577006 | unchanged |
| hsa_circ_101670 | 587 | 884 | 1030 | 651 | 1297 | 1309 | 833.9605838 | 1085.753704 | 1.301924486 | 0.377066734 | unchanged |
| hsa_circ_101671 | 102 | 88 | 116 | 84 | 112 | 95 | 101.7587086 | 96.88226681 | 0.952078384 | 0.694250131 | unchanged |
| hsa_circ_101672 | 248 | 320 | 259 | 230 | 225 | 203 | 275.4946182 | 219.2044947 | 0.795676141 | 0.077434927 | unchanged |
| hsa_circ_101673 | 103 | 131 | 129 | 104 | 130 | 131 | 121.114611 | 121.5172059 | 1.003324082 | 0.97608766 | unchanged |
| hsa_circ_101674 | 952 | 865 | 880 | 875 | 970 | 989 | 899.1625697 | 944.6392327 | 1.050576686 | 0.362286699 | unchanged |
| hsa_circ_101675 | 135 | 243 | 232 | 128 | 185 | 187 | 203.3002091 | 166.505485 | 0.819012857 | 0.403700421 | unchanged |
| hsa_circ_101677 | 75 | 147 | 64 | 73 | 55 | 54 | 95.30849399 | 60.48469432 | 0.634620187 | 0.265063801 | unchanged |
| hsa_circ_101678 | 418 | 495 | 455 | 481 | 500 | 442 | 456.0485303 | 474.4589856 | 1.040369509 | 0.547740265 | unchanged |
| hsa_circ_101679 | 73 | 58 | 67 | 72 | 70 | 71 | 66.06819351 | 71.02072923 | 1.074960968 | 0.332598157 | unchanged |
| hsa_circ_101682 | 506 | 399 | 455 | 391 | 421 | 421 | 453.3469675 | 411.0514654 | 0.906703904 | 0.265256629 | unchanged |
| hsa_circ_101683 | 193 | 279 | 248 | 208 | 248 | 246 | 240.1838876 | 234.1697115 | 0.974960119 | 0.8421579 | unchanged |
| hsa_circ_101684 | 719 | 546 | 363 | 774 | 398 | 402 | 542.5242855 | 524.850782 | 0.967423572 | 0.918251207 | unchanged |
| hsa_circ_101685 | 68 | 137 | 58 | 74 | 60 | 61 | 87.6582556 | 65.08173186 | 0.742448403 | 0.423105594 | unchanged |
| hsa_circ_101687 | 59 | 69 | 50 | 60 | 47 | 52 | 59.48087474 | 53.09009789 | 0.89255745 | 0.382525885 | unchanged |
| hsa_circ_101688 | 333 | 511 | 502 | 313 | 488 | 502 | 448.7289532 | 433.9551793 | 0.967076397 | 0.868733261 | unchanged |
| hsa_circ_101689 | 247 | 162 | 147 | 163 | 96 | 97 | 185.256478 | 118.4091288 | 0.63916323 | 0.155134812 | unchanged |
| hsa_circ_101692 | 1161 | 1370 | 993 | 1093 | 1187 | 1191 | 1174.675003 | 1157.192356 | 0.985117035 | 0.885142003 | unchanged |
| hsa_circ_101693 | 196 | 342 | 258 | 189 | 246 | 236 | 265.4810731 | 223.5598619 | 0.842093409 | 0.412630687 | unchanged |
| hsa_circ_101694 | 75 | 92 | 394 | 69 | 87 | 101 | 187.0114016 | 85.87429237 | 0.459192817 | 0.385973253 | unchanged |
| hsa_circ_101695 | 3302 | 1880 | 1318 | 3125 | 2053 | 2058 | 2166.604208 | 2412.029613 | 1.113276529 | 0.739985181 | unchanged |
| hsa_circ_101696 | 152 | 87 | 90 | 123 | 117 | 111 | 109.6558605 | 116.75945 | 1.064780755 | 0.75951889 | unchanged |
| hsa_circ_101697 | 177 | 159 | 151 | 177 | 149 | 150 | 162.3503504 | 158.6739852 | 0.97735536 | 0.776594924 | unchanged |
| hsa_circ_101698 | 2263 | 2367 | 1825 | 2562 | 2432 | 2533 | 2151.621809 | 2509.126314 | 1.166155829 | 0.104279976 | unchanged |
| hsa_circ_101699 | 109 | 156 | 89 | 108 | 93 | 100 | 118.0002609 | 100.1891918 | 0.84905907 | 0.424117179 | unchanged |
| hsa_circ_101700 | 178 | 67 | 86 | 124 | 84 | 79 | 110.3699575 | 96.05333857 | 0.870285182 | 0.719598176 | unchanged |
| hsa_circ_101702 | 99 | 121 | 109 | 122 | 132 | 134 | 109.5180521 | 129.4164627 | 1.181690691 | 0.055731696 | unchanged |
| hsa_circ_101703 | 189 | 225 | 144 | 191 | 142 | 150 | 186.1177245 | 160.7676581 | 0.863795528 | 0.415067819 | unchanged |
| hsa_circ_101704 | 2217 | 1095 | 782 | 1556 | 801 | 820 | 1364.720835 | 1058.950797 | 0.775946823 | 0.57495319 | unchanged |
| hsa_circ_101705 | 478 | 492 | 331 | 388 | 214 | 201 | 433.7627075 | 267.6714102 | 0.617091801 | 0.103374679 | unchanged |
| hsa_circ_101706 | 2251 | 922 | 1025 | 1457 | 695 | 691 | 1399.596495 | 947.5090985 | 0.676987333 | 0.414640895 | unchanged |
| hsa_circ_101707 | 8805 | 7180 | 4163 | 10809 | 4333 | 3841 | 6716.269243 | 6327.953591 | 0.942182834 | 0.889548384 | unchanged |
| hsa_circ_101708 | 467 | 455 | 376 | 441 | 434 | 468 | 433.0679375 | 447.8597066 | 1.034155771 | 0.65243613 | unchanged |
| hsa_circ_101710 | 94 | 75 | 125 | 97 | 141 | 129 | 98.08912319 | 122.3881014 | 1.247723472 | 0.287118084 | unchanged |
| hsa_circ_101712 | 490 | 197 | 167 | 478 | 220 | 225 | 284.7623548 | 307.5975561 | 1.08019038 | 0.87264969 | unchanged |
| hsa_circ_101713 | 67 | 55 | 71 | 70 | 78 | 78 | 64.57758713 | 75.52760567 | 1.169563761 | 0.118318754 | unchanged |
| hsa_circ_101714 | 82 | 61 | 107 | 80 | 116 | 117 | 83.46027082 | 104.31567 | 1.249884155 | 0.313195112 | unchanged |
| hsa_circ_101715 | 262 | 142 | 148 | 248 | 178 | 166 | 183.9884171 | 197.2658725 | 1.072164626 | 0.789264253 | unchanged |
| hsa_circ_101717 | 242 | 153 | 175 | 201 | 183 | 182 | 190.0498479 | 188.8822811 | 0.993856523 | 0.96794021 | unchanged |
| hsa_circ_101718 | 1214 | 1365 | 1221 | 1181 | 1362 | 1309 | 1266.586457 | 1283.9938 | 1.013743509 | 0.823242788 | unchanged |
| hsa_circ_101719 | 148 | 179 | 177 | 153 | 164 | 161 | 167.8539321 | 159.4866664 | 0.950151506 | 0.476021485 | unchanged |
| hsa_circ_101720 | 539 | 1519 | 1085 | 561 | 979 | 1036 | 1047.776337 | 858.6707729 | 0.819517241 | 0.586981151 | unchanged |
| hsa_circ_101721 | 2627 | 4510 | 4222 | 2725 | 3887 | 4142 | 3786.362317 | 3584.624075 | 0.946719773 | 0.795986312 | unchanged |
| hsa_circ_101722 | 4238 | 5109 | 4174 | 4073 | 4234 | 4086 | 4507.328158 | 4130.764125 | 0.916455155 | 0.2857291 | unchanged |
| hsa_circ_101724 | 84 | 168 | 99 | 79 | 84 | 85 | 117.1075197 | 82.87039174 | 0.707643642 | 0.257970665 | unchanged |
| hsa_circ_101725 | 4545 | 7461 | 7198 | 4706 | 6575 | 6891 | 6401.21522 | 6057.59927 | 0.9463202 | 0.780747571 | unchanged |
| hsa_circ_101726 | 195 | 90 | 196 | 154 | 101 | 109 | 160.3709018 | 121.2733715 | 0.756205584 | 0.371388428 | unchanged |
| hsa_circ_101727 | 221 | 227 | 241 | 307 | 240 | 259 | 229.7520102 | 268.8294779 | 1.170085423 | 0.131064604 | unchanged |
| hsa_circ_101728 | 808 | 2340 | 1836 | 1022 | 1459 | 1570 | 1661.273761 | 1350.427134 | 0.812886573 | 0.553012683 | unchanged |
| hsa_circ_101730 | 120 | 70 | 104 | 126 | 131 | 131 | 97.9626692 | 129.2560953 | 1.319442358 | 0.100232161 | unchanged |
| hsa_circ_101731 | 75 | 73 | 61 | 71 | 68 | 62 | 69.9305065 | 66.91151977 | 0.956828759 | 0.596182103 | unchanged |
| hsa_circ_101735 | 238 | 339 | 304 | 271 | 377 | 346 | 293.7152074 | 331.660945 | 1.129192281 | 0.428967971 | unchanged |
| hsa_circ_101737 | 72 | 57 | 59 | 82 | 65 | 63 | 62.68624241 | 70.05561215 | 1.117559603 | 0.397335223 | unchanged |
| hsa_circ_101739 | 146 | 102 | 162 | 157 | 169 | 163 | 136.8233547 | 162.6941699 | 1.189081866 | 0.230478766 | unchanged |
| hsa_circ_101740 | 1423 | 1401 | 1019 | 1571 | 831 | 892 | 1280.901676 | 1097.895414 | 0.857127002 | 0.536637955 | unchanged |
| hsa_circ_101741 | 503 | 673 | 485 | 538 | 484 | 528 | 553.695558 | 516.4952255 | 0.932814465 | 0.581516581 | unchanged |
| hsa_circ_101742 | 97 | 158 | 118 | 110 | 105 | 104 | 124.4383507 | 106.217781 | 0.853577538 | 0.36938718 | unchanged |
| hsa_circ_101743 | 123 | 124 | 138 | 130 | 139 | 131 | 128.3175152 | 133.2214399 | 1.038217111 | 0.41975767 | unchanged |
| hsa_circ_101744 | 7078 | 6183 | 3976 | 9066 | 4587 | 4498 | 5745.588586 | 6050.604824 | 1.053087031 | 0.871370103 | unchanged |
| hsa_circ_101745 | 88 | 110 | 98 | 94 | 89 | 88 | 98.72923923 | 90.25073048 | 0.914123629 | 0.278518178 | unchanged |
| hsa_circ_101746 | 226 | 323 | 245 | 258 | 273 | 260 | 264.3940349 | 263.8387458 | 0.997899767 | 0.986103286 | unchanged |
| hsa_circ_101747 | 123 | 126 | 79 | 106 | 84 | 76 | 109.6463695 | 88.74420658 | 0.809367487 | 0.301333006 | unchanged |
| hsa_circ_101748 | 8992 | 8247 | 4298 | 11340 | 5404 | 5862 | 7179.304108 | 7535.436228 | 1.049605382 | 0.889186281 | unchanged |
| hsa_circ_101749 | 419 | 202 | 270 | 438 | 421 | 424 | 296.8981812 | 427.7092555 | 1.440592374 | 0.111534045 | unchanged |
| hsa_circ_101751 | 123 | 59 | 92 | 103 | 118 | 116 | 91.41291029 | 112.5976026 | 1.231747269 | 0.330080512 | unchanged |
| hsa_circ_101752 | 58 | 70 | 65 | 67 | 83 | 88 | 64.23958121 | 79.30805319 | 1.234566784 | 0.099939061 | unchanged |
| hsa_circ_101753 | 2704 | 798 | 620 | 1173 | 474 | 458 | 1374.180237 | 701.4244034 | 0.510431154 | 0.39536951 | unchanged |
| hsa_circ_101755 | 79 | 54 | 78 | 79 | 84 | 84 | 70.25354336 | 82.30944852 | 1.171605653 | 0.230644376 | unchanged |
| hsa_circ_101756 | 1187 | 1472 | 1331 | 1255 | 1567 | 1586 | 1330.123851 | 1469.58758 | 1.104850183 | 0.360778556 | unchanged |
| hsa_circ_101757 | 459 | 520 | 532 | 541 | 402 | 455 | 503.7919147 | 465.7791636 | 0.924546723 | 0.458979457 | unchanged |
| hsa_circ_101758 | 254 | 366 | 315 | 259 | 332 | 337 | 311.5062041 | 309.4288175 | 0.993331155 | 0.961999172 | unchanged |
| hsa_circ_101759 | 468 | 297 | 266 | 336 | 222 | 217 | 343.7035145 | 258.2634164 | 0.751413371 | 0.312114778 | unchanged |
| hsa_circ_101760 | 129 | 175 | 155 | 157 | 171 | 184 | 152.8488982 | 170.7785599 | 1.117303179 | 0.309646908 | unchanged |
| hsa_circ_101761 | 181 | 102 | 94 | 117 | 83 | 83 | 125.5436051 | 94.39379603 | 0.751880559 | 0.359555431 | unchanged |
| hsa_circ_101762 | 985 | 870 | 922 | 970 | 1347 | 1317 | 925.8902644 | 1211.514137 | 1.308485663 | 0.085436674 | unchanged |
| hsa_circ_101763 | 68 | 57 | 90 | 67 | 107 | 109 | 71.44950844 | 94.14862657 | 1.317694532 | 0.248710675 | unchanged |
| hsa_circ_101764 | 71 | 117 | 121 | 72 | 87 | 101 | 103.095141 | 87.05270609 | 0.84439194 | 0.428612486 | unchanged |
| hsa_circ_101765 | 317 | 205 | 230 | 297 | 308 | 314 | 250.8870392 | 306.4346283 | 1.221404777 | 0.18075278 | unchanged |
| hsa_circ_101766 | 1207 | 1436 | 1266 | 1089 | 1237 | 1301 | 1303.146342 | 1208.911919 | 0.927686999 | 0.367834728 | unchanged |
| hsa_circ_101767 | 342 | 439 | 403 | 368 | 341 | 335 | 394.5880917 | 348.0849504 | 0.882147631 | 0.196869226 | unchanged |
| hsa_circ_101768 | 237 | 380 | 337 | 265 | 296 | 298 | 318.0982196 | 286.5805051 | 0.900918293 | 0.510940666 | unchanged |
| hsa_circ_101772 | 62 | 64 | 62 | 66 | 57 | 64 | 62.42031237 | 62.35839204 | 0.99900801 | 0.983173401 | unchanged |
| hsa_circ_101774 | 72 | 125 | 66 | 56 | 59 | 62 | 87.34997105 | 59.1149731 | 0.676760077 | 0.209744079 | unchanged |
| hsa_circ_101775 | 625 | 658 | 512 | 497 | 504 | 545 | 598.3330237 | 515.4172033 | 0.861421956 | 0.14953632 | unchanged |
| hsa_circ_101777 | 7159 | 7035 | 5485 | 8102 | 8365 | 8340 | 6559.357512 | 8268.887784 | 1.260624652 | 0.034955421 | unchanged |
| hsa_circ_101778 | 135 | 202 | 230 | 145 | 219 | 216 | 189.2440486 | 193.4564743 | 1.022259224 | 0.915452175 | unchanged |
| hsa_circ_101779 | 1184 | 1514 | 1040 | 1391 | 1648 | 1764 | 1246.23519 | 1600.965386 | 1.284641454 | 0.11773019 | unchanged |
| hsa_circ_101780 | 246 | 418 | 409 | 304 | 562 | 558 | 357.771268 | 474.9473063 | 1.327516625 | 0.314993394 | unchanged |
| hsa_circ_101781 | 57 | 98 | 56 | 53 | 51 | 53 | 70.27973638 | 52.54809115 | 0.747699036 | 0.271562648 | unchanged |
| hsa_circ_101782 | 232 | 181 | 177 | 239 | 200 | 185 | 196.606876 | 207.9843259 | 1.057869033 | 0.66224106 | unchanged |
| hsa_circ_101784 | 2225 | 3530 | 2759 | 2133 | 2286 | 2307 | 2838.086642 | 2242.012355 | 0.789973189 | 0.194245482 | unchanged |
| hsa_circ_101785 | 464 | 690 | 613 | 410 | 601 | 670 | 588.9392491 | 560.3191131 | 0.951403925 | 0.793282126 | unchanged |
| hsa_circ_101787 | 1030 | 948 | 932 | 1049 | 963 | 987 | 969.964056 | 999.663833 | 1.030619461 | 0.495245731 | unchanged |
| hsa_circ_101788 | 602 | 485 | 392 | 548 | 426 | 445 | 493.0901478 | 472.8255545 | 0.958902863 | 0.791352411 | unchanged |
| hsa_circ_101789 | 144 | 109 | 90 | 142 | 117 | 123 | 114.2153879 | 127.4182602 | 1.115596266 | 0.492792329 | unchanged |
| hsa_circ_101790 | 102 | 83 | 90 | 92 | 103 | 100 | 91.71997552 | 98.16241915 | 1.070240355 | 0.381308049 | unchanged |
| hsa_circ_101794 | 326 | 402 | 338 | 356 | 346 | 372 | 355.1258639 | 357.9414113 | 1.007928309 | 0.914900416 | unchanged |
| hsa_circ_101795 | 173 | 279 | 211 | 197 | 238 | 236 | 221.1022968 | 223.7330112 | 1.011898177 | 0.941550568 | unchanged |
| hsa_circ_101796 | 1570 | 2144 | 2481 | 1723 | 2482 | 2559 | 2064.638597 | 2254.726531 | 1.092068382 | 0.640347111 | unchanged |
| hsa_circ_101798 | 8378 | 13251 | 11845 | 8648 | 11082 | 11505 | 11157.84571 | 10411.50403 | 0.933110594 | 0.683284097 | unchanged |
| hsa_circ_101799 | 65 | 86 | 65 | 57 | 63 | 63 | 71.96316826 | 61.08588657 | 0.848849322 | 0.21248819 | unchanged |
| hsa_circ_101800 | 297 | 526 | 354 | 316 | 284 | 262 | 392.4649434 | 287.4345811 | 0.732382818 | 0.21108481 | unchanged |
| hsa_circ_101801 | 64 | 63 | 63 | 62 | 61 | 63 | 63.34250911 | 62.32288125 | 0.983902945 | 0.191453827 | unchanged |
| hsa_circ_101802 | 385 | 384 | 227 | 346 | 214 | 222 | 332.1016761 | 260.6146638 | 0.784743597 | 0.351670623 | unchanged |
| hsa_circ_101803 | 313 | 162 | 180 | 200 | 107 | 107 | 218.2279788 | 138.0326621 | 0.63251588 | 0.230241206 | unchanged |
| hsa_circ_101808 | 91 | 68 | 73 | 92 | 92 | 82 | 77.19972421 | 88.75986064 | 1.149743235 | 0.218302554 | unchanged |
| hsa_circ_101809 | 89 | 77 | 73 | 80 | 66 | 68 | 79.71099753 | 71.14561913 | 0.892544584 | 0.258076255 | unchanged |
| hsa_circ_101811 | 64 | 98 | 56 | 57 | 58 | 61 | 72.52427037 | 58.73546467 | 0.809873224 | 0.341408649 | unchanged |
| hsa_circ_101812 | 154 | 210 | 160 | 151 | 146 | 146 | 174.7104918 | 147.7715898 | 0.845808333 | 0.204639952 | unchanged |
| hsa_circ_101813 | 139 | 142 | 185 | 126 | 136 | 152 | 155.3209468 | 137.8979447 | 0.887825805 | 0.355989919 | unchanged |
| hsa_circ_101815 | 137 | 119 | 156 | 133 | 170 | 153 | 136.9465426 | 151.8880789 | 1.109104882 | 0.376073726 | unchanged |
| hsa_circ_101816 | 215 | 268 | 238 | 177 | 229 | 249 | 240.5170703 | 218.3595962 | 0.90787567 | 0.447726661 | unchanged |
| hsa_circ_101817 | 99 | 77 | 93 | 95 | 121 | 132 | 89.74363207 | 115.7930887 | 1.290265236 | 0.109519864 | unchanged |
| hsa_circ_101818 | 72 | 81 | 67 | 73 | 70 | 73 | 73.19023869 | 71.83977574 | 0.981548592 | 0.776998839 | unchanged |
| hsa_circ_101819 | 114 | 89 | 92 | 123 | 95 | 102 | 98.11806677 | 106.7408542 | 1.08788175 | 0.499010467 | unchanged |
| hsa_circ_101820 | 2633 | 5981 | 6153 | 2472 | 4202 | 4434 | 4922.368227 | 3702.454637 | 0.752169376 | 0.401934092 | unchanged |
| hsa_circ_101821 | 127 | 79 | 131 | 121 | 130 | 123 | 112.3874267 | 124.8051746 | 1.110490544 | 0.50230345 | unchanged |
| hsa_circ_101825 | 78 | 88 | 62 | 75 | 65 | 66 | 75.90869025 | 68.8975837 | 0.90763763 | 0.436154931 | unchanged |
| hsa_circ_101826 | 267 | 408 | 319 | 280 | 337 | 352 | 331.4312303 | 322.955975 | 0.974428314 | 0.864617018 | unchanged |
| hsa_circ_101828 | 76 | 115 | 141 | 93 | 171 | 167 | 110.3733538 | 143.5296478 | 1.300401255 | 0.355827068 | unchanged |
| hsa_circ_101829 | 81 | 102 | 79 | 77 | 74 | 72 | 87.14813752 | 74.01151761 | 0.849261037 | 0.14846862 | unchanged |
| hsa_circ_101830 | 201 | 239 | 307 | 202 | 265 | 274 | 248.9193382 | 246.7930269 | 0.99145783 | 0.958413581 | unchanged |
| hsa_circ_101831 | 110 | 103 | 133 | 110 | 131 | 134 | 115.5499336 | 125.1896671 | 1.083424829 | 0.462215792 | unchanged |
| hsa_circ_101832 | 390 | 388 | 321 | 343 | 366 | 349 | 366.4956272 | 352.7594237 | 0.962520143 | 0.590905722 | unchanged |
| hsa_circ_101834 | 311 | 341 | 322 | 340 | 306 | 343 | 324.8196844 | 329.8124842 | 1.015370989 | 0.751230089 | unchanged |
| hsa_circ_101835 | 255 | 304 | 326 | 264 | 339 | 346 | 295.237753 | 316.3290746 | 1.07143843 | 0.560857866 | unchanged |
| hsa_circ_101836 | 201 | 246 | 298 | 229 | 327 | 323 | 248.2760996 | 293.102453 | 1.180550417 | 0.351476556 | unchanged |
| hsa_circ_101837 | 129 | 155 | 226 | 149 | 233 | 230 | 169.9594235 | 203.9827713 | 1.200185121 | 0.443031538 | unchanged |
| hsa_circ_101838 | 274 | 260 | 327 | 324 | 457 | 516 | 287.1009089 | 432.564633 | 1.506664101 | 0.073432633 | unchanged |
| hsa_circ_101839 | 256 | 130 | 133 | 149 | 120 | 117 | 173.1395661 | 128.7090536 | 0.743383252 | 0.357951034 | unchanged |
| hsa_circ_101841 | 747 | 303 | 228 | 715 | 304 | 300 | 425.8162484 | 439.7976395 | 1.03283433 | 0.950647285 | unchanged |
| hsa_circ_101842 | 649 | 428 | 358 | 571 | 415 | 414 | 478.1729448 | 466.742095 | 0.976094737 | 0.916141823 | unchanged |
| hsa_circ_101843 | 153 | 98 | 72 | 90 | 69 | 75 | 107.8655834 | 78.35582109 | 0.726420964 | 0.299699778 | unchanged |
| hsa_circ_101844 | 84 | 59 | 64 | 78 | 60 | 64 | 69.14005311 | 67.38035607 | 0.974548804 | 0.863154553 | unchanged |
| hsa_circ_101846 | 878 | 1070 | 735 | 921 | 619 | 762 | 894.1936918 | 767.3095292 | 0.85810215 | 0.385783301 | unchanged |
| hsa_circ_101849 | 159 | 158 | 157 | 156 | 169 | 192 | 157.9509762 | 172.1701278 | 1.090022562 | 0.251637387 | unchanged |
| hsa_circ_101850 | 577 | 984 | 1077 | 1065 | 1368 | 1307 | 879.5872574 | 1246.564269 | 1.417215016 | 0.10994155 | unchanged |
| hsa_circ_101851 | 280 | 218 | 157 | 315 | 190 | 187 | 218.1875737 | 230.5807377 | 1.056800503 | 0.833283691 | unchanged |
| hsa_circ_101852 | 4066 | 736 | 431 | 5165 | 547 | 518 | 1744.425667 | 2076.636004 | 1.190441097 | 0.871951131 | unchanged |
| hsa_circ_101853 | 8429 | 2954 | 2357 | 10405 | 3208 | 3206 | 4580.232872 | 5606.534004 | 1.224071823 | 0.755748486 | unchanged |
| hsa_circ_101854 | 174 | 138 | 205 | 140 | 203 | 218 | 172.2244216 | 186.9742129 | 1.085642856 | 0.657210551 | unchanged |
| hsa_circ_101855 | 78 | 85 | 96 | 78 | 86 | 89 | 86.34808809 | 83.89376592 | 0.971576416 | 0.720467605 | unchanged |
| hsa_circ_101856 | 99 | 147 | 94 | 106 | 91 | 95 | 113.3241429 | 97.13286939 | 0.857124236 | 0.408351622 | unchanged |
| hsa_circ_101857 | 582 | 845 | 693 | 662 | 988 | 891 | 706.6972963 | 846.7713111 | 1.198209354 | 0.317960404 | unchanged |
| hsa_circ_101858 | 149 | 135 | 100 | 158 | 102 | 102 | 128.2307234 | 120.5850915 | 0.940375975 | 0.763387529 | unchanged |
| hsa_circ_101859 | 67 | 79 | 69 | 71 | 72 | 70 | 71.68815266 | 71.19533836 | 0.993125582 | 0.896485526 | unchanged |
| hsa_circ_101860 | 135 | 72 | 99 | 113 | 94 | 89 | 102.0683092 | 98.97474096 | 0.969691198 | 0.882847528 | unchanged |
| hsa_circ_101861 | 5973 | 1699 | 1197 | 6149 | 1719 | 1610 | 2956.302825 | 3159.1518 | 1.068615764 | 0.9286677 | unchanged |
| hsa_circ_101862 | 246 | 273 | 632 | 242 | 339 | 350 | 383.7820105 | 310.2373037 | 0.808368541 | 0.599416997 | unchanged |
| hsa_circ_101863 | 363 | 604 | 565 | 391 | 603 | 623 | 510.7179196 | 538.6748805 | 1.054740513 | 0.803776785 | unchanged |
| hsa_circ_101864 | 167 | 207 | 259 | 214 | 274 | 253 | 211.1246091 | 246.6646336 | 1.168336721 | 0.32720166 | unchanged |
| hsa_circ_101865 | 449 | 444 | 519 | 526 | 554 | 529 | 470.7099978 | 536.3200535 | 1.139385303 | 0.064073218 | unchanged |
| hsa_circ_101866 | 119 | 99 | 169 | 126 | 181 | 183 | 129.1389112 | 163.1275608 | 1.263194489 | 0.288049267 | unchanged |
| hsa_circ_101867 | 2669 | 3139 | 3010 | 2925 | 3241 | 3178 | 2939.23748 | 3114.868666 | 1.059753996 | 0.360517195 | unchanged |
| hsa_circ_101868 | 383 | 170 | 219 | 286 | 244 | 246 | 257.0924622 | 258.3503522 | 1.004892753 | 0.985651203 | unchanged |
| hsa_circ_101869 | 99 | 71 | 93 | 98 | 115 | 118 | 87.65673411 | 110.3415123 | 1.258791049 | 0.10244662 | unchanged |
| hsa_circ_101870 | 134 | 116 | 120 | 136 | 112 | 107 | 123.5590728 | 118.3564161 | 0.957893366 | 0.645353261 | unchanged |
| hsa_circ_101871 | 82 | 102 | 86 | 85 | 88 | 89 | 90.02408112 | 87.47562077 | 0.971691348 | 0.694545237 | unchanged |
| hsa_circ_101873 | 578 | 793 | 480 | 907 | 604 | 582 | 616.9551245 | 697.7582419 | 1.130970818 | 0.594477966 | unchanged |
| hsa_circ_101874 | 89 | 103 | 102 | 97 | 100 | 103 | 98.29477886 | 100.0705654 | 1.01806593 | 0.730851134 | unchanged |
| hsa_circ_101875 | 558 | 550 | 548 | 632 | 485 | 475 | 552.2032233 | 530.9703348 | 0.961548778 | 0.697942379 | unchanged |
| hsa_circ_101876 | 86 | 72 | 65 | 79 | 63 | 71 | 74.29149239 | 70.88804287 | 0.954187897 | 0.687565235 | unchanged |
| hsa_circ_101877 | 1782 | 761 | 361 | 2542 | 701 | 559 | 968.088451 | 1267.240502 | 1.309013139 | 0.716129125 | unchanged |
| hsa_circ_101878 | 61 | 103 | 63 | 64 | 67 | 69 | 75.59438754 | 66.52957485 | 0.880086168 | 0.542592512 | unchanged |
| hsa_circ_101880 | 385 | 164 | 187 | 323 | 215 | 214 | 245.4466911 | 250.7451385 | 1.021586958 | 0.949729946 | unchanged |
| hsa_circ_101881 | 494 | 182 | 283 | 313 | 246 | 236 | 319.9587312 | 264.8960905 | 0.827907054 | 0.593633968 | unchanged |
| hsa_circ_101882 | 967 | 604 | 750 | 756 | 774 | 731 | 773.6615842 | 753.951055 | 0.974523061 | 0.861981359 | unchanged |
| hsa_circ_101884 | 71 | 83 | 70 | 75 | 73 | 69 | 74.75604282 | 72.02674531 | 0.96349061 | 0.583878957 | unchanged |
| hsa_circ_101885 | 345 | 231 | 243 | 292 | 262 | 277 | 273.1809084 | 277.0594719 | 1.014197784 | 0.921617116 | unchanged |
| hsa_circ_101886 | 148 | 120 | 112 | 130 | 119 | 120 | 126.805619 | 122.9309446 | 0.969443986 | 0.755081415 | unchanged |
| hsa_circ_101887 | 2467 | 3173 | 2223 | 2290 | 2573 | 2611 | 2620.777255 | 2491.265796 | 0.950582806 | 0.690444989 | unchanged |
| hsa_circ_101888 | 565 | 639 | 869 | 604 | 774 | 767 | 691.3188044 | 715.0962705 | 1.034394358 | 0.835163683 | unchanged |
| hsa_circ_101889 | 167 | 100 | 137 | 140 | 120 | 118 | 134.7496819 | 126.2469987 | 0.936900161 | 0.700649163 | unchanged |
| hsa_circ_101890 | 77 | 83 | 60 | 76 | 67 | 76 | 73.56515989 | 72.86906504 | 0.99053771 | 0.930517707 | unchanged |
| hsa_circ_101891 | 501 | 929 | 781 | 613 | 861 | 868 | 736.9807869 | 780.5814257 | 1.059161161 | 0.787020285 | unchanged |
| hsa_circ_101893 | 323 | 275 | 239 | 343 | 331 | 283 | 278.8827846 | 318.8791908 | 1.143416548 | 0.260336853 | unchanged |
| hsa_circ_101894 | 88 | 95 | 78 | 84 | 80 | 85 | 87.28552501 | 82.95762307 | 0.950416728 | 0.461181919 | unchanged |
| hsa_circ_101895 | 5889 | 6906 | 8402 | 6371 | 7874 | 7792 | 7065.866452 | 7345.519791 | 1.039578067 | 0.765996757 | unchanged |
| hsa_circ_101896 | 139 | 102 | 96 | 154 | 131 | 117 | 112.3472472 | 133.9576294 | 1.192353464 | 0.283943428 | unchanged |
| hsa_circ_101897 | 121 | 142 | 130 | 121 | 127 | 130 | 130.9801331 | 126.1186659 | 0.962883935 | 0.498956901 | unchanged |
| hsa_circ_101898 | 89 | 124 | 90 | 96 | 77 | 83 | 101.1308733 | 85.42876227 | 0.844734743 | 0.278952198 | unchanged |
| hsa_circ_101899 | 73 | 147 | 67 | 63 | 65 | 66 | 95.5682208 | 64.55844807 | 0.67552213 | 0.292727194 | unchanged |
| hsa_circ_101900 | 320 | 448 | 530 | 351 | 426 | 426 | 432.3950622 | 400.826196 | 0.926990688 | 0.657612693 | unchanged |
| hsa_circ_101901 | 1618 | 1335 | 1421 | 1347 | 1224 | 1221 | 1457.940597 | 1264.131723 | 0.867066687 | 0.107405226 | unchanged |
| hsa_circ_101902 | 360 | 355 | 337 | 393 | 293 | 308 | 350.6969598 | 331.165637 | 0.944307123 | 0.575596426 | unchanged |
| hsa_circ_101904 | 1606 | 3777 | 5242 | 1639 | 3696 | 4137 | 3541.56314 | 3157.114298 | 0.891446566 | 0.783267934 | unchanged |
| hsa_circ_101905 | 1462 | 1085 | 998 | 957 | 929 | 925 | 1181.546636 | 936.804018 | 0.792862499 | 0.161576678 | unchanged |
| hsa_circ_101906 | 6146 | 13135 | 17587 | 7581 | 11631 | 11862 | 12289.31439 | 10357.9444 | 0.842841519 | 0.620848525 | unchanged |
| hsa_circ_101907 | 292 | 466 | 427 | 275 | 409 | 424 | 395.0004775 | 369.4651864 | 0.935353772 | 0.73718397 | unchanged |
| hsa_circ_101908 | 143 | 185 | 118 | 109 | 90 | 100 | 148.5555118 | 99.5405061 | 0.67005596 | 0.074591651 | unchanged |
| hsa_circ_101909 | 67 | 96 | 61 | 63 | 57 | 55 | 74.89037395 | 58.29107334 | 0.778352013 | 0.209004017 | unchanged |
| hsa_circ_101910 | 589 | 851 | 698 | 648 | 821 | 839 | 712.3762777 | 769.5665532 | 1.080280994 | 0.588062267 | unchanged |
| hsa_circ_101911 | 844 | 791 | 743 | 814 | 865 | 907 | 792.9334651 | 862.2218462 | 1.087382339 | 0.156982791 | unchanged |
| hsa_circ_101912 | 169 | 251 | 187 | 200 | 209 | 208 | 202.4362048 | 205.8646344 | 1.016935852 | 0.897268738 | unchanged |
| hsa_circ_101913 | 77 | 70 | 74 | 75 | 88 | 85 | 73.47403579 | 82.75857241 | 1.126364865 | 0.103092665 | unchanged |
| hsa_circ_101915 | 182 | 302 | 280 | 222 | 348 | 344 | 254.417752 | 304.5176675 | 1.196919889 | 0.416539146 | unchanged |
| hsa_circ_101916 | 78 | 177 | 96 | 72 | 89 | 84 | 116.9009252 | 81.60377748 | 0.698059296 | 0.318715536 | unchanged |
| hsa_circ_101917 | 435 | 475 | 354 | 349 | 334 | 333 | 421.2908606 | 338.4967507 | 0.803475182 | 0.083472893 | unchanged |
| hsa_circ_101918 | 97 | 185 | 111 | 100 | 122 | 128 | 130.8871674 | 116.7976133 | 0.892353435 | 0.648897485 | unchanged |
| hsa_circ_101919 | 383 | 433 | 391 | 440 | 421 | 433 | 402.342111 | 431.5003375 | 1.072471227 | 0.154296084 | unchanged |
| hsa_circ_101920 | 212 | 282 | 305 | 247 | 324 | 334 | 266.4420678 | 301.8761077 | 1.132989659 | 0.418242771 | unchanged |
| hsa_circ_101921 | 217 | 263 | 269 | 246 | 277 | 264 | 249.6923073 | 262.2036186 | 1.050106915 | 0.538169811 | unchanged |
| hsa_circ_101922 | 312 | 227 | 600 | 248 | 134 | 135 | 379.6319264 | 172.4217525 | 0.454181381 | 0.156228426 | unchanged |
| hsa_circ_101923 | 157 | 195 | 174 | 134 | 151 | 166 | 175.3932923 | 150.3555224 | 0.857247848 | 0.158085726 | unchanged |
| hsa_circ_101924 | 2453 | 5146 | 7004 | 2392 | 4067 | 4888 | 4867.72937 | 3782.287285 | 0.777012648 | 0.512416773 | unchanged |
| hsa_circ_101925 | 123 | 128 | 180 | 113 | 118 | 121 | 144.0142134 | 117.2679966 | 0.814280715 | 0.220349334 | unchanged |
| hsa_circ_101926 | 112 | 110 | 135 | 108 | 125 | 123 | 119.0737368 | 118.7285111 | 0.997100739 | 0.973132879 | unchanged |
| hsa_circ_101928 | 172 | 211 | 213 | 184 | 229 | 255 | 198.9680411 | 222.3323155 | 1.117427272 | 0.396237868 | unchanged |
| hsa_circ_101929 | 65 | 49 | 67 | 57 | 67 | 66 | 60.45903918 | 63.29312956 | 1.046876206 | 0.689761711 | unchanged |
| hsa_circ_101930 | 184 | 165 | 142 | 475 | 160 | 171 | 163.8056552 | 268.7912257 | 1.640915422 | 0.370117579 | unchanged |
| hsa_circ_101932 | 174 | 128 | 164 | 169 | 199 | 195 | 155.2280032 | 187.5265179 | 1.208071444 | 0.125694669 | unchanged |
| hsa_circ_101933 | 258 | 187 | 141 | 251 | 137 | 131 | 195.3705855 | 172.8211654 | 0.884581295 | 0.686041005 | unchanged |
| hsa_circ_101934 | 167 | 71 | 209 | 178 | 229 | 231 | 149.0008799 | 212.8758931 | 1.428688832 | 0.221904734 | unchanged |
| hsa_circ_101935 | 141 | 223 | 178 | 147 | 186 | 180 | 180.6847932 | 170.9648318 | 0.946204873 | 0.734402748 | unchanged |
| hsa_circ_101936 | 93 | 164 | 270 | 89 | 86 | 86 | 175.7236151 | 86.78046997 | 0.493846373 | 0.157512585 | unchanged |
| hsa_circ_101938 | 635 | 500 | 785 | 543 | 821 | 770 | 640.1400913 | 711.4163005 | 1.111344704 | 0.5803507 | unchanged |
| hsa_circ_101939 | 101 | 137 | 108 | 105 | 95 | 108 | 115.1907779 | 102.8043637 | 0.892470435 | 0.358635679 | unchanged |
| hsa_circ_101941 | 87 | 66 | 90 | 97 | 98 | 99 | 81.12429316 | 97.90072044 | 1.206799056 | 0.087412906 | unchanged |
| hsa_circ_101942 | 136 | 187 | 129 | 162 | 123 | 131 | 150.5682196 | 138.7938833 | 0.921800654 | 0.619945757 | unchanged |
| hsa_circ_101943 | 1925 | 818 | 738 | 1060 | 495 | 520 | 1160.277657 | 691.6293567 | 0.596089524 | 0.332127427 | unchanged |
| hsa_circ_101944 | 170 | 206 | 232 | 185 | 303 | 275 | 202.6944806 | 254.6210196 | 1.256181317 | 0.261475709 | unchanged |
| hsa_circ_101945 | 2944 | 1202 | 980 | 3673 | 1461 | 1484 | 1708.933889 | 2206.135215 | 1.2909424 | 0.632262351 | unchanged |
| hsa_circ_101947 | 1172 | 1493 | 1263 | 1223 | 1383 | 1397 | 1309.454129 | 1334.338796 | 1.019003848 | 0.832861738 | unchanged |
| hsa_circ_101948 | 36298 | 35811 | 39820 | 32802 | 40309 | 37985 | 37309.6431 | 37032.04629 | 0.99255965 | 0.91864962 | unchanged |
| hsa_circ_101949 | 80 | 66 | 66 | 61 | 65 | 64 | 70.65733049 | 63.29766987 | 0.895840098 | 0.194696917 | unchanged |
| hsa_circ_101950 | 49 | 89 | 50 | 48 | 53 | 55 | 62.76172023 | 51.68025754 | 0.823435963 | 0.44623135 | unchanged |
| hsa_circ_101951 | 276 | 367 | 307 | 284 | 337 | 329 | 316.4915399 | 316.5697076 | 1.000246982 | 0.998136861 | unchanged |
| hsa_circ_101952 | 3292 | 2784 | 2389 | 3050 | 2927 | 2994 | 2821.596389 | 2990.127764 | 1.059729087 | 0.557818508 | unchanged |
| hsa_circ_101954 | 149 | 131 | 106 | 136 | 111 | 112 | 128.9243398 | 119.9003338 | 0.930005413 | 0.574959385 | unchanged |
| hsa_circ_101955 | 100 | 173 | 108 | 110 | 125 | 113 | 127.1110949 | 116.0202354 | 0.912746724 | 0.66255222 | unchanged |
| hsa_circ_101956 | 82 | 157 | 75 | 68 | 69 | 67 | 104.5711629 | 68.39483712 | 0.65405065 | 0.241197151 | unchanged |
| hsa_circ_101957 | 696 | 536 | 526 | 505 | 505 | 489 | 585.9341106 | 499.9631199 | 0.853275327 | 0.194094317 | unchanged |
| hsa_circ_101958 | 3024 | 1336 | 1427 | 1800 | 1057 | 1195 | 1928.989763 | 1350.418625 | 0.700065211 | 0.384886222 | unchanged |
| hsa_circ_101959 | 98 | 80 | 83 | 87 | 94 | 95 | 87.43434926 | 92.05294868 | 1.052823627 | 0.493775289 | unchanged |
| hsa_circ_101960 | 127 | 122 | 131 | 124 | 134 | 156 | 126.7001544 | 137.5536184 | 1.085662595 | 0.328868068 | unchanged |
| hsa_circ_101961 | 84 | 71 | 83 | 87 | 88 | 87 | 79.18753033 | 87.56078011 | 1.105739499 | 0.097607152 | unchanged |
| hsa_circ_101963 | 355 | 517 | 723 | 312 | 560 | 527 | 531.6584652 | 466.4961053 | 0.877435677 | 0.647091258 | unchanged |
| hsa_circ_101964 | 318 | 354 | 725 | 257 | 502 | 457 | 465.8865135 | 405.5020498 | 0.870388041 | 0.708776066 | unchanged |
| hsa_circ_101965 | 652 | 1644 | 845 | 980 | 1206 | 1186 | 1046.917456 | 1123.928956 | 1.073560242 | 0.817347647 | unchanged |
| hsa_circ_101966 | 126 | 146 | 115 | 126 | 108 | 107 | 128.8756524 | 113.6279269 | 0.881686531 | 0.237928087 | unchanged |
| hsa_circ_101967 | 21474 | 19372 | 10400 | 29729 | 18694 | 19633 | 17081.98186 | 22685.2067 | 1.328019599 | 0.316572619 | unchanged |
| hsa_circ_101971 | 4091 | 5307 | 4407 | 4256 | 4895 | 4851 | 4601.750964 | 4667.471995 | 1.014281744 | 0.882818771 | unchanged |
| hsa_circ_101972 | 108 | 175 | 115 | 102 | 93 | 94 | 132.853494 | 96.22637345 | 0.724304424 | 0.159845327 | unchanged |
| hsa_circ_101974 | 120 | 114 | 138 | 113 | 133 | 132 | 124.2145744 | 126.0838193 | 1.015048515 | 0.853778258 | unchanged |
| hsa_circ_101975 | 4442 | 5878 | 6761 | 4675 | 6115 | 6331 | 5693.648886 | 5707.148337 | 1.002370967 | 0.988125756 | unchanged |
| hsa_circ_101976 | 9872 | 12233 | 12870 | 8604 | 12233 | 12093 | 11658.39095 | 10976.65291 | 0.941523831 | 0.672405201 | unchanged |
| hsa_circ_101978 | 213 | 77 | 88 | 229 | 137 | 106 | 125.7508515 | 157.2960718 | 1.250854924 | 0.61126481 | unchanged |
| hsa_circ_101979 | 103 | 142 | 130 | 122 | 154 | 156 | 125.1950428 | 144.3623918 | 1.153099904 | 0.294625179 | unchanged |
| hsa_circ_101980 | 83 | 68 | 68 | 85 | 71 | 68 | 72.81404279 | 74.79054642 | 1.027144539 | 0.801580557 | unchanged |
| hsa_circ_101981 | 101 | 89 | 127 | 93 | 153 | 152 | 105.569038 | 132.5934479 | 1.255988029 | 0.29790833 | unchanged |
| hsa_circ_101983 | 71 | 77 | 106 | 67 | 115 | 115 | 85.05565116 | 98.7131723 | 1.16057159 | 0.518531231 | unchanged |
| hsa_circ_101987 | 150 | 90 | 102 | 148 | 121 | 131 | 114.1192412 | 133.2460096 | 1.167603361 | 0.391973193 | unchanged |
| hsa_circ_101988 | 311 | 397 | 334 | 340 | 369 | 389 | 347.0158587 | 365.911288 | 1.054451198 | 0.554441381 | unchanged |
| hsa_circ_101989 | 114 | 136 | 165 | 153 | 162 | 161 | 138.6065483 | 158.7321129 | 1.145199234 | 0.250967494 | unchanged |
| hsa_circ_101990 | 90 | 71 | 95 | 85 | 88 | 89 | 85.4820359 | 87.29821002 | 1.021246266 | 0.819753583 | unchanged |
| hsa_circ_101991 | 64 | 82 | 66 | 68 | 73 | 69 | 70.97749436 | 69.83166617 | 0.983856458 | 0.85725833 | unchanged |
| hsa_circ_101992 | 208 | 224 | 210 | 219 | 259 | 282 | 213.9962017 | 253.2818522 | 1.183581065 | 0.109920018 | unchanged |
| hsa_circ_101994 | 104 | 85 | 89 | 99 | 98 | 101 | 92.68419364 | 99.28405833 | 1.071208093 | 0.307921248 | unchanged |
| hsa_circ_101995 | 543 | 478 | 753 | 548 | 628 | 550 | 591.272089 | 575.4888777 | 0.973306348 | 0.865012332 | unchanged |
| hsa_circ_101997 | 76 | 129 | 85 | 98 | 97 | 88 | 96.89310939 | 94.39359484 | 0.974203382 | 0.887046815 | unchanged |
| hsa_circ_101998 | 653 | 957 | 737 | 693 | 708 | 719 | 782.5674388 | 706.7928614 | 0.903171824 | 0.451832261 | unchanged |
| hsa_circ_102001 | 60 | 86 | 61 | 61 | 60 | 59 | 68.9981474 | 59.78308798 | 0.866444828 | 0.340961703 | unchanged |
| hsa_circ_102002 | 4115 | 3941 | 3022 | 4357 | 3760 | 3782 | 3692.727826 | 3966.220875 | 1.074062607 | 0.523067568 | unchanged |
| hsa_circ_102003 | 733 | 1281 | 1059 | 983 | 1226 | 1352 | 1024.509078 | 1186.855797 | 1.158462938 | 0.447107323 | unchanged |
| hsa_circ_102004 | 126 | 182 | 98 | 139 | 98 | 96 | 135.106921 | 110.7723563 | 0.819886617 | 0.439565473 | unchanged |
| hsa_circ_102005 | 1117 | 856 | 844 | 927 | 899 | 614 | 939.0455611 | 813.399587 | 0.866198213 | 0.400557381 | unchanged |
| hsa_circ_102007 | 182 | 117 | 149 | 163 | 169 | 172 | 149.6425866 | 168.1021915 | 1.123357965 | 0.386791516 | unchanged |
| hsa_circ_102009 | 1014 | 859 | 710 | 906 | 891 | 879 | 860.8067218 | 891.7490184 | 1.035945696 | 0.743149957 | unchanged |
| hsa_circ_102011 | 680 | 887 | 1079 | 770 | 1638 | 1517 | 882.1712913 | 1308.21208 | 1.482945651 | 0.222036099 | unchanged |
| hsa_circ_102012 | 178 | 321 | 381 | 183 | 235 | 233 | 293.1981073 | 217.092284 | 0.740428668 | 0.292299556 | unchanged |
| hsa_circ_102014 | 407 | 402 | 306 | 344 | 310 | 325 | 371.7021076 | 326.5152214 | 0.878432526 | 0.259231393 | unchanged |
| hsa_circ_102015 | 128 | 126 | 136 | 134 | 121 | 117 | 129.6568011 | 123.8687661 | 0.955358802 | 0.385847484 | unchanged |
| hsa_circ_102016 | 91 | 85 | 304 | 93 | 61 | 59 | 160.133019 | 70.95162582 | 0.443079299 | 0.288671238 | unchanged |
| hsa_circ_102017 | 87 | 61 | 88 | 81 | 87 | 88 | 78.77444975 | 85.01721892 | 1.079248655 | 0.52590693 | unchanged |
| hsa_circ_102018 | 310 | 421 | 296 | 441 | 406 | 397 | 342.0626347 | 414.9284313 | 1.213018872 | 0.156519336 | unchanged |
| hsa_circ_102019 | 69 | 64 | 73 | 76 | 67 | 70 | 68.50855644 | 70.91157054 | 1.035076116 | 0.560375554 | unchanged |
| hsa_circ_102021 | 346 | 208 | 144 | 286 | 142 | 156 | 232.6123477 | 194.6561876 | 0.836826547 | 0.64056205 | unchanged |
| hsa_circ_102023 | 245 | 423 | 516 | 265 | 401 | 325 | 394.7482955 | 330.3213189 | 0.836789728 | 0.508181041 | unchanged |
| hsa_circ_102024 | 88 | 76 | 93 | 94 | 97 | 100 | 85.3888644 | 96.95832877 | 1.135491489 | 0.094555364 | unchanged |
| hsa_circ_102025 | 92 | 91 | 69 | 92 | 68 | 70 | 83.97123662 | 76.82608593 | 0.914909546 | 0.540628415 | unchanged |
| hsa_circ_102027 | 230 | 403 | 478 | 241 | 364 | 372 | 370.3355563 | 325.5824196 | 0.879155172 | 0.625241618 | unchanged |
| hsa_circ_102028 | 180 | 219 | 340 | 179 | 299 | 294 | 246.4409994 | 257.1326594 | 1.043384259 | 0.871451667 | unchanged |
| hsa_circ_102029 | 88 | 86 | 87 | 90 | 92 | 86 | 87.05810892 | 89.42675855 | 1.027207685 | 0.248137881 | unchanged |
| hsa_circ_102030 | 149 | 164 | 188 | 158 | 192 | 215 | 167.007525 | 188.3137362 | 1.127576354 | 0.347583978 | unchanged |
| hsa_circ_102031 | 274 | 107 | 79 | 272 | 88 | 84 | 153.1837594 | 147.8439654 | 0.965141253 | 0.953935662 | unchanged |
| hsa_circ_102032 | 216 | 278 | 382 | 210 | 370 | 368 | 291.7760912 | 315.8687907 | 1.082572562 | 0.754188161 | unchanged |
| hsa_circ_102033 | 1327 | 2127 | 1983 | 1544 | 2313 | 2112 | 1812.080028 | 1989.533538 | 1.097928075 | 0.626380744 | unchanged |
| hsa_circ_102034 | 2327 | 3697 | 2976 | 4012 | 3299 | 3547 | 2999.957733 | 3619.536617 | 1.206529205 | 0.238323942 | unchanged |
| hsa_circ_102036 | 73 | 58 | 58 | 64 | 72 | 67 | 63.31383431 | 67.59455599 | 1.067611158 | 0.473331645 | unchanged |
| hsa_circ_102037 | 73 | 64 | 80 | 66 | 80 | 82 | 72.24220756 | 75.8864119 | 1.050444255 | 0.623910429 | unchanged |
| hsa_circ_102039 | 186 | 209 | 175 | 236 | 181 | 206 | 189.9704336 | 207.6842596 | 1.093245173 | 0.399225411 | unchanged |
| hsa_circ_102040 | 122 | 139 | 159 | 133 | 168 | 172 | 140.0225183 | 157.7796024 | 1.126815917 | 0.336542364 | unchanged |
| hsa_circ_102041 | 505 | 217 | 261 | 408 | 142 | 148 | 327.7805057 | 232.662333 | 0.70981138 | 0.490463423 | unchanged |
| hsa_circ_102043 | 279 | 404 | 388 | 289 | 380 | 362 | 357.1419013 | 343.5691036 | 0.961996065 | 0.792248537 | unchanged |
| hsa_circ_102045 | 958 | 596 | 639 | 762 | 425 | 433 | 731.0033891 | 540.0239897 | 0.738743483 | 0.296730406 | unchanged |
| hsa_circ_102046 | 561 | 385 | 527 | 497 | 286 | 288 | 491.0614454 | 357.0167398 | 0.727030686 | 0.204023418 | unchanged |
| hsa_circ_102050 | 450 | 472 | 517 | 484 | 846 | 855 | 479.4517288 | 728.5765179 | 1.519603485 | 0.114482486 | unchanged |
| hsa_circ_102051 | 4841 | 1413 | 1266 | 940 | 978 | 963 | 2506.837081 | 960.5065896 | 0.383154772 | 0.256089349 | unchanged |
| hsa_circ_102052 | 81 | 79 | 94 | 80 | 90 | 94 | 84.63093409 | 87.88384601 | 1.038436441 | 0.627266642 | unchanged |
| hsa_circ_102053 | 146 | 125 | 175 | 147 | 173 | 182 | 148.4991026 | 167.2437363 | 1.126227252 | 0.353698379 | unchanged |
| hsa_circ_102054 | 189 | 183 | 311 | 184 | 182 | 197 | 227.5116182 | 187.8465452 | 0.825656934 | 0.398614977 | unchanged |
| hsa_circ_102055 | 133 | 102 | 111 | 123 | 125 | 136 | 115.4099985 | 128.1658757 | 1.110526621 | 0.266692255 | unchanged |
| hsa_circ_102058 | 74 | 89 | 69 | 72 | 70 | 74 | 77.50951131 | 71.97655483 | 0.928615774 | 0.410497183 | unchanged |
| hsa_circ_102059 | 103 | 95 | 121 | 120 | 143 | 136 | 106.4944693 | 133.0179343 | 1.249059554 | 0.061042919 | unchanged |
| hsa_circ_102060 | 73 | 55 | 65 | 62 | 65 | 67 | 64.18928924 | 64.85579595 | 1.010383457 | 0.906291448 | unchanged |
| hsa_circ_102061 | 23735 | 16309 | 7404 | 34878 | 16386 | 16852 | 15815.87053 | 22705.26016 | 1.435599774 | 0.421725585 | unchanged |
| hsa_circ_102062 | 572 | 443 | 367 | 587 | 362 | 392 | 460.8728369 | 447.1316319 | 0.970184389 | 0.889269737 | unchanged |
| hsa_circ_102063 | 461 | 440 | 418 | 427 | 624 | 475 | 439.4808653 | 508.8337249 | 1.157806323 | 0.317684547 | unchanged |
| hsa_circ_102065 | 392 | 420 | 300 | 419 | 406 | 456 | 370.8678476 | 426.9372509 | 1.15118432 | 0.225054458 | unchanged |
| hsa_circ_102066 | 200 | 227 | 211 | 200 | 207 | 219 | 212.4348286 | 208.6184472 | 0.982035049 | 0.71584841 | unchanged |
| hsa_circ_102067 | 87 | 105 | 101 | 84 | 82 | 83 | 97.74341996 | 83.27444993 | 0.851969882 | 0.056176243 | unchanged |
| hsa_circ_102068 | 86 | 90 | 120 | 77 | 110 | 108 | 98.36707653 | 98.1984782 | 0.998286029 | 0.991580276 | unchanged |
| hsa_circ_102069 | 374 | 374 | 295 | 411 | 426 | 407 | 347.7408957 | 414.5728662 | 1.192188987 | 0.067436983 | unchanged |
| hsa_circ_102070 | 109 | 143 | 104 | 132 | 140 | 154 | 118.3655654 | 141.8781631 | 1.198643901 | 0.163575692 | unchanged |
| hsa_circ_102071 | 270 | 274 | 236 | 242 | 128 | 145 | 259.9757688 | 171.343757 | 0.659075874 | 0.077437461 | unchanged |
| hsa_circ_102072 | 144 | 78 | 90 | 113 | 80 | 82 | 103.8836757 | 91.54926633 | 0.881267107 | 0.622271204 | unchanged |
| hsa_circ_102073 | 2550 | 3068 | 2585 | 2476 | 2881 | 2960 | 2734.650981 | 2772.540762 | 1.013855436 | 0.874175182 | unchanged |
| hsa_circ_102074 | 83 | 101 | 82 | 94 | 100 | 86 | 88.57539791 | 93.39241016 | 1.054383185 | 0.548466311 | unchanged |
| hsa_circ_102075 | 85 | 113 | 170 | 86 | 86 | 91 | 122.7354341 | 87.57666441 | 0.713540185 | 0.234724395 | unchanged |
| hsa_circ_102076 | 107 | 106 | 140 | 115 | 156 | 155 | 117.6097385 | 141.8142088 | 1.205803283 | 0.240977957 | unchanged |
| hsa_circ_102077 | 194 | 184 | 258 | 229 | 258 | 258 | 211.8699338 | 248.400441 | 1.172419496 | 0.218721104 | unchanged |
| hsa_circ_102078 | 90 | 56 | 88 | 86 | 104 | 95 | 78.0384837 | 94.99575919 | 1.217293759 | 0.24266877 | unchanged |
| hsa_circ_102079 | 281 | 377 | 311 | 328 | 371 | 405 | 323.0890017 | 367.8451768 | 1.138525839 | 0.284367434 | unchanged |
| hsa_circ_102080 | 324 | 381 | 318 | 317 | 293 | 292 | 340.6767165 | 300.5631082 | 0.882253156 | 0.137231911 | unchanged |
| hsa_circ_102081 | 145 | 95 | 111 | 116 | 91 | 96 | 116.8150476 | 100.932912 | 0.864040328 | 0.396424721 | unchanged |
| hsa_circ_102082 | 1535 | 659 | 583 | 921 | 485 | 473 | 925.8096068 | 626.4373493 | 0.67663734 | 0.42742109 | unchanged |
| hsa_circ_102083 | 434 | 394 | 415 | 560 | 444 | 411 | 414.3926973 | 471.6622479 | 1.138201158 | 0.28751321 | unchanged |
| hsa_circ_102084 | 68 | 99 | 61 | 70 | 59 | 64 | 75.94566006 | 64.42834029 | 0.848347888 | 0.390127202 | unchanged |
| hsa_circ_102085 | 113 | 137 | 76 | 117 | 99 | 103 | 108.8677438 | 106.3623596 | 0.976986901 | 0.899478253 | unchanged |
| hsa_circ_102086 | 283 | 353 | 327 | 330 | 400 | 382 | 320.9987 | 370.5083298 | 1.154236232 | 0.166041124 | unchanged |
| hsa_circ_102087 | 220 | 229 | 271 | 219 | 269 | 273 | 239.8969094 | 253.6679363 | 1.057403936 | 0.586458555 | unchanged |
| hsa_circ_102088 | 138 | 272 | 154 | 149 | 175 | 175 | 187.8666946 | 166.5564652 | 0.886567284 | 0.648679305 | unchanged |
| hsa_circ_102091 | 1696 | 2233 | 2225 | 1578 | 2574 | 2574 | 2051.260209 | 2241.884185 | 1.092930178 | 0.639205206 | unchanged |
| hsa_circ_102092 | 100 | 114 | 134 | 115 | 138 | 135 | 115.8893064 | 129.3805002 | 1.116414484 | 0.329589845 | unchanged |
| hsa_circ_102093 | 481 | 207 | 245 | 570 | 361 | 348 | 311.2525087 | 426.2540039 | 1.369479737 | 0.361774835 | unchanged |
| hsa_circ_102094 | 147 | 142 | 204 | 139 | 102 | 114 | 164.3770036 | 118.5297843 | 0.721084956 | 0.113267942 | unchanged |
| hsa_circ_102095 | 138 | 107 | 62 | 69 | 50 | 55 | 102.0684007 | 58.1941465 | 0.570148509 | 0.126200396 | unchanged |
| hsa_circ_102096 | 1207 | 1545 | 747 | 1098 | 1167 | 941 | 1166.568644 | 1068.468633 | 0.915907211 | 0.704487528 | unchanged |
| hsa_circ_102097 | 145 | 123 | 107 | 129 | 115 | 108 | 125.0426913 | 117.3776001 | 0.938700206 | 0.574659518 | unchanged |
| hsa_circ_102098 | 125 | 114 | 125 | 125 | 126 | 129 | 120.9896027 | 126.8219242 | 1.048205146 | 0.208773955 | unchanged |
| hsa_circ_102102 | 77 | 138 | 70 | 91 | 67 | 67 | 94.77921616 | 74.53472817 | 0.78640372 | 0.430323663 | unchanged |
| hsa_circ_102103 | 96 | 78 | 78 | 118 | 82 | 85 | 84.04595503 | 94.88883058 | 1.129011272 | 0.456863606 | unchanged |
| hsa_circ_102105 | 172 | 143 | 146 | 149 | 164 | 162 | 153.8548838 | 158.5704921 | 1.030649714 | 0.672275614 | unchanged |
| hsa_circ_102109 | 76 | 86 | 84 | 83 | 76 | 87 | 81.87492492 | 81.92260741 | 1.000582382 | 0.991874341 | unchanged |
| hsa_circ_102113 | 7095 | 2268 | 2590 | 7645 | 3184 | 3066 | 3984.061219 | 4631.276262 | 1.162451079 | 0.780130762 | unchanged |
| hsa_circ_102115 | 637 | 904 | 1195 | 537 | 832 | 845 | 911.8773223 | 738.1294802 | 0.809461385 | 0.412085692 | unchanged |
| hsa_circ_102116 | 255 | 381 | 348 | 430 | 260 | 249 | 328.2936347 | 313.0529473 | 0.95357605 | 0.837733815 | unchanged |
| hsa_circ_102117 | 88 | 49 | 73 | 72 | 73 | 69 | 70.1081447 | 71.48693824 | 1.019666667 | 0.908306249 | unchanged |
| hsa_circ_102119 | 955 | 519 | 504 | 625 | 688 | 664 | 659.5421922 | 659.2451309 | 0.999549595 | 0.998506131 | unchanged |
| hsa_circ_102120 | 217 | 135 | 172 | 179 | 149 | 161 | 174.6556848 | 162.9556543 | 0.933010881 | 0.665375441 | unchanged |
| hsa_circ_102121 | 1787 | 3644 | 4276 | 1802 | 2895 | 2993 | 3235.808829 | 2563.240808 | 0.792148407 | 0.467628084 | unchanged |
| hsa_circ_102122 | 2983 | 2765 | 3433 | 2968 | 3165 | 3322 | 3060.676015 | 3151.735805 | 1.029751529 | 0.702304122 | unchanged |
| hsa_circ_102123 | 82 | 71 | 99 | 67 | 93 | 95 | 84.04198534 | 84.77859005 | 1.008764723 | 0.954910958 | unchanged |
| hsa_circ_102124 | 60 | 54 | 54 | 65 | 60 | 56 | 55.96195938 | 60.32596386 | 1.077981624 | 0.227002961 | unchanged |
| hsa_circ_102126 | 322 | 311 | 295 | 312 | 396 | 388 | 309.5295828 | 365.594322 | 1.181128856 | 0.113907754 | unchanged |
| hsa_circ_102128 | 257 | 303 | 267 | 307 | 327 | 392 | 275.7413266 | 341.8995325 | 1.239928511 | 0.086822914 | unchanged |
| hsa_circ_102129 | 75 | 55 | 69 | 74 | 68 | 74 | 66.37374286 | 71.99267368 | 1.084655928 | 0.422394524 | unchanged |
| hsa_circ_102133 | 386 | 209 | 253 | 388 | 337 | 319 | 282.806921 | 347.9564493 | 1.230367517 | 0.318595236 | unchanged |
| hsa_circ_102134 | 123 | 82 | 102 | 135 | 123 | 122 | 102.1739426 | 126.4411557 | 1.237508826 | 0.124557557 | unchanged |
| hsa_circ_102135 | 113 | 67 | 127 | 107 | 127 | 133 | 102.3130536 | 122.5894991 | 1.198180436 | 0.366979774 | unchanged |
| hsa_circ_102136 | 73 | 88 | 91 | 66 | 95 | 99 | 83.85674912 | 86.65804663 | 1.033405749 | 0.823794105 | unchanged |
| hsa_circ_102137 | 337 | 292 | 267 | 299 | 338 | 334 | 298.3092927 | 323.6643444 | 1.084995849 | 0.349519852 | unchanged |
| hsa_circ_102138 | 727 | 611 | 562 | 723 | 749 | 761 | 633.1922424 | 744.5834461 | 1.175920039 | 0.091540365 | unchanged |
| hsa_circ_102139 | 174 | 216 | 169 | 195 | 231 | 251 | 186.2545263 | 225.585148 | 1.211165991 | 0.14775248 | unchanged |
| hsa_circ_102140 | 442 | 549 | 314 | 533 | 413 | 513 | 434.9765238 | 486.4820933 | 1.118409998 | 0.54255814 | unchanged |
| hsa_circ_102141 | 82 | 68 | 86 | 80 | 100 | 101 | 78.59568806 | 93.39855076 | 1.188341919 | 0.16529817 | unchanged |
| hsa_circ_102142 | 189 | 218 | 278 | 268 | 377 | 356 | 228.3575018 | 333.7041384 | 1.461323301 | 0.067830345 | unchanged |
| hsa_circ_102143 | 82 | 82 | 96 | 93 | 130 | 143 | 86.75615322 | 122.0777318 | 1.407136293 | 0.088832616 | unchanged |
| hsa_circ_102144 | 195 | 255 | 319 | 274 | 393 | 368 | 256.5700239 | 345.2144003 | 1.34549779 | 0.157705216 | unchanged |
| hsa_circ_102146 | 107 | 155 | 158 | 108 | 147 | 143 | 139.8581673 | 132.6912553 | 0.948755857 | 0.74507097 | unchanged |
| hsa_circ_102147 | 234 | 159 | 173 | 155 | 115 | 114 | 188.6805489 | 128.2059707 | 0.67948695 | 0.085832861 | unchanged |
| hsa_circ_102148 | 2844 | 4014 | 4717 | 3587 | 4638 | 4623 | 3858.087561 | 4282.490597 | 1.110003474 | 0.548131986 | unchanged |
| hsa_circ_102150 | 86 | 71 | 106 | 90 | 116 | 105 | 87.56746616 | 103.8119751 | 1.185508496 | 0.276990973 | unchanged |
| hsa_circ_102152 | 68 | 70 | 147 | 67 | 66 | 64 | 95.13167208 | 65.50346691 | 0.688555825 | 0.316262879 | unchanged |
| hsa_circ_102153 | 2448 | 3897 | 3708 | 2771 | 4349 | 4009 | 3350.798835 | 3709.840166 | 1.107150966 | 0.61567244 | unchanged |
| hsa_circ_102154 | 157 | 181 | 143 | 175 | 122 | 159 | 160.3010519 | 151.7729545 | 0.946799492 | 0.677055356 | unchanged |
| hsa_circ_102155 | 94 | 73 | 98 | 101 | 96 | 105 | 88.41458821 | 100.7340154 | 1.139337042 | 0.218245567 | unchanged |
| hsa_circ_102156 | 145 | 156 | 138 | 177 | 168 | 156 | 146.4931665 | 167.2914478 | 1.141974413 | 0.061699313 | unchanged |
| hsa_circ_102157 | 563 | 849 | 835 | 580 | 780 | 783 | 749.0081553 | 714.4077481 | 0.953805033 | 0.778273264 | unchanged |
| hsa_circ_102158 | 945 | 1501 | 1798 | 753 | 1564 | 1556 | 1414.609458 | 1291.26649 | 0.91280776 | 0.753746736 | unchanged |
| hsa_circ_102159 | 188 | 269 | 304 | 187 | 235 | 233 | 253.8346654 | 218.2171633 | 0.859682278 | 0.398701257 | unchanged |
| hsa_circ_102160 | 359 | 376 | 368 | 365 | 441 | 414 | 367.777208 | 406.6778343 | 1.105772259 | 0.164486469 | unchanged |
| hsa_circ_102161 | 184 | 115 | 134 | 187 | 164 | 170 | 144.4339049 | 173.6332989 | 1.202164402 | 0.252735342 | unchanged |
| hsa_circ_102162 | 107 | 67 | 87 | 102 | 110 | 99 | 86.92795785 | 103.664918 | 1.192538288 | 0.240612176 | unchanged |
| hsa_circ_102163 | 77 | 88 | 89 | 81 | 82 | 87 | 84.9006861 | 83.2876524 | 0.981000934 | 0.726052747 | unchanged |
| hsa_circ_102164 | 142 | 110 | 138 | 149 | 179 | 169 | 129.9699897 | 165.8568607 | 1.276116594 | 0.056583706 | unchanged |
| hsa_circ_102166 | 128 | 141 | 130 | 136 | 124 | 127 | 133.0725571 | 129.0309268 | 0.969628372 | 0.487824383 | unchanged |
| hsa_circ_102167 | 111 | 163 | 138 | 119 | 129 | 137 | 137.2885095 | 128.3826985 | 0.935130689 | 0.605132239 | unchanged |
| hsa_circ_102168 | 67 | 59 | 117 | 68 | 73 | 68 | 80.8147248 | 69.69553941 | 0.862411393 | 0.572067985 | unchanged |
| hsa_circ_102170 | 67 | 68 | 68 | 68 | 75 | 72 | 67.57478415 | 71.30975247 | 1.055271628 | 0.142544208 | unchanged |
| hsa_circ_102171 | 427 | 313 | 436 | 374 | 386 | 381 | 391.853532 | 380.2773526 | 0.970457892 | 0.784522165 | unchanged |
| hsa_circ_102173 | 494 | 785 | 487 | 570 | 714 | 716 | 588.7743124 | 666.6134429 | 1.132205378 | 0.516466362 | unchanged |
| hsa_circ_102175 | 191 | 113 | 173 | 178 | 188 | 193 | 158.8293799 | 186.283906 | 1.172855464 | 0.316383431 | unchanged |
| hsa_circ_102178 | 146 | 173 | 157 | 167 | 160 | 155 | 158.9755137 | 160.3931176 | 1.008917121 | 0.876248116 | unchanged |
| hsa_circ_102179 | 198 | 247 | 184 | 212 | 188 | 183 | 209.6348145 | 194.2389539 | 0.926558665 | 0.505462443 | unchanged |
| hsa_circ_102180 | 110 | 118 | 97 | 113 | 97 | 98 | 108.2696186 | 102.4179284 | 0.945952611 | 0.512588673 | unchanged |
| hsa_circ_102183 | 68 | 80 | 73 | 72 | 76 | 78 | 73.48370832 | 75.42083861 | 1.026361357 | 0.651558716 | unchanged |
| hsa_circ_102184 | 70 | 94 | 60 | 68 | 68 | 68 | 74.91485764 | 67.76675675 | 0.904583669 | 0.517677828 | unchanged |
| hsa_circ_102185 | 80 | 65 | 65 | 79 | 60 | 64 | 70.1342698 | 67.75644879 | 0.966096161 | 0.773552095 | unchanged |
| hsa_circ_102187 | 63 | 68 | 117 | 54 | 60 | 57 | 82.5750652 | 57.30833072 | 0.694014962 | 0.2176588 | unchanged |
| hsa_circ_102188 | 103 | 92 | 108 | 94 | 106 | 107 | 100.9149347 | 102.4180958 | 1.014895328 | 0.816555287 | unchanged |
| hsa_circ_102189 | 145 | 94 | 104 | 189 | 107 | 102 | 114.5384895 | 132.6275861 | 1.157930288 | 0.6051919 | unchanged |
| hsa_circ_102190 | 170 | 137 | 148 | 162 | 118 | 131 | 151.6102632 | 137.2028981 | 0.904971043 | 0.418410034 | unchanged |
| hsa_circ_102191 | 397 | 999 | 655 | 437 | 680 | 679 | 683.8463264 | 598.7289439 | 0.875531418 | 0.680598521 | unchanged |
| hsa_circ_102192 | 98 | 91 | 87 | 88 | 89 | 85 | 91.85833931 | 87.26646054 | 0.950011302 | 0.260176834 | unchanged |
| hsa_circ_102193 | 181 | 208 | 151 | 206 | 107 | 114 | 180.2740027 | 142.7467995 | 0.791832418 | 0.355921493 | unchanged |
| hsa_circ_102194 | 62 | 83 | 60 | 66 | 70 | 69 | 68.16980075 | 68.51120585 | 1.005008157 | 0.966239189 | unchanged |
| hsa_circ_102195 | 730 | 969 | 686 | 732 | 613 | 669 | 794.9157275 | 671.4612888 | 0.844694935 | 0.260802371 | unchanged |
| hsa_circ_102196 | 605 | 406 | 275 | 548 | 284 | 297 | 428.9260448 | 376.4793682 | 0.877725596 | 0.704613403 | unchanged |
| hsa_circ_102197 | 131 | 150 | 109 | 124 | 105 | 111 | 129.6638404 | 113.4711803 | 0.875118151 | 0.285115202 | unchanged |
| hsa_circ_102199 | 82 | 88 | 62 | 65 | 63 | 62 | 77.36661079 | 63.30881086 | 0.818296294 | 0.146881485 | unchanged |
| hsa_circ_102200 | 79 | 129 | 78 | 79 | 69 | 72 | 95.20331628 | 73.15318808 | 0.768389074 | 0.270901647 | unchanged |
| hsa_circ_102201 | 357 | 216 | 254 | 328 | 309 | 321 | 275.3673556 | 319.3734422 | 1.159808655 | 0.358178041 | unchanged |
| hsa_circ_102202 | 131 | 177 | 137 | 127 | 156 | 145 | 148.2546041 | 142.5120976 | 0.961265914 | 0.747868306 | unchanged |
| hsa_circ_102203 | 148 | 121 | 97 | 176 | 105 | 104 | 122.1479344 | 128.4903276 | 1.051923868 | 0.833059107 | unchanged |
| hsa_circ_102205 | 7685 | 5659 | 3862 | 6173 | 4580 | 4503 | 5735.198611 | 5085.716828 | 0.886755137 | 0.625706281 | unchanged |
| hsa_circ_102206 | 176 | 115 | 101 | 153 | 102 | 104 | 130.5812891 | 119.7522884 | 0.917070808 | 0.721578216 | unchanged |
| hsa_circ_102207 | 63 | 73 | 70 | 60 | 63 | 60 | 68.74570777 | 60.630955 | 0.881959863 | 0.054322431 | unchanged |
| hsa_circ_102208 | 177 | 208 | 230 | 173 | 240 | 236 | 204.9983675 | 216.1819702 | 1.054554594 | 0.696685962 | unchanged |
| hsa_circ_102209 | 909 | 938 | 800 | 957 | 962 | 981 | 882.3344472 | 966.7010141 | 1.095617447 | 0.118852893 | unchanged |
| hsa_circ_102210 | 211 | 211 | 243 | 244 | 308 | 274 | 222.0081195 | 275.2353585 | 1.239753569 | 0.067000385 | unchanged |
| hsa_circ_102211 | 222 | 322 | 336 | 275 | 403 | 413 | 293.2523198 | 363.70823 | 1.240256958 | 0.284531683 | unchanged |
| hsa_circ_102212 | 547 | 319 | 363 | 442 | 331 | 322 | 409.4858565 | 365.1087613 | 0.891627282 | 0.607972243 | unchanged |
| hsa_circ_102213 | 504 | 358 | 390 | 452 | 319 | 324 | 417.4593146 | 364.8960128 | 0.874087606 | 0.445959552 | unchanged |
| hsa_circ_102214 | 246 | 279 | 235 | 214 | 233 | 242 | 253.1136768 | 229.8894458 | 0.908245847 | 0.211017589 | unchanged |
| hsa_circ_102215 | 106 | 102 | 83 | 92 | 74 | 75 | 97.20341837 | 80.37707953 | 0.826895606 | 0.142473384 | unchanged |
| hsa_circ_102216 | 122 | 112 | 95 | 122 | 79 | 73 | 109.9024046 | 91.27701946 | 0.830527956 | 0.342181905 | unchanged |
| hsa_circ_102217 | 1569 | 1284 | 1273 | 1443 | 1250 | 1265 | 1375.262611 | 1319.356335 | 0.959348654 | 0.652390104 | unchanged |
| hsa_circ_102218 | 166 | 262 | 209 | 176 | 229 | 221 | 212.5146591 | 208.5325343 | 0.981261882 | 0.907850165 | unchanged |
| hsa_circ_102219 | 4259 | 5315 | 3736 | 4056 | 3555 | 3601 | 4436.807975 | 3737.373213 | 0.842356314 | 0.227337319 | unchanged |
| hsa_circ_102220 | 1025 | 1207 | 862 | 982 | 1045 | 993 | 1031.488956 | 1006.751688 | 0.976017903 | 0.819496844 | unchanged |
| hsa_circ_102221 | 62 | 89 | 76 | 66 | 76 | 81 | 75.72602921 | 74.44098617 | 0.983030365 | 0.892782807 | unchanged |
| hsa_circ_102222 | 86 | 78 | 83 | 83 | 81 | 80 | 82.12021918 | 81.54520419 | 0.992997888 | 0.836291445 | unchanged |
| hsa_circ_102224 | 941 | 476 | 596 | 1235 | 556 | 556 | 670.9498352 | 782.4915843 | 1.166244544 | 0.69610686 | unchanged |
| hsa_circ_102225 | 389 | 62 | 84 | 85 | 72 | 75 | 178.3128055 | 77.14016696 | 0.432611482 | 0.392712169 | unchanged |
| hsa_circ_102226 | 936 | 530 | 954 | 1375 | 1031 | 1014 | 806.4300474 | 1140.202962 | 1.413889482 | 0.139860801 | unchanged |
| hsa_circ_102228 | 198 | 161 | 415 | 227 | 215 | 214 | 257.9893396 | 218.6400984 | 0.847477259 | 0.645150854 | unchanged |
| hsa_circ_102229 | 973 | 1511 | 1320 | 1159 | 1846 | 2033 | 1267.908971 | 1679.222232 | 1.324402832 | 0.253740433 | unchanged |
| hsa_circ_102230 | 107 | 123 | 143 | 118 | 137 | 142 | 124.2542702 | 132.3288318 | 1.064984178 | 0.560264516 | unchanged |
| hsa_circ_102231 | 63 | 57 | 68 | 63 | 60 | 62 | 62.85179941 | 61.94987299 | 0.985649951 | 0.798312943 | unchanged |
| hsa_circ_102232 | 113 | 171 | 121 | 101 | 132 | 122 | 135.0181166 | 118.3654907 | 0.876663767 | 0.461016614 | unchanged |
| hsa_circ_102234 | 349 | 638 | 493 | 416 | 684 | 678 | 493.398659 | 592.5418451 | 1.20093931 | 0.460651313 | unchanged |
| hsa_circ_102235 | 127 | 156 | 153 | 127 | 136 | 139 | 145.2529912 | 133.8346618 | 0.921390057 | 0.308564042 | unchanged |
| hsa_circ_102236 | 71 | 85 | 98 | 75 | 97 | 97 | 84.59872437 | 89.61976279 | 1.059351231 | 0.663536935 | unchanged |
| hsa_circ_102237 | 5139 | 1478 | 643 | 3751 | 672 | 734 | 2420.134335 | 1718.947328 | 0.710269386 | 0.703481256 | unchanged |
| hsa_circ_102238 | 148 | 195 | 139 | 128 | 135 | 129 | 160.5246714 | 130.5753522 | 0.81342856 | 0.161120708 | unchanged |
| hsa_circ_102239 | 208 | 157 | 209 | 276 | 195 | 194 | 191.3467584 | 221.8148466 | 1.159229707 | 0.39811526 | unchanged |
| hsa_circ_102240 | 113 | 129 | 137 | 127 | 135 | 142 | 126.3890713 | 134.789498 | 1.066464819 | 0.357120553 | unchanged |
| hsa_circ_102243 | 323 | 121 | 176 | 256 | 191 | 181 | 206.8499502 | 209.2978423 | 1.011834144 | 0.971704124 | unchanged |
| hsa_circ_102244 | 119 | 168 | 124 | 122 | 108 | 105 | 136.8309428 | 111.42844 | 0.814351182 | 0.19504111 | unchanged |
| hsa_circ_102245 | 124 | 87 | 82 | 135 | 90 | 93 | 98.01481514 | 105.9334658 | 1.080790344 | 0.707777709 | unchanged |
| hsa_circ_102246 | 127 | 143 | 89 | 131 | 84 | 81 | 119.4972599 | 98.91699518 | 0.827776262 | 0.417205435 | unchanged |
| hsa_circ_102248 | 569 | 238 | 234 | 337 | 193 | 190 | 347.1646929 | 239.9325952 | 0.691120382 | 0.426445607 | unchanged |
| hsa_circ_102249 | 108 | 112 | 124 | 101 | 121 | 124 | 114.6269125 | 115.4970093 | 1.007590686 | 0.925549708 | unchanged |
| hsa_circ_102250 | 114 | 126 | 104 | 111 | 94 | 94 | 114.4524967 | 99.64012286 | 0.870580597 | 0.155789017 | unchanged |
| hsa_circ_102251 | 152 | 148 | 294 | 160 | 88 | 89 | 197.7359946 | 112.0787407 | 0.566810008 | 0.184754535 | unchanged |
| hsa_circ_102254 | 151 | 102 | 160 | 151 | 186 | 193 | 137.8784953 | 176.7113419 | 1.281645419 | 0.156525551 | unchanged |
| hsa_circ_102255 | 171 | 378 | 398 | 224 | 367 | 362 | 315.4936461 | 317.3193377 | 1.005786778 | 0.984165423 | unchanged |
| hsa_circ_102256 | 85 | 79 | 85 | 92 | 84 | 89 | 83.06673065 | 88.24916477 | 1.062388806 | 0.161047847 | unchanged |
| hsa_circ_102257 | 166 | 196 | 177 | 165 | 144 | 168 | 179.8088639 | 158.7801872 | 0.883049833 | 0.140857725 | unchanged |
| hsa_circ_102259 | 176 | 110 | 163 | 165 | 197 | 198 | 149.3083908 | 186.9273389 | 1.251954681 | 0.174662296 | unchanged |
| hsa_circ_102260 | 145 | 209 | 147 | 139 | 133 | 155 | 166.7902571 | 142.5888459 | 0.854899131 | 0.331235735 | unchanged |
| hsa_circ_102261 | 144 | 130 | 120 | 134 | 123 | 132 | 131.3047143 | 129.5756719 | 0.986831833 | 0.832542091 | unchanged |
| hsa_circ_102262 | 165 | 110 | 106 | 143 | 108 | 104 | 127.3132062 | 118.2560897 | 0.928859568 | 0.71126632 | unchanged |
| hsa_circ_102263 | 136 | 279 | 169 | 138 | 158 | 160 | 194.7811991 | 152.0347346 | 0.780541117 | 0.385701839 | unchanged |
| hsa_circ_102264 | 336 | 467 | 488 | 444 | 571 | 665 | 430.2958444 | 560.3608423 | 1.302268775 | 0.177649129 | unchanged |
| hsa_circ_102265 | 374 | 507 | 446 | 503 | 568 | 710 | 442.291739 | 593.8282424 | 1.342616626 | 0.102923021 | unchanged |
| hsa_circ_102266 | 80 | 95 | 105 | 78 | 96 | 95 | 93.22893639 | 89.71343065 | 0.962291689 | 0.724981582 | unchanged |
| hsa_circ_102267 | 95 | 66 | 111 | 93 | 112 | 113 | 90.68114413 | 105.9991711 | 1.168921853 | 0.352257908 | unchanged |
| hsa_circ_102268 | 113 | 112 | 175 | 108 | 114 | 118 | 133.2379722 | 113.5729567 | 0.852406823 | 0.402028401 | unchanged |
| hsa_circ_102269 | 77 | 98 | 86 | 88 | 80 | 85 | 87.12270869 | 84.3090219 | 0.967704324 | 0.681981489 | unchanged |
| hsa_circ_102270 | 85 | 76 | 84 | 82 | 76 | 76 | 81.76393817 | 77.74941555 | 0.950901061 | 0.293624421 | unchanged |
| hsa_circ_102271 | 101 | 104 | 133 | 102 | 125 | 133 | 112.6280294 | 119.9450811 | 1.064966525 | 0.62041365 | unchanged |
| hsa_circ_102273 | 201 | 160 | 143 | 152 | 118 | 123 | 167.625824 | 131.2311965 | 0.782881738 | 0.14624333 | unchanged |
| hsa_circ_102283 | 94 | 113 | 77 | 91 | 83 | 82 | 94.74924686 | 85.30145192 | 0.900286332 | 0.426541887 | unchanged |
| hsa_circ_102284 | 74 | 118 | 64 | 73 | 70 | 66 | 85.34280029 | 69.58485024 | 0.815357007 | 0.395113138 | unchanged |
| hsa_circ_102286 | 97 | 71 | 106 | 109 | 102 | 95 | 91.23538401 | 101.7318361 | 1.11504804 | 0.406391364 | unchanged |
| hsa_circ_102287 | 116 | 129 | 129 | 126 | 146 | 160 | 124.8017292 | 143.8265079 | 1.152440025 | 0.15403306 | unchanged |
| hsa_circ_102288 | 138 | 69 | 80 | 115 | 73 | 70 | 95.9770579 | 85.88862416 | 0.894887029 | 0.717182175 | unchanged |
| hsa_circ_102289 | 351 | 249 | 291 | 310 | 352 | 372 | 296.9466572 | 344.5294067 | 1.160240058 | 0.242285308 | unchanged |
| hsa_circ_102290 | 347 | 150 | 225 | 350 | 410 | 384 | 240.5244504 | 381.1218799 | 1.584545269 | 0.07850327 | unchanged |
| hsa_circ_102291 | 2206 | 1857 | 1586 | 2314 | 1984 | 1805 | 1882.889083 | 2034.276433 | 1.080401629 | 0.551582708 | unchanged |
| hsa_circ_102292 | 1124 | 1229 | 1283 | 1296 | 1504 | 1451 | 1212.017204 | 1416.90587 | 1.169047656 | 0.058413038 | unchanged |
| hsa_circ_102294 | 104 | 80 | 90 | 89 | 96 | 91 | 91.37139692 | 92.07322086 | 1.007681003 | 0.927363109 | unchanged |
| hsa_circ_102295 | 243 | 278 | 255 | 252 | 233 | 232 | 258.622403 | 238.9376725 | 0.923886213 | 0.185240062 | unchanged |
| hsa_circ_102296 | 116 | 117 | 120 | 137 | 113 | 143 | 117.5800178 | 131.2171868 | 1.115982029 | 0.21159894 | unchanged |
| hsa_circ_102297 | 224 | 356 | 341 | 293 | 373 | 376 | 306.9490011 | 347.2615106 | 1.131332923 | 0.464641314 | unchanged |
| hsa_circ_102298 | 69 | 66 | 71 | 72 | 70 | 72 | 68.90629973 | 71.34803225 | 1.035435548 | 0.202666697 | unchanged |
| hsa_circ_102299 | 2027 | 1778 | 1572 | 1983 | 1698 | 1761 | 1792.27183 | 1814.206802 | 1.012238641 | 0.896064629 | unchanged |
| hsa_circ_102301 | 754 | 861 | 646 | 708 | 655 | 663 | 753.4039858 | 675.0992911 | 0.896065463 | 0.290251273 | unchanged |
| hsa_circ_102303 | 118 | 87 | 110 | 134 | 131 | 132 | 104.8352199 | 132.5834683 | 1.264684411 | 0.04039249 | unchanged |
| hsa_circ_102304 | 421 | 288 | 289 | 305 | 290 | 290 | 332.7721995 | 295.0748489 | 0.886717248 | 0.445294783 | unchanged |
| hsa_circ_102305 | 130 | 111 | 150 | 111 | 158 | 164 | 130.0173665 | 144.4387181 | 1.110918656 | 0.511852363 | unchanged |
| hsa_circ_102306 | 87 | 59 | 68 | 83 | 59 | 72 | 71.62309761 | 71.1429944 | 0.99329681 | 0.966900063 | unchanged |
| hsa_circ_102309 | 694 | 207 | 132 | 271 | 105 | 104 | 344.1387548 | 159.987211 | 0.464891584 | 0.37481363 | unchanged |
| hsa_circ_102310 | 97 | 88 | 80 | 89 | 90 | 82 | 88.32887424 | 87.01670819 | 0.98514454 | 0.828393566 | unchanged |
| hsa_circ_102311 | 114 | 111 | 84 | 118 | 117 | 122 | 102.7977109 | 118.800588 | 1.155673477 | 0.167196431 | unchanged |
| hsa_circ_102312 | 72 | 60 | 70 | 75 | 106 | 73 | 67.44574692 | 84.35104736 | 1.250650355 | 0.20930301 | unchanged |
| hsa_circ_102313 | 1008 | 1199 | 1107 | 1024 | 1237 | 1179 | 1104.928042 | 1146.837121 | 1.037929239 | 0.644198332 | unchanged |
| hsa_circ_102314 | 169 | 113 | 94 | 143 | 97 | 104 | 125.3250781 | 114.6746854 | 0.915017865 | 0.710506726 | unchanged |
| hsa_circ_102315 | 71 | 95 | 76 | 76 | 84 | 89 | 80.4635671 | 83.0476149 | 1.032114507 | 0.768755825 | unchanged |
| hsa_circ_102317 | 68 | 69 | 71 | 69 | 61 | 64 | 69.40986925 | 64.53165107 | 0.929718666 | 0.110661506 | unchanged |
| hsa_circ_102318 | 73 | 74 | 79 | 79 | 77 | 77 | 75.16162118 | 77.7149158 | 1.033970723 | 0.282036469 | unchanged |
| hsa_circ_102319 | 120 | 75 | 95 | 121 | 108 | 111 | 96.76779213 | 113.3059126 | 1.170905216 | 0.2920826 | unchanged |
| hsa_circ_102320 | 118 | 142 | 126 | 116 | 145 | 138 | 128.4869419 | 133.1244345 | 1.036093105 | 0.699477886 | unchanged |
| hsa_circ_102322 | 504 | 863 | 690 | 479 | 724 | 809 | 685.537827 | 670.6917403 | 0.978343884 | 0.922410696 | unchanged |
| hsa_circ_102323 | 9200 | 11764 | 13978 | 8180 | 12692 | 10941 | 11647.34699 | 10604.52013 | 0.910466576 | 0.613253317 | unchanged |
| hsa_circ_102324 | 7684 | 2777 | 2851 | 7655 | 3404 | 3320 | 4437.480811 | 4793.095674 | 1.080138907 | 0.877443296 | unchanged |
| hsa_circ_102325 | 137 | 189 | 146 | 164 | 184 | 209 | 157.2235481 | 185.474085 | 1.179683878 | 0.244160037 | unchanged |
| hsa_circ_102327 | 66 | 75 | 67 | 66 | 61 | 61 | 69.3040918 | 62.68029471 | 0.904424156 | 0.112117521 | unchanged |
| hsa_circ_102328 | 137 | 118 | 146 | 107 | 131 | 132 | 133.6833321 | 123.1861937 | 0.921477582 | 0.423881063 | unchanged |
| hsa_circ_102329 | 100 | 110 | 109 | 93 | 91 | 100 | 106.1487932 | 94.8805392 | 0.893844728 | 0.053573334 | unchanged |
| hsa_circ_102330 | 88 | 72 | 99 | 95 | 114 | 123 | 86.41678578 | 110.7044067 | 1.281052121 | 0.099687368 | unchanged |
| hsa_circ_102331 | 263 | 349 | 249 | 290 | 223 | 243 | 286.9424506 | 251.9853921 | 0.878173974 | 0.399446611 | unchanged |
| hsa_circ_102332 | 993 | 636 | 637 | 760 | 646 | 670 | 755.3077453 | 691.8964809 | 0.916045791 | 0.635613738 | unchanged |
| hsa_circ_102333 | 544 | 313 | 315 | 1346 | 355 | 341 | 390.7688447 | 680.6444749 | 1.741808448 | 0.443890744 | unchanged |
| hsa_circ_102334 | 383 | 481 | 429 | 394 | 417 | 415 | 431.0722178 | 408.7323168 | 0.948175967 | 0.4869328 | unchanged |
| hsa_circ_102335 | 113 | 104 | 126 | 121 | 159 | 181 | 114.4546544 | 153.6177698 | 1.342171453 | 0.102495183 | unchanged |
| hsa_circ_102340 | 116 | 98 | 122 | 106 | 85 | 87 | 112.2853976 | 92.55315431 | 0.824267058 | 0.118378725 | unchanged |
| hsa_circ_102341 | 200 | 186 | 226 | 208 | 192 | 196 | 204.1532366 | 198.8350972 | 0.973950257 | 0.698288622 | unchanged |
| hsa_circ_102343 | 1199 | 660 | 623 | 866 | 692 | 647 | 827.3766909 | 735.0466521 | 0.888406285 | 0.664920095 | unchanged |
| hsa_circ_102344 | 155 | 84 | 116 | 130 | 139 | 137 | 118.3633415 | 135.494463 | 1.144733337 | 0.455450443 | unchanged |
| hsa_circ_102345 | 1047 | 1034 | 1020 | 1016 | 1386 | 1274 | 1033.627736 | 1225.520188 | 1.18564948 | 0.155347771 | unchanged |
| hsa_circ_102346 | 267 | 147 | 114 | 209 | 117 | 120 | 175.754475 | 148.6599994 | 0.845839057 | 0.651079837 | unchanged |
| hsa_circ_102347 | 85 | 75 | 124 | 98 | 134 | 128 | 94.64016002 | 120.1221193 | 1.269251016 | 0.246566115 | unchanged |
| hsa_circ_102350 | 82 | 86 | 106 | 93 | 126 | 147 | 91.11207327 | 122.3355158 | 1.342692702 | 0.145940659 | unchanged |
| hsa_circ_102352 | 527 | 616 | 503 | 550 | 444 | 486 | 548.9517411 | 493.1905084 | 0.898422341 | 0.292455784 | unchanged |
| hsa_circ_102353 | 91 | 65 | 90 | 91 | 99 | 98 | 82.30055324 | 96.14342989 | 1.168199072 | 0.194186452 | unchanged |
| hsa_circ_102354 | 183 | 148 | 129 | 184 | 148 | 141 | 153.5338843 | 157.5644149 | 1.026251733 | 0.854188175 | unchanged |
| hsa_circ_102355 | 283 | 239 | 286 | 291 | 330 | 337 | 269.1274827 | 319.3163365 | 1.186487286 | 0.074219787 | unchanged |
| hsa_circ_102358 | 67 | 86 | 192 | 65 | 69 | 70 | 115.1451596 | 67.9653553 | 0.590258032 | 0.293208958 | unchanged |
| hsa_circ_102359 | 9982 | 4752 | 3358 | 9015 | 4342 | 4346 | 6030.626925 | 5900.913038 | 0.978490812 | 0.961827861 | unchanged |
| hsa_circ_102361 | 93 | 100 | 135 | 115 | 139 | 154 | 109.5034628 | 136.1449929 | 1.243293951 | 0.199353448 | unchanged |
| hsa_circ_102362 | 225 | 262 | 212 | 222 | 249 | 242 | 233.1010193 | 237.8283581 | 1.020280215 | 0.794191729 | unchanged |
| hsa_circ_102363 | 68 | 161 | 89 | 62 | 71 | 73 | 105.8903136 | 68.519356 | 0.647078601 | 0.257122313 | unchanged |
| hsa_circ_102364 | 7285 | 7308 | 8486 | 7138 | 9437 | 8617 | 7692.856735 | 8397.309682 | 1.091572347 | 0.418077081 | unchanged |
| hsa_circ_102366 | 73 | 64 | 65 | 73 | 68 | 63 | 67.59278714 | 67.59887648 | 1.000090089 | 0.998856531 | unchanged |
| hsa_circ_102369 | 99 | 137 | 98 | 98 | 91 | 98 | 111.5964387 | 95.83620903 | 0.858774798 | 0.296371416 | unchanged |
| hsa_circ_102370 | 175 | 128 | 142 | 196 | 170 | 171 | 148.5221525 | 178.9739043 | 1.205031715 | 0.136382274 | unchanged |
| hsa_circ_102371 | 135 | 140 | 160 | 145 | 168 | 180 | 144.6582655 | 164.4562866 | 1.136860628 | 0.198641157 | unchanged |
| hsa_circ_102372 | 65 | 60 | 157 | 62 | 70 | 71 | 94.24937839 | 67.76895976 | 0.719038798 | 0.451282911 | unchanged |
| hsa_circ_102373 | 408 | 393 | 220 | 474 | 302 | 334 | 340.5652326 | 370.0842194 | 1.086676454 | 0.731200462 | unchanged |
| hsa_circ_102374 | 655 | 319 | 336 | 430 | 230 | 227 | 436.6267508 | 295.7883193 | 0.677439755 | 0.334425409 | unchanged |
| hsa_circ_102375 | 119 | 132 | 142 | 126 | 147 | 132 | 131.1570001 | 135.0041821 | 1.029332647 | 0.698056638 | unchanged |
| hsa_circ_102376 | 179 | 284 | 185 | 231 | 175 | 191 | 216.0050354 | 198.9251835 | 0.920928455 | 0.676970865 | unchanged |
| hsa_circ_102377 | 76 | 79 | 88 | 74 | 95 | 92 | 80.82256183 | 86.8132584 | 1.074121587 | 0.470589113 | unchanged |
| hsa_circ_102378 | 78 | 159 | 92 | 76 | 96 | 102 | 109.720597 | 91.20044109 | 0.831206205 | 0.519230629 | unchanged |
| hsa_circ_102379 | 208 | 94 | 126 | 142 | 100 | 102 | 142.3239591 | 114.5904968 | 0.805138485 | 0.489597653 | unchanged |
| hsa_circ_102382 | 337 | 427 | 475 | 337 | 563 | 510 | 413.0976077 | 470.1727704 | 1.138163867 | 0.51287326 | unchanged |
| hsa_circ_102383 | 255 | 152 | 193 | 268 | 270 | 242 | 199.9976026 | 260.0301197 | 1.300166183 | 0.127477884 | unchanged |
| hsa_circ_102384 | 95 | 135 | 106 | 92 | 98 | 95 | 111.7811998 | 94.99033339 | 0.8497881 | 0.233405553 | unchanged |
| hsa_circ_102385 | 5105 | 1830 | 1297 | 2790 | 1172 | 1242 | 2743.838942 | 1734.391122 | 0.632103837 | 0.481533153 | unchanged |
| hsa_circ_102386 | 120 | 204 | 158 | 139 | 156 | 164 | 160.4980544 | 153.1269747 | 0.954073713 | 0.785536576 | unchanged |
| hsa_circ_102387 | 132 | 118 | 176 | 131 | 161 | 171 | 142.1966961 | 154.4280244 | 1.086016966 | 0.597650902 | unchanged |
| hsa_circ_102388 | 113 | 94 | 142 | 110 | 142 | 146 | 116.3913972 | 132.8121892 | 1.141082523 | 0.415780305 | unchanged |
| hsa_circ_102389 | 94 | 90 | 127 | 91 | 145 | 126 | 103.4620458 | 120.4179254 | 1.163885022 | 0.436208979 | unchanged |
| hsa_circ_102390 | 182 | 146 | 159 | 185 | 187 | 191 | 162.1900863 | 187.5587841 | 1.156413369 | 0.078736647 | unchanged |
| hsa_circ_102391 | 79 | 69 | 66 | 75 | 87 | 77 | 71.34060441 | 79.50663778 | 1.114465436 | 0.197631124 | unchanged |
| hsa_circ_102392 | 585 | 770 | 594 | 816 | 529 | 550 | 649.6439082 | 631.5766375 | 0.972188963 | 0.87783514 | unchanged |
| hsa_circ_102393 | 2167 | 6523 | 6791 | 2579 | 5389 | 5520 | 5160.373465 | 4495.900619 | 0.871235512 | 0.727757741 | unchanged |
| hsa_circ_102394 | 106 | 155 | 139 | 118 | 135 | 162 | 133.331288 | 138.5213638 | 1.038926166 | 0.802492307 | unchanged |
| hsa_circ_102395 | 83 | 112 | 99 | 80 | 93 | 93 | 97.97761012 | 88.91617718 | 0.907515269 | 0.383520456 | unchanged |
| hsa_circ_102396 | 1290 | 1484 | 1329 | 1209 | 1301 | 1363 | 1367.979398 | 1291.141066 | 0.943830783 | 0.359392753 | unchanged |
| hsa_circ_102397 | 414 | 264 | 275 | 368 | 349 | 331 | 317.7562801 | 349.4408086 | 1.099713304 | 0.556869733 | unchanged |
| hsa_circ_102398 | 484 | 570 | 448 | 445 | 400 | 372 | 500.6538508 | 405.6795065 | 0.810299383 | 0.086297997 | unchanged |
| hsa_circ_102399 | 377 | 121 | 107 | 296 | 102 | 103 | 201.4190434 | 167.1398551 | 0.829811582 | 0.768570927 | unchanged |
| hsa_circ_102400 | 155 | 294 | 237 | 184 | 212 | 218 | 228.5687043 | 204.8734114 | 0.896331858 | 0.600336616 | unchanged |
| hsa_circ_102401 | 84 | 140 | 100 | 84 | 78 | 78 | 108.0815185 | 80.22469126 | 0.742260956 | 0.170490111 | unchanged |
| hsa_circ_102402 | 97 | 151 | 136 | 80 | 92 | 91 | 128.1318792 | 87.80035158 | 0.685234246 | 0.070753402 | unchanged |
| hsa_circ_102403 | 533 | 484 | 349 | 539 | 425 | 460 | 455.3550188 | 474.4674787 | 1.041972657 | 0.781343908 | unchanged |
| hsa_circ_102404 | 8256 | 10978 | 12702 | 8491 | 14242 | 13901 | 10645.62121 | 12211.31407 | 1.147073884 | 0.528021657 | unchanged |
| hsa_circ_102405 | 67 | 66 | 77 | 69 | 66 | 69 | 70.17299466 | 67.8004817 | 0.966190513 | 0.557722286 | unchanged |
| hsa_circ_102406 | 122 | 68 | 99 | 103 | 87 | 94 | 96.21826859 | 94.4114425 | 0.98122159 | 0.917437428 | unchanged |
| hsa_circ_102407 | 107 | 129 | 154 | 100 | 122 | 123 | 129.8612281 | 114.9626574 | 0.885273142 | 0.392241848 | unchanged |
| hsa_circ_102408 | 140 | 115 | 100 | 119 | 89 | 87 | 118.2791812 | 98.17211763 | 0.830003358 | 0.268561498 | unchanged |
| hsa_circ_102410 | 102 | 136 | 140 | 98 | 108 | 113 | 125.9956276 | 106.3311162 | 0.843927033 | 0.201088068 | unchanged |
| hsa_circ_102411 | 6001 | 9556 | 12282 | 6046 | 12680 | 13596 | 9279.448689 | 10774.08416 | 1.161069425 | 0.643856044 | unchanged |
| hsa_circ_102413 | 3945 | 3888 | 3822 | 4045 | 3811 | 3664 | 3885.150973 | 3839.737091 | 0.98831091 | 0.71657479 | unchanged |
| hsa_circ_102414 | 2506 | 5151 | 6552 | 2733 | 5520 | 5567 | 4736.272898 | 4606.830621 | 0.972670013 | 0.935881408 | unchanged |
| hsa_circ_102415 | 1808 | 1684 | 1400 | 2491 | 1727 | 1706 | 1630.888898 | 1974.463202 | 1.210666898 | 0.294337798 | unchanged |
| hsa_circ_102416 | 282 | 256 | 203 | 292 | 197 | 196 | 247.1060628 | 228.3995088 | 0.924297471 | 0.658605331 | unchanged |
| hsa_circ_102417 | 747 | 1110 | 971 | 1014 | 1050 | 1055 | 942.8249586 | 1039.627026 | 1.102672364 | 0.415925806 | unchanged |
| hsa_circ_102419 | 133 | 125 | 123 | 150 | 137 | 134 | 127.1952472 | 140.6762776 | 1.105986903 | 0.080466356 | unchanged |
| hsa_circ_102420 | 104 | 119 | 126 | 94 | 139 | 125 | 116.4469943 | 119.4919616 | 1.026148956 | 0.847552822 | unchanged |
| hsa_circ_102421 | 147 | 210 | 204 | 179 | 209 | 224 | 186.8451569 | 204.013983 | 1.091887991 | 0.514621946 | unchanged |
| hsa_circ_102422 | 342 | 441 | 373 | 276 | 363 | 400 | 385.1378486 | 346.350434 | 0.899289528 | 0.456318079 | unchanged |
| hsa_circ_102423 | 656 | 570 | 746 | 794 | 603 | 725 | 657.3630603 | 707.1302617 | 1.075707329 | 0.546372749 | unchanged |
| hsa_circ_102424 | 1219 | 1828 | 2199 | 1629 | 1952 | 2119 | 1748.618489 | 1899.716993 | 1.086410217 | 0.661379453 | unchanged |
| hsa_circ_102425 | 75 | 90 | 210 | 74 | 81 | 81 | 124.9655793 | 78.80398934 | 0.630605562 | 0.342679307 | unchanged |
| hsa_circ_102427 | 219 | 336 | 310 | 219 | 309 | 296 | 288.0565611 | 274.7679645 | 0.953868099 | 0.783064119 | unchanged |
| hsa_circ_102428 | 151 | 170 | 122 | 156 | 142 | 141 | 147.7088377 | 146.2882849 | 0.99038275 | 0.927549822 | unchanged |
| hsa_circ_102429 | 206 | 394 | 321 | 209 | 266 | 259 | 307.2128485 | 244.6129369 | 0.796232769 | 0.338819468 | unchanged |
| hsa_circ_102430 | 11579 | 13422 | 16554 | 12405 | 17740 | 18038 | 13851.64616 | 16060.70775 | 1.159480077 | 0.397930503 | unchanged |
| hsa_circ_102431 | 8545 | 10079 | 11894 | 8182 | 11191 | 11189 | 10172.82756 | 10187.26357 | 1.001419076 | 0.9922305 | unchanged |
| hsa_circ_102432 | 569 | 295 | 332 | 323 | 181 | 197 | 398.6326206 | 233.5086934 | 0.585774172 | 0.163669131 | unchanged |
| hsa_circ_102433 | 829 | 846 | 850 | 587 | 882 | 867 | 841.7155631 | 778.7696298 | 0.925217097 | 0.547955932 | unchanged |
| hsa_circ_102435 | 81 | 63 | 101 | 75 | 93 | 96 | 81.9966164 | 87.97442321 | 1.072903091 | 0.660369367 | unchanged |
| hsa_circ_102436 | 154 | 294 | 126 | 161 | 133 | 132 | 191.3460117 | 141.8750434 | 0.741458064 | 0.40189313 | unchanged |
| hsa_circ_102437 | 90 | 76 | 88 | 88 | 86 | 92 | 84.55580301 | 88.69969709 | 1.049007802 | 0.418906172 | unchanged |
| hsa_circ_102438 | 826 | 918 | 688 | 853 | 861 | 841 | 810.3948734 | 851.4939617 | 1.050714892 | 0.57272271 | unchanged |
| hsa_circ_102439 | 731 | 941 | 748 | 786 | 879 | 872 | 806.5942077 | 845.646237 | 1.048415956 | 0.624150204 | unchanged |
| hsa_circ_102440 | 198 | 206 | 295 | 182 | 225 | 229 | 232.7402028 | 212.1600213 | 0.911574445 | 0.581627458 | unchanged |
| hsa_circ_102441 | 844 | 1131 | 1246 | 978 | 1634 | 1596 | 1073.733721 | 1402.645401 | 1.306325185 | 0.248890673 | unchanged |
| hsa_circ_102442 | 4242 | 3576 | 4756 | 3619 | 3948 | 4144 | 4191.348678 | 3903.569619 | 0.931339748 | 0.484932695 | unchanged |
| hsa_circ_102443 | 64 | 75 | 55 | 63 | 66 | 67 | 64.72799632 | 65.38610801 | 1.010167342 | 0.919374131 | unchanged |
| hsa_circ_102444 | 59 | 134 | 62 | 63 | 57 | 60 | 85.29526988 | 59.84351405 | 0.701604135 | 0.360371954 | unchanged |
| hsa_circ_102445 | 12429 | 6686 | 7075 | 8745 | 5329 | 5194 | 8730.149484 | 6422.652619 | 0.735686443 | 0.350898007 | unchanged |
| hsa_circ_102446 | 3196 | 1265 | 1040 | 2194 | 1663 | 1660 | 1833.689524 | 1839.412399 | 1.003120961 | 0.993929936 | unchanged |
| hsa_circ_102447 | 70 | 85 | 64 | 65 | 59 | 61 | 72.68788145 | 61.66948547 | 0.848414952 | 0.165058671 | unchanged |
| hsa_circ_102448 | 1444 | 2040 | 2029 | 1628 | 2405 | 2382 | 1837.69834 | 2138.429097 | 1.163645333 | 0.403383446 | unchanged |
| hsa_circ_102449 | 327 | 419 | 398 | 292 | 385 | 379 | 381.3327157 | 351.6388602 | 0.922131372 | 0.509081125 | unchanged |
| hsa_circ_102450 | 198 | 105 | 117 | 152 | 101 | 104 | 139.6855626 | 118.9422881 | 0.851500226 | 0.568727202 | unchanged |
| hsa_circ_102451 | 3845 | 3591 | 2681 | 4625 | 2000 | 1972 | 3372.739685 | 2865.490635 | 0.849603261 | 0.620990751 | unchanged |
| hsa_circ_102452 | 176 | 242 | 348 | 184 | 237 | 247 | 255.1312128 | 222.6210654 | 0.872574794 | 0.576851448 | unchanged |
| hsa_circ_102454 | 91 | 100 | 98 | 89 | 92 | 90 | 96.53475362 | 90.45594694 | 0.937029863 | 0.120268895 | unchanged |
| hsa_circ_102455 | 236 | 234 | 368 | 340 | 530 | 518 | 279.4157647 | 462.6409193 | 1.655743797 | 0.072968702 | unchanged |
| hsa_circ_102456 | 1547 | 2013 | 1640 | 1939 | 2191 | 2189 | 1733.117234 | 2106.462739 | 1.215418494 | 0.086880164 | unchanged |
| hsa_circ_102457 | 476 | 403 | 440 | 437 | 505 | 510 | 439.6080187 | 484.0358971 | 1.101062484 | 0.231257295 | unchanged |
| hsa_circ_102459 | 129 | 112 | 105 | 135 | 109 | 108 | 115.4645247 | 117.2750783 | 1.015680605 | 0.883199894 | unchanged |
| hsa_circ_102460 | 61 | 99 | 55 | 59 | 52 | 52 | 71.62270531 | 54.22414751 | 0.757080416 | 0.278060697 | unchanged |
| hsa_circ_102461 | 66 | 75 | 56 | 58 | 49 | 53 | 65.46673148 | 53.61026002 | 0.818893181 | 0.121442342 | unchanged |
| hsa_circ_102462 | 1841 | 2691 | 2997 | 2080 | 2596 | 2721 | 2509.814227 | 2465.927974 | 0.982514143 | 0.917420533 | unchanged |
| hsa_circ_102463 | 203 | 249 | 253 | 182 | 222 | 243 | 234.729194 | 215.3578141 | 0.917473495 | 0.465019552 | unchanged |
| hsa_circ_102464 | 175 | 128 | 80 | 139 | 75 | 75 | 127.3559821 | 96.59619705 | 0.758473967 | 0.425078687 | unchanged |
| hsa_circ_102465 | 5703 | 4166 | 2419 | 4707 | 2499 | 2513 | 4096.002456 | 3239.662325 | 0.790932711 | 0.514703956 | unchanged |
| hsa_circ_102466 | 2004 | 2047 | 2522 | 2529 | 2040 | 2025 | 2190.858247 | 2197.791934 | 1.003164827 | 0.977807745 | unchanged |
| hsa_circ_102467 | 117 | 151 | 207 | 127 | 238 | 206 | 158.5687999 | 190.5388085 | 1.201616009 | 0.489709614 | unchanged |
| hsa_circ_102471 | 116 | 254 | 179 | 103 | 134 | 138 | 183.0669139 | 125.0678668 | 0.683181161 | 0.233620243 | unchanged |
| hsa_circ_102472 | 91 | 74 | 109 | 96 | 115 | 105 | 91.43236283 | 105.4483458 | 1.153293457 | 0.293727963 | unchanged |
| hsa_circ_102473 | 2846 | 2567 | 2008 | 2420 | 2406 | 2456 | 2473.71441 | 2427.109416 | 0.981159913 | 0.85943438 | unchanged |
| hsa_circ_102474 | 99 | 73 | 111 | 111 | 114 | 114 | 94.15636258 | 112.9928785 | 1.200055688 | 0.168313604 | unchanged |
| hsa_circ_102475 | 199 | 237 | 189 | 123 | 160 | 172 | 208.2336461 | 151.742197 | 0.728711233 | 0.054012473 | unchanged |
| hsa_circ_102476 | 263 | 484 | 376 | 633 | 415 | 441 | 374.4050901 | 496.3439158 | 1.32568688 | 0.26362889 | unchanged |
| hsa_circ_102477 | 122 | 110 | 144 | 137 | 145 | 145 | 124.9281246 | 142.184102 | 1.138127243 | 0.168445207 | unchanged |
| hsa_circ_102478 | 273 | 503 | 422 | 265 | 397 | 416 | 399.1628913 | 359.4732337 | 0.900567767 | 0.654874318 | unchanged |
| hsa_circ_102479 | 906 | 1665 | 1395 | 935 | 1546 | 1603 | 1322.169764 | 1361.454962 | 1.029712673 | 0.904760673 | unchanged |
| hsa_circ_102480 | 151 | 416 | 399 | 172 | 270 | 286 | 321.6711222 | 242.4139912 | 0.753608187 | 0.441242321 | unchanged |
| hsa_circ_102481 | 63 | 105 | 79 | 60 | 79 | 79 | 81.98840558 | 72.45683533 | 0.883744901 | 0.526146585 | unchanged |
| hsa_circ_102482 | 9934 | 8601 | 5857 | 8727 | 7057 | 7210 | 8130.637375 | 7664.555788 | 0.942675886 | 0.740569251 | unchanged |
| hsa_circ_102483 | 322 | 533 | 675 | 434 | 666 | 653 | 509.9109659 | 584.1687627 | 1.145628947 | 0.5910059 | unchanged |
| hsa_circ_102485 | 763 | 1293 | 1262 | 1120 | 1264 | 1255 | 1105.873746 | 1213.085309 | 1.096947381 | 0.579351826 | unchanged |
| hsa_circ_102486 | 121 | 163 | 142 | 159 | 195 | 190 | 141.7694974 | 181.4280616 | 1.279739753 | 0.076323626 | unchanged |
| hsa_circ_102487 | 118 | 294 | 163 | 124 | 152 | 151 | 191.7798222 | 142.514271 | 0.743114001 | 0.410506666 | unchanged |
| hsa_circ_102488 | 67 | 81 | 77 | 70 | 79 | 79 | 75.17477479 | 76.26984949 | 1.01456705 | 0.846248982 | unchanged |
| hsa_circ_102489 | 59 | 91 | 61 | 60 | 61 | 67 | 70.26119961 | 62.62618312 | 0.891333815 | 0.508691065 | unchanged |
| hsa_circ_102490 | 191 | 138 | 135 | 158 | 147 | 150 | 154.3204266 | 151.5586477 | 0.982103608 | 0.887790971 | unchanged |
| hsa_circ_102491 | 151 | 115 | 126 | 138 | 144 | 145 | 130.5425066 | 142.6122829 | 1.092458592 | 0.325239515 | unchanged |
| hsa_circ_102492 | 490 | 712 | 727 | 515 | 878 | 891 | 643.2699286 | 761.5519197 | 1.18387614 | 0.460630492 | unchanged |
| hsa_circ_102494 | 2367 | 848 | 444 | 2609 | 558 | 634 | 1219.824368 | 1267.139428 | 1.03878842 | 0.960184943 | unchanged |
| hsa_circ_102496 | 80 | 58 | 83 | 85 | 82 | 79 | 73.68146237 | 81.94637193 | 1.112170813 | 0.372488915 | unchanged |
| hsa_circ_102497 | 99 | 154 | 103 | 90 | 105 | 101 | 118.6824565 | 98.65634163 | 0.831263057 | 0.332808965 | unchanged |
| hsa_circ_102499 | 220 | 183 | 226 | 230 | 234 | 239 | 209.7853565 | 234.1851621 | 1.116308431 | 0.152849593 | unchanged |
| hsa_circ_102500 | 211 | 269 | 233 | 229 | 229 | 253 | 237.9378678 | 237.055904 | 0.996293302 | 0.96478132 | unchanged |
| hsa_circ_102502 | 75 | 92 | 108 | 71 | 70 | 68 | 91.51206389 | 69.62207606 | 0.760796698 | 0.0820524 | unchanged |
| hsa_circ_102503 | 74 | 85 | 84 | 77 | 83 | 75 | 80.86012453 | 78.48477767 | 0.970624002 | 0.604391239 | unchanged |
| hsa_circ_102504 | 462 | 287 | 311 | 449 | 387 | 391 | 353.1479259 | 408.8014785 | 1.157592749 | 0.392767592 | unchanged |
| hsa_circ_102505 | 95 | 102 | 106 | 91 | 104 | 107 | 100.947718 | 100.7346265 | 0.99788909 | 0.97169199 | unchanged |
| hsa_circ_102506 | 127 | 175 | 136 | 135 | 167 | 188 | 146.0089894 | 163.4795925 | 1.119654298 | 0.462789884 | unchanged |
| hsa_circ_102507 | 71 | 103 | 69 | 75 | 71 | 67 | 81.14157296 | 71.14722158 | 0.876828227 | 0.42441006 | unchanged |
| hsa_circ_102508 | 83 | 53 | 71 | 70 | 66 | 67 | 69.08841521 | 67.86975132 | 0.982360807 | 0.894757103 | unchanged |
| hsa_circ_102509 | 15292 | 4518 | 2928 | 11409 | 2780 | 2761 | 7579.459208 | 5650.046023 | 0.745441841 | 0.710214197 | unchanged |
| hsa_circ_102512 | 111 | 154 | 245 | 113 | 141 | 157 | 169.9688824 | 136.8982384 | 0.805431185 | 0.471856549 | unchanged |
| hsa_circ_102513 | 32907 | 25434 | 22488 | 31870 | 22691 | 21645 | 26943.25048 | 25401.96346 | 0.94279506 | 0.74870176 | unchanged |
| hsa_circ_102514 | 108 | 157 | 103 | 117 | 118 | 105 | 122.5636776 | 113.3193785 | 0.92457554 | 0.632199076 | unchanged |
| hsa_circ_102519 | 59 | 56 | 59 | 60 | 56 | 56 | 57.92407405 | 57.30895912 | 0.989380669 | 0.715774476 | unchanged |
| hsa_circ_102520 | 256 | 129 | 167 | 208 | 126 | 131 | 184.2369448 | 154.839201 | 0.840435132 | 0.556484043 | unchanged |
| hsa_circ_102521 | 99 | 100 | 106 | 77 | 100 | 92 | 101.7316774 | 89.64144543 | 0.881155681 | 0.171714473 | unchanged |
| hsa_circ_102525 | 79 | 72 | 78 | 82 | 94 | 107 | 76.25593081 | 94.48681689 | 1.239074992 | 0.069501623 | unchanged |
| hsa_circ_102526 | 16418 | 18650 | 20389 | 14871 | 18666 | 18753 | 18485.55697 | 17430.38981 | 0.942919374 | 0.57271444 | unchanged |
| hsa_circ_102527 | 117 | 90 | 134 | 118 | 132 | 137 | 113.4695519 | 128.8820381 | 1.135829268 | 0.336659814 | unchanged |
| hsa_circ_102528 | 1114 | 959 | 817 | 917 | 806 | 862 | 963.5359777 | 861.695731 | 0.894305715 | 0.328464807 | unchanged |
| hsa_circ_102529 | 140 | 84 | 99 | 120 | 81 | 80 | 107.6868628 | 93.73398083 | 0.870430973 | 0.547676924 | unchanged |
| hsa_circ_102530 | 82 | 71 | 68 | 86 | 82 | 81 | 73.66267552 | 82.8375466 | 1.124552509 | 0.111189018 | unchanged |
| hsa_circ_102531 | 289 | 141 | 138 | 270 | 168 | 173 | 189.0610874 | 203.3746253 | 1.075708535 | 0.822628253 | unchanged |
| hsa_circ_102533 | 1329 | 869 | 386 | 1062 | 451 | 364 | 861.519634 | 625.7448505 | 0.72632686 | 0.537141868 | unchanged |
| hsa_circ_102534 | 669 | 412 | 289 | 571 | 317 | 322 | 456.4761131 | 403.4554272 | 0.883847841 | 0.723975676 | unchanged |
| hsa_circ_102535 | 327 | 213 | 314 | 483 | 345 | 337 | 284.5332925 | 388.6468932 | 1.365910083 | 0.155017626 | unchanged |
| hsa_circ_102536 | 551 | 667 | 540 | 548 | 554 | 585 | 586.3411453 | 562.0803878 | 0.958623478 | 0.595963451 | unchanged |
| hsa_circ_102537 | 103 | 198 | 119 | 117 | 125 | 130 | 140.1942621 | 123.8638905 | 0.88351612 | 0.609911623 | unchanged |
| hsa_circ_102538 | 896 | 981 | 864 | 839 | 806 | 829 | 913.3945173 | 824.7559638 | 0.90295699 | 0.071533259 | unchanged |
| hsa_circ_102539 | 99 | 155 | 129 | 126 | 120 | 127 | 127.9825509 | 124.213706 | 0.970551885 | 0.828492499 | unchanged |
| hsa_circ_102540 | 317 | 297 | 282 | 265 | 151 | 149 | 298.5483255 | 188.3697078 | 0.63095215 | 0.048983901 | unchanged |
| hsa_circ_102541 | 252 | 410 | 415 | 280 | 482 | 456 | 358.9766556 | 405.8215172 | 1.130495565 | 0.602332022 | unchanged |
| hsa_circ_102542 | 71 | 65 | 76 | 63 | 80 | 71 | 70.65676684 | 71.14553228 | 1.006917461 | 0.936852631 | unchanged |
| hsa_circ_102543 | 15723 | 13146 | 11360 | 20109 | 16899 | 16387 | 13409.84259 | 17798.18562 | 1.327247915 | 0.063279755 | unchanged |
| hsa_circ_102544 | 444 | 647 | 692 | 421 | 831 | 745 | 594.2636394 | 665.6065872 | 1.120052689 | 0.651558751 | unchanged |
| hsa_circ_102545 | 963 | 1401 | 1231 | 1225 | 1543 | 1558 | 1198.755031 | 1442.173214 | 1.203059155 | 0.219651588 | unchanged |
| hsa_circ_102546 | 410 | 910 | 725 | 580 | 778 | 714 | 681.9772808 | 690.3871307 | 1.012331569 | 0.959873125 | unchanged |
| hsa_circ_102547 | 1405 | 2685 | 2503 | 1369 | 2258 | 2304 | 2197.485011 | 1976.957425 | 0.899645466 | 0.683405191 | unchanged |
| hsa_circ_102548 | 62 | 58 | 69 | 60 | 74 | 77 | 63.03285501 | 70.37858177 | 1.116538062 | 0.317375782 | unchanged |
| hsa_circ_102549 | 69 | 86 | 81 | 71 | 82 | 86 | 78.78824105 | 79.70561223 | 1.011643504 | 0.898804137 | unchanged |
| hsa_circ_102550 | 861 | 841 | 817 | 866 | 712 | 708 | 839.8531334 | 762.0296288 | 0.907336769 | 0.221156235 | unchanged |
| hsa_circ_102551 | 438 | 274 | 249 | 345 | 217 | 200 | 320.2594444 | 254.2058393 | 0.793749704 | 0.427777839 | unchanged |
| hsa_circ_102552 | 82 | 61 | 58 | 66 | 51 | 57 | 66.85784216 | 58.09894542 | 0.868992231 | 0.368743808 | unchanged |
| hsa_circ_102554 | 1612 | 2708 | 2238 | 1967 | 3047 | 2521 | 2186.105767 | 2511.895296 | 1.149027341 | 0.50470285 | unchanged |
| hsa_circ_102555 | 136 | 178 | 177 | 145 | 197 | 190 | 163.940783 | 176.9923461 | 1.079611447 | 0.574672518 | unchanged |
| hsa_circ_102556 | 83 | 95 | 84 | 77 | 92 | 97 | 87.74891515 | 88.72844202 | 1.011162837 | 0.897211482 | unchanged |
| hsa_circ_102557 | 95 | 85 | 88 | 84 | 93 | 100 | 89.35235161 | 92.4162971 | 1.034290597 | 0.603901705 | unchanged |
| hsa_circ_102558 | 107 | 60 | 69 | 73 | 65 | 65 | 78.79572951 | 67.73273128 | 0.859599012 | 0.497598004 | unchanged |
| hsa_circ_102559 | 897 | 1092 | 1697 | 1038 | 1852 | 1827 | 1228.771735 | 1572.139342 | 1.279439702 | 0.394035038 | unchanged |
| hsa_circ_102560 | 199 | 189 | 215 | 213 | 263 | 233 | 200.9140206 | 236.1225267 | 1.175241658 | 0.098585945 | unchanged |
| hsa_circ_102561 | 66 | 73 | 81 | 80 | 70 | 67 | 73.25493155 | 72.36987053 | 0.987918069 | 0.882655601 | unchanged |
| hsa_circ_102562 | 17504 | 30343 | 29441 | 20627 | 26975 | 26162 | 25762.6729 | 24587.76275 | 0.954394866 | 0.810716401 | unchanged |
| hsa_circ_102563 | 3119 | 3526 | 3569 | 3299 | 3940 | 3837 | 3404.355434 | 3691.926816 | 1.084471609 | 0.305459448 | unchanged |
| hsa_circ_102564 | 117 | 139 | 99 | 100 | 87 | 93 | 118.5075408 | 93.06166177 | 0.785280507 | 0.105023336 | unchanged |
| hsa_circ_102565 | 222 | 167 | 121 | 157 | 117 | 103 | 169.7333918 | 125.6456077 | 0.740252736 | 0.257865234 | unchanged |
| hsa_circ_102566 | 72 | 76 | 74 | 67 | 75 | 80 | 74.33588281 | 74.28027563 | 0.999251947 | 0.989400451 | unchanged |
| hsa_circ_102567 | 474 | 481 | 371 | 423 | 343 | 333 | 441.9578285 | 366.1553541 | 0.828484825 | 0.171403602 | unchanged |
| hsa_circ_102568 | 61 | 62 | 64 | 58 | 63 | 62 | 62.60346668 | 60.89299266 | 0.972677647 | 0.403459133 | unchanged |
| hsa_circ_102570 | 1208 | 1206 | 977 | 1458 | 1119 | 1054 | 1129.974467 | 1210.1928 | 1.070991279 | 0.614502057 | unchanged |
| hsa_circ_102571 | 99 | 173 | 107 | 97 | 90 | 94 | 126.3182022 | 93.84110401 | 0.74289455 | 0.236006507 | unchanged |
| hsa_circ_102572 | 105 | 113 | 74 | 98 | 74 | 74 | 97.37996234 | 82.0795074 | 0.842878816 | 0.344051204 | unchanged |
| hsa_circ_102573 | 81 | 154 | 93 | 78 | 85 | 91 | 109.2482514 | 84.70524556 | 0.775346465 | 0.341696969 | unchanged |
| hsa_circ_102574 | 1152 | 1778 | 1491 | 1251 | 1750 | 1725 | 1473.711884 | 1575.419034 | 1.06901427 | 0.696874633 | unchanged |
| hsa_circ_102575 | 265 | 211 | 198 | 222 | 161 | 167 | 224.3512411 | 183.4191221 | 0.81755341 | 0.21883081 | unchanged |
| hsa_circ_102576 | 1752 | 2171 | 1574 | 1629 | 1698 | 1664 | 1832.390117 | 1663.582947 | 0.907875966 | 0.396423898 | unchanged |
| hsa_circ_102577 | 74 | 70 | 69 | 73 | 74 | 68 | 71.05605427 | 71.49536381 | 1.006182577 | 0.875192943 | unchanged |
| hsa_circ_102578 | 251 | 325 | 214 | 217 | 160 | 169 | 263.6580829 | 182.1056913 | 0.690688824 | 0.092154316 | unchanged |
| hsa_circ_102579 | 2160 | 2363 | 2121 | 2222 | 2635 | 2736 | 2214.295098 | 2530.823663 | 1.142947778 | 0.143309663 | unchanged |
| hsa_circ_102581 | 383 | 524 | 338 | 364 | 323 | 312 | 414.9701564 | 332.6099188 | 0.801527324 | 0.230048324 | unchanged |
| hsa_circ_102582 | 259 | 178 | 119 | 165 | 109 | 109 | 185.1495486 | 127.7032224 | 0.689730131 | 0.269237871 | unchanged |
| hsa_circ_102583 | 157 | 241 | 206 | 146 | 170 | 182 | 201.2220662 | 165.9023171 | 0.824473778 | 0.252604649 | unchanged |
| hsa_circ_102584 | 186 | 298 | 217 | 190 | 197 | 220 | 233.5070304 | 202.4182106 | 0.866861312 | 0.41988839 | unchanged |
| hsa_circ_102585 | 111 | 92 | 93 | 117 | 102 | 108 | 98.65175461 | 109.034475 | 1.105246181 | 0.239773345 | unchanged |
| hsa_circ_102586 | 254 | 320 | 345 | 268 | 458 | 482 | 306.671652 | 402.5091978 | 1.312508656 | 0.259059403 | unchanged |
| hsa_circ_102587 | 6289 | 12070 | 12439 | 7487 | 14447 | 14923 | 10266.11558 | 12285.68661 | 1.196722023 | 0.552810086 | unchanged |
| hsa_circ_102589 | 274 | 382 | 398 | 318 | 492 | 492 | 351.0827428 | 433.8341952 | 1.23570356 | 0.301418426 | unchanged |
| hsa_circ_102590 | 732 | 1335 | 1196 | 685 | 1034 | 1047 | 1087.537208 | 921.9767735 | 0.847765729 | 0.488657044 | unchanged |
| hsa_circ_102591 | 319 | 376 | 652 | 349 | 386 | 480 | 448.9321424 | 405.0854236 | 0.902331077 | 0.710285952 | unchanged |
| hsa_circ_102592 | 763 | 358 | 427 | 449 | 352 | 332 | 515.6673027 | 377.7385113 | 0.732523682 | 0.349142333 | unchanged |
| hsa_circ_102594 | 72 | 65 | 64 | 70 | 53 | 56 | 66.95839245 | 59.75786922 | 0.892462723 | 0.289699858 | unchanged |
| hsa_circ_102595 | 72 | 82 | 90 | 79 | 85 | 81 | 81.22077875 | 81.77360508 | 1.006806464 | 0.92624525 | unchanged |
| hsa_circ_102596 | 131 | 109 | 73 | 129 | 69 | 75 | 104.3596934 | 90.99793402 | 0.871964367 | 0.625783696 | unchanged |
| hsa_circ_102597 | 298 | 223 | 234 | 211 | 187 | 200 | 251.607124 | 199.3483539 | 0.792300117 | 0.097862067 | unchanged |
| hsa_circ_102598 | 2015 | 840 | 584 | 2049 | 890 | 917 | 1146.494617 | 1285.611633 | 1.121341185 | 0.823152683 | unchanged |
| hsa_circ_102599 | 3441 | 4902 | 4360 | 3331 | 4129 | 4336 | 4234.492442 | 3931.966902 | 0.928556835 | 0.595467597 | unchanged |
| hsa_circ_102600 | 684 | 401 | 407 | 476 | 310 | 312 | 497.1130967 | 365.88552 | 0.736020681 | 0.291925172 | unchanged |
| hsa_circ_102601 | 666 | 639 | 709 | 627 | 771 | 788 | 671.5116261 | 728.7725026 | 1.085271608 | 0.357081166 | unchanged |
| hsa_circ_102602 | 3219 | 1173 | 1068 | 1674 | 909 | 897 | 1819.987404 | 1159.901177 | 0.63731275 | 0.426056729 | unchanged |
| hsa_circ_102603 | 321 | 217 | 385 | 299 | 202 | 209 | 307.4188788 | 236.4105772 | 0.769017759 | 0.28809388 | unchanged |
| hsa_circ_102604 | 65 | 61 | 58 | 67 | 61 | 62 | 61.3122171 | 63.35185021 | 1.033266341 | 0.503525929 | unchanged |
| hsa_circ_102606 | 70 | 77 | 62 | 65 | 64 | 63 | 69.43769739 | 64.19096537 | 0.924439718 | 0.29454408 | unchanged |
| hsa_circ_102608 | 353 | 476 | 610 | 365 | 672 | 655 | 479.8311424 | 563.7591284 | 1.174911502 | 0.536537118 | unchanged |
| hsa_circ_102609 | 321 | 389 | 569 | 309 | 595 | 602 | 426.4756107 | 502.1349329 | 1.177405977 | 0.567647519 | unchanged |
| hsa_circ_102610 | 599 | 724 | 915 | 498 | 992 | 1006 | 746.0932371 | 832.1203036 | 1.115303373 | 0.674874832 | unchanged |
| hsa_circ_102611 | 788 | 1121 | 1580 | 919 | 1588 | 1623 | 1162.768381 | 1376.420479 | 1.18374433 | 0.546106865 | unchanged |
| hsa_circ_102612 | 168 | 117 | 132 | 175 | 136 | 134 | 138.9601379 | 148.4324288 | 1.068165525 | 0.665679488 | unchanged |
| hsa_circ_102613 | 139 | 211 | 184 | 156 | 140 | 151 | 178.1411432 | 148.9069267 | 0.835892955 | 0.249291887 | unchanged |
| hsa_circ_102614 | 367 | 530 | 437 | 401 | 419 | 484 | 444.7833099 | 434.8627356 | 0.977695714 | 0.862503283 | unchanged |
| hsa_circ_102615 | 405 | 678 | 613 | 377 | 582 | 593 | 565.1892063 | 517.1738645 | 0.915045544 | 0.680053587 | unchanged |
| hsa_circ_102616 | 398 | 591 | 536 | 447 | 731 | 772 | 508.1719406 | 650.0482229 | 1.279189524 | 0.292814186 | unchanged |
| hsa_circ_102617 | 76 | 93 | 79 | 75 | 72 | 72 | 82.57469412 | 73.15286678 | 0.885899337 | 0.151624114 | unchanged |
| hsa_circ_102618 | 74 | 59 | 80 | 83 | 94 | 81 | 71.14994381 | 85.76723192 | 1.205443424 | 0.121412236 | unchanged |
| hsa_circ_102619 | 1757 | 454 | 469 | 278 | 234 | 254 | 893.5221026 | 255.6078091 | 0.286067696 | 0.213887272 | unchanged |
| hsa_circ_102620 | 108 | 241 | 108 | 128 | 121 | 121 | 152.210816 | 123.2385621 | 0.809657062 | 0.548279736 | unchanged |
| hsa_circ_102621 | 192 | 188 | 165 | 197 | 207 | 185 | 181.8596802 | 196.2426071 | 1.079088047 | 0.237591251 | unchanged |
| hsa_circ_102622 | 119 | 57 | 60 | 121 | 76 | 67 | 78.77288966 | 88.09478252 | 1.118338846 | 0.74000966 | unchanged |
| hsa_circ_102624 | 1758 | 1846 | 1507 | 1655 | 1592 | 1512 | 1703.654392 | 1586.422221 | 0.931187821 | 0.345608091 | unchanged |
| hsa_circ_102625 | 152 | 195 | 229 | 142 | 217 | 206 | 191.9075412 | 188.2672989 | 0.98103127 | 0.91613692 | unchanged |
| hsa_circ_102626 | 149 | 118 | 177 | 150 | 202 | 200 | 148.1136583 | 183.9269948 | 1.241796313 | 0.21235474 | unchanged |
| hsa_circ_102627 | 85 | 90 | 99 | 99 | 121 | 118 | 91.53253626 | 112.4410939 | 1.2284276 | 0.060184321 | unchanged |
| hsa_circ_102628 | 227 | 161 | 209 | 201 | 176 | 208 | 198.9822258 | 194.8508437 | 0.979237431 | 0.860232925 | unchanged |
| hsa_circ_102629 | 171 | 102 | 137 | 162 | 123 | 122 | 136.5845854 | 135.4972707 | 0.992039258 | 0.965961653 | unchanged |
| hsa_circ_102631 | 965 | 388 | 256 | 514 | 173 | 185 | 536.3212051 | 290.9556469 | 0.542502598 | 0.372677367 | unchanged |
| hsa_circ_102632 | 154 | 116 | 107 | 135 | 125 | 115 | 125.8658148 | 125.020867 | 0.993286916 | 0.959415355 | unchanged |
| hsa_circ_102633 | 205 | 185 | 303 | 214 | 150 | 146 | 231.0261568 | 169.8968318 | 0.735400849 | 0.223876097 | unchanged |
| hsa_circ_102634 | 112 | 88 | 129 | 110 | 154 | 157 | 109.5928122 | 140.1063957 | 1.278426868 | 0.191248109 | unchanged |
| hsa_circ_102635 | 111 | 61 | 63 | 104 | 76 | 77 | 78.41970274 | 85.4398806 | 1.089520588 | 0.727749097 | unchanged |
| hsa_circ_102636 | 68 | 53 | 61 | 63 | 66 | 61 | 60.7266792 | 63.50955244 | 1.045826205 | 0.575323948 | unchanged |
| hsa_circ_102637 | 92 | 114 | 122 | 106 | 135 | 145 | 109.2797402 | 128.6652911 | 1.177393823 | 0.253477028 | unchanged |
| hsa_circ_102639 | 11770 | 6702 | 6028 | 12174 | 7858 | 7642 | 8166.755746 | 9224.705577 | 1.129543464 | 0.674282037 | unchanged |
| hsa_circ_102641 | 832 | 1037 | 1010 | 785 | 1205 | 1342 | 959.4555347 | 1110.547726 | 1.157477013 | 0.44770167 | unchanged |
| hsa_circ_102643 | 65 | 55 | 78 | 62 | 76 | 70 | 65.836392 | 69.28009566 | 1.052306992 | 0.678431168 | unchanged |
| hsa_circ_102644 | 3219 | 5800 | 7880 | 4387 | 5893 | 6252 | 5633.054472 | 5510.83354 | 0.978302903 | 0.937482325 | unchanged |
| hsa_circ_102645 | 587 | 764 | 521 | 1331 | 632 | 648 | 624.1870697 | 870.1356937 | 1.394030309 | 0.36596562 | unchanged |
| hsa_circ_102646 | 115 | 90 | 89 | 122 | 94 | 91 | 98.21470573 | 102.1253052 | 1.039816843 | 0.781676265 | unchanged |
| hsa_circ_102647 | 124 | 106 | 120 | 136 | 136 | 117 | 116.5871808 | 129.8300292 | 1.113587517 | 0.182747562 | unchanged |
| hsa_circ_102648 | 104 | 88 | 94 | 111 | 108 | 104 | 95.29065512 | 107.715087 | 1.130384578 | 0.060941144 | unchanged |
| hsa_circ_102649 | 293 | 202 | 251 | 283 | 221 | 233 | 248.8928145 | 245.5813854 | 0.986695361 | 0.923801648 | unchanged |
| hsa_circ_102651 | 190 | 108 | 157 | 201 | 216 | 193 | 151.5612776 | 203.1525065 | 1.340398483 | 0.103524119 | unchanged |
| hsa_circ_102653 | 95 | 69 | 118 | 98 | 124 | 122 | 94.20198889 | 114.9288825 | 1.220026072 | 0.276703427 | unchanged |
| hsa_circ_102655 | 94 | 59 | 93 | 86 | 97 | 94 | 82.13311393 | 92.3944488 | 1.124935417 | 0.440520689 | unchanged |
| hsa_circ_102656 | 255 | 156 | 196 | 270 | 247 | 244 | 202.002617 | 253.802918 | 1.256433811 | 0.158381477 | unchanged |
| hsa_circ_102657 | 392 | 339 | 306 | 339 | 177 | 208 | 345.7193801 | 241.1438344 | 0.697513209 | 0.133111621 | unchanged |
| hsa_circ_102658 | 81 | 82 | 94 | 75 | 91 | 88 | 85.78774606 | 84.70533158 | 0.987382645 | 0.87328436 | unchanged |
| hsa_circ_102660 | 100 | 65 | 74 | 121 | 84 | 89 | 79.88701756 | 98.23413644 | 1.229663335 | 0.308462392 | unchanged |
| hsa_circ_102661 | 458 | 288 | 291 | 453 | 421 | 415 | 346.0016224 | 429.687532 | 1.241865657 | 0.218160921 | unchanged |
| hsa_circ_102662 | 85 | 74 | 70 | 75 | 85 | 73 | 76.55479516 | 77.65808636 | 1.014411784 | 0.855338417 | unchanged |
| hsa_circ_102664 | 100 | 87 | 73 | 88 | 62 | 65 | 86.73016962 | 71.81550655 | 0.828033738 | 0.260414065 | unchanged |
| hsa_circ_102666 | 87 | 82 | 91 | 96 | 95 | 112 | 86.5726821 | 101.2584895 | 1.169635583 | 0.073847923 | unchanged |
| hsa_circ_102668 | 65 | 70 | 65 | 69 | 71 | 76 | 66.74861789 | 71.97662039 | 1.078323757 | 0.09191446 | unchanged |
| hsa_circ_102669 | 65 | 92 | 67 | 68 | 73 | 78 | 74.89085518 | 72.97418497 | 0.974407153 | 0.845749159 | unchanged |
| hsa_circ_102670 | 85 | 73 | 121 | 100 | 124 | 124 | 92.91833561 | 115.8870787 | 1.247192795 | 0.233618698 | unchanged |
| hsa_circ_102671 | 60 | 59 | 64 | 65 | 76 | 84 | 61.24884344 | 75.09152227 | 1.226007187 | 0.073416374 | unchanged |
| hsa_circ_102672 | 68 | 62 | 82 | 62 | 77 | 75 | 70.55004194 | 71.36759166 | 1.011588224 | 0.919149995 | unchanged |
| hsa_circ_102673 | 678 | 1143 | 835 | 687 | 962 | 998 | 885.4529485 | 882.1786089 | 0.996302074 | 0.985391753 | unchanged |
| hsa_circ_102676 | 70 | 119 | 80 | 78 | 75 | 83 | 89.5144077 | 78.50414046 | 0.877000055 | 0.503424295 | unchanged |
| hsa_circ_102678 | 90 | 81 | 79 | 92 | 88 | 90 | 83.25379121 | 89.8079232 | 1.078724727 | 0.147709718 | unchanged |
| hsa_circ_102680 | 2600 | 1260 | 1026 | 1266 | 773 | 781 | 1628.592194 | 939.8328005 | 0.577082958 | 0.253572709 | unchanged |
| hsa_circ_102681 | 288 | 218 | 172 | 233 | 174 | 158 | 226.3440836 | 188.6428325 | 0.833433901 | 0.407139551 | unchanged |
| hsa_circ_102682 | 179 | 187 | 155 | 187 | 214 | 211 | 173.6411982 | 203.9822258 | 1.174734037 | 0.078131095 | unchanged |
| hsa_circ_102683 | 136 | 224 | 200 | 155 | 188 | 199 | 186.3040962 | 180.7157277 | 0.970004049 | 0.858559132 | unchanged |
| hsa_circ_102684 | 78 | 77 | 96 | 74 | 94 | 91 | 83.65715783 | 86.27900632 | 1.031340396 | 0.779041054 | unchanged |
| hsa_circ_102685 | 7795 | 3367 | 3271 | 3409 | 2077 | 2075 | 4810.774182 | 2520.447076 | 0.523917145 | 0.215241821 | unchanged |
| hsa_circ_102686 | 1761 | 781 | 515 | 1043 | 594 | 620 | 1018.947309 | 752.3524568 | 0.738362475 | 0.547130174 | unchanged |
| hsa_circ_102687 | 236 | 275 | 244 | 331 | 276 | 301 | 251.482098 | 302.6145672 | 1.203324489 | 0.060439169 | unchanged |
| hsa_circ_102688 | 141 | 106 | 99 | 161 | 115 | 101 | 115.2267868 | 125.589206 | 1.089930645 | 0.663323199 | unchanged |
| hsa_circ_102689 | 2182 | 2666 | 2818 | 2605 | 3223 | 3454 | 2555.544678 | 3094.31213 | 1.210822944 | 0.165358548 | unchanged |
| hsa_circ_102690 | 1513 | 2332 | 2042 | 1586 | 2725 | 2469 | 1962.279989 | 2260.096452 | 1.151770626 | 0.517367607 | unchanged |
| hsa_circ_102691 | 113 | 85 | 113 | 106 | 130 | 113 | 103.9637495 | 116.4864178 | 1.120452257 | 0.347456943 | unchanged |
| hsa_circ_102692 | 77 | 52 | 70 | 82 | 96 | 91 | 66.4390059 | 89.54442693 | 1.347768916 | 0.056745301 | unchanged |
| hsa_circ_102693 | 86 | 74 | 95 | 90 | 112 | 115 | 85.02249805 | 105.6244921 | 1.242312264 | 0.108822927 | unchanged |
| hsa_circ_102694 | 1528 | 461 | 214 | 2202 | 365 | 321 | 734.3101572 | 963.0057118 | 1.311442723 | 0.772533045 | unchanged |
| hsa_circ_102696 | 164 | 175 | 138 | 176 | 125 | 122 | 159.1081804 | 141.156903 | 0.887175647 | 0.436402168 | unchanged |
| hsa_circ_102697 | 116 | 87 | 130 | 98 | 125 | 126 | 111.0737886 | 116.3568556 | 1.04756358 | 0.748260861 | unchanged |
| hsa_circ_102698 | 103 | 72 | 141 | 102 | 127 | 126 | 105.5502126 | 118.7303497 | 1.124870778 | 0.573504063 | unchanged |
| hsa_circ_102699 | 143 | 105 | 109 | 148 | 125 | 135 | 119.1315556 | 136.0252516 | 1.141807062 | 0.291517593 | unchanged |
| hsa_circ_102700 | 64 | 59 | 62 | 64 | 64 | 57 | 61.49205333 | 61.79648502 | 1.004950748 | 0.920652446 | unchanged |
| hsa_circ_102701 | 211 | 300 | 203 | 199 | 220 | 195 | 237.915226 | 204.781099 | 0.860731372 | 0.356406793 | unchanged |
| hsa_circ_102702 | 267 | 471 | 392 | 309 | 427 | 425 | 376.9905668 | 386.5783706 | 1.025432476 | 0.899157104 | unchanged |
| hsa_circ_102704 | 191 | 156 | 162 | 170 | 173 | 186 | 169.9607977 | 176.4474415 | 1.038165529 | 0.617301912 | unchanged |
| hsa_circ_102705 | 341 | 313 | 321 | 340 | 431 | 421 | 324.8709996 | 397.3563884 | 1.223120528 | 0.073618364 | unchanged |
| hsa_circ_102707 | 1224 | 1591 | 1430 | 1336 | 1787 | 1751 | 1415.043423 | 1624.78671 | 1.148223923 | 0.307526522 | unchanged |
| hsa_circ_102708 | 63 | 68 | 85 | 67 | 109 | 111 | 71.60434605 | 95.60520137 | 1.335187131 | 0.203642257 | unchanged |
| hsa_circ_102710 | 79 | 62 | 73 | 79 | 66 | 68 | 71.36662879 | 70.93628126 | 0.993969905 | 0.951307682 | unchanged |
| hsa_circ_102711 | 149 | 226 | 197 | 161 | 241 | 247 | 190.4807604 | 216.6169317 | 1.137211608 | 0.504030079 | unchanged |
| hsa_circ_102713 | 148 | 88 | 88 | 120 | 78 | 79 | 107.8040252 | 92.5650895 | 0.858642238 | 0.565309387 | unchanged |
| hsa_circ_102714 | 221 | 354 | 250 | 222 | 243 | 234 | 275.1157574 | 233.0884599 | 0.847237767 | 0.363465729 | unchanged |
| hsa_circ_102716 | 89 | 62 | 176 | 86 | 77 | 83 | 108.7383245 | 82.26961211 | 0.756583408 | 0.484509585 | unchanged |
| hsa_circ_102719 | 208 | 147 | 357 | 197 | 216 | 209 | 237.4406828 | 207.441924 | 0.873657882 | 0.657346481 | unchanged |
| hsa_circ_102720 | 656 | 626 | 511 | 658 | 643 | 627 | 597.4987367 | 642.9047458 | 1.075993481 | 0.371068944 | unchanged |
| hsa_circ_102721 | 4074 | 5624 | 5317 | 4897 | 6778 | 6196 | 5005.217621 | 5956.939144 | 1.190145883 | 0.262475507 | unchanged |
| hsa_circ_102723 | 813 | 1109 | 1094 | 830 | 1301 | 1313 | 1005.151069 | 1148.015341 | 1.142132139 | 0.484612468 | unchanged |
| hsa_circ_102724 | 186 | 161 | 170 | 178 | 202 | 207 | 172.1379737 | 195.3206688 | 1.134675079 | 0.113335172 | unchanged |
| hsa_circ_102725 | 236 | 256 | 264 | 207 | 234 | 242 | 252.4214324 | 227.5069291 | 0.901297988 | 0.137459149 | unchanged |
| hsa_circ_102726 | 120 | 132 | 156 | 123 | 157 | 157 | 135.7741861 | 145.7059147 | 1.073148872 | 0.557324677 | unchanged |
| hsa_circ_102727 | 104 | 72 | 85 | 112 | 99 | 99 | 87.02776882 | 103.2013788 | 1.185844245 | 0.188531308 | unchanged |
| hsa_circ_102728 | 763 | 375 | 297 | 583 | 177 | 173 | 478.0724911 | 310.9793594 | 0.650485784 | 0.446707534 | unchanged |
| hsa_circ_102731 | 87 | 103 | 79 | 93 | 82 | 82 | 89.72129073 | 85.6554901 | 0.954684105 | 0.639946159 | unchanged |
| hsa_circ_102732 | 106 | 118 | 107 | 113 | 117 | 114 | 110.3610943 | 114.4477586 | 1.037029936 | 0.375654197 | unchanged |
| hsa_circ_102733 | 91 | 119 | 103 | 108 | 105 | 104 | 104.3816078 | 105.6709751 | 1.012352438 | 0.881597748 | unchanged |
| hsa_circ_102734 | 266 | 196 | 218 | 240 | 255 | 253 | 226.448282 | 249.375244 | 1.101245908 | 0.342367524 | unchanged |
| hsa_circ_102735 | 214 | 180 | 374 | 214 | 236 | 268 | 255.8601019 | 239.3128794 | 0.93532707 | 0.802350588 | unchanged |
| hsa_circ_102736 | 235 | 231 | 311 | 264 | 319 | 340 | 259.1384411 | 307.569201 | 1.186891453 | 0.233544172 | unchanged |
| hsa_circ_102737 | 326 | 307 | 358 | 329 | 463 | 475 | 330.4088303 | 422.4911234 | 1.278691986 | 0.134050428 | unchanged |
| hsa_circ_102739 | 95 | 81 | 83 | 95 | 82 | 84 | 86.40053835 | 86.93792853 | 1.006219755 | 0.931756132 | unchanged |
| hsa_circ_102740 | 305 | 193 | 253 | 273 | 305 | 336 | 250.6982481 | 304.8201657 | 1.215884706 | 0.217195865 | unchanged |
| hsa_circ_102742 | 129 | 88 | 166 | 122 | 187 | 181 | 127.2628712 | 163.2185336 | 1.282530655 | 0.305374398 | unchanged |
| hsa_circ_102743 | 141 | 120 | 138 | 149 | 159 | 150 | 132.8917453 | 152.6307096 | 1.148534164 | 0.051598172 | unchanged |
| hsa_circ_102744 | 75 | 84 | 89 | 83 | 80 | 84 | 82.56568919 | 82.41861878 | 0.998218747 | 0.974081012 | unchanged |
| hsa_circ_102745 | 1511 | 5250 | 5104 | 3271 | 5610 | 5925 | 3954.991028 | 4935.30333 | 1.247867137 | 0.544422624 | unchanged |
| hsa_circ_102746 | 4989 | 5700 | 6171 | 4813 | 6636 | 6484 | 5620.154689 | 5977.686985 | 1.063616095 | 0.625636811 | unchanged |
| hsa_circ_102747 | 299 | 183 | 213 | 255 | 122 | 126 | 231.4849969 | 167.7850829 | 0.72482055 | 0.317524924 | unchanged |
| hsa_circ_102748 | 94 | 89 | 75 | 73 | 74 | 78 | 85.96398175 | 74.67918316 | 0.868726432 | 0.126007821 | unchanged |
| hsa_circ_102749 | 166 | 192 | 129 | 179 | 146 | 168 | 162.2008184 | 164.0520391 | 1.01141314 | 0.932519654 | unchanged |
| hsa_circ_102750 | 168 | 131 | 164 | 237 | 167 | 169 | 154.1829037 | 190.9316087 | 1.238344875 | 0.227598672 | unchanged |
| hsa_circ_102751 | 141 | 240 | 207 | 193 | 224 | 229 | 196.2833534 | 215.4253278 | 1.097522149 | 0.571049158 | unchanged |
| hsa_circ_102752 | 66 | 59 | 66 | 64 | 97 | 72 | 63.28588591 | 77.44472988 | 1.223728305 | 0.233164096 | unchanged |
| hsa_circ_102754 | 95 | 135 | 147 | 121 | 160 | 139 | 125.8736803 | 139.8239664 | 1.110827665 | 0.508709828 | unchanged |
| hsa_circ_102755 | 165 | 171 | 179 | 165 | 111 | 113 | 171.8450703 | 129.7635523 | 0.755119435 | 0.079449732 | unchanged |
| hsa_circ_102758 | 66 | 57 | 74 | 72 | 85 | 86 | 65.95322433 | 81.26449384 | 1.232153464 | 0.081820888 | unchanged |
| hsa_circ_102759 | 80 | 87 | 66 | 82 | 74 | 75 | 77.53857487 | 76.97455313 | 0.992725921 | 0.935711238 | unchanged |
| hsa_circ_102760 | 123 | 139 | 437 | 168 | 149 | 139 | 233.0627469 | 152.0568572 | 0.652428838 | 0.472405728 | unchanged |
| hsa_circ_102761 | 73 | 89 | 72 | 77 | 66 | 75 | 77.58778448 | 72.53537753 | 0.934881412 | 0.478433479 | unchanged |
| hsa_circ_102762 | 76 | 70 | 76 | 83 | 66 | 67 | 73.99843551 | 71.99491739 | 0.972924858 | 0.753443622 | unchanged |
| hsa_circ_102763 | 6429 | 4439 | 3666 | 5529 | 4017 | 4006 | 4844.469411 | 4517.041613 | 0.932412041 | 0.75173958 | unchanged |
| hsa_circ_102764 | 271 | 233 | 211 | 253 | 227 | 237 | 238.4166046 | 238.9867234 | 1.002391271 | 0.977528779 | unchanged |
| hsa_circ_102765 | 940 | 824 | 669 | 866 | 777 | 776 | 810.8174124 | 806.4207565 | 0.994577502 | 0.960838 | unchanged |
| hsa_circ_102766 | 1341 | 1028 | 974 | 1117 | 1224 | 1142 | 1114.1567 | 1160.843702 | 1.041903443 | 0.714696271 | unchanged |
| hsa_circ_102767 | 709 | 823 | 714 | 789 | 919 | 965 | 748.9889194 | 890.7783898 | 1.189307835 | 0.092860016 | unchanged |
| hsa_circ_102768 | 80 | 104 | 78 | 75 | 73 | 72 | 87.19484602 | 73.33319956 | 0.841026768 | 0.177910333 | unchanged |
| hsa_circ_102770 | 108 | 91 | 101 | 102 | 128 | 109 | 99.88059691 | 112.7825057 | 1.129173325 | 0.23445225 | unchanged |
| hsa_circ_102772 | 89 | 72 | 89 | 80 | 91 | 90 | 83.29808693 | 86.77744561 | 1.041769971 | 0.620473482 | unchanged |
| hsa_circ_102773 | 159 | 132 | 104 | 161 | 116 | 113 | 131.5234011 | 129.7684903 | 0.986657045 | 0.940747457 | unchanged |
| hsa_circ_102774 | 1994 | 1487 | 1371 | 1400 | 1141 | 1183 | 1617.174199 | 1241.021402 | 0.767401188 | 0.143905572 | unchanged |
| hsa_circ_102775 | 78 | 73 | 109 | 67 | 94 | 85 | 86.67111576 | 81.96399335 | 0.945689837 | 0.747208132 | unchanged |
| hsa_circ_102776 | 59 | 57 | 52 | 60 | 55 | 54 | 56.06564951 | 56.25935701 | 1.003455012 | 0.947280677 | unchanged |
| hsa_circ_102777 | 194 | 205 | 242 | 202 | 238 | 239 | 213.600462 | 226.3036099 | 1.059471537 | 0.543057017 | unchanged |
| hsa_circ_102778 | 107 | 79 | 121 | 94 | 91 | 95 | 102.2728995 | 93.63855459 | 0.915575436 | 0.519427018 | unchanged |
| hsa_circ_102780 | 129 | 114 | 127 | 121 | 105 | 113 | 123.3027837 | 112.9188147 | 0.915784797 | 0.18229745 | unchanged |
| hsa_circ_102781 | 109 | 77 | 87 | 106 | 91 | 89 | 91.0073339 | 95.53826288 | 1.049786416 | 0.698428872 | unchanged |
| hsa_circ_102782 | 87500 | 88499 | 74258 | 84444 | 92948 | 92948 | 83419.32125 | 90113.16743 | 1.080243355 | 0.28244838 | unchanged |
| hsa_circ_102783 | 52 | 127 | 46 | 51 | 53 | 53 | 75.21156522 | 52.59897463 | 0.699346895 | 0.435306056 | unchanged |
| hsa_circ_102786 | 106 | 90 | 98 | 98 | 102 | 97 | 97.8201999 | 99.32059864 | 1.015338332 | 0.778136613 | unchanged |
| hsa_circ_102787 | 111 | 173 | 91 | 116 | 83 | 77 | 124.9411834 | 92.11176475 | 0.737241014 | 0.297745575 | unchanged |
| hsa_circ_102788 | 284 | 189 | 162 | 257 | 165 | 169 | 211.7249303 | 196.947417 | 0.930204189 | 0.77314462 | unchanged |
| hsa_circ_102790 | 66 | 79 | 63 | 59 | 56 | 65 | 68.99092889 | 59.82639286 | 0.867163174 | 0.183846478 | unchanged |
| hsa_circ_102791 | 63 | 50 | 219 | 65 | 51 | 46 | 110.8374657 | 54.15617575 | 0.488608932 | 0.358485021 | unchanged |
| hsa_circ_102792 | 66 | 59 | 63 | 65 | 71 | 69 | 62.83575123 | 68.6295511 | 1.092205468 | 0.110717796 | unchanged |
| hsa_circ_102793 | 93 | 68 | 85 | 78 | 84 | 84 | 81.72278795 | 81.94767884 | 1.002751875 | 0.977586294 | unchanged |
| hsa_circ_102794 | 90 | 84 | 127 | 97 | 108 | 115 | 100.4223955 | 106.557147 | 1.061089476 | 0.692172613 | unchanged |
| hsa_circ_102795 | 174 | 208 | 500 | 157 | 182 | 184 | 293.965338 | 174.2433106 | 0.592734204 | 0.313052108 | unchanged |
| hsa_circ_102796 | 1532 | 1848 | 1417 | 1681 | 1795 | 1737 | 1599.108368 | 1737.467362 | 1.086522588 | 0.357511814 | unchanged |
| hsa_circ_102797 | 66 | 67 | 71 | 60 | 63 | 66 | 67.95912799 | 62.67775083 | 0.922285978 | 0.075502194 | unchanged |
| hsa_circ_102798 | 586 | 358 | 347 | 542 | 354 | 342 | 430.5121782 | 412.5807089 | 0.95834852 | 0.867982308 | unchanged |
| hsa_circ_102799 | 650 | 553 | 314 | 798 | 531 | 561 | 505.3091342 | 629.8903223 | 1.2465445 | 0.394353508 | unchanged |
| hsa_circ_102800 | 92 | 67 | 89 | 88 | 118 | 109 | 82.55240687 | 104.9421067 | 1.271218014 | 0.137454796 | unchanged |
| hsa_circ_102801 | 107 | 84 | 116 | 122 | 118 | 124 | 102.2225567 | 121.2939829 | 1.186567689 | 0.116601059 | unchanged |
| hsa_circ_102802 | 194 | 179 | 170 | 203 | 182 | 203 | 181.0544099 | 195.7417724 | 1.081121264 | 0.215682968 | unchanged |
| hsa_circ_102803 | 71 | 90 | 66 | 68 | 67 | 69 | 75.83954091 | 67.95166698 | 0.895992594 | 0.345362877 | unchanged |
| hsa_circ_102804 | 860 | 1015 | 1225 | 968 | 1342 | 1310 | 1033.425334 | 1206.498982 | 1.167475717 | 0.339807846 | unchanged |
| hsa_circ_102805 | 482 | 677 | 599 | 558 | 713 | 801 | 585.8136669 | 690.5493686 | 1.178786716 | 0.312924117 | unchanged |
| hsa_circ_102806 | 474 | 841 | 903 | 583 | 733 | 736 | 739.5426476 | 683.6797139 | 0.924462864 | 0.716263623 | unchanged |
| hsa_circ_102807 | 758 | 516 | 482 | 825 | 572 | 589 | 585.3449485 | 661.8410252 | 1.130685465 | 0.556808554 | unchanged |
| hsa_circ_102808 | 131 | 90 | 248 | 128 | 107 | 100 | 156.2316761 | 111.4254904 | 0.713206779 | 0.402500523 | unchanged |
| hsa_circ_102809 | 132 | 111 | 319 | 141 | 192 | 202 | 187.3721282 | 178.1336487 | 0.950694484 | 0.899393777 | unchanged |
| hsa_circ_102810 | 1952 | 1024 | 1133 | 1265 | 1054 | 1027 | 1369.807781 | 1115.22265 | 0.814145361 | 0.447370149 | unchanged |
| hsa_circ_102812 | 352 | 328 | 369 | 362 | 368 | 379 | 349.4179265 | 369.3802214 | 1.057130139 | 0.20040261 | unchanged |
| hsa_circ_102813 | 59 | 52 | 65 | 62 | 72 | 73 | 58.61991982 | 68.79873531 | 1.173640898 | 0.116231947 | unchanged |
| hsa_circ_102815 | 88 | 78 | 71 | 96 | 81 | 76 | 78.79097437 | 84.32252735 | 1.070205414 | 0.508481687 | unchanged |
| hsa_circ_102816 | 227 | 253 | 216 | 261 | 232 | 240 | 231.8076332 | 244.4172683 | 1.05439698 | 0.415674511 | unchanged |
| hsa_circ_102817 | 812 | 864 | 621 | 665 | 680 | 646 | 765.7196205 | 663.4636985 | 0.866457749 | 0.24204812 | unchanged |
| hsa_circ_102818 | 55 | 96 | 127 | 52 | 74 | 76 | 92.39345475 | 67.49873381 | 0.730557527 | 0.325347648 | unchanged |
| hsa_circ_102820 | 214 | 215 | 271 | 238 | 249 | 248 | 233.164948 | 244.9250901 | 1.050437007 | 0.572242294 | unchanged |
| hsa_circ_102821 | 326 | 213 | 240 | 248 | 193 | 185 | 259.7031173 | 208.5107112 | 0.802881049 | 0.264653485 | unchanged |
| hsa_circ_102822 | 78 | 57 | 86 | 68 | 95 | 90 | 73.6243922 | 84.05675908 | 1.141697154 | 0.436088421 | unchanged |
| hsa_circ_102823 | 71 | 61 | 62 | 67 | 80 | 75 | 64.55030445 | 73.98444852 | 1.146151814 | 0.127012906 | unchanged |
| hsa_circ_102824 | 699 | 949 | 917 | 968 | 1403 | 1364 | 854.6684805 | 1245.161141 | 1.456893719 | 0.070663766 | unchanged |
| hsa_circ_102825 | 94 | 78 | 112 | 107 | 147 | 144 | 94.76010712 | 132.6405793 | 1.39975126 | 0.078200697 | unchanged |
| hsa_circ_102826 | 109 | 107 | 105 | 107 | 107 | 108 | 107.002513 | 107.4953002 | 1.00460538 | 0.701792006 | unchanged |
| hsa_circ_102827 | 449 | 438 | 341 | 545 | 490 | 461 | 409.4154766 | 498.4911945 | 1.217568028 | 0.102594916 | unchanged |
| hsa_circ_102828 | 259 | 151 | 166 | 207 | 131 | 119 | 192.220843 | 152.0377401 | 0.790953456 | 0.408160653 | unchanged |
| hsa_circ_102829 | 163 | 192 | 229 | 196 | 233 | 226 | 194.9263024 | 218.4115035 | 1.120482463 | 0.351275473 | unchanged |
| hsa_circ_102831 | 137 | 144 | 204 | 149 | 185 | 178 | 161.4456001 | 170.5925108 | 1.056656302 | 0.722023066 | unchanged |
| hsa_circ_102832 | 104 | 97 | 487 | 109 | 127 | 122 | 229.3000607 | 119.3956695 | 0.520696196 | 0.442021865 | unchanged |
| hsa_circ_102834 | 117 | 98 | 95 | 109 | 104 | 96 | 103.343286 | 103.2682297 | 0.999273719 | 0.992850721 | unchanged |
| hsa_circ_102835 | 73 | 104 | 92 | 78 | 95 | 95 | 89.84543897 | 89.29011578 | 0.993819128 | 0.961022068 | unchanged |
| hsa_circ_102836 | 107 | 124 | 110 | 103 | 103 | 106 | 113.4643282 | 104.058116 | 0.91709983 | 0.154208883 | unchanged |
| hsa_circ_102837 | 85 | 145 | 75 | 81 | 76 | 71 | 101.5561523 | 75.80924962 | 0.74647619 | 0.305246664 | unchanged |
| hsa_circ_102838 | 1923 | 1074 | 1321 | 1033 | 656 | 629 | 1439.346465 | 772.60184 | 0.536772667 | 0.07855504 | unchanged |
| hsa_circ_102839 | 557 | 531 | 397 | 693 | 545 | 643 | 495.2474276 | 626.9741679 | 1.265981675 | 0.116326272 | unchanged |
| hsa_circ_102840 | 124 | 181 | 143 | 131 | 154 | 147 | 149.2452809 | 144.0331853 | 0.965076982 | 0.787318857 | unchanged |
| hsa_circ_102841 | 63 | 61 | 70 | 65 | 77 | 79 | 64.50151873 | 73.75056171 | 1.143392639 | 0.156344287 | unchanged |
| hsa_circ_102842 | 72 | 90 | 95 | 74 | 90 | 88 | 85.77363587 | 83.93271932 | 0.978537501 | 0.846105319 | unchanged |
| hsa_circ_102843 | 481 | 514 | 695 | 500 | 459 | 444 | 563.3170586 | 467.5093116 | 0.829922163 | 0.235757541 | unchanged |
| hsa_circ_102844 | 14069 | 16557 | 18753 | 14008 | 16110 | 15948 | 16459.77154 | 15355.37424 | 0.932903243 | 0.505748367 | unchanged |
| hsa_circ_102845 | 226 | 151 | 256 | 204 | 239 | 285 | 210.9726 | 242.6370954 | 1.150088189 | 0.463194548 | unchanged |
| hsa_circ_102848 | 68 | 63 | 73 | 64 | 67 | 69 | 68.0338661 | 66.53179867 | 0.977921769 | 0.663279688 | unchanged |
| hsa_circ_102849 | 104 | 76 | 121 | 98 | 121 | 121 | 100.2614087 | 113.1798514 | 1.128847608 | 0.437841372 | unchanged |
| hsa_circ_102850 | 141 | 123 | 138 | 123 | 126 | 139 | 134.1435062 | 129.3678777 | 0.964399108 | 0.550951961 | unchanged |
| hsa_circ_102851 | 137 | 121 | 101 | 152 | 148 | 143 | 119.4664093 | 147.846813 | 1.237559695 | 0.055797498 | unchanged |
| hsa_circ_102852 | 121 | 77 | 99 | 113 | 116 | 103 | 99.01308413 | 110.46071 | 1.115617304 | 0.436249078 | unchanged |
| hsa_circ_102854 | 72 | 64 | 75 | 69 | 77 | 69 | 70.13865555 | 71.55971969 | 1.020260784 | 0.754296916 | unchanged |
| hsa_circ_102856 | 71 | 87 | 75 | 75 | 73 | 75 | 77.67310116 | 74.37660772 | 0.957559395 | 0.525850493 | unchanged |
| hsa_circ_102858 | 138 | 150 | 152 | 129 | 204 | 202 | 146.7639375 | 178.2496688 | 1.214533161 | 0.277223677 | unchanged |
| hsa_circ_102859 | 74 | 77 | 96 | 85 | 93 | 93 | 82.20090093 | 89.91024531 | 1.093786617 | 0.346420186 | unchanged |
| hsa_circ_102860 | 83 | 121 | 91 | 85 | 91 | 96 | 98.59725911 | 90.75637056 | 0.920475593 | 0.545784535 | unchanged |
| hsa_circ_102861 | 338 | 462 | 532 | 347 | 710 | 703 | 443.9422194 | 586.5456995 | 1.321220812 | 0.343052114 | unchanged |
| hsa_circ_102863 | 706 | 880 | 598 | 745 | 680 | 771 | 728.2361838 | 732.2442035 | 1.005503736 | 0.965303031 | unchanged |
| hsa_circ_102864 | 125 | 80 | 298 | 115 | 144 | 137 | 167.6918508 | 131.9059431 | 0.786597217 | 0.621346798 | unchanged |
| hsa_circ_102866 | 110 | 101 | 88 | 114 | 90 | 96 | 99.72612451 | 100.1375987 | 1.004126042 | 0.968005046 | unchanged |
| hsa_circ_102867 | 123 | 108 | 121 | 141 | 137 | 183 | 117.5185257 | 153.5712644 | 1.30678345 | 0.080840264 | unchanged |
| hsa_circ_102870 | 71 | 98 | 72 | 78 | 84 | 94 | 80.21256278 | 85.17266929 | 1.061837028 | 0.642003564 | unchanged |
| hsa_circ_102871 | 432 | 408 | 478 | 413 | 478 | 527 | 439.1666081 | 472.5284627 | 1.075966283 | 0.439902823 | unchanged |
| hsa_circ_102872 | 239 | 92 | 142 | 143 | 73 | 78 | 157.7387294 | 98.15239273 | 0.62224663 | 0.289711119 | unchanged |
| hsa_circ_102873 | 123 | 75 | 204 | 119 | 69 | 71 | 133.8485792 | 86.21650833 | 0.644134655 | 0.310933353 | unchanged |
| hsa_circ_102874 | 433 | 385 | 409 | 436 | 526 | 449 | 409.0098175 | 470.195584 | 1.14959486 | 0.121468043 | unchanged |
| hsa_circ_102878 | 63 | 97 | 54 | 68 | 59 | 60 | 71.16015648 | 62.29966866 | 0.875485268 | 0.544898273 | unchanged |
| hsa_circ_102879 | 1238 | 236 | 132 | 1047 | 144 | 153 | 535.4618665 | 448.1270842 | 0.836898222 | 0.859413965 | unchanged |
| hsa_circ_102880 | 57 | 70 | 48 | 55 | 46 | 50 | 58.41983052 | 50.24301918 | 0.860033635 | 0.292877175 | unchanged |
| hsa_circ_102881 | 587 | 506 | 435 | 555 | 302 | 295 | 509.3716602 | 383.967681 | 0.753806525 | 0.262027922 | unchanged |
| hsa_circ_102882 | 101 | 113 | 85 | 84 | 87 | 84 | 99.90510113 | 84.99127028 | 0.850720026 | 0.146159092 | unchanged |
| hsa_circ_102885 | 825 | 402 | 367 | 357 | 319 | 367 | 531.5110953 | 347.8138592 | 0.654386827 | 0.282132673 | unchanged |
| hsa_circ_102886 | 93 | 105 | 102 | 66 | 104 | 106 | 100.2773754 | 92.18712059 | 0.919321235 | 0.57784962 | unchanged |
| hsa_circ_102888 | 487 | 165 | 277 | 192 | 362 | 302 | 309.8723258 | 284.9580286 | 0.919598186 | 0.826615183 | unchanged |
| hsa_circ_102889 | 40701 | 36821 | 34324 | 44430 | 37018 | 40291 | 37282.10661 | 40579.65227 | 1.08844848 | 0.309538315 | unchanged |
| hsa_circ_102890 | 84650 | 82811 | 67253 | 85879 | 78848 | 77958 | 78238.1178 | 80894.8918 | 1.03395754 | 0.683736668 | unchanged |
| hsa_circ_102891 | 84 | 79 | 57 | 81 | 54 | 50 | 73.48731702 | 61.63962043 | 0.838779029 | 0.408205504 | unchanged |
| hsa_circ_102893 | 110 | 136 | 124 | 109 | 122 | 125 | 123.4596162 | 118.9598627 | 0.963552831 | 0.647419477 | unchanged |
| hsa_circ_102894 | 112 | 159 | 98 | 97 | 88 | 87 | 123.1578069 | 90.77623997 | 0.737072559 | 0.158617177 | unchanged |
| hsa_circ_102895 | 609 | 555 | 454 | 524 | 451 | 480 | 539.2522522 | 484.8381408 | 0.8990934 | 0.338793822 | unchanged |
| hsa_circ_102896 | 133 | 90 | 112 | 147 | 99 | 114 | 111.622499 | 119.9334873 | 1.07445621 | 0.684538532 | unchanged |
| hsa_circ_102897 | 108 | 111 | 107 | 112 | 114 | 110 | 108.6023091 | 112.1230578 | 1.032418728 | 0.107998443 | unchanged |
| hsa_circ_102898 | 107 | 67 | 85 | 109 | 93 | 92 | 86.53412014 | 98.06087076 | 1.13320469 | 0.42068051 | unchanged |
| hsa_circ_102899 | 282 | 251 | 227 | 286 | 256 | 257 | 253.2890768 | 266.6263995 | 1.052656525 | 0.516708014 | unchanged |
| hsa_circ_102901 | 101 | 85 | 88 | 96 | 91 | 92 | 91.11594055 | 92.88060882 | 1.019367284 | 0.741793826 | unchanged |
| hsa_circ_102903 | 68 | 61 | 56 | 65 | 69 | 64 | 61.65430872 | 66.03917655 | 1.071120217 | 0.316353437 | unchanged |
| hsa_circ_102904 | 349 | 158 | 155 | 286 | 175 | 177 | 220.9327719 | 212.9085441 | 0.963680228 | 0.918651616 | unchanged |
| hsa_circ_102905 | 62 | 72 | 55 | 59 | 63 | 62 | 62.96720176 | 61.45443787 | 0.975975367 | 0.778086006 | unchanged |
| hsa_circ_102906 | 80 | 65 | 72 | 76 | 93 | 95 | 72.21728883 | 88.15235364 | 1.220654431 | 0.090338671 | unchanged |
| hsa_circ_102907 | 107 | 97 | 93 | 113 | 109 | 120 | 99.18170184 | 113.6612051 | 1.145989663 | 0.051993104 | unchanged |
| hsa_circ_102908 | 220 | 140 | 84 | 228 | 98 | 101 | 148.0017313 | 142.1159108 | 0.960231408 | 0.924415039 | unchanged |
| hsa_circ_102911 | 78 | 59 | 65 | 70 | 86 | 99 | 67.44863288 | 84.9921988 | 1.260102617 | 0.157817824 | unchanged |
| hsa_circ_102912 | 1120 | 725 | 1032 | 769 | 551 | 582 | 959.010408 | 633.9616312 | 0.661058134 | 0.077838377 | unchanged |
| hsa_circ_102914 | 622 | 947 | 767 | 725 | 1063 | 1048 | 778.6031191 | 945.0077536 | 1.213722024 | 0.31480248 | unchanged |
| hsa_circ_102915 | 170 | 90 | 146 | 145 | 193 | 192 | 135.1092782 | 176.7604238 | 1.308277464 | 0.218035487 | unchanged |
| hsa_circ_102916 | 1065 | 1864 | 1507 | 1319 | 1902 | 1832 | 1478.809786 | 1684.309213 | 1.138962718 | 0.524782586 | unchanged |
| hsa_circ_102917 | 507 | 503 | 344 | 493 | 390 | 386 | 451.4709867 | 423.019126 | 0.936979648 | 0.680601493 | unchanged |
| hsa_circ_102918 | 609 | 412 | 311 | 549 | 361 | 332 | 443.8364763 | 414.2954471 | 0.933441637 | 0.803065342 | unchanged |
| hsa_circ_102919 | 676 | 749 | 485 | 540 | 348 | 348 | 636.9246383 | 412.1923263 | 0.647160278 | 0.091247174 | unchanged |
| hsa_circ_102920 | 141 | 186 | 160 | 157 | 198 | 199 | 162.4796679 | 184.5128111 | 1.13560554 | 0.312197303 | unchanged |
| hsa_circ_102922 | 624 | 304 | 273 | 408 | 278 | 270 | 400.3932938 | 318.9691417 | 0.796639571 | 0.537682271 | unchanged |
| hsa_circ_102924 | 1414 | 676 | 638 | 968 | 528 | 518 | 909.6267921 | 671.6588889 | 0.738389518 | 0.462210282 | unchanged |
| hsa_circ_102926 | 95 | 62 | 60 | 83 | 58 | 59 | 72.53852431 | 66.83450039 | 0.921365592 | 0.706589451 | unchanged |
| hsa_circ_102927 | 360 | 476 | 549 | 508 | 793 | 856 | 461.7108185 | 718.9031348 | 1.557041997 | 0.098984939 | unchanged |
| hsa_circ_102928 | 809 | 742 | 765 | 932 | 605 | 617 | 771.7868343 | 717.9439354 | 0.930236049 | 0.646745367 | unchanged |
| hsa_circ_102929 | 836 | 998 | 965 | 1024 | 1055 | 1076 | 932.7745918 | 1051.274301 | 1.127040027 | 0.083807766 | unchanged |
| hsa_circ_102930 | 74 | 67 | 68 | 69 | 75 | 73 | 69.66460471 | 72.18479999 | 1.036176123 | 0.454005279 | unchanged |
| hsa_circ_102931 | 59 | 92 | 62 | 81 | 58 | 61 | 70.7759217 | 66.66204527 | 0.941874633 | 0.766512034 | unchanged |
| hsa_circ_102932 | 79 | 69 | 93 | 82 | 96 | 93 | 80.18632486 | 90.19553934 | 1.124824457 | 0.289630599 | unchanged |
| hsa_circ_102933 | 79 | 113 | 128 | 76 | 102 | 117 | 106.8222104 | 98.45351423 | 0.921657714 | 0.678302246 | unchanged |
| hsa_circ_102934 | 136 | 168 | 143 | 124 | 141 | 134 | 149.0234474 | 133.1874756 | 0.893735033 | 0.212303412 | unchanged |
| hsa_circ_102936 | 307 | 193 | 198 | 295 | 212 | 232 | 232.8426822 | 246.3096783 | 1.057837317 | 0.778089742 | unchanged |
| hsa_circ_102937 | 108 | 89 | 98 | 99 | 95 | 93 | 98.58993256 | 95.59975905 | 0.9696706 | 0.628075105 | unchanged |
| hsa_circ_102938 | 119 | 126 | 93 | 121 | 102 | 94 | 112.7837951 | 105.3761452 | 0.934319909 | 0.596576145 | unchanged |
| hsa_circ_102939 | 90 | 117 | 115 | 79 | 115 | 115 | 107.2229817 | 103.1478828 | 0.961994166 | 0.795342677 | unchanged |
| hsa_circ_102943 | 144 | 145 | 110 | 115 | 102 | 111 | 133.2301628 | 109.3476989 | 0.820742815 | 0.119238067 | unchanged |
| hsa_circ_102945 | 638 | 415 | 338 | 487 | 174 | 172 | 463.6976056 | 277.6000602 | 0.59866615 | 0.248568898 | unchanged |
| hsa_circ_102948 | 488 | 616 | 538 | 585 | 679 | 791 | 547.0995328 | 684.6320215 | 1.251384767 | 0.121933913 | unchanged |
| hsa_circ_102949 | 348 | 390 | 341 | 325 | 319 | 297 | 359.5719465 | 313.4201835 | 0.871648043 | 0.058517533 | unchanged |
| hsa_circ_102950 | 434 | 464 | 360 | 367 | 272 | 278 | 419.3573012 | 305.7331172 | 0.729051614 | 0.059427658 | unchanged |
| hsa_circ_102951 | 1604 | 1460 | 1062 | 1590 | 1529 | 1488 | 1375.380403 | 1535.7438 | 1.116595668 | 0.385237752 | unchanged |
| hsa_circ_102952 | 240 | 156 | 148 | 192 | 161 | 161 | 181.3592199 | 171.3236037 | 0.944664428 | 0.764136792 | unchanged |
| hsa_circ_102953 | 86 | 87 | 100 | 89 | 100 | 102 | 91.23640637 | 96.79317585 | 1.060905177 | 0.407639668 | unchanged |
| hsa_circ_102954 | 2196 | 2065 | 1542 | 2421 | 1452 | 1905 | 1934.409195 | 1925.906473 | 0.995604486 | 0.981451162 | unchanged |
| hsa_circ_102955 | 661 | 1291 | 1150 | 667 | 1010 | 1076 | 1033.935559 | 917.8278105 | 0.88770311 | 0.638951782 | unchanged |
| hsa_circ_102956 | 392 | 305 | 269 | 387 | 270 | 283 | 321.8959186 | 313.5786267 | 0.974161549 | 0.880688072 | unchanged |
| hsa_circ_102957 | 114 | 110 | 138 | 112 | 183 | 177 | 120.7307337 | 157.3779105 | 1.30354472 | 0.205641096 | unchanged |
| hsa_circ_102958 | 129 | 186 | 169 | 137 | 170 | 163 | 161.2201569 | 156.7467133 | 0.972252579 | 0.831623575 | unchanged |
| hsa_circ_102959 | 233 | 272 | 174 | 189 | 145 | 147 | 226.1714437 | 160.4999003 | 0.709638218 | 0.109138949 | unchanged |
| hsa_circ_102960 | 77 | 64 | 83 | 87 | 94 | 95 | 74.5020279 | 91.6612809 | 1.230319274 | 0.052114009 | unchanged |
| hsa_circ_102961 | 331 | 464 | 599 | 359 | 499 | 547 | 464.6042938 | 468.1158776 | 1.007558225 | 0.972508566 | unchanged |
| hsa_circ_102962 | 155 | 84 | 94 | 93 | 73 | 71 | 110.6224581 | 78.92075027 | 0.713424305 | 0.244725063 | unchanged |
| hsa_circ_102964 | 99 | 104 | 206 | 101 | 115 | 112 | 136.1784319 | 109.3194562 | 0.802766302 | 0.485564655 | unchanged |
| hsa_circ_102966 | 271 | 609 | 455 | 304 | 427 | 443 | 445.2283049 | 391.1084692 | 0.878444755 | 0.640116534 | unchanged |
| hsa_circ_102967 | 70 | 83 | 46 | 73 | 52 | 49 | 66.37342284 | 57.90597993 | 0.872427207 | 0.554657314 | unchanged |
| hsa_circ_102968 | 78 | 66 | 59 | 73 | 65 | 63 | 67.88838468 | 66.95601609 | 0.986266154 | 0.890945845 | unchanged |
| hsa_circ_102969 | 205 | 321 | 247 | 279 | 213 | 208 | 257.2436859 | 233.3026616 | 0.906932509 | 0.589437238 | unchanged |
| hsa_circ_102970 | 88 | 66 | 70 | 69 | 69 | 75 | 74.59797446 | 70.83183102 | 0.949514133 | 0.625762352 | unchanged |
| hsa_circ_102971 | 225 | 161 | 166 | 203 | 214 | 212 | 184.0477814 | 209.83774 | 1.14012643 | 0.286895319 | unchanged |
| hsa_circ_102972 | 10415 | 10339 | 8980 | 8142 | 10296 | 10065 | 9911.566309 | 9501.101207 | 0.958587262 | 0.645536303 | unchanged |
| hsa_circ_102973 | 63 | 85 | 63 | 58 | 72 | 73 | 70.48201386 | 67.64635438 | 0.959767616 | 0.765697449 | unchanged |
| hsa_circ_102974 | 177 | 141 | 155 | 161 | 152 | 149 | 157.7907172 | 154.0827977 | 0.976501029 | 0.75516534 | unchanged |
| hsa_circ_102975 | 229 | 424 | 331 | 236 | 281 | 304 | 327.8834385 | 273.4455873 | 0.83397194 | 0.413717154 | unchanged |
| hsa_circ_102976 | 314 | 373 | 199 | 254 | 186 | 176 | 295.5430262 | 205.5704347 | 0.695568552 | 0.189040731 | unchanged |
| hsa_circ_102977 | 172 | 238 | 117 | 141 | 117 | 114 | 175.7225983 | 124.0619776 | 0.706010375 | 0.223684701 | unchanged |
| hsa_circ_102978 | 765 | 471 | 451 | 798 | 289 | 314 | 562.1283262 | 466.8501603 | 0.830504599 | 0.649465547 | unchanged |
| hsa_circ_102979 | 638 | 401 | 385 | 602 | 262 | 261 | 474.877223 | 374.9040065 | 0.789475655 | 0.514573062 | unchanged |
| hsa_circ_102980 | 697 | 882 | 807 | 731 | 938 | 945 | 795.3284274 | 871.0392729 | 1.095194442 | 0.439982782 | unchanged |
| hsa_circ_102981 | 273 | 179 | 145 | 248 | 139 | 146 | 199.0431046 | 177.2730873 | 0.890626619 | 0.696562866 | unchanged |
| hsa_circ_102983 | 11177 | 7977 | 6229 | 12127 | 7100 | 6564 | 8461.23063 | 8596.925724 | 1.016037276 | 0.955565096 | unchanged |
| hsa_circ_102984 | 10930 | 8585 | 6748 | 11569 | 7115 | 6873 | 8754.664263 | 8518.762909 | 0.973054209 | 0.909457228 | unchanged |
| hsa_circ_102985 | 67 | 71 | 69 | 49 | 68 | 64 | 69.21433151 | 60.27770505 | 0.870884739 | 0.193474244 | unchanged |
| hsa_circ_102986 | 78 | 106 | 83 | 79 | 88 | 87 | 88.96518741 | 84.90533444 | 0.954365824 | 0.677916872 | unchanged |
| hsa_circ_102987 | 82 | 73 | 223 | 84 | 87 | 85 | 126.1552402 | 85.22056512 | 0.675521405 | 0.444874234 | unchanged |
| hsa_circ_102989 | 130 | 112 | 176 | 137 | 222 | 209 | 139.6301084 | 189.481253 | 1.357022888 | 0.200289149 | unchanged |
| hsa_circ_102991 | 1011 | 2171 | 1750 | 757 | 1266 | 1359 | 1644.119553 | 1127.140616 | 0.685558793 | 0.252734911 | unchanged |
| hsa_circ_102993 | 1530 | 601 | 458 | 1373 | 703 | 771 | 863.1458138 | 949.0108746 | 1.099479207 | 0.839694998 | unchanged |
| hsa_circ_102995 | 136 | 87 | 144 | 127 | 135 | 132 | 122.3201348 | 131.6564071 | 1.076326537 | 0.628565935 | unchanged |
| hsa_circ_102999 | 473 | 234 | 420 | 366 | 256 | 272 | 375.538913 | 298.2289993 | 0.794136077 | 0.389378711 | unchanged |
| hsa_circ_103000 | 371 | 643 | 540 | 587 | 610 | 640 | 518.0041085 | 612.177746 | 1.181800947 | 0.308176064 | unchanged |
| hsa_circ_103001 | 232 | 111 | 175 | 168 | 152 | 155 | 172.7224295 | 158.4448101 | 0.91733778 | 0.705136304 | unchanged |
| hsa_circ_103002 | 849 | 425 | 436 | 650 | 414 | 400 | 569.9225404 | 487.9204892 | 0.856117199 | 0.638391382 | unchanged |
| hsa_circ_103003 | 226 | 108 | 137 | 205 | 115 | 112 | 157.0145346 | 143.9855346 | 0.917020421 | 0.793914803 | unchanged |
| hsa_circ_103004 | 116 | 169 | 125 | 118 | 103 | 104 | 136.9144737 | 108.3069667 | 0.791055641 | 0.169681701 | unchanged |
| hsa_circ_103005 | 65 | 76 | 68 | 72 | 61 | 62 | 69.66560345 | 65.07430599 | 0.934095203 | 0.391194171 | unchanged |
| hsa_circ_103006 | 91 | 78 | 59 | 94 | 54 | 55 | 76.16733228 | 67.57516257 | 0.887193506 | 0.618696954 | unchanged |
| hsa_circ_103007 | 117 | 118 | 92 | 89 | 91 | 86 | 108.8316608 | 88.68149948 | 0.81485019 | 0.082513846 | unchanged |
| hsa_circ_103008 | 96 | 80 | 107 | 102 | 120 | 106 | 94.26379925 | 109.4089345 | 1.160667567 | 0.19737773 | unchanged |
| hsa_circ_103010 | 295 | 466 | 369 | 320 | 388 | 390 | 376.6645408 | 365.9534123 | 0.971563215 | 0.853576282 | unchanged |
| hsa_circ_103011 | 156 | 148 | 88 | 141 | 105 | 109 | 130.6356045 | 118.4746686 | 0.906909483 | 0.644801006 | unchanged |
| hsa_circ_103012 | 962 | 1078 | 953 | 1054 | 1230 | 1255 | 997.4584994 | 1179.576599 | 1.182582132 | 0.072039867 | unchanged |
| hsa_circ_103013 | 246 | 162 | 206 | 184 | 194 | 177 | 204.7282946 | 185.0501398 | 0.903881607 | 0.471884794 | unchanged |
| hsa_circ_103014 | 2518 | 2232 | 2086 | 2476 | 2587 | 2577 | 2278.495885 | 2546.661082 | 1.117693957 | 0.111683797 | unchanged |
| hsa_circ_103015 | 110 | 111 | 103 | 120 | 87 | 104 | 108.0301482 | 103.7601582 | 0.960474089 | 0.690178665 | unchanged |
| hsa_circ_103017 | 258 | 301 | 311 | 270 | 306 | 317 | 290.0597396 | 297.8044322 | 1.026700336 | 0.736263078 | unchanged |
| hsa_circ_103018 | 1932 | 3214 | 3004 | 1890 | 2963 | 2931 | 2716.871105 | 2594.57069 | 0.95498483 | 0.829159676 | unchanged |
| hsa_circ_103019 | 397 | 505 | 434 | 448 | 553 | 600 | 445.2120537 | 533.4374776 | 1.198164949 | 0.185144945 | unchanged |
| hsa_circ_103020 | 70 | 210 | 225 | 75 | 69 | 71 | 168.1841285 | 71.87839035 | 0.427379153 | 0.122275852 | unchanged |
| hsa_circ_103021 | 123 | 186 | 306 | 175 | 130 | 122 | 204.9256624 | 142.1583683 | 0.693707009 | 0.32509052 | unchanged |
| hsa_circ_103023 | 1106 | 1860 | 2081 | 1351 | 2412 | 2371 | 1682.214515 | 2044.66168 | 1.21545835 | 0.470732763 | unchanged |
| hsa_circ_103025 | 284 | 432 | 458 | 305 | 421 | 452 | 391.1608751 | 392.6157681 | 1.003719424 | 0.984484414 | unchanged |
| hsa_circ_103026 | 120 | 161 | 279 | 126 | 118 | 120 | 186.5014068 | 121.4099272 | 0.650986656 | 0.244256221 | unchanged |
| hsa_circ_103027 | 122 | 130 | 121 | 132 | 135 | 125 | 124.283276 | 130.764165 | 1.052146106 | 0.206649542 | unchanged |
| hsa_circ_103029 | 117 | 105 | 95 | 114 | 123 | 124 | 105.6915044 | 120.1674626 | 1.136964256 | 0.108155919 | unchanged |
| hsa_circ_103030 | 70 | 117 | 87 | 80 | 83 | 95 | 91.63837246 | 85.97211288 | 0.938167174 | 0.716812124 | unchanged |
| hsa_circ_103031 | 90 | 89 | 106 | 90 | 112 | 111 | 94.79253415 | 104.2919039 | 1.100212215 | 0.358839246 | unchanged |
| hsa_circ_103032 | 245 | 218 | 290 | 265 | 313 | 347 | 251.1337504 | 308.2162279 | 1.227299108 | 0.147582054 | unchanged |
| hsa_circ_103033 | 228 | 215 | 177 | 173 | 141 | 159 | 206.7836579 | 157.7328162 | 0.762791498 | 0.051727095 | unchanged |
| hsa_circ_103034 | 176 | 212 | 181 | 236 | 194 | 194 | 189.4897058 | 208.1066133 | 1.098247593 | 0.356449225 | unchanged |
| hsa_circ_103035 | 135 | 185 | 224 | 157 | 240 | 246 | 181.5249928 | 214.0296913 | 1.17906459 | 0.446560796 | unchanged |
| hsa_circ_103036 | 1287 | 2583 | 2424 | 1117 | 1925 | 1919 | 2098.001961 | 1653.593119 | 0.788175202 | 0.414146044 | unchanged |
| hsa_circ_103037 | 89 | 117 | 108 | 92 | 103 | 107 | 105.043979 | 100.8829001 | 0.960387269 | 0.68288044 | unchanged |
| hsa_circ_103038 | 2616 | 6288 | 5827 | 4461 | 6739 | 6911 | 4910.778002 | 6036.889635 | 1.229314303 | 0.465930317 | unchanged |
| hsa_circ_103039 | 366 | 476 | 424 | 399 | 468 | 462 | 422.0812021 | 442.935344 | 1.049407891 | 0.61756313 | unchanged |
| hsa_circ_103041 | 528 | 658 | 589 | 596 | 722 | 724 | 591.3618073 | 680.7432181 | 1.151145052 | 0.189554174 | unchanged |
| hsa_circ_103042 | 112 | 112 | 126 | 103 | 152 | 157 | 116.442479 | 137.1030089 | 1.177431209 | 0.310468581 | unchanged |
| hsa_circ_103043 | 68 | 55 | 80 | 60 | 78 | 73 | 67.39367092 | 70.21493922 | 1.041862511 | 0.768918161 | unchanged |
| hsa_circ_103044 | 82 | 77 | 176 | 83 | 83 | 87 | 111.7201558 | 84.65729477 | 0.757762055 | 0.450646801 | unchanged |
| hsa_circ_103045 | 87 | 91 | 100 | 88 | 106 | 108 | 92.65279556 | 100.678024 | 1.08661615 | 0.338087843 | unchanged |
| hsa_circ_103046 | 123 | 91 | 79 | 118 | 82 | 74 | 97.77359552 | 91.34982937 | 0.934299581 | 0.75272048 | unchanged |
| hsa_circ_103048 | 80 | 65 | 83 | 82 | 83 | 78 | 75.69352129 | 81.10920346 | 1.071547499 | 0.403465844 | unchanged |
| hsa_circ_103050 | 324 | 433 | 385 | 329 | 405 | 440 | 380.518976 | 391.6036353 | 1.029130372 | 0.819641249 | unchanged |
| hsa_circ_103051 | 73 | 53 | 80 | 73 | 88 | 78 | 68.92742908 | 79.8650534 | 1.15868319 | 0.308131579 | unchanged |
| hsa_circ_103052 | 600 | 398 | 394 | 588 | 525 | 510 | 464.1235784 | 540.9767052 | 1.165587638 | 0.346971641 | unchanged |
| hsa_circ_103054 | 119 | 69 | 78 | 91 | 79 | 73 | 88.37318199 | 80.95347517 | 0.916041194 | 0.670455974 | unchanged |
| hsa_circ_103056 | 139 | 169 | 158 | 154 | 156 | 170 | 155.4773881 | 159.9305132 | 1.028641625 | 0.684293044 | unchanged |
| hsa_circ_103057 | 61 | 121 | 60 | 62 | 61 | 57 | 80.436396 | 59.88166423 | 0.744459812 | 0.368064639 | unchanged |
| hsa_circ_103059 | 1131 | 1430 | 1424 | 1178 | 1697 | 1625 | 1328.279052 | 1500.045917 | 1.129315346 | 0.417615712 | unchanged |
| hsa_circ_103060 | 2988 | 2687 | 1970 | 2698 | 2259 | 2032 | 2548.460546 | 2329.617114 | 0.914127204 | 0.575695172 | unchanged |
| hsa_circ_103061 | 61 | 77 | 55 | 59 | 58 | 56 | 64.62858978 | 57.62021883 | 0.891559278 | 0.346102092 | unchanged |
| hsa_circ_103064 | 70 | 63 | 62 | 66 | 59 | 56 | 64.82786002 | 60.34362055 | 0.930828513 | 0.307159125 | unchanged |
| hsa_circ_103065 | 18819 | 27305 | 26472 | 19586 | 22397 | 22942 | 24198.54729 | 21641.78074 | 0.894342147 | 0.426829508 | unchanged |
| hsa_circ_103066 | 459 | 448 | 725 | 431 | 442 | 462 | 543.8551117 | 444.9905334 | 0.818215226 | 0.33863138 | unchanged |
| hsa_circ_103067 | 315 | 275 | 253 | 275 | 186 | 167 | 280.939477 | 209.4204114 | 0.745428922 | 0.131760956 | unchanged |
| hsa_circ_103068 | 62 | 107 | 69 | 55 | 62 | 64 | 79.42933393 | 60.33615496 | 0.759620558 | 0.244128096 | unchanged |
| hsa_circ_103069 | 73 | 66 | 87 | 79 | 96 | 100 | 75.22268934 | 91.73720902 | 1.219541734 | 0.136760095 | unchanged |
| hsa_circ_103071 | 69 | 72 | 85 | 76 | 76 | 74 | 75.26077844 | 75.07848962 | 0.997577904 | 0.97245107 | unchanged |
| hsa_circ_103072 | 248 | 210 | 189 | 204 | 166 | 165 | 215.6887226 | 178.3607993 | 0.826936138 | 0.157881693 | unchanged |
| hsa_circ_103073 | 1107 | 1318 | 1463 | 1088 | 1270 | 944 | 1296.145144 | 1100.44552 | 0.849014113 | 0.234057198 | unchanged |
| hsa_circ_103074 | 93 | 52 | 74 | 88 | 93 | 86 | 72.87937353 | 89.03513275 | 1.221678075 | 0.245338559 | unchanged |
| hsa_circ_103075 | 87 | 74 | 146 | 85 | 68 | 71 | 102.470141 | 74.87971976 | 0.730746723 | 0.291320967 | unchanged |
| hsa_circ_103076 | 1400 | 599 | 472 | 1427 | 757 | 882 | 823.8902154 | 1022.338226 | 1.240867056 | 0.606915751 | unchanged |
| hsa_circ_103077 | 108 | 80 | 103 | 110 | 135 | 118 | 97.29049394 | 121.0546726 | 1.244260027 | 0.102165306 | unchanged |
| hsa_circ_103078 | 60 | 62 | 77 | 74 | 87 | 76 | 66.3615102 | 79.03971252 | 1.191047526 | 0.132918069 | unchanged |
| hsa_circ_103079 | 86 | 69 | 111 | 94 | 134 | 112 | 88.4346741 | 113.2668733 | 1.280797091 | 0.216212727 | unchanged |
| hsa_circ_103080 | 364 | 292 | 452 | 403 | 687 | 565 | 369.20725 | 551.7795055 | 1.494498024 | 0.125377493 | unchanged |
| hsa_circ_103081 | 796 | 843 | 1114 | 929 | 1065 | 1029 | 917.5100237 | 1007.569808 | 1.098156731 | 0.448102587 | unchanged |
| hsa_circ_103082 | 83 | 70 | 122 | 85 | 153 | 166 | 91.6952215 | 134.7201197 | 1.469216361 | 0.216519552 | unchanged |
| hsa_circ_103083 | 167 | 169 | 184 | 159 | 180 | 183 | 173.1259315 | 174.0094577 | 1.005103373 | 0.929743182 | unchanged |
| hsa_circ_103084 | 167 | 204 | 145 | 176 | 149 | 156 | 172.1732826 | 160.4459954 | 0.931886719 | 0.568143723 | unchanged |
| hsa_circ_103085 | 741 | 400 | 544 | 615 | 550 | 544 | 561.6347399 | 569.602729 | 1.014187137 | 0.941057421 | unchanged |
| hsa_circ_103086 | 791 | 829 | 754 | 773 | 1108 | 1025 | 791.2862669 | 968.5600468 | 1.224032423 | 0.159787578 | unchanged |
| hsa_circ_103087 | 152 | 121 | 135 | 160 | 163 | 162 | 135.8620498 | 161.5720368 | 1.189235972 | 0.049180829 | unchanged |
| hsa_circ_103088 | 131 | 138 | 330 | 128 | 132 | 136 | 199.6611429 | 131.7859952 | 0.660048286 | 0.357943642 | unchanged |
| hsa_circ_103089 | 1379 | 1782 | 2078 | 1250 | 1796 | 1821 | 1746.197906 | 1622.224815 | 0.929003986 | 0.675641016 | unchanged |
| hsa_circ_103090 | 221 | 208 | 223 | 207 | 208 | 214 | 217.0282959 | 209.5235858 | 0.965420591 | 0.234239985 | unchanged |
| hsa_circ_103091 | 79 | 115 | 64 | 70 | 69 | 65 | 85.6941163 | 67.98928847 | 0.793395059 | 0.307000236 | unchanged |
| hsa_circ_103092 | 75 | 59 | 71 | 60 | 63 | 62 | 68.23434615 | 62.01033631 | 0.908784796 | 0.248600309 | unchanged |
| hsa_circ_103093 | 354 | 248 | 254 | 313 | 302 | 301 | 285.7438494 | 305.3901886 | 1.068755073 | 0.600617772 | unchanged |
| hsa_circ_103094 | 173 | 156 | 173 | 150 | 168 | 160 | 167.1380074 | 159.4732907 | 0.95414139 | 0.377511549 | unchanged |
| hsa_circ_103095 | 115 | 91 | 139 | 119 | 172 | 160 | 115.137655 | 150.2914484 | 1.305319692 | 0.17433213 | unchanged |
| hsa_circ_103096 | 610 | 923 | 1311 | 638 | 1210 | 1197 | 947.8859436 | 1015.035321 | 1.0708412 | 0.820381398 | unchanged |
| hsa_circ_103097 | 132 | 94 | 132 | 109 | 115 | 115 | 119.176755 | 113.2543205 | 0.950305456 | 0.668178737 | unchanged |
| hsa_circ_103098 | 116 | 211 | 133 | 117 | 132 | 136 | 153.3441449 | 128.0924704 | 0.835326778 | 0.443858929 | unchanged |
| hsa_circ_103099 | 69 | 119 | 68 | 74 | 73 | 74 | 85.06365785 | 73.68632349 | 0.866249176 | 0.533777432 | unchanged |
| hsa_circ_103100 | 101 | 71 | 117 | 100 | 122 | 123 | 96.01164694 | 114.8184991 | 1.195880945 | 0.289353687 | unchanged |
| hsa_circ_103101 | 107 | 90 | 108 | 101 | 108 | 97 | 101.6484024 | 102.1452411 | 1.004887817 | 0.944429078 | unchanged |
| hsa_circ_103102 | 141 | 358 | 297 | 235 | 376 | 377 | 265.2367662 | 329.3066861 | 1.241557461 | 0.468684214 | unchanged |
| hsa_circ_103103 | 178 | 291 | 266 | 150 | 232 | 215 | 245.0929262 | 199.0432019 | 0.812113205 | 0.339498562 | unchanged |
| hsa_circ_103104 | 627 | 618 | 435 | 603 | 422 | 437 | 560.2500662 | 487.4778913 | 0.870107691 | 0.442077379 | unchanged |
| hsa_circ_103105 | 98 | 165 | 139 | 103 | 121 | 111 | 133.7984197 | 111.4675742 | 0.833100828 | 0.33259218 | unchanged |
| hsa_circ_103107 | 209 | 155 | 129 | 196 | 103 | 101 | 164.3341072 | 133.4620163 | 0.812138263 | 0.476346371 | unchanged |
| hsa_circ_103108 | 1157 | 115 | 95 | 1549 | 105 | 105 | 455.569503 | 586.2403241 | 1.286829606 | 0.837035001 | unchanged |
| hsa_circ_103111 | 115 | 87 | 164 | 80 | 137 | 86 | 121.745645 | 101.082539 | 0.830276426 | 0.512803374 | unchanged |
| hsa_circ_103112 | 313 | 360 | 412 | 339 | 407 | 416 | 361.4063972 | 387.4091388 | 1.071948759 | 0.524887183 | unchanged |
| hsa_circ_103113 | 141 | 152 | 179 | 126 | 171 | 162 | 157.1668702 | 152.9645236 | 0.973261881 | 0.82441328 | unchanged |
| hsa_circ_103114 | 168 | 191 | 218 | 207 | 309 | 275 | 192.4800195 | 263.6290004 | 1.369643463 | 0.098940646 | unchanged |
| hsa_circ_103119 | 535 | 521 | 501 | 564 | 535 | 498 | 519.2090481 | 532.6409483 | 1.025869927 | 0.564974972 | unchanged |
| hsa_circ_103120 | 257 | 192 | 144 | 176 | 131 | 129 | 197.6982694 | 145.2968374 | 0.734942384 | 0.219648291 | unchanged |
| hsa_circ_103121 | 85 | 84 | 87 | 89 | 94 | 103 | 85.78694908 | 95.70265929 | 1.1155853 | 0.078296276 | unchanged |
| hsa_circ_103122 | 140 | 142 | 189 | 176 | 250 | 238 | 156.9801228 | 221.2396308 | 1.409348056 | 0.082344521 | unchanged |
| hsa_circ_103123 | 204 | 238 | 281 | 225 | 336 | 344 | 240.8314141 | 301.6879743 | 1.252693613 | 0.243312645 | unchanged |
| hsa_circ_103124 | 95 | 101 | 103 | 102 | 99 | 97 | 99.49312601 | 99.31546299 | 0.998214319 | 0.955970792 | unchanged |
| hsa_circ_103125 | 105 | 81 | 89 | 117 | 94 | 91 | 91.628148 | 100.4319152 | 1.096081471 | 0.464167213 | unchanged |
| hsa_circ_103126 | 268 | 184 | 284 | 287 | 382 | 372 | 245.3287721 | 347.1102341 | 1.414877803 | 0.078147922 | unchanged |
| hsa_circ_103127 | 108 | 96 | 113 | 106 | 122 | 123 | 105.7740358 | 117.1276424 | 1.107338313 | 0.200926368 | unchanged |
| hsa_circ_103128 | 78 | 89 | 76 | 64 | 74 | 83 | 80.9506222 | 73.26061066 | 0.905003676 | 0.324176919 | unchanged |
| hsa_circ_103129 | 84 | 83 | 166 | 90 | 92 | 96 | 110.947548 | 92.44855189 | 0.833263588 | 0.536756936 | unchanged |
| hsa_circ_103130 | 165 | 129 | 190 | 130 | 176 | 157 | 161.3145377 | 154.4724999 | 0.957585734 | 0.773107944 | unchanged |
| hsa_circ_103134 | 2838 | 1405 | 1033 | 1729 | 508 | 484 | 1758.772804 | 906.8697611 | 0.515626441 | 0.282714016 | unchanged |
| hsa_circ_103135 | 407 | 264 | 230 | 376 | 243 | 251 | 300.1059178 | 289.7662694 | 0.965546669 | 0.888394989 | unchanged |
| hsa_circ_103136 | 197 | 231 | 182 | 194 | 189 | 196 | 203.292539 | 192.9810569 | 0.949277617 | 0.518071467 | unchanged |
| hsa_circ_103137 | 8534 | 3242 | 4325 | 5935 | 3784 | 3667 | 5366.737584 | 4462.097766 | 0.831435802 | 0.637052662 | unchanged |
| hsa_circ_103138 | 170 | 164 | 224 | 196 | 281 | 276 | 185.9408372 | 251.0284896 | 1.350044957 | 0.125314288 | unchanged |
| hsa_circ_103139 | 342 | 294 | 437 | 373 | 435 | 427 | 357.4665074 | 411.5438824 | 1.151279557 | 0.309630673 | unchanged |
| hsa_circ_103140 | 295 | 163 | 114 | 167 | 92 | 91 | 190.6695043 | 116.6501533 | 0.611792398 | 0.281056124 | unchanged |
| hsa_circ_103141 | 426 | 716 | 634 | 407 | 669 | 713 | 592.0692835 | 596.0236251 | 1.006678849 | 0.976994762 | unchanged |
| hsa_circ_103142 | 66 | 59 | 61 | 58 | 66 | 64 | 62.33492098 | 62.54196927 | 1.003321546 | 0.952544524 | unchanged |
| hsa_circ_103143 | 1103 | 1293 | 926 | 1183 | 964 | 1148 | 1107.576355 | 1098.140027 | 0.991480201 | 0.943788236 | unchanged |
| hsa_circ_103144 | 107 | 122 | 130 | 134 | 161 | 155 | 119.6463039 | 149.8836079 | 1.252722424 | 0.04897425 | unchanged |
| hsa_circ_103147 | 80 | 63 | 67 | 88 | 73 | 70 | 69.86510882 | 76.99511379 | 1.102053873 | 0.395290058 | unchanged |
| hsa_circ_103148 | 1399 | 2439 | 3243 | 1522 | 2733 | 2786 | 2360.488796 | 2347.358914 | 0.994437643 | 0.985410438 | unchanged |
| hsa_circ_103149 | 655 | 758 | 854 | 604 | 1003 | 1009 | 755.5315398 | 871.9472787 | 1.154084552 | 0.468986743 | unchanged |
| hsa_circ_103152 | 109 | 63 | 66 | 95 | 59 | 63 | 79.56423523 | 72.36458585 | 0.909511486 | 0.720294557 | unchanged |
| hsa_circ_103153 | 116 | 150 | 98 | 105 | 101 | 105 | 121.4720782 | 103.3825151 | 0.851080484 | 0.30525982 | unchanged |
| hsa_circ_103154 | 148 | 153 | 168 | 165 | 166 | 193 | 156.3825935 | 174.8502588 | 1.118092844 | 0.166974256 | unchanged |
| hsa_circ_103155 | 130 | 127 | 86 | 114 | 93 | 96 | 113.9897869 | 101.0044944 | 0.88608372 | 0.453608694 | unchanged |
| hsa_circ_103156 | 66 | 84 | 76 | 57 | 75 | 76 | 75.16716372 | 69.30826216 | 0.92205504 | 0.500028725 | unchanged |
| hsa_circ_103157 | 520 | 328 | 379 | 492 | 461 | 454 | 409.1048323 | 468.7503802 | 1.145795266 | 0.366298765 | unchanged |
| hsa_circ_103159 | 248 | 260 | 356 | 230 | 424 | 410 | 287.5187556 | 354.8301279 | 1.23411124 | 0.398016882 | unchanged |
| hsa_circ_103160 | 793 | 822 | 612 | 877 | 777 | 874 | 742.3451024 | 842.647918 | 1.135116155 | 0.242990995 | unchanged |
| hsa_circ_103161 | 165 | 137 | 177 | 183 | 232 | 226 | 159.8009014 | 213.6744139 | 1.337128966 | 0.048875773 | unchanged |
| hsa_circ_103162 | 561 | 464 | 416 | 443 | 345 | 358 | 480.1530549 | 381.9003632 | 0.795372141 | 0.134408603 | unchanged |
| hsa_circ_103163 | 90 | 192 | 85 | 98 | 94 | 97 | 122.36745 | 96.31869631 | 0.787126775 | 0.495863047 | unchanged |
| hsa_circ_103164 | 2397 | 6041 | 6330 | 3530 | 5004 | 4878 | 4922.718569 | 4470.748896 | 0.908186977 | 0.754710467 | unchanged |
| hsa_circ_103165 | 668 | 1430 | 1278 | 828 | 1424 | 1490 | 1125.333836 | 1247.353614 | 1.108429848 | 0.717253013 | unchanged |
| hsa_circ_103166 | 305 | 217 | 286 | 280 | 365 | 353 | 269.3918156 | 332.5733834 | 1.234534103 | 0.167806685 | unchanged |
| hsa_circ_103167 | 223 | 244 | 203 | 199 | 206 | 214 | 223.28688 | 206.395371 | 0.924350643 | 0.248551473 | unchanged |
| hsa_circ_103168 | 232 | 191 | 330 | 149 | 267 | 261 | 251.0490168 | 225.9071208 | 0.899852641 | 0.678717757 | unchanged |
| hsa_circ_103169 | 176 | 272 | 228 | 202 | 306 | 314 | 224.9273026 | 273.9703564 | 1.218039577 | 0.339972449 | unchanged |
| hsa_circ_103171 | 205 | 120 | 153 | 237 | 186 | 182 | 159.1850954 | 201.9085013 | 1.268388229 | 0.23003344 | unchanged |
| hsa_circ_103176 | 299 | 229 | 243 | 256 | 305 | 291 | 256.8662978 | 283.8827038 | 1.10517692 | 0.357166492 | unchanged |
| hsa_circ_103177 | 167 | 291 | 403 | 157 | 341 | 351 | 286.9980514 | 282.6591025 | 0.984881608 | 0.964960995 | unchanged |
| hsa_circ_103179 | 84 | 122 | 157 | 85 | 96 | 98 | 120.9490461 | 93.13499402 | 0.770034961 | 0.268519696 | unchanged |
| hsa_circ_103180 | 80 | 66 | 85 | 84 | 82 | 88 | 77.08064254 | 84.51345988 | 1.096429105 | 0.288292727 | unchanged |
| hsa_circ_103181 | 65 | 66 | 62 | 54 | 63 | 64 | 64.60403907 | 60.11785604 | 0.930558784 | 0.255372404 | unchanged |
| hsa_circ_103182 | 73 | 126 | 66 | 69 | 59 | 61 | 88.09239122 | 62.71700938 | 0.711945816 | 0.255011184 | unchanged |
| hsa_circ_103186 | 84 | 70 | 102 | 83 | 118 | 107 | 85.16748206 | 102.7603877 | 1.206568343 | 0.274089602 | unchanged |
| hsa_circ_103187 | 133 | 145 | 179 | 151 | 195 | 202 | 152.3986269 | 182.6889551 | 1.198757225 | 0.226281027 | unchanged |
| hsa_circ_103188 | 22339 | 30775 | 35496 | 35309 | 37691 | 40301 | 29536.46174 | 37766.81135 | 1.278650492 | 0.115754337 | unchanged |
| hsa_circ_103189 | 420 | 392 | 349 | 371 | 361 | 347 | 387.1997061 | 359.5661489 | 0.928632288 | 0.271415855 | unchanged |
| hsa_circ_103190 | 74 | 79 | 101 | 75 | 83 | 88 | 84.58168259 | 81.93633672 | 0.968724364 | 0.786864539 | unchanged |
| hsa_circ_103191 | 513 | 447 | 503 | 471 | 504 | 482 | 487.6844313 | 485.7866017 | 0.996108489 | 0.937385919 | unchanged |
| hsa_circ_103192 | 176 | 148 | 123 | 230 | 149 | 167 | 149.2701677 | 181.8246312 | 1.218090889 | 0.322645266 | unchanged |
| hsa_circ_103193 | 219 | 268 | 191 | 250 | 253 | 271 | 226.2246886 | 258.0532082 | 1.14069428 | 0.245973965 | unchanged |
| hsa_circ_103194 | 210 | 195 | 150 | 182 | 144 | 148 | 184.7602581 | 158.0183909 | 0.8552618 | 0.287064043 | unchanged |
| hsa_circ_103196 | 80 | 63 | 73 | 84 | 82 | 82 | 72.03759172 | 82.77394415 | 1.149038192 | 0.100194503 | unchanged |
| hsa_circ_103197 | 111 | 140 | 104 | 98 | 90 | 89 | 118.2051404 | 92.42766657 | 0.781925949 | 0.082739195 | unchanged |
| hsa_circ_103198 | 212 | 161 | 402 | 223 | 244 | 246 | 258.5430481 | 237.7345709 | 0.919516393 | 0.792088481 | unchanged |
| hsa_circ_103199 | 78 | 105 | 90 | 79 | 84 | 90 | 91.17879277 | 84.18838091 | 0.923332919 | 0.459285977 | unchanged |
| hsa_circ_103200 | 81 | 78 | 94 | 75 | 104 | 100 | 84.15589909 | 93.09440412 | 1.106213648 | 0.436717528 | unchanged |
| hsa_circ_103201 | 60 | 108 | 77 | 59 | 92 | 85 | 81.4472228 | 78.63898393 | 0.965520753 | 0.879058057 | unchanged |
| hsa_circ_103203 | 69 | 73 | 56 | 61 | 61 | 58 | 66.00285827 | 59.9881921 | 0.908872641 | 0.321498775 | unchanged |
| hsa_circ_103204 | 80 | 96 | 77 | 83 | 86 | 89 | 84.14324534 | 85.96566871 | 1.021658582 | 0.777209573 | unchanged |
| hsa_circ_103205 | 102 | 103 | 144 | 115 | 163 | 158 | 116.3724397 | 145.3551206 | 1.249051073 | 0.230009124 | unchanged |
| hsa_circ_103207 | 152 | 95 | 111 | 149 | 125 | 140 | 119.1779749 | 138.2611653 | 1.160123466 | 0.354093909 | unchanged |
| hsa_circ_103209 | 575 | 653 | 584 | 627 | 723 | 784 | 603.8020067 | 711.4044679 | 1.178208188 | 0.107119858 | unchanged |
| hsa_circ_103210 | 187 | 186 | 155 | 138 | 170 | 180 | 175.9378685 | 162.8126602 | 0.925398617 | 0.467096584 | unchanged |
| hsa_circ_103211 | 1687 | 978 | 1097 | 1079 | 786 | 790 | 1254.140111 | 884.8633918 | 0.705553856 | 0.19842167 | unchanged |
| hsa_circ_103212 | 67 | 60 | 187 | 70 | 63 | 73 | 104.7026608 | 68.79727219 | 0.657072816 | 0.433619176 | unchanged |
| hsa_circ_103213 | 24495 | 31086 | 40309 | 25338 | 33666 | 34523 | 31963.39772 | 31175.71351 | 0.975356681 | 0.891908215 | unchanged |
| hsa_circ_103215 | 169 | 226 | 124 | 149 | 120 | 125 | 172.9886932 | 131.5088893 | 0.760216675 | 0.25007395 | unchanged |
| hsa_circ_103216 | 116 | 102 | 91 | 116 | 82 | 85 | 102.8430002 | 94.45503289 | 0.918439103 | 0.560225654 | unchanged |
| hsa_circ_103218 | 608 | 467 | 540 | 507 | 383 | 395 | 538.3700234 | 428.3168721 | 0.795580834 | 0.124379459 | unchanged |
| hsa_circ_103219 | 222 | 577 | 565 | 318 | 705 | 621 | 454.8289784 | 547.9585984 | 1.204757446 | 0.60355046 | unchanged |
| hsa_circ_103220 | 270 | 320 | 239 | 265 | 277 | 299 | 276.2832775 | 280.1874338 | 1.01413099 | 0.886035538 | unchanged |
| hsa_circ_103221 | 80 | 121 | 86 | 76 | 77 | 85 | 95.80077432 | 79.17156321 | 0.826418824 | 0.273631051 | unchanged |
| hsa_circ_103222 | 84 | 147 | 75 | 81 | 70 | 74 | 102.1480591 | 75.04338705 | 0.734653088 | 0.304328778 | unchanged |
| hsa_circ_103224 | 88 | 77 | 81 | 83 | 78 | 77 | 82.26824188 | 79.59109873 | 0.967458364 | 0.517928158 | unchanged |
| hsa_circ_103227 | 68 | 87 | 75 | 67 | 81 | 75 | 76.53345196 | 74.28993484 | 0.970685797 | 0.767823788 | unchanged |
| hsa_circ_103228 | 69 | 75 | 70 | 81 | 74 | 72 | 71.14521474 | 75.76985578 | 1.065002841 | 0.229219389 | unchanged |
| hsa_circ_103229 | 133 | 86 | 108 | 153 | 145 | 135 | 109.3503236 | 144.4532289 | 1.321013273 | 0.072706246 | unchanged |
| hsa_circ_103230 | 70 | 63 | 85 | 81 | 87 | 93 | 72.68175385 | 87.09182725 | 1.198262599 | 0.127308048 | unchanged |
| hsa_circ_103231 | 131 | 139 | 142 | 130 | 156 | 160 | 137.503775 | 148.647481 | 1.08104291 | 0.328601207 | unchanged |
| hsa_circ_103232 | 253 | 177 | 208 | 226 | 244 | 238 | 212.793276 | 235.6410594 | 1.107370796 | 0.369912163 | unchanged |
| hsa_circ_103233 | 77 | 110 | 72 | 73 | 67 | 78 | 86.21030911 | 72.92103299 | 0.845850499 | 0.340428923 | unchanged |
| hsa_circ_103234 | 135 | 118 | 105 | 119 | 107 | 109 | 119.5209932 | 111.805381 | 0.935445548 | 0.453697454 | unchanged |
| hsa_circ_103236 | 148 | 152 | 197 | 127 | 193 | 172 | 165.8698487 | 163.7900696 | 0.987461379 | 0.937409938 | unchanged |
| hsa_circ_103237 | 95 | 97 | 104 | 84 | 101 | 103 | 98.39178359 | 96.06956401 | 0.976398237 | 0.743710858 | unchanged |
| hsa_circ_103238 | 1221 | 1644 | 1282 | 1189 | 1319 | 1316 | 1382.05271 | 1275.080019 | 0.92259869 | 0.484098775 | unchanged |
| hsa_circ_103239 | 118 | 163 | 88 | 136 | 95 | 98 | 122.7385348 | 109.7467236 | 0.894150511 | 0.635044841 | unchanged |
| hsa_circ_103240 | 230 | 173 | 187 | 198 | 167 | 155 | 196.6854516 | 173.3488294 | 0.881350542 | 0.334052619 | unchanged |
| hsa_circ_103241 | 96 | 81 | 137 | 91 | 141 | 140 | 104.9646681 | 123.9730306 | 1.181092961 | 0.464527949 | unchanged |
| hsa_circ_103242 | 128 | 144 | 104 | 142 | 105 | 123 | 125.2563856 | 123.329911 | 0.98461975 | 0.910400588 | unchanged |
| hsa_circ_103243 | 85 | 124 | 94 | 80 | 104 | 107 | 100.998119 | 96.92422982 | 0.959663712 | 0.792373862 | unchanged |
| hsa_circ_103246 | 265 | 375 | 361 | 328 | 518 | 522 | 333.8602255 | 455.9476597 | 1.365684274 | 0.167927918 | unchanged |
| hsa_circ_103247 | 80 | 86 | 99 | 85 | 89 | 84 | 88.41963773 | 86.00974401 | 0.972744813 | 0.703820984 | unchanged |
| hsa_circ_103248 | 379 | 513 | 427 | 325 | 366 | 367 | 439.6502034 | 352.3079222 | 0.80133688 | 0.104391895 | unchanged |
| hsa_circ_103249 | 235 | 176 | 145 | 214 | 125 | 120 | 185.2462158 | 152.7432616 | 0.824541872 | 0.466533655 | unchanged |
| hsa_circ_103250 | 483 | 489 | 580 | 495 | 369 | 370 | 517.3706494 | 411.2173055 | 0.794821481 | 0.112547236 | unchanged |
| hsa_circ_103251 | 272 | 314 | 275 | 307 | 266 | 243 | 286.8660023 | 272.1455698 | 0.948685336 | 0.559697671 | unchanged |
| hsa_circ_103252 | 795 | 877 | 863 | 814 | 1102 | 1108 | 844.8787451 | 1007.723728 | 1.192743614 | 0.17943469 | unchanged |
| hsa_circ_103253 | 109 | 106 | 141 | 112 | 145 | 152 | 118.7855773 | 136.2258871 | 1.146821779 | 0.359586921 | unchanged |
| hsa_circ_103254 | 73 | 65 | 63 | 83 | 69 | 65 | 66.86742427 | 72.56470607 | 1.085202651 | 0.425279056 | unchanged |
| hsa_circ_103255 | 278 | 414 | 349 | 263 | 359 | 400 | 346.7370708 | 340.4112577 | 0.981756167 | 0.916097887 | unchanged |
| hsa_circ_103257 | 158 | 142 | 115 | 147 | 92 | 102 | 138.2358814 | 113.9087966 | 0.824017581 | 0.314528649 | unchanged |
| hsa_circ_103258 | 98 | 117 | 125 | 97 | 137 | 140 | 113.462975 | 124.5188212 | 1.097440123 | 0.525437404 | unchanged |
| hsa_circ_103259 | 885 | 1151 | 1265 | 1234 | 1089 | 994 | 1100.141982 | 1105.790202 | 1.005134083 | 0.968015688 | unchanged |
| hsa_circ_103260 | 391 | 159 | 198 | 251 | 158 | 155 | 249.5537111 | 187.7590136 | 0.752379169 | 0.474507759 | unchanged |
| hsa_circ_103261 | 191 | 270 | 193 | 173 | 195 | 197 | 217.966627 | 188.467446 | 0.864661937 | 0.339349072 | unchanged |
| hsa_circ_103262 | 354 | 435 | 444 | 340 | 505 | 495 | 411.0408253 | 446.4608544 | 1.08617156 | 0.589798362 | unchanged |
| hsa_circ_103264 | 64 | 103 | 78 | 62 | 61 | 68 | 81.78657893 | 63.89591065 | 0.781251783 | 0.196380833 | unchanged |
| hsa_circ_103265 | 367 | 367 | 654 | 382 | 454 | 442 | 462.5925043 | 425.6543902 | 0.920149778 | 0.726161851 | unchanged |
| hsa_circ_103266 | 68 | 61 | 71 | 70 | 78 | 74 | 66.60303687 | 73.6520642 | 1.105836425 | 0.138235056 | unchanged |
| hsa_circ_103267 | 147 | 143 | 148 | 145 | 178 | 180 | 146.4249441 | 167.6196872 | 1.144748173 | 0.142094554 | unchanged |
| hsa_circ_103268 | 97 | 103 | 132 | 98 | 77 | 81 | 110.4910398 | 85.41263145 | 0.773027673 | 0.116622917 | unchanged |
| hsa_circ_103270 | 986 | 703 | 712 | 657 | 591 | 593 | 800.2572936 | 613.6740441 | 0.766845924 | 0.122060302 | unchanged |
| hsa_circ_103271 | 8344 | 12046 | 18683 | 10583 | 12386 | 12610 | 13024.28894 | 11859.62477 | 0.910577524 | 0.725535798 | unchanged |
| hsa_circ_103272 | 84 | 67 | 82 | 82 | 84 | 95 | 77.36690184 | 86.76591446 | 1.121486222 | 0.236992124 | unchanged |
| hsa_circ_103273 | 1115 | 1592 | 1736 | 1139 | 1855 | 1845 | 1481.194814 | 1613.220261 | 1.089134424 | 0.68480534 | unchanged |
| hsa_circ_103278 | 110 | 137 | 232 | 121 | 96 | 93 | 159.3463729 | 103.4561447 | 0.649253214 | 0.215023034 | unchanged |
| hsa_circ_103279 | 3395 | 1757 | 2023 | 3406 | 2006 | 1948 | 2391.50191 | 2453.133433 | 1.025771053 | 0.933720207 | unchanged |
| hsa_circ_103280 | 88 | 72 | 102 | 76 | 108 | 100 | 87.56465072 | 94.56966713 | 1.079998222 | 0.61283315 | unchanged |
| hsa_circ_103281 | 261 | 127 | 135 | 227 | 151 | 142 | 174.3051059 | 173.2778058 | 0.994106311 | 0.984900503 | unchanged |
| hsa_circ_103282 | 81 | 65 | 92 | 77 | 101 | 106 | 79.19606031 | 94.7216598 | 1.196040048 | 0.263772603 | unchanged |
| hsa_circ_103283 | 180 | 99 | 150 | 184 | 192 | 212 | 142.8626746 | 196.104889 | 1.37268107 | 0.100213165 | unchanged |
| hsa_circ_103284 | 186 | 152 | 141 | 168 | 160 | 145 | 159.4629195 | 157.6256701 | 0.988478517 | 0.909542792 | unchanged |
| hsa_circ_103285 | 7454 | 6255 | 4910 | 6400 | 5723 | 5433 | 6206.338588 | 5851.864943 | 0.942885223 | 0.676345386 | unchanged |
| hsa_circ_103287 | 399 | 339 | 287 | 355 | 296 | 304 | 341.3805502 | 318.4161191 | 0.932730699 | 0.57116278 | unchanged |
| hsa_circ_103288 | 86 | 64 | 76 | 95 | 83 | 82 | 75.45062381 | 86.75814378 | 1.149866488 | 0.210018465 | unchanged |
| hsa_circ_103289 | 95 | 106 | 69 | 96 | 80 | 79 | 89.69982905 | 85.15792632 | 0.949365536 | 0.729891664 | unchanged |
| hsa_circ_103290 | 10595 | 7326 | 6604 | 9985 | 5820 | 5991 | 8174.91827 | 7265.583698 | 0.888765301 | 0.645807015 | unchanged |
| hsa_circ_103291 | 75 | 67 | 76 | 79 | 86 | 80 | 72.58799302 | 81.28452879 | 1.119806808 | 0.075856737 | unchanged |
| hsa_circ_103293 | 86 | 63 | 83 | 91 | 90 | 101 | 77.21362037 | 93.97963495 | 1.217138045 | 0.107440638 | unchanged |
| hsa_circ_103295 | 73 | 91 | 106 | 75 | 98 | 98 | 90.16922849 | 90.03394628 | 0.998499685 | 0.991753193 | unchanged |
| hsa_circ_103296 | 114 | 141 | 194 | 113 | 145 | 149 | 149.6634363 | 135.3396606 | 0.90429342 | 0.611818408 | unchanged |
| hsa_circ_103297 | 122 | 180 | 172 | 134 | 184 | 199 | 158.0233788 | 172.3579945 | 1.090711994 | 0.620057875 | unchanged |
| hsa_circ_103298 | 70 | 99 | 196 | 60 | 58 | 56 | 121.3738382 | 57.85392525 | 0.476658942 | 0.171394575 | unchanged |
| hsa_circ_103299 | 350 | 463 | 476 | 370 | 493 | 553 | 429.5563884 | 471.7349274 | 1.098190925 | 0.563871463 | unchanged |
| hsa_circ_103300 | 344 | 417 | 334 | 361 | 345 | 315 | 365.0794456 | 340.2225593 | 0.931913761 | 0.445381176 | unchanged |
| hsa_circ_103302 | 60 | 77 | 56 | 57 | 64 | 69 | 64.35666385 | 63.48155984 | 0.986402278 | 0.908574377 | unchanged |
| hsa_circ_103303 | 49 | 123 | 55 | 48 | 52 | 53 | 75.98654588 | 51.24917849 | 0.674450692 | 0.356647088 | unchanged |
| hsa_circ_103306 | 114 | 91 | 87 | 105 | 92 | 96 | 97.34822 | 97.70755901 | 1.003691275 | 0.970454788 | unchanged |
| hsa_circ_103307 | 1967 | 1774 | 1474 | 2424 | 1219 | 1249 | 1738.667463 | 1630.378897 | 0.937717494 | 0.810077653 | unchanged |
| hsa_circ_103308 | 133 | 75 | 123 | 130 | 142 | 136 | 110.2567511 | 135.9307917 | 1.232856857 | 0.232319581 | unchanged |
| hsa_circ_103309 | 1224 | 1708 | 1545 | 1475 | 1356 | 1363 | 1492.508123 | 1398.068538 | 0.93672424 | 0.556142534 | unchanged |
| hsa_circ_103310 | 106 | 56 | 80 | 107 | 113 | 110 | 80.50048382 | 109.9255211 | 1.365526216 | 0.112318432 | unchanged |
| hsa_circ_103312 | 77 | 55 | 105 | 84 | 107 | 115 | 78.77938163 | 101.8321862 | 1.292624848 | 0.250841493 | unchanged |
| hsa_circ_103313 | 72 | 59 | 61 | 68 | 69 | 76 | 64.13456824 | 70.99355677 | 1.106946826 | 0.218729486 | unchanged |
| hsa_circ_103314 | 75 | 75 | 157 | 77 | 84 | 71 | 102.2601518 | 77.40159822 | 0.756908697 | 0.41529531 | unchanged |
| hsa_circ_103315 | 151 | 140 | 171 | 127 | 137 | 128 | 154.0649325 | 130.8120902 | 0.849071155 | 0.069572226 | unchanged |
| hsa_circ_103316 | 5207 | 12030 | 11171 | 5935 | 10642 | 10971 | 9469.303836 | 9182.472266 | 0.969709329 | 0.920291962 | unchanged |
| hsa_circ_103318 | 134 | 93 | 119 | 149 | 140 | 174 | 115.6724654 | 154.5312819 | 1.335938344 | 0.068252895 | unchanged |
| hsa_circ_103319 | 87 | 68 | 108 | 104 | 116 | 142 | 87.87150186 | 120.9856521 | 1.376847437 | 0.108452214 | unchanged |
| hsa_circ_103321 | 68 | 59 | 207 | 53 | 52 | 48 | 111.4340335 | 50.8142721 | 0.456003166 | 0.275722561 | unchanged |
| hsa_circ_103322 | 668 | 602 | 400 | 506 | 396 | 392 | 556.8701821 | 431.3426211 | 0.774583799 | 0.231012782 | unchanged |
| hsa_circ_103325 | 256 | 230 | 187 | 233 | 173 | 173 | 224.0455341 | 192.8731683 | 0.860865935 | 0.333995821 | unchanged |
| hsa_circ_103327 | 102 | 126 | 119 | 105 | 96 | 99 | 115.8645107 | 100.1151572 | 0.864070945 | 0.10630358 | unchanged |
| hsa_circ_103329 | 502 | 309 | 290 | 483 | 340 | 350 | 366.8935704 | 390.9208234 | 1.065488346 | 0.78432057 | unchanged |
| hsa_circ_103331 | 80 | 56 | 72 | 77 | 90 | 89 | 69.02297294 | 85.14836594 | 1.233623565 | 0.120638305 | unchanged |
| hsa_circ_103333 | 64 | 70 | 59 | 55 | 65 | 63 | 64.21767416 | 61.19012365 | 0.952854871 | 0.540923977 | unchanged |
| hsa_circ_103334 | 157 | 85 | 69 | 132 | 76 | 71 | 103.5735728 | 93.05344335 | 0.898428439 | 0.768967251 | unchanged |
| hsa_circ_103335 | 72 | 77 | 569 | 79 | 106 | 91 | 239.3065672 | 91.68543686 | 0.383129631 | 0.421671214 | unchanged |
| hsa_circ_103337 | 78 | 72 | 80 | 80 | 76 | 79 | 77.01648412 | 78.43608102 | 1.018432377 | 0.640679627 | unchanged |
| hsa_circ_103338 | 98 | 139 | 337 | 88 | 86 | 83 | 191.5481255 | 85.85727194 | 0.448228202 | 0.225002842 | unchanged |
| hsa_circ_103339 | 578 | 692 | 644 | 735 | 586 | 587 | 637.9578045 | 635.8702287 | 0.996727721 | 0.973703743 | unchanged |
| hsa_circ_103340 | 312 | 391 | 344 | 335 | 321 | 294 | 349.0816204 | 316.9918749 | 0.908073804 | 0.282501878 | unchanged |
| hsa_circ_103341 | 253 | 247 | 270 | 253 | 258 | 232 | 256.4126342 | 247.8338522 | 0.96654306 | 0.458828323 | unchanged |
| hsa_circ_103342 | 451 | 251 | 247 | 377 | 292 | 285 | 316.1534007 | 317.969549 | 1.005744516 | 0.981489254 | unchanged |
[truncated: 181,672 more chars]
